# Single-cell RNA sequencing reveals a novel inhibitory effect of ApoA4 on NAFL mediated by liver-specific subsets of myeloid cells

**DOI:** 10.3389/fimmu.2022.1038401

**Published:** 2022-11-08

**Authors:** Xiao-Huan Liu, Jin-Ting Zhou, Chun-xia Yan, Cheng Cheng, Jing-Na Fan, Jing Xu, Qiangsun Zheng, Qiang Bai, Zongfang Li, Shengbin Li, Xiaoming Li

**Affiliations:** ^1^ National & Local Joint Engineering Research Center of Biodiagnosis and Biotherapy, Precision Medical Institute, The Second Affiliated Hospital, Xi’an Jiaotong University, Xi’an, China; ^2^ Key laboratory of Ministry of Public Health for Forensic Sciences, Western China Science & Technology Innovation Harbour, Xi’an, China; ^3^ College of Forensic Medicine, Xi’an Jiaotong University Health Science Center, Xi’an, China; ^4^ Department of Pathology, Bio-Evidence Sciences Academy, The Western China Science and Technology Innovation Port, Xi’an Jiaotong University, Xi’an, China; ^5^ Division of Endocrinology, The Second Affiliated Hospital, Xi’an Jiaotong University, Xi’an, China; ^6^ Division of Cardiology, The Second Affiliated Hospital, Xi’an Jiaotong University, Xi’an, China; ^7^ Laboratory of Immunophysiology, GIGA Institute, Liège University, Liège, Belgium

**Keywords:** ApoA4, NAFLD, single cell RNA sequencing, immunity, macrophage

## Abstract

The liver immune microenvironment is a key element in the development of hepatic inflammation in NAFLD. *ApoA4* deficiency increases the hepatic lipid burden, insulin resistance, and metabolic inflammation. However, the effect of ApoA4 on liver immune cells and the precise immune cell subsets that exacerbate fatty liver remain elusive. The aim of this study was to profile the hepatic immune cells affected by ApoA4 in NAFL. We performed scRNA-seq on liver immune cells from WT and *ApoA4*-deficient mice administered a high-fat diet. Immunostaining and qRT–PCR analysis were used to validate the results of scRNA-seq. We identified 10 discrete immune cell populations comprising macrophages, DCs, granulocytes, B, T and NK&NKT cells and characterized their subsets, gene expression profiles, and functional modules. *ApoA4* deficiency led to significant increases in the abundance of specific subsets, including inflammatory macrophages (2-Mφ-Cxcl9 and 4-Mφ-Cxcl2) and activated granulocytes (0-Gran-Wfdc17). Moreover, *ApoA4* deficiency resulted in higher *Lgals3*, *Ctss*, *Fcgr2b*, *Spp1*, *Cxcl2*, and *Elane* levels and lower *Nr4a1* levels in hepatic immune cells. These genes were consistent with human NAFLD-associated marker genes linked to disease severity. The expression of NE and IL-1β in granulocytes and macrophages as key ApoA4 targets were validate in the presence or absence of ApoA4 by immunostaining. The scRNA-seq data analyses revealed reprogramming of liver immune cells resulted from *ApoA4* deficiency. We uncovered that the emergence of ApoA4-associated immune subsets (namely Cxcl9^+^ macrophage, Cxcl2^+^ macrophage and Wfdc17^+^ granulocyte), pathways, and NAFLD-related marker genes may promote the development of NAFL. These findings may provide novel therapeutic targets for NAFL and the foundations for further studying the effects of ApoA4 on immune cells in various diseases.

## Introduction

Epidemiological evidence shows that nearly a quarter of the global population has nonalcoholic fatty liver disease (NAFLD) (1). NAFLD includes nonalcoholic simple fatty liver (NAFL) and nonalcoholic steatohepatitis, which can deteriorate to hepatocirrhosis and NAFLD-related hepatocellular carcinoma (HCC) (1).

Previous studies have shown that inflammation is crucial to the development of obesity and NAFLD (2). The innate immune system is the major factor in supporting hepatic inflammation in NAFLD (2). Activation of macrophages and neutrophils plays a key role in NAFLD, and depletion of one of these cell types can attenuate or even alleviate insulin resistance and inflammatory development (3, 4). T cells and B cells are also involved in sustaining NAFLD progression (2). There are also strictly controlled interactions between innate and adaptive cells during the pathogenesis of fatty liver disease. In fact, cytotoxic T cells stimulate hepatic macrophage activation, and in turn, macrophages support lymphocyte functions through the release of a variety of cytokines (such as CXCL9 and CXCL10) (5).

Apolipoprotein A4 (ApoA4) is an apolipoprotein that contains 376 amino acids and is primarily expressed in the intestine and liver in response to fat absorption (6). Population-based cohorts showed that SNPs of *ApoA4* gene lead to differential ApoA4 concentrations in plasma (7), which is related to obesity, T2DM, renal impairment in T2DM patients. Therefore, ApoA4 was considered as a therapeutic target for arthrosclerosis, and a predictor of cardiovascular risk in human populations (8–10). It was reported that hepatic steatosis was more pronounced in *ApoA4* knockout rats and mice than wildtype control (11, 12). ApoA4 is known to regulate glucose homeostasis and lipid metabolism and reduce susceptibility to atherogenesis (6). ApoA4 was also shown to alleviate oxidative stress and inflammation and can modulate hepatic immune cells such as macrophages and dendritic cells (DCs) in the context of acute and chronic liver damage (6).

Single-cell RNA sequencing (scRNA-seq) is a new next-generation sequencing technology that supports more powerful analyses with a high resolution that conventional bulk-RNA sequencing cannot reach, which allows the identification of cell subset-, cell function-, and cell-type-specific transcriptome differences in complex tissues, such as liver tissue, at a high resolution. Recent scRNA-seq analysis of diet-induced NASH mouse livers revealed that the emergence of Trem2^+^ NASH-associated macrophages is one of the conserved features of mouse and human NASH (13) and osteopontin-expressing recruited macrophages distinct from kupffer cells in the fatty liver (4). In contrast to NASH, which is accompanied by liver fibrosis, NAFL is reversible. Therefore, it is of great significance to uncover the causes of NAFL exacerbation in order to reduce severity or reverse the condition through intervention. Utilization of *ApoA4*-deficient mice fed a high-fat diet (HFD) enabled us to simulate the changes in the liver immune system during NAFLD exacerbation and explore the mechanisms by which ApoA4 affects NAFL through hepatic immune cells.

After founding the restriction of ApoA4 on diet-induced hepatic steatosis *via* SREBF1-mediated lipogenesis (12), meanwhile, we hypothesized that regulation of ApoA4 on hepatic immune cells involved in the pathogenesis of NAFL. In this study, we used scRNA-seq to profile the single-cell gene expression pattern of immune cells and to explore the heterogeneity, intercellular crosstalk, and cellular functions of macrophage and granulocyte in NAFL. We defined the identities of the major myeloid populations and lymphocytes observed in response to ApoA4, thereby illuminating the underlying mechanism by which *ApoA4* knockout aggravates fat accumulation in the liver.

## Materials and methods

### NAFL animal model with *ApoA4* gene loss and gene gain

Five-week-old male C57BL/6 wide type mice were purchased from the Experimental Animal Center of Xi’an Jiaotong University, China. *ApoA4* knockout (global knockout) mice were kindly provided by Professor Patrick Tso. All the above animals were bred under specific pathogen–free conditions with a 12-h light-dark. Our animal experiments were approved by the laboratory animal care committee of Xi’an Jiaotong University. For diet-induced NAFL, two groups of mice, KO group and WT group (as the control), were fed a diet containing 60 kcal% fat (Research Diets Inc., New Brunswick, New Jersey, USA) for 16 weeks. For *in vivo ApoA4* overexpression, KO group of mice were infected adeno-associated viruses (AAVs)-*GFP* (KO-GFP group as control) and AAV-*ApoA4*-*GFP* (KO-ApoA4-GFP group) with the TBG hepatic expression promoter (BrainVTA, Development Zone, Wuhan, China). The viruses were diluted to 2×10^10^ vg/ml in 100 μL normal saline (per mouse) and injected intravenously into *ApoA4* KO mice at 5 weeks of age. In addition, all mice were fed a HFD for 16 weeks.

### Liver immune cell isolation

After anesthesia, the primary liver cells were isolated as described previously (14). Briefly, the mouse liver was perfused with Hanks (ZHONGHUIHECAI, Xi’an, China) solution with 0.5 M EDTA (ZHONGHUIHECAI, Xi’an, China) through the left ventricle until the liver turned white (approximately 6 minutes). The liver tissue was minced and digested with PSCeasy dispase (Cellapy, Beijing, China), disassociated by pipetting every 20 minutes, and then swiftly ground on a 40 µm filter (FALCON, One Riverfront Plaza, NY, USA). The filtered product was centrifuged at 50 g for 3 min to remove hepatocytes and collect the immune cells in the supernatant. The cells were lysed with red blood cell lysate (CWBIO, Beijing, China) on ice for 2 minutes and washed once with PBS. Subsequently, the isolated hepatic living immune cells were stored in liquid nitrogen. Then the cells were recovered, dead cells were removed before the single cell was separated and cDNA libraries for each cell were established by loading on 10x Genomics platform for scRNA-seq analysis or the recovered cells were analyzed for quantitative reverse transcription polymerase chain reaction (qRT–PCR).

### ScRNA-seq and data analysis

Liver immune cells isolated from WT and KO mice (n=3/group) were subjected to scRNA-seq. Briefly, The construction of a cDNA library were performed with the 10x Genomics platform, as described previously (15). Library sequencing was performed by Illumina NovaSeq using PE150 protocol. Over 0.77 billion reads and 46356 reads per cell were obtained. STAR was used with default parameters to map sequences from the raw data to the mouse reference genome, and approximately 54.1% of the sequence reads could be mapped. Further analysis was then performed with the Seurat package (v4.0). After removing cells that expressed fewer than 500 genes and contained greater than 5% mitochondria, 16,856 cells from 6 livers in the WT and KO groups were used for further analysis. The alignment method was used to integrate two NAFL datasets (WT and KO), and the 35 most significant principal components were chosen to perform cluster identification. Then, the appropriate cluster numbers determination using a method controlled by a resolution parameter was based on a resolution of 2.6, which produced 10 clusters. The identity of each cluster was determined based on marker genes, which were evaluated by comparing differential expression between the cells in one cluster and all other cells using a Fisher test with a 10% cut-off. GO, KEGG and GSEA were performed with clusterProfiler (16, 17).

The interaction between a receptor and its ligand is a classic method of intercellular crosstalk in organ biology. CellChat (18) is a method that can infer, visualize and analyze cell–cell communication from scRNA-seq data through network analysis and pattern recognition approaches. We used this method to build a global network of communication among cells.

### QRT–PCR analysis

The liver immune cells were isolated as described above. Total RNA from liver immune cells was extracted with RNAiso Plus (Takara, Dalian, China) according to the manufacturer’s instructions and reverse transcribed into cDNA (Takara, Dalian, China). QRT–PCR was performed using the SYBR Green PCR Master Mix kit (Takara, Dalian, China) with the primers listed in **Table S1**.

### Immunohistochemistry

The murine and human NAFL liver tissues were fixed with formalin and embedded in paraffin. Antigen retrieval was performed by placing the tissue sections in a repair box filled with boiling citric acid (pH 6.0) antigen retrieval buffer. The sections were stained with a polyclonal neutrophil elastase antibody (1:400 dilution; Thermo Fisher Scientific, Shanghai, China). IHC was performed on representative serial sections of liver tissue samples from human NAFL and health people as control. The diagnosis of NAFL was mainly established by pathological examination which showing steatosis but without feature of steatohepatitis and other chronic liver disease were excluded. We divided these patients into mild and moderate NAFL according to fat deposition. The sections were stained with CTSS antibody (1:400 dilution; ABclonal, Wuhan, China), CD48 antibody (1:300 dilution, Cell Signaling Technology, Danvers, Massachusetts, USA), and Galectin-3 antibody (1:300 dilution; Proteintech, Rosemont, IL, USA) with the nuclei counterstained with haematoxylin (Servicebio, Wuhan, China). The sections were scanned with Pannoramic DESK (3D HISTECH, Budapest, Hungary). The browser software Caseviewer (C. V 2.3) was used to acquire images.

### Immunofluorescence staining

Liver tissues were fixed with 4% paraformaldehyde and then subjected to paraffin embedding and sectioning. Briefly, the paraffin sections were recovered with EDTA antigen retrieval solution (Servicebio, Wuhan, China) and blocked in 5% BSA for 30 min. The mouse liver sections were then incubated with primary antibody at 4°C overnight and incubated with IL-1β (1:800 dilution; Servicebio) for 50 min with nuclei stained with DAPI (Servicebio, Wuhan, China). Finally, the sections were sealed with antifade mounting medium. The sections were scanned with Pannoramic DESK and Caseviewer was used to acquire images.

### Statistical analysis

We used GraphPad Prism 6.01 to calculate the statistical significance of differences in histological data and qRT–PCR and microarray expression values using analysis of variance (ANOVA) at a 95% confidence level or Student’s t test. *P* < 0.05 was considered to indicate statistical significance.

## Results

### ApoA4 deficiency activated myeloid cell-related functions profiled by scRNA-seq in the NAFL mouse liver

**Figure 1:**
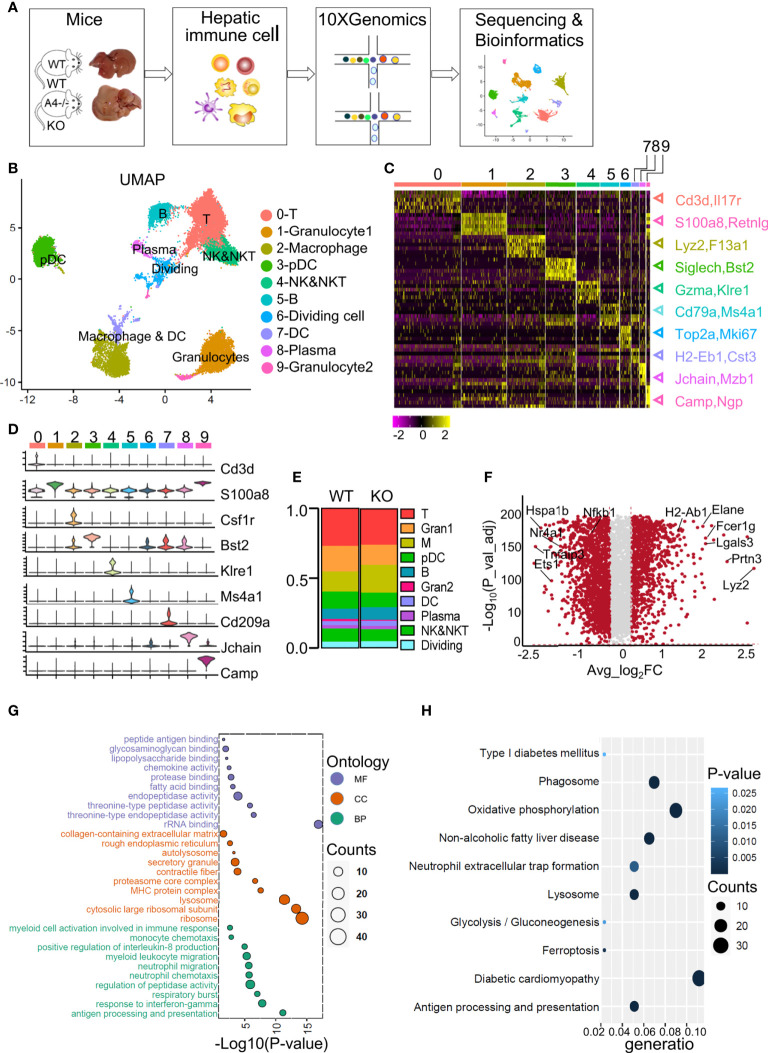
ScRNA-Seq profiles of liver immune cell populations and the differences between WT and *ApoA4* KO mice. **(A)** Schematic diagram of hepatic immune cell isolation and data analysis using the 10X genomics platform. **(B)** UMAP visualization of hepatic immune cells showing 10 clusters. **(C)** Heatmap of the 6 most strongly upregulated genes in each cluster (genes were decreased by *P value*). **(D)** Violin plots showing specific marker expression in each cluster. **(E)** The proportion of each type of immune cell in the WT and KO groups is shown in **(B)**. **(F)** Volcano plot showing differentially expressed genes (DEGs) between WT and KO mice. Average_log_2_ fold change > 0.2 & -log_10_ (*P* value-adjust) > 2 are red; others are grey. **(G)** Gene Ontology (GO) enrichment analysis of molecular function (MF), cellular component (CC), and biological process (BP) for the upregulated genes of KO mice vs. WT mice. **(H)** Kyoto Encyclopedia of Genes and Genomes (KEGG) analysis of upregulated genes in KO mice vs. WT mice.

KO mice and WT mice (as control) were fed a HFD for 16 weeks to establish a NAFL model, and body weight (weekly) and IPGTT (before sacrifice) were recorded and used to evaluate this model (12). Both WT mice and *ApoA4* knockout mice had increased body weight and decreased glucose tolerance, indicating that the NAFL model was successfully established and that KO mice showed more severely impaired glucose tolerance (12, 19). As expected, the KO mice gained more body weight and exhibited hepatic fat accumulation (12). To explore the pathogenic mechanisms underlying *ApoA4* regulation of liver steatosis through liver immune cells, we performed scRNA-seq to profile the reprogramming of the liver immune microenvironment from the two groups of mice. Hepatic immune cells were isolated, and a cDNA library was built and sequenced by 10X Genomics (**Figure 1A**). After integration and filtering of low-quality data, we obtained 16,856 hepatic immune cells from WT and KO mice.

We used UMAP to visualize the data and revealed 10 clusters **(** **Figure 1B**). The cell types were annotated based on the expression of specific markers (**Table S2**). T cells (identified by *Cd3d* and *Il7r* expression (15)) represented 26.9% and 25.7% of all immune cells in WT and KO mice respectively (**Figures 1B, C, E**). The NK&NKT cluster (identified by *Gzma* and *Klre1* expression (15)) was adjacent to the T cells (**Figures 1B, C**). The fact that macrophages (identified by *Csf1r*, *Lyz2* and *F13a1* expression (20)) and DCs (identified by *H2-Eb1* and *Cst3* expression (20)) were clustered together indicated the similarity of their gene expression patterns. Other cell types or special cell states were also characterized, such as granulocyte1 *(S100a8*, *Retnlg*), granulocyte2 (*Camp*, *Npg*) (20), plasmacytoid dendritic cells (pDCs) (*Bst2*, *Siglech*) *(* 15), B cells (*Cd79b*, *Ms4a1*) (13), plasma cells (*Jchain*, *Mzb1*) (13), and dividing cells (*Mki67*, *Top2a*) (13) (**Figures 1B, C**), and the expression of their marker genes were showed in the heatmap and violin plots (**Figures 1C, D**; **Tables S2**; **S3**). Compared with WT mice, KO mice harboured a relatively higher proportion of macrophages, DCs, B cells, and plasma cells (**Figure 1E**). The proportions of T cells, NK&NKT cells, granulocytes, and pDCs were smaller than those in the WT group (**Figure 1E**). We used volcano plot to show the differentially expressed genes (DEGs) among all analysed immune cells between the WT and KO groups (**Figure 1F**; **S1A**). Gene ontology (GO) and Kyoto Encyclopedia of Genes and Genomes (KEGG) analyses were performed on the genes that were upregulated in the KO group (**Figures 1G, H**; **Table S4**). These genes were mainly involved in myeloid cell-related functions, such as antigen processing (*Fcer1g*, *H2-Ab1*), threonine-type peptidase activity (*Elane*, *Prtn3*), and neutrophil migration (*Lgals3*, *Cxcl2*) (**Figure 1G**; **S1A**). The top KEGG pathways revealed that the genes upregulated in the KO group were highly enriched in glucolipid metabolism-related pathways, such as NAFLD and neutrophil extracellular trap (NET) formation (*Elane*) (**Figure 1H**). The GO and KEGG results were consistent with the Gene Set Enrichment Analysis (GSEA) analysis (**Figure S1B**). These results indicate that almost all types of liver-resident immune cells were obtained in this scRNA-seq study and that ApoA4 mainly influences myeloid cell number and function. Therefore, we analyzed macrophages, DCs and granulocytes.

### ApoA4 deficiency increased inflammatory subclusters of macrophages and DCs in NAFL mice

**Figure 2:**
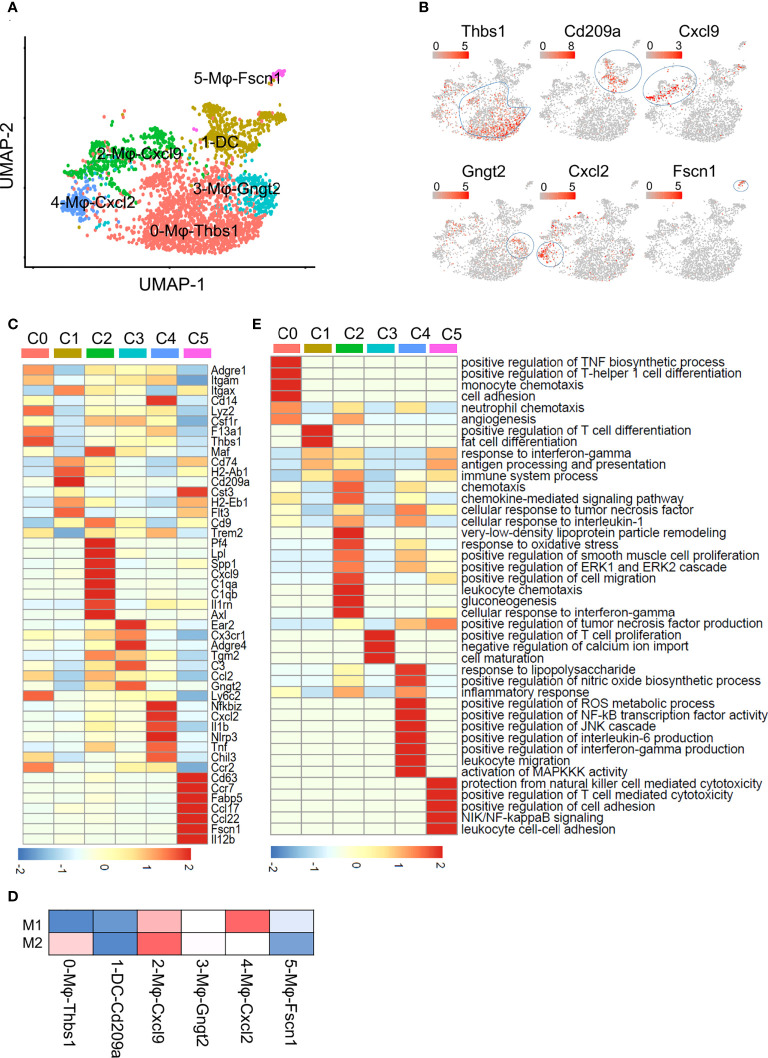
Subclustering of macrophages and DCs. **(A)** UMAP plot of liver macrophages and DCs showing a six-cluster distribution. **(B)** Feature plots of marker gene expression in six clusters. **(C)** Heatmap showing the marker genes of each cluster mentioned in **(A)**. **(D)** Gene enrichment for classical cell type M1 and M2 in comparisons with the macrophage and DCs subsets in NAFL. **(E)** GO BP terms enriched in the indicated subset. The gene list is the marker genes of each cluster, colored by -log_10_ (*P value*), scaled by row.

The expression matrix of DCs and macrophages was extracted for further analysis. They were divided into 6 subpopulations, which were named according to the marker genes of each subpopulation (**Figures 2A, B**). Cluster 0, 0-Mφ-Thbs1, was a kind of monocyte-derived macrophages (MDMs) that highly expressed the *Thbs1*, *F13a1*, *Lyz2*, *Ly6c2*, *Ccr2* and *Fn1* genes and showed low expression of the *Itgax* (encoding CD11C) gene (**Figure 2C**; **Tables S2, S5**). This subset tended to be myeloid-derived suppressor cells because of the high expression of *Thbs1* (21). 3-Mφ-Gngt2, adjacent to 0-Mφ-Thbs1 is a group of macrophages derived from monocytes for highly expressed *Cx3cr1* (22) (**Figure 2C**). This cluster was characterized by specific expression of the common macrophage markers *Adgre4* (encoding EMR4) (23), *Tgm2*, *C3*, and *Ear2* (**Figure 2C**; **Tables S2**; **S5**). *Tgm2* was associated with wound healing, fibrosis, and metastasis. *Ear2* is upregulated in anti-inflammatory macrophages (24), while complement C3 expressed by *Cx3cr1^+^* macrophages suppresses the infiltration and function of CD8^+^ T cells and CD4^+^ T cells (25). DCs expressed high levels of the *Cd74*, *Cd209a* and *H2-Ab1* genes (**Figure 2C**; **Tables S2**; **S5**). Cluster 2-Mφ-Cxcl9 was a group of macrophages that are closely related to NAFLD (26), specifically expressing the inflammatory cytokines *Cxcl9*, *Cxcl10*, *Isg15*, *Lpl* and *Il1rn* (27) (**Figure 2C**; **Tables S2**; **S5**). *Lpl*, which aggravates inflammation and atherosclerosis, is a marker gene of unique Kupffer cells in NASH (26). This cluster was considered as Kupffer cell (KC) with high expression of *Adgre1* (encoding F4/80) and high expression of KC lineage-determining transcription factors Maf, and KC-specific genes C1qa, as well as the low expression of Itgam (encoding CD11b) (**Figure 2C**). 4-Mφ-Cxcl2 highly expressed markers such as the *Tnf*, *Nfkbiz*, *Il1b* and *Tnfsf9* genes that are enriched in inflammatory macrophages (20) (**Figure 2C**; **Table S2**). The upregulated *Tnfsf9* and *Nlrp3* genes in this cluster can mediate M1 macrophage polarization (20). Genes upregulated in 4-Mφ-Cxcl2 were enriched for signatures of M1 macrophages, and those in 2-Mφ-Cxcl9 simultaneously resembled the signatures for M1 and M2 macrophages (**Figure 2D**). The last cluster, 5-Mφ-Fscn1, showed higher *Ccr7*, *Fscn1*, *Il12b*, *Fabp5*, and *Ccl22* expression (**Figure 2C**; **Table S2**). FABP5 inhibits liver macrophage differentiation to the M2 phenotype and plays an important role in the occurrence and development of diabetes and atherosclerosis (28).

To better understand the functional heterogeneity of macrophages and DCs, we performed GO term analyses of these six clusters (**Figure 2E**; **Table S5**). Some biological processes overlapped for similar macrophage lineages, including processes related to inflammatory responses and neutrophil chemotaxis in 0-Mφ-Thbs1, 2-Mφ-Cxcl9, and 4-Mφ-Cxcl2; regulation of the TNF biosynthetic process in clusters 2-Mφ-Cxcl9, 4-Mφ-Cxcl2 and 5-Mφ-Fscn1; and positive regulation of the ERK1 and ERK2 cascade shared in 2-Mφ-Cxcl9 and 4-Mφ-Cxcl2 (**Figure 2E**). However, each subset was also enriched in particular functions not found in the other populations, such as gluconeogenesis in 2-Mφ-Cxcl9, monocyte chemotaxis in 0-Mφ-Thbs1, and positive regulation of NF-κB transcription factor activity in 4-Mφ-Cxcl2 (**Figure 2E**). The function-related gene expression in macrophage and DC subsets is also shown in **Figure S2C**.

The pseudotime trajectory (Monocle2) of the four macrophage subsets revealed that the cells in 0-Mφ-Thbs1 were located at the beginning of the trajectory, and 2-Mφ-Cxcl9 showed a trajectory similar to that of 4-Mφ-Cxcl2 from a to b, while 3-Mφ-Gngt2 lay in a different direction (**Figure S2A**). Cells in the fifth cluster were found at the end of the trajectory (**Figure S2A**). A heatmap of the top 50 differentially expressed genes in the analyzed macrophages was shown in **Figure S2B**. 0-Mφ-Thbs1 were relatively naïve cells, while the cells of other groups were in a mature state of differentiation. The differentiation trajectories of 2-Mφ-Cxcl9 and 4-Mφ-Cxcl2 were similar, suggesting that although the expression profiles of the two groups of cells were inconsistent, they might have similar functions.

**Figure 3:**
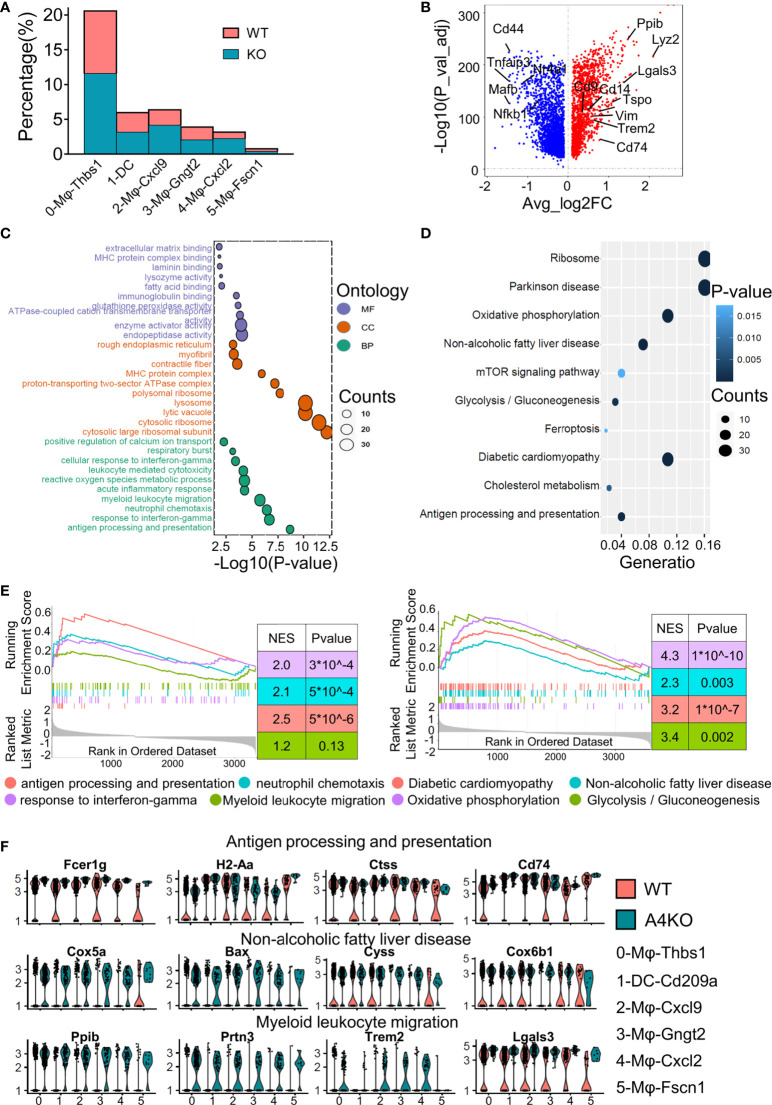
Gene expression heterogeneity and gene expression upregulation by *ApoA4* knockout in macrophages and DCs. **(A)** Fraction of each macrophage and DC subset in WT and KO mice. **(B)** Volcano plot showing DEGs in macrophages and DCs between WT and KO mice. Genes upregulated in WT are shown in blue. GO **(C)** and KEGG **(D)** analyses, respectively, with the gene set in **(B)**, cut-off = 0.5. **(E)** GSEA plots of enriched pathways upregulated in KO macrophage and DC subsets. **(F)** Violin plots showing the expression of function-related genes in **(C, D)** across six groups.

The KO group contributed to a larger proportion of cells in all macrophage subsets (**Figure 3A**), especially 2-Mφ-Cxcl9 and 4-Mφ-Cxcl2. To further elucidate the effect of ApoA4 on macrophages and DCs, we analyzed the upregulated genes in KO macrophages and DCs (**Figure 3B**; **Table S6**) by GO and KEGG. *ApoA4* deletion resulted in the upregulation of genes in the pathways of neutrophil chemotaxis, myeloid leukocyte migration, NAFLD, and antigen processing and presentation (**Figures 3C–F**), which was consistent with the enriched pathways in the whole population of hepatic immune cells. These results suggest that liver-resident macrophages, especially inflammatory macrophages (M1 macrophages), exhibit both marked amplification and functional plasticity, implying that they are the key contributors during the exacerbation of NAFL resulting from *ApoA4* deletion.

### ApoA4 deletion increased mature neutrophils and lost its naive granulocytes in NAFL mice

**Figure 4:**
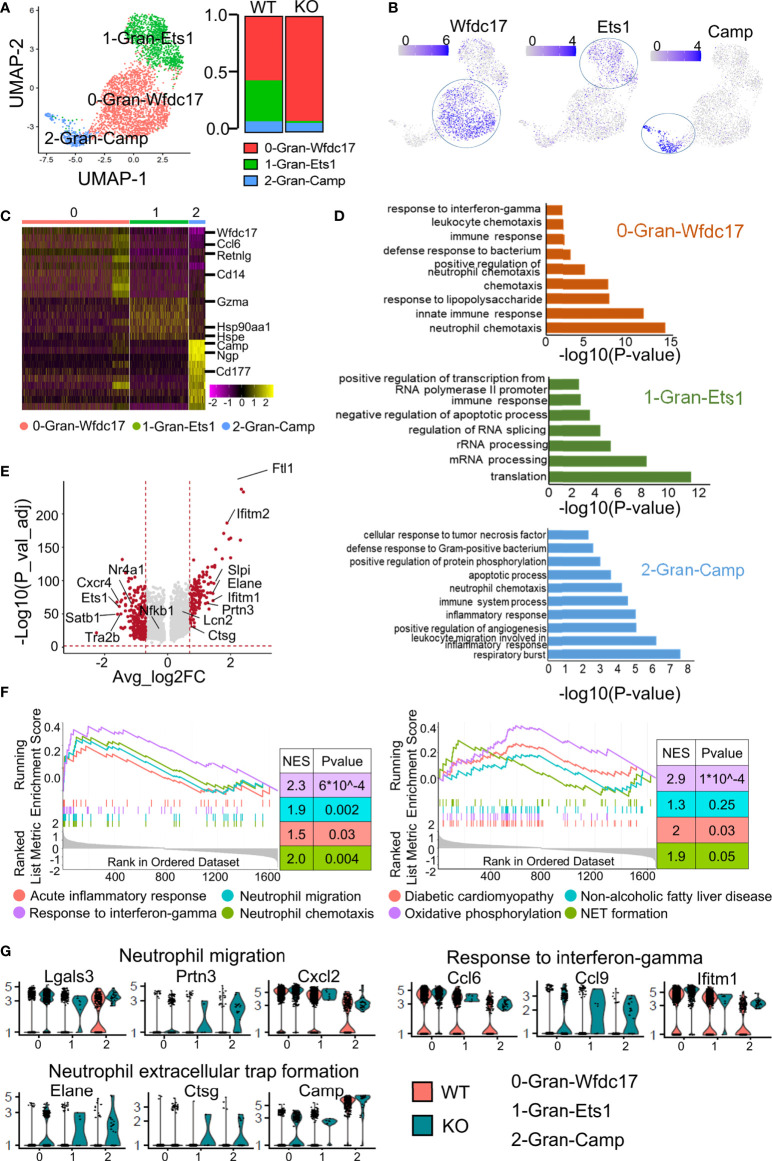
Subclustering of granulocytes revealed more effector neutrophils in the *ApoA4* knockout group. **(A)** UMAP plot of liver granulocytes showing a three-cluster distribution; fraction of each granulocyte subset in the WT and KO. **(B)** Feature plots of marker gene expression in three subsets. **(C)** Heatmap of the 6 most upregulated genes (decreased by *P value*) among the three granulocyte subsets. **(D)** GO BP terms enriched in the indicated subsets; the gene list is the maker genes of each cluster. **(E)** Volcano plot showing upregulated genes in WT and KO granulocytes. Average_log_2_ fold change > 0.7 & -log_10_(*P* value-adjust) > 2 are red; others are grey. **(F)** GSEA plot of 8 MSigDB hallmark gene sets. **(G)** Violin plots showing the expression of genes involved in neutrophil migration, response to interferon-gamma, and NET formation pathways among granulocyte subsets.

We divided the granulocytes into subsets to explore their heterogeneity *in vivo* and the role of ApoA4 in the gene expression profile of granulocytes (**Figure 4A**). 0-Gran-Wfdc17 was a kind of neutrophil that mainly expresses *Wfdc17*, *Ccl6*, *Il1b* and a set of interferon-stimulated genes (e.g., *Ifitm2* and *Ifitm3*) (29) (**Figures 4B, C**; **Table S2**). This cluster also expressed high levels of *Cxcl2*, which is important for neutrophil mobilization (30). 2-Gran-Camp, a kind of neutrophil, was characterized by high expression of *Camp*, *Ngp*, *Ltf*, and *Chil3* (**Figures 4B, C**; **Table S2**). The second cluster, 1-Gran-Ets1, almost completely disappeared in *ApoA4*-deficient mice and showed high expression of *Ets1*, *Foxp1*, and *Hsp90aa1* (**Figures 4B, C**; **Table S2**); these could be granulocyte-macrophage progenitors, which typically express *Irf8* and *Cebpa* (31).

As shown in **Figure 4A**, the proportion of 0-Gran-Wfdc17 (representing the major neutrophils) was higher in the KO group. The marker genes were enriched in neutrophil chemotaxis, innate immune response, and response to interferon-gamma (**Figure 4D**; **Table S7**). No cell proportion difference was found between the two groups in 2-Gran-Camp with marker genes involved in the respiratory burst, leukocyte migration, and inflammatory response (**Figures 4A, D**). The markers of 1-Gran-Ets1 were enriched in the function of mRNA processing and translation (**Figure 4D**). Further pseudotime analysis of two kinds of neutrophils (0-Gran-Wfdc17 and 2-Gran-Camp) with Monocle2 showed that (**Figure S3**) cell development begins with 2-Gran-Camp and ends with 0-Gran-Wfdc17. This was consistent with the result published by Xie et al. (30), implying that 0-Gran-Wfdc17 were a set of more mature cells.

GO analysis showed that *ApoA4* deficiency induced the upregulation of genes associated with neutrophil chemotaxis and migration and the acute inflammatory response, which was confirmed with GSEA (**Figures 4E, F**; **Figure S4A**; **Table S8**). KEGG analysis and GSEA revealed gene enrichment in NAFLD, type I diabetes, and NET formation (**Figure 4F**; **Figure S4B**). NETs that consist of long chromatin filaments occur in the early stages of NAFLD and dysregulation of NETs can lead to chronic inflammation and exacerbate the progression of NAFLD (32). Hence, we examined key genes involved in neutrophil migration, response to interferon-gamma, and NET formation (**Figure 4G**) and found that the function-related genes were higher in KO group especially in NET formation pathway. Based on the results of pseudotime analysis, cell proportion data, and GO enrichment analysis, *ApoA4* knockout mainly affected the proportion and function of 0-Gran-Wfdc17. These results suggested that the increase in the proportion of mature neutrophils and the formation of NETs contribute to the aggravation of NAFL.

### Validation of results from the scRNA-seq data by qRT–PCR and immune staining

**Figure 5:**
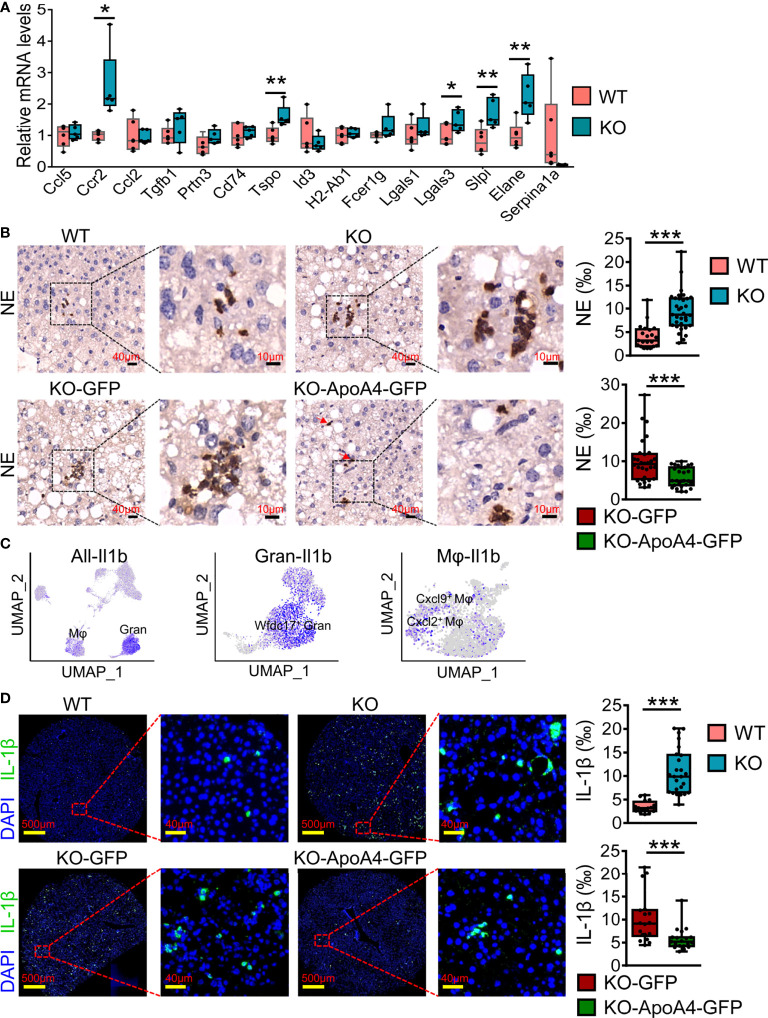
ScRNA-seq data were further validated by qRT–PCR and immune staining in enlarged samples. **(A)** Liver qRT–PCR analysis for representative DEGs found in scRNA-seq analysis in mice fed a HFD for 16 weeks (n = 5-6). The results are expressed as the mean ± SEM. Boxes include the values from the first to third quartiles, whereas horizontal bars represent the medians. Data were analyzed using two-way ANOVA. *P < 0.05, **P < 0.01, vs. WT. **(B)** NE (neutrophil elastase) immunostaining experiments in liver paraffin sections for HFD sections; zoomed in areas with quantification of positive cells from multiple views in liver sections. **(C)** Feature plot of the inflammatory gene *Il1b* in all immune cell (left), granulocyte (Gran, middle) and macrophage (Mφ, right) subsets. **(D)** IL-1β immunostaining experiments in murine liver paraffin sections with DAPI nuclear counterstain and the quantification of positive cells from multiple views in liver sections. Data are presented as the mean ± standard error of the mean. The statistical significance of differences was assessed with Student’s t test, ***P < 0.001. KO-GFP: KO-GFP group, which were *ApoA4* knockout NAFL mice subjected to AAV-*GFP* adenoviral infection as a control; KO-ApoA4-GFP: KO-ApoA4-GFP group, which were *ApoA4* knockout NAFL mice with *ApoA4* stable overexpression induced by AAV-*ApoA4*-*GFP* adenoviral infection.

To further validate the expression of genes of interest that were DEGs of liver immune cells between the two groups found in scRNA-seq analysis, we examined the expression of these genes in liver immune cells by real-time PCR after enlarging the size of the samples. The qRT–PCR results were basically consistent with the scRNA-seq analysis (**Figure 5A**), especially *Tspo*, *Lgals3*, *Slpi* and *Serpina1a*. In particular, Ccr2, which could promote obesity-associated macrophage infiltration in hepatic tissue to induce inflammation and angiogenesis (33), showed no obvious expression differences between WT and KO mice by scRNA-seq analysis, but its expression changed significantly after enlarging the samples (using qRT–PCR). Neutrophil infiltration was characterized by immunostaining NAFL liver biopsy sections with neutrophil elastase (NE, encoded by *Elane*), which has been used as a neutrophil marker (34). Consistent with the results of scRNA-seq, there were more neutrophils in KO samples (**Figure 5B**). Interestingly, the neutrophils were clustered together in lobules, and their localization had no obvious correlation with the portal area. To further confirm the effect of ApoA4 on neutrophil infiltration in NAFL, we injected KO mice with adenoviruses containing *ApoA4* genes and then fed them a HFD for 16 weeks. In line with our expectations, the stable expression of *ApoA4* decreased neutrophil infiltration (**Figure 5B**). NAFLD is characterized by increases in both lipid accumulation and the expression of proinflammatory cytokines such as IL-1β (35). In this single-cell study, we found that *Il1b* (encoding IL-1β) was mainly expressed by 4-Mφ-Cxcl2 and 0-Gran-Wfdc17 (**Figure 5C**), although *Il1b* was not differentially expressed in whole hepatic immune cells (**Figure 1F**). IL-1β positive cells were abundantly detected in KO murine liver paraffin sections, and the stable expression of ApoA4 decreased IL-1β positive cells in NAFL liver tissue (**Figure 5D**). We infer that ApoA4 may reduce the fat accumulation caused by hepatic metabolic inflammation by inhibiting the expression of IL-1β in inflammatory macrophages (4-Mφ-Cxcl2) and activated neutrophils (0-Gran-Wfdc17).

### Consistent gene profiles between *ApoA4*-deficient NAFL mice and human NAFLD

**Figure 6:**
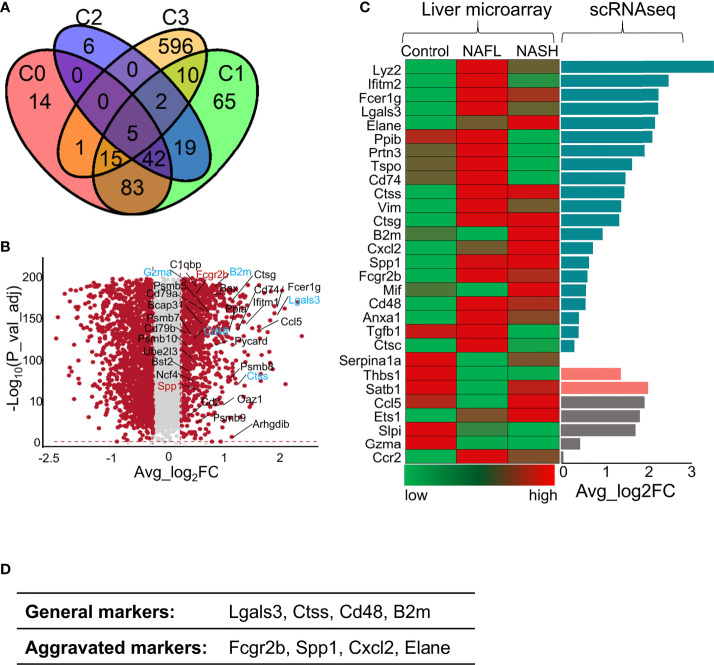
Comparison of gene profile alterations during human NAFLD pathogenesis with mouse NAFL regulated by *ApoA4* deficiency. **(A)** Venn diagram showing the overlap of upregulated genes identified in the KO group with genes identified in a cohort study by Michael et al. (2020) (36) (*P* = 3.7e-11), as determined by a hypergeometric test. C0 was a set of differentially expressed genes between healthy controls and patients with mild liver steatosis, while C1 included genes upregulated in advanced liver steatosis compared with healthy controls. C2 consists of differentially expressed genes between patients with mild and advanced liver steatosis. C3 was upregulated in the KO group in this study. **(B)** Volcano plot showing differentially expressed genes in the KO group vs. WT group. Red dots indicate genes passing our *P value*-adjust and fold difference thresholds. Thirty-two genes that were crossed between C1 and C3 are marked with gene names, genes crossed between C2 and C3 are colored blue, and genes crossed among C1, C2 and C3 are colored red. **(C)** Comparison of the expression of genes of interest between NAFLD datasets (GSE63067) (left) obtained by liver microarrays and the data from this study obtained by scRNA-seq (right); Average_log_2_ fold change > 0 in dark green, Average_log_2_ fold change (KO vs. WT) < 0 in rose- Bengal. The gene expression patterns in patients from control to NAFL and NASH were inconsistent with our DEGs identified by scRNA-seq in grey. **(D)** The proposed universal NAFLD markers both in humans and in mice.

To detect whether liver immune disruptions after *ApoA4* knockout in HFD-induced NAFL occurred during human NAFLD pathogenesis, we analyzed two microarray datasets (GSE151158 and GSE63067) from human liver tissue and the DEGs in whole hepatic immune cells from this study (**Figure 6**; **Table S9**). Compared with dataset (GSE151158) shown in the Venn plot (**Figure 6A**), we found that 33 genes intersected between C3 (DEGs in this study) and human DEGs. Fifteen genes intersected among C0, C1, and C3; 10 genes intersected between C1 and C3, which are shown in **Figure 6B**. Notably, 5 genes (*Lgals3*, *Ctss*, *Cd48*, *B2m*, and *Gzma*) intersected among C0, C1, C2, and C3, and 2 genes (*Fcgr2b* and *Spp1*) intersected among C1, C2 and C3, which are considered aggravation markers (**Figure 6B**). The results of a comparison with GSE63067 human data also showed many NAFLD markers (**Figure 6C**), most of which are consistent with the markers shown in **Figure 6B**. Based on the above analysis of human and mouse transcriptome data, it can be concluded that (**Figure 6D**) the expression of *Lgals3*, *Ctss*, *Cd48*, and *B2m* as universal NAFLD markers, gradually increased with the aggravation of NAFLD (**Figures 6A, B, D**). The expression of *Fcgr2b* and *Spp1* (**Figure 6B**) was increased only in aggravated fatty liver, and the expression levels of *Cxcl2* and *Elane* (**Figures 6C, D**) increased when liver fibrosis occurred which is not completely reversible, suggesting that these genes may play a key role in the prognosis of NAFLD.

**Figure 7:**
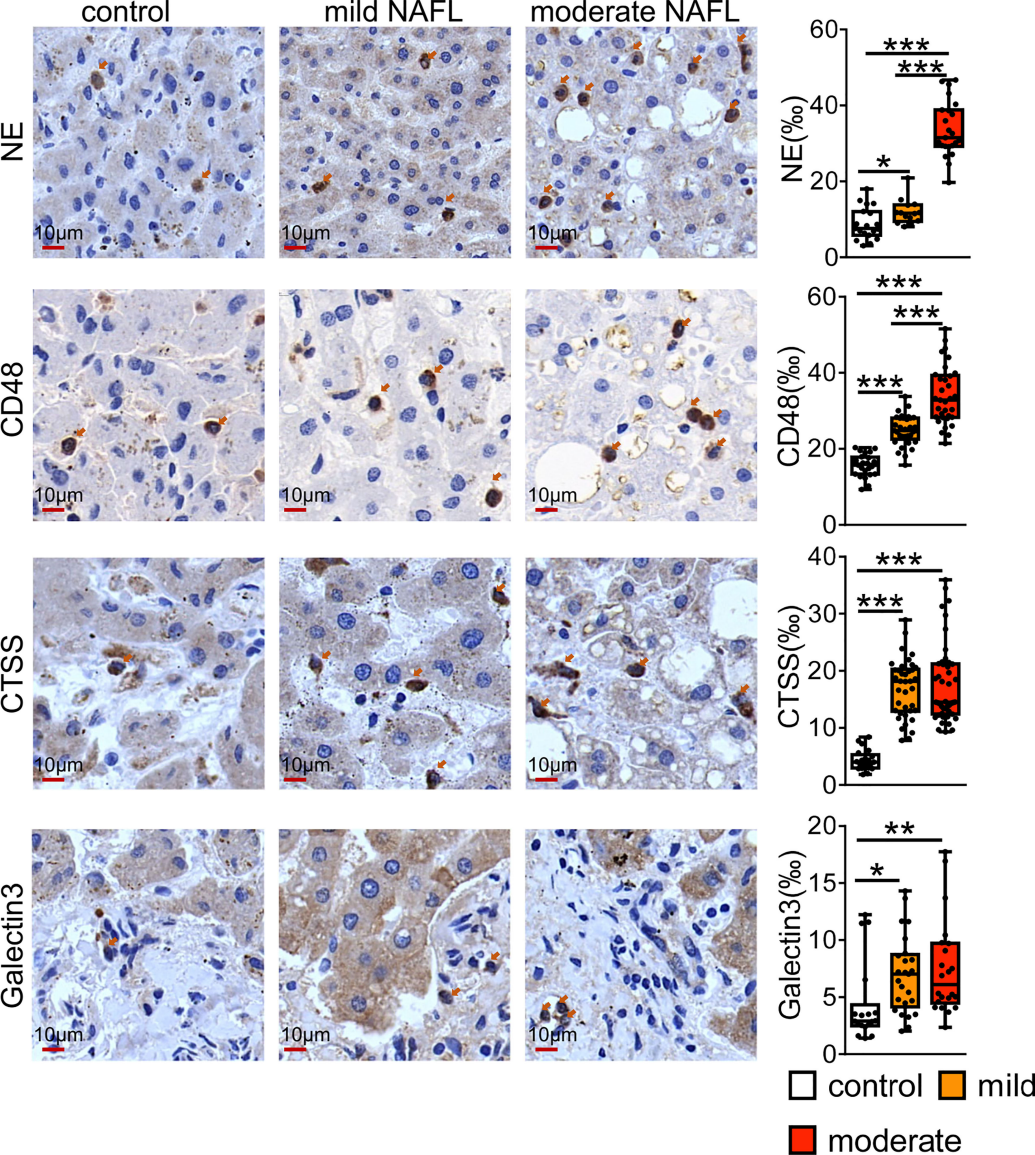
Immunostaining for CD48, NE, CTSS and Galtectin3 in human NAFL liver. Bars = 10 μm (×100). *P < 0.05, **P < 0.01, ***P <0.001, vs. control.

We checked the value of universal NAFLD markers by staining human sections with some of universal markers. As shown in the **Figure 7**, CD48 and NE positive immune cells were mainly distributed in the liver lobules. Compared with normal controls, CD48 and NE positive cells were significantly higher in patients with mild and moderate fatty liver, and highest in patients with moderate NAFL. CTSS and Galtectin3 positive immune cells were significantly higher in mild and moderate fatty liver compared with normal controls, and CTSS and Galtectin3-positive cells were mainly distributed in the portal area.

### Impact of ApoA4 on cell crosstalk among hepatic immune cells by CellChat analysis

**Figure 8:**
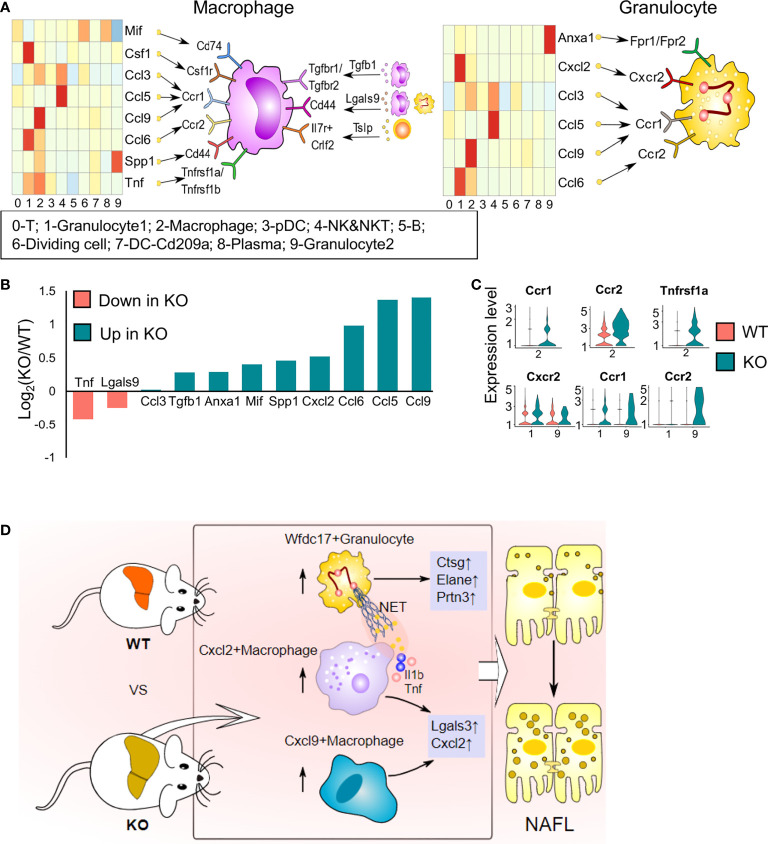
Cell–cell interaction plots according to receptor–ligand analysis using CellChat and the effects of ApoA4 on the immune microenvironment in NAFL livers. **(A)** Signal interaction between macrophages and other cell types (left) and signal interactions between granulocytes and other cell types (right). **(B)** Average expression values of ligands shown in **(A)** in WT and KO NAFL livers based on the scRNA-seq dataset. **(C)** Violin plots showing values of receptors in **(A)** in WT and KO NAFL livers based on the scRNA-seq dataset. **(D)** Profiling of the effects of *ApoA4* deletion on the immune microenvironment in NAFL and its potential mechanisms, as described in the discussion. Wfdc17^+^ granulocyte: 0-Gran-Wfdc17; Cxcl9^+^ macrophage: 4-Mφ-Cxcl9; Cxcl2^+^ macrophage: 4-Mφ-Cxcl2; NET: neutrophil extracellular trap.

It is gradually being realized that interactions among immune cells are vital for the development of NAFL. ScRNA-seq enables us to explore potential interactions by mapping ligands and receptors to specific clusters (18). We used CellChat to reveal the paracrine or autocrine system in specific cell types and display ligand–receptor crosstalk between macrophages or granulocytes and other cells (**Figure 8A**). Macrophages express various membrane receptors (Ccr1/2, Tgfbr1/2, Tnfrsf1a/Tnfrsf1b, Csf1r) and secrete many factors (Tnf, Spp1, Lgals9, Mif, Csf1, chemokine ligands and Tgfb1) to influence themselves and crosstalk with other immune cells. Ccr2- and Ccr1-mediates accumulation of myeloid cells in the liver (33) (**Figure 8A**). Tgfb1, a pleiotropic cytokine, regulates the growth and differentiation of various cell types and is associated with insulin resistance, obesity, and hepatic steatosis (37). Csf1 controls the proliferation and differentiation of the mononuclear phagocyte system through Csf1r and leads to the recruitment of monocytes and neutrophils (38). Tnf promotes inflammatory activity and affects the survival of macrophages by stimulating Tnfr1 and Tnfr2 expression (39). Mif, Spp1, and Lgals9 are anti-inflammatory signals (40). Granulocytes exhibit abundant expression of Anxa1, which exerts protective effects through paracrine or autocrine signalling, and Anxa1 is upregulated with NAFLD progression (41). Granulocytes also expressed Ccr1, Ccr2, and Cxcr2 in response to chemokine ligands and Cxcl2 expressed by other cells (42) (**Figure 8A**). Cxcr2 is mainly expressed on neutrophils and is involved in neutrophil chemotaxis (42). T cells also have extensive connections with other cells mainly through Il2, Ccl5, and Lgals9 (**Figure 8A**). We analysed the expression of ligands and receptors detected by CellChat in total immune cells (scRNA-seq data) and found that most ligands and some receptors increased after *ApoA4* knockout (**Figures 8B, C**; **S5**). This finding indicates that *ApoA4* deletion strengthens the interaction among immune cells by upregulating ligands, which leads to the aggravation of NAFL.

## Discussion

The liver is the largest digestive organ and plays a crucial role in nutrient and energy metabolism. In addition to the role of the liver in metabolic functions, it is also a vital immune organ that hosts lymphocytes and nonlymphoid cells (Kupffer cells, DCs and neutrophils). Metabolic disturbance linked to hepatic immune system imbalance (innate and adaptive immunity) can contribute to a diverse array of liver diseases, such as NAFLD (2). Although both innate and adaptive immunity are involved in the development and progression of NAFLD, innate immune mechanisms play a leading role in promoting hepatic inflammation in NAFLD (43).

Several studies have indicated that macrophages are vital in the pathogenesis of NAFLD because chemical deletion of liver macrophages can alleviate and even reverse insulin resistance and liver steatosis as well as inflammation in diet-induced steatosis (26). During the progression of NAFLD, tissue-resident macrophages (MTRs) exhibit a different transcriptome, with higher expression of *Trem2* and *Cd9*, and the apoptosis in MTRs is accelerated, while the loss of MTRs is compensated by infiltrating MDMs that exhibit characteristics similar to those of MTRs (26). In NAFLD, MTRs have homeostatic, metabolic, and tolerogenic functions, playing an adaptive and protective role. However, MDMs infiltrate the liver in a *Ccr2*-dependent manner and promote inflammation (33, 44). In this study, the macrophage subsets 0-Mφ-Thbs1, 3-Mφ-Gngt2 and 4-Mφ-Cxcl2 were MDMs that make up the bulk of macrophages in NAFLD, while 2-Mφ-Cxcl9 was an MTRs population. Our transcriptomics data showed that the computationally determined macrophage clusters were not entirely consistent with traditional M1 or M2 markers, and there was overlap in the expression of markers of these subsets (20). Previous studies have shown that macrophages display high degrees of plasticity, and M1 or M2 polarized macrophages represent only extreme states, as macrophages can exhibit characteristics of the M1 and M2 states or states that are distinct from both M1 and M2 (45). In that context, using a single marker to identify macrophage subtypes is irrational, and the heterogeneity of macrophages in NAFLD cannot be readily captured by the simplified nomenclature previously defined *in vitro*.

Increased expression of proinflammatory cytokines such as IL-1β is a main feature of NAFLD (35). IL-1β is mainly expressed by inflammatory macrophages (20, 35). NETs, released by neutrophils, can induce IL-1β production by macrophages (46). As an immune mediator, IL-1β might not only promote fat accumulation in the liver and induce hepatocyte steatosis but also activate stellate cells and amplify inflammation-induced *Ccl2*, *Tnf*, and *Il1b* expression (35). In our study, we found that *Il1b* was prominently expressed by 4-Mφ-Cxcl2 and 0-Gran-Wfdc17 and was strongly upregulated by *ApoA4* deficiency. These results confirm that IL-1β plays a vital role in the aggravation of NAFLD.

Neutrophils play an important role in regulating the inflammatory response (47). Biopsies of the human liver show that excessive neutrophil infiltration correlates with the progression of NAFL to NASH (47). Neutrophil depletion can alleviate liver injuries in NAFLD mice (3). Neutrophils in liver tissue can produce various cytokines and chemokines (such as IL-1β, IL-6, and chemokine ligands) to induce monocytes, lymphocytes, and other neutrophils to surround steatotic hepatocytes, which can accelerate inflammation and promote NAFLD development (3). In addition, neutrophils can release diversified granule proteins, such as neutrophil serine proteases (including NE, PRTN3 and Cathepsin G) and Lipocalin 2 (48), to promote the development of obesity, NAFLD and type 2 diabetes mellitus through insulin resistance and metabolic inflammation (49, 50). Therefore, increased abundance of 0-Gran-Wfdc17, elevated levels of neutrophil serine proteases (NE, PRTN3 and Cathepsin G), and upregulated genes enriched in NETs formation are involved in the aggravation of fatty liver caused by loss of *ApoA4*.

ApoA4 can regulate some subset of liver immune cells and their function, but the underlying molecular and signal transduction mechanisms need to be discussed. It was reported that ApoA4 can inhibit hepatic gluconeogenesis by not only interacting with the nuclear receptors NR4A1 and NR1D1 but also stimulating their expression (14, 51). NR4A1 has anti-inflammatory properties and controls the differentiation and survival of ‘patrolling’ Ly6C monocytes, which are essential for neutrophil recruitment (52). Moreover, *Nr4a1* deletion in B cells exacerbates atherosclerosis in association, with an enhanced T follicular helper response (53). Moreover, it was considered that low−density lipoprotein receptor−related protein 1(LRP1) is a novel receptor of ApoA4 in adipose tissue (54). In our scRNA-seq data, *Nr4a1* was significantly downregulated in KO mice in whole hepatic immune cells (**Figures 1F**; **3B**; **4E**; **S6**), but *Lrp1* was upregulated mainly in macrophages and granulocytes of *ApoA4* deficiency mice (**Figure S6**). These results suggest that ApoA4 may directly suppress inflammation *via* ApoA4-NR4A1 pathways or by more complex mechanism. Therefore, future studies are warranted to investigate possible receptors for ApoA4 in liver immune cells.

In conclusion, we found the following changes in hepatic immune cell proportions and their functions resulting from ApoA4 deficiency in diet-induced mouse NAFL (**Figure 8D**): 1) Extraordinarily, ApoA4 mediates liver-specific subsets, namely, Cxcl9^+^ macrophage, Cxcl2^+^ macrophage, and Wfdc17^+^ granulocyte to inhibit NAFL. 2) We explored many novel NAFLD-associated marker genes in these hepatic immune cells, including lower Thbs1 and Serpina1a expression; higher Lgals3, Ctss, Cd48, and B2m as NAFLD general markers and Fcgr2b, Spp1, Cxcl2 and Elane as aggravation markers in both humans and mice. 3) ApoA4 deletion leads to the activation of many functional pathways, not only in whole immune cells but also in their subsets, including NAFLD, neutrophil chemotaxis, and myeloid leukocyte migration. In addition, the novel NAFLD-associated diagnostic module established in this study could be a diagnosis and treatment standard and a therapeutic target for obesity-associated NAFLD.

This study also has some limitations. First, the insufficient investigation limits our interpretation of how adaptive immune cells (T and B cells) regulated by ApoA4 in NAFL. Second, the cellular crosstalk between immune cells also needs to be further explored. Third, it remains to be determined whether the near disappearance of some kind of granulocytes after *ApoA4* deficiency are pathogenic factors that are linked to NAFL progression. Fourth, scRNA-seq of liver immune cells is a powerful tool for visualization of the cellular landscape, but it remains to be interpreted carefully owing to technical limitations and heterogeneity of algorithms for data analyses in different researches.

## Data availability statement

The data presented in the study are deposited in the NCBI GEO repository, accession number GSE212546.

## Ethics statement

The studies involving human participants were reviewed and approved by College of Forensic Medicine. The patients/participants provided their written informed consent to participate in this study. The animal study was reviewed and approved by Xi’an Jiaotong university health science center.

## Author contributions

XL is the first corresponding author, and SL and ZL are joint corresponding authors. XL conceived the project. XL, SL, ZL, X-HL designed and supervised research. X-HL was responsible for the experiments, data collection and data analysis with support from JZ, C-XY, QB, CC, and J-NF. X-HL performed bioinformatic analyses. J-TZ, QB and J-NF supervised bioinformatic analyses. XL and X-HL finished the manuscript. SL, ZL, C-XY, QZ, JX and QB provided support in writing, review and editing the manuscript. All authors contributed to the article and approved the submitted version.

## Funding

This work was supported by grants from the National Natural Science Foundation of China (No. 81770798) and Natural Science Foundation of Shaanxi Province (No.2020JM-405).

## Acknowledgments

We are grateful to Wanbao Yang PhD and professor Shaodong Guo for their help in writing, review and editing the manuscript. We thank professor Patrick Tso (Mouse Metabolic Phenotyping Center, Department of Pathology and Laboratory Medicine, Metabolic Diseases Institute, University of Cincinnati, USA) for the kind donation of *ApoA4* knock-out mice.

## Conflict of interest

The authors declare that the research was conducted in the absence of any commercial or financial relationships that could be construed as a potential conflict of interest.

## Publisher’s note

All claims expressed in this article are solely those of the authors and do not necessarily represent those of their affiliated organizations, or those of the publisher, the editors and the reviewers. Any product that may be evaluated in this article, or claim that may be made by its manufacturer, is not guaranteed or endorsed by the publisher.
