# Supplementary material for: Single-cell RNA sequencing reveals a novel inhibitory effect of ApoA4 on NAFL mediated by liver-specific subsets of myeloid cells
Source: Front Immunol. 2022 Nov 8;13:1038401. doi: 10.3389/fimmu.2022.1038401 (PMC9678944; doi:10.3389/fimmu.2022.1038401)
Supplement: Supplementary file 1 [file DataSheet_1.pdf]

SUPPLEMENTAL MATERIAL

Summary

Supplementary Figures 1 to 6

Supplementary Table 1: The sequences of primers used for qRT–PCR

Supplementary Table 2: The marker genes specifically expressed by each cell type identified in the study

Supplementary Table 3: Cell clusters in 16 weeks HFD NAFLD liver

Supplementary Table 4: DEGs between WT and KO mice

Supplementary Table 5: Subcluster markers of macrophages and DCs

Supplementary Table 6: DEGs in macrophages and DCs between WT and KO mice

Supplementary Table 7: Subcluster markers of granulocytes

Supplementary Table 8: DEGs in granulocytes between WT and KO mice

Supplementary Table 9: Basic information on the human liver microarray data

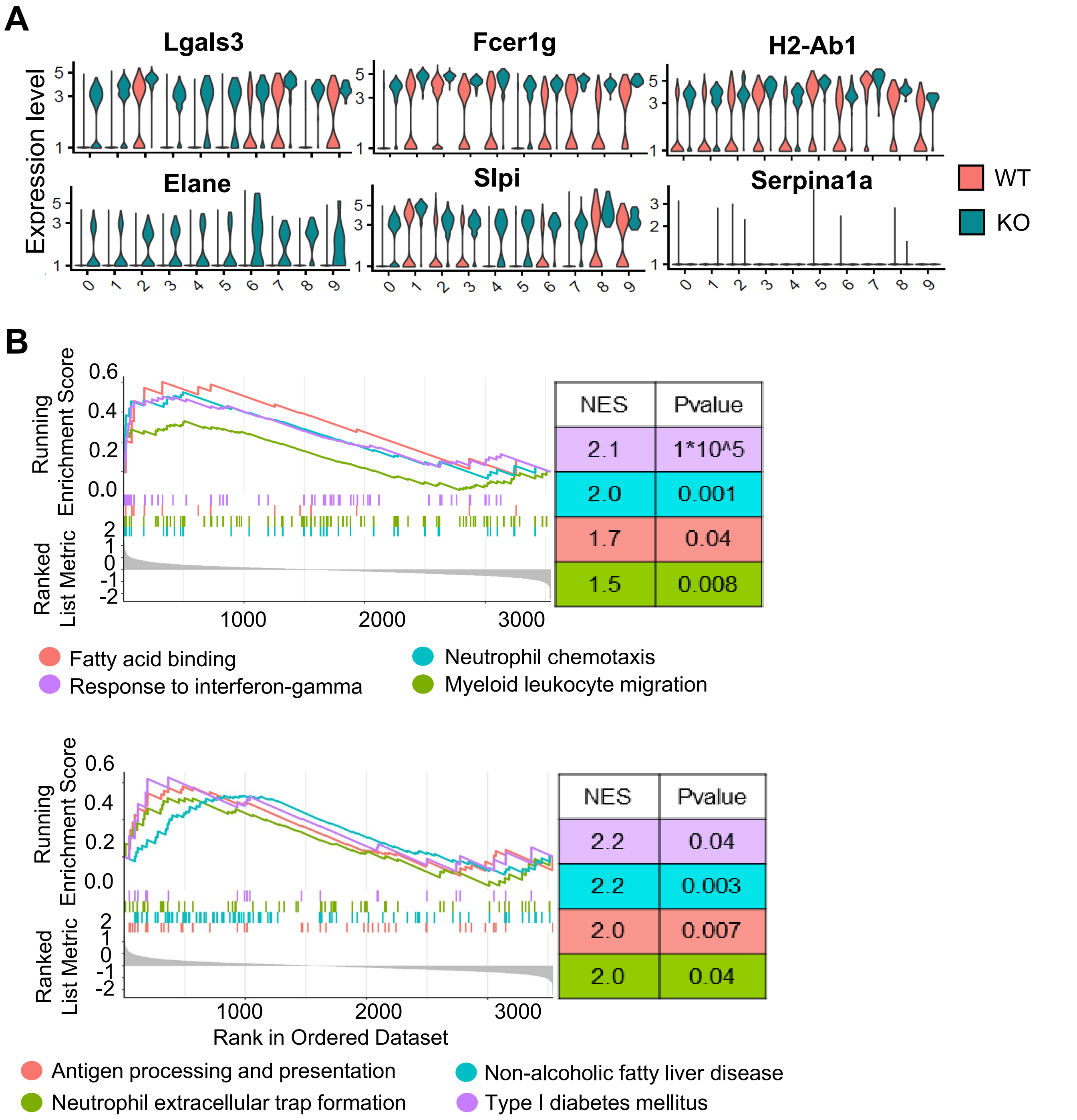

Supplementary FIGURE 1 | Analysis of the differences between the WT and KO groups. **(A)** Violin plots showing the representative differentially expressed genes in **Figure 1F** across nine clusters. **(B)** Gene set enrichment analysis (GSEA) plot of 8 MSigDB hallmark gene sets. NES, normalized enrichment score. The gene list contained all differentially expressed genes in KO mice vs. WT mice (*P* <0.05).

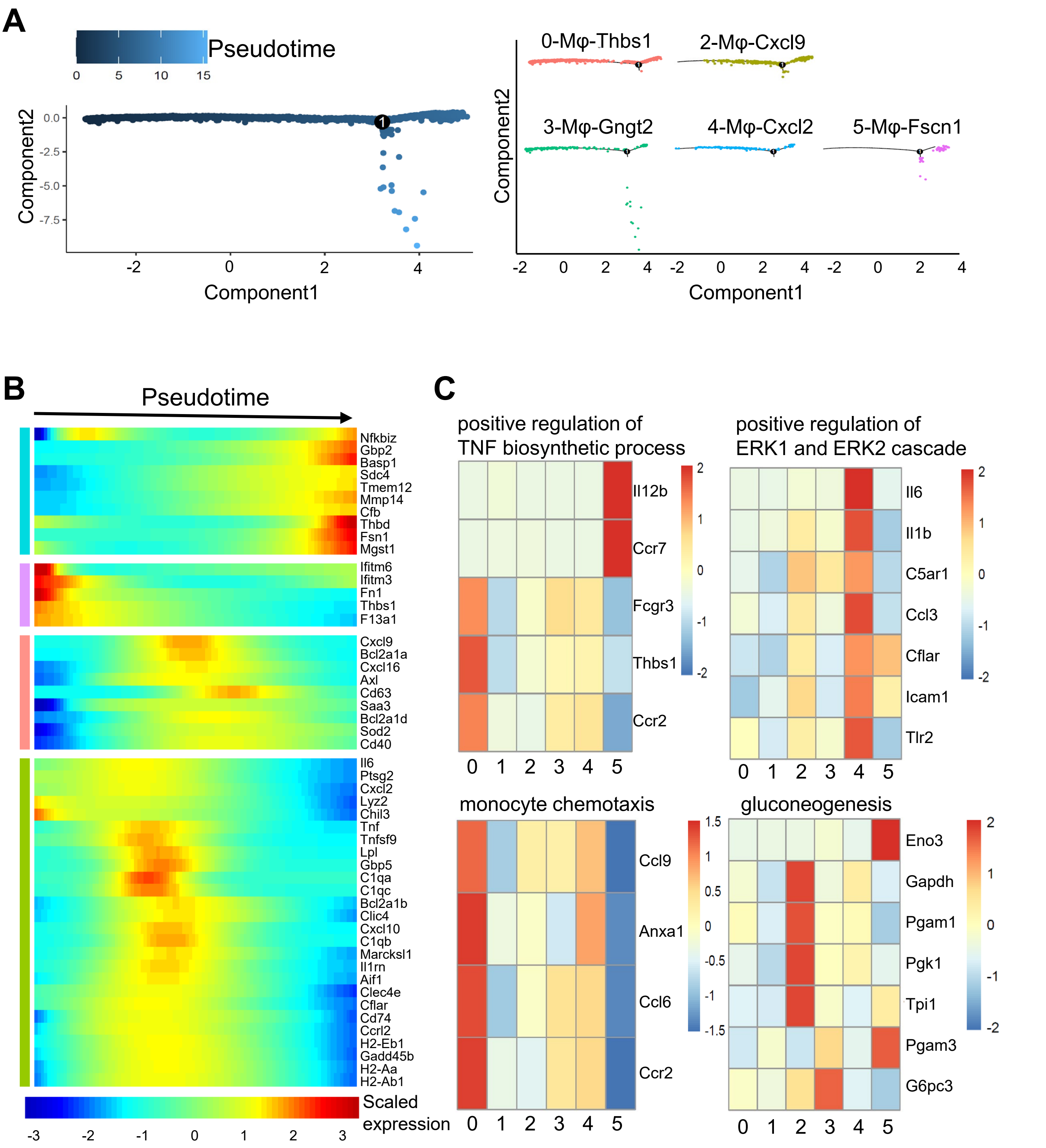

Supplementary FIGURE 2 | Pseudotime analysis shows dynamic changes in macrophages in NAFLD mice. **(A)** The ordering of macrophages along pseudotime defined by Monocle2. **(B)** Heatmap of the expression levels of the top 50 DEGs (*P*<0.01) in the analysed macrophages is shown. **(C)** GO BP term-related gene (in **FIgure 2D**) expression in macrophage and DCs subsets.

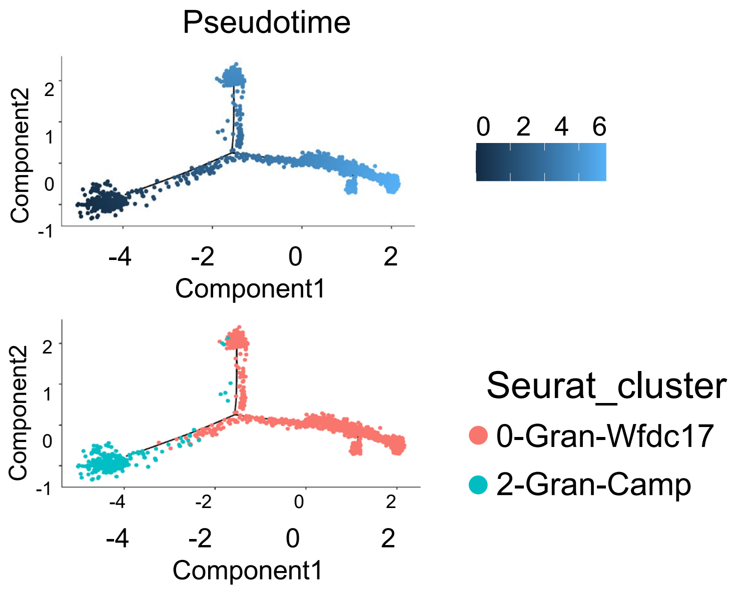

Supplementary FIGURE 3 | Illustration of neutrophil differentiation pathways inferred by Monocle2.

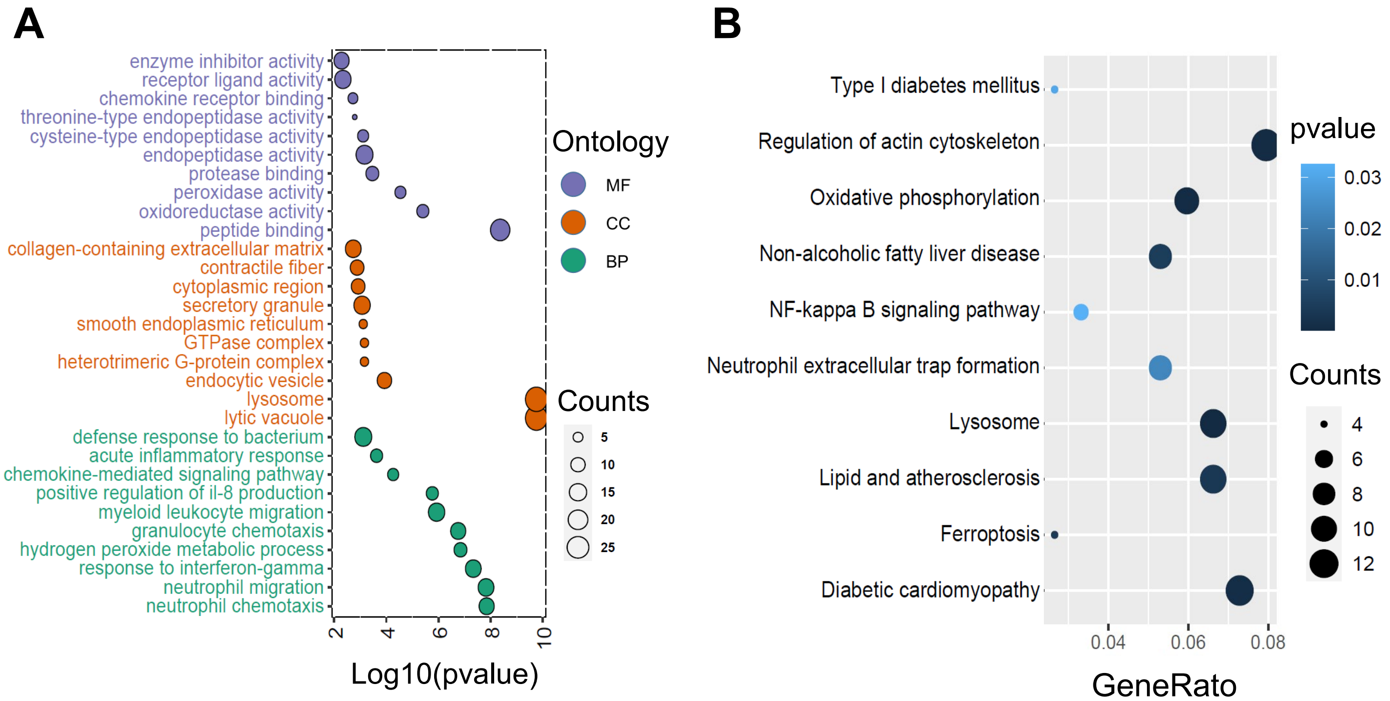

Supplementary FIGURE 4 | Analysis of the differences in granulocytes between WT and KO mice. **(A)** GO enrichment analysis of biological processes for the upregulated genes of granulocytes in the KO group. **(B)** KEGG enrichment analysis of upregulated genes of granulocytes in the KO group.

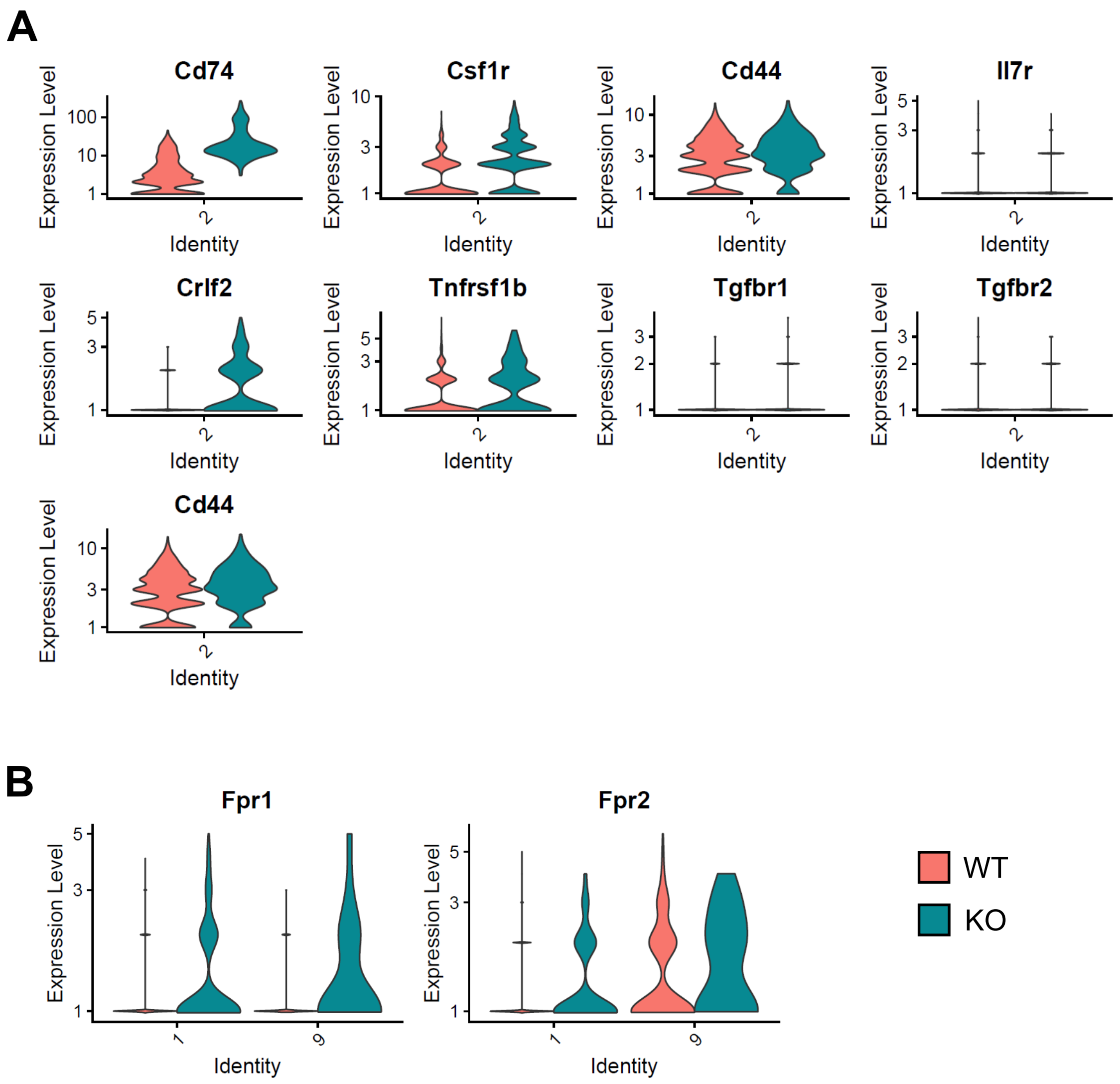

Supplementary FIGURE 5 | Violin plots showing the values for receptors on *ApoA4*-associated myeloid cells in WT and KO NAFL livers based on the scRNA-seq dataset. **(A)** Specific receptors expressed by 2-macrophages. **(B)** Specific receptors expressed by 1,9-granulocytes.

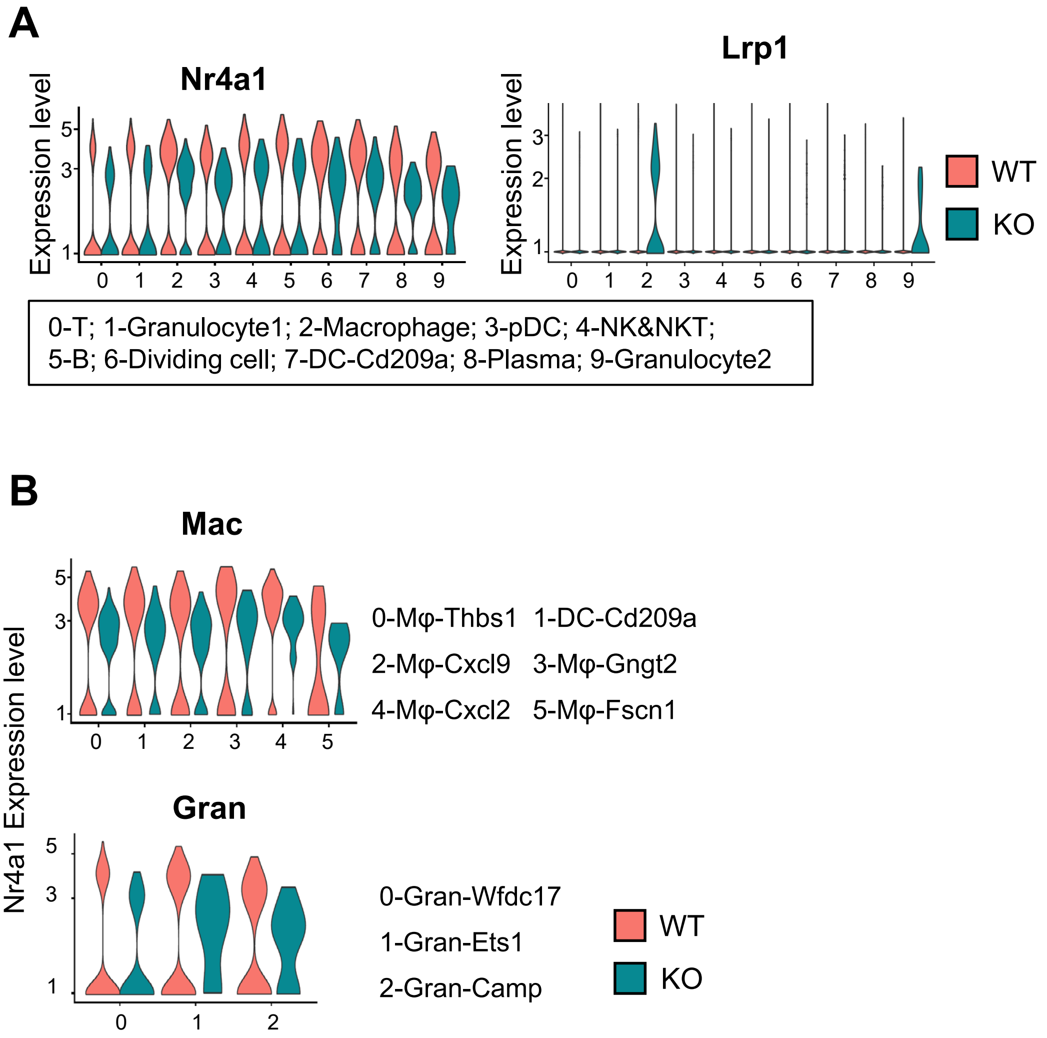

Supplementary FIGURE 6 | Volin plots showing the expression of *Nr4a1* and *Lrp1* in the subsets of whole hepatic immune cells from WT and KO mice. (A) The expression of *Nr4a1* (left) and *Lrp1* (right) in the subsets of whole hepatic immune cells. (B) The expression of *Nr4a1* in the subsets of macrophages (Mac, up) and granulocyte (Gran, down).

Supplementary Table 1 | The sequences of primers used for qRT–PCR.

Primer	Sense	antisense
Lgals3	5’ GGAGAGGGAATGATGTTGCCT 3’	5’ TCCTGCTTCGTGTTACACACA 3’
Lgals1	5’ CAA GCT GCC AGA CGG ACA T 3’	5’ AGGCCACGCACTTAATCTTGA 3’
Fcer1g	5’ ATCTCAGCCGTGATCTTGTTCT 3’	5’ ACCATACAAAAACAGGACAGCAT 3’
H2-Ab1	5’ TGAACAGCCCAATGTCGTCAT 3’	5’ CAGCGCACTTTGATCTTGGC 3’
Serpina1a	5’ TAGGGAGCAAGGGTGACACTC 3’	5’ ACTGTCTGGTCTGTTGAGGGT 3’
Slpi	5’ GGCCTTTTACCTTTCACGGTG 3’	5’ TACGGCATTGTGGCTTCTCAA 3’
Ccl5	5’ GCTGCTTTGCCTACCTCTCC 3’	5’ TCGAGTGACAAACACGACTGC 3’
Cd74	5’ CCGCCTAGACAAGCTGACC 3’	5’ ACAGGTTTGGCAGATTTCGGA 3’
Prtn3	5’ ATGGCTGGAAGCTACCCATC 3’	5’ TGCCCACCTACAATCTTGGAG 3’
Tspo	5’ GCCTACTTTGTACGTGGCGAG 3’	5’ CCTCCCAGCTCTTTCCAGAC 3’
Id3	5’ GACGACATGAACCACTGCTAC 3’	5’ CCTGGCTAAGCTGAGTGCC 3’
Elane	5’ CCCATCACAACTGCTGAACGA 3’	5’ AGACATGGAGTTCTGTCACCC 3’
Ccr2	5’ GCCATCATAAAGGAGCCATACC 3’	5’ TGTGGTGAATCCAATGCCCT 3’
Ccl2	5’ TCAGCCAGATGCAGTTAACGC 3’	5’ TCTGGACCCATTCCTTCTTGG 3’
Tgfb1	5’ TGATACGCCTGAGTGGCTGTCT 3’	5’CACAAGAGCAGTGAGCGCTGAA 3’

Supplementary Table 2 | The marker genes specifically expressed by each cell type identified in the study.

Category	Subcategory	WT (%)	A4KO（%）	Cluster identity genes
**macrophages& DCs**		17.3	23.5	
	0-Mφ-Thbs1	9.0	11.6	Adgre1, Lyz2, Mafb, Thbs1, F13a1, Lyz2, Ly6c2, Ccr2, Fn1, Chil3
	1-DC-Cd209a	2.8	3.2	Cd74, Cd209a, H2-Ab1, H2-Eb1, H2-Aa, Cst3, Ckb, Tcf4, Cd83, Gm2a
	2-Mφ-Cxcl9	2.2	4.1	Cxcl9, Lpl, Cxcl10, Il1rn, Gbp2, Isg15, Mmp14, Sdc4, Apoe, Cstb, Aif1, Rgs1
	3-Mφ-Gngt2	1.8	2.0	Ear2, Ace, Gngt2, Itgal, Adgre4, Hes1, Tgm2, C3
	4-Mφ-Cxcl2	0.9	2.2	Saa3, Cxcl2, Tnfsf9, Il6, Nfkbia, Tnf, Nlrp3, Il1b, Ccl3, Chil3
	5-Mφ-Fscn1	0.4	0.4	Ccr7, Fscn1, Tmem123, Tbc1d4, Mabp5, Ccl22, Il12b, Fabp5, Mreg
**granulocytes**		20.1	15.6	
	0-Gran-Wfdc17	11.1	14.1	Wfdc17, Ifitm1, Ccl6, Il1b, Ifitm2, Ifitm3, Cxcl2, Slpi, Ftl1
	1-Gran-Ets1	7.1	0.3	Gzma, Hspa1b, Satb1, Ets1, Tpt1, Wdr89, Foxp1, Il12rb, Hsp90aa1, Irf8, Cebpa
	2-Gran-Camp	1.8	1.2	Camp, Ngp, Ltf, Chil3, Cd177, Ifitm6, Lyz2, Cybb, Lcn2, Anxa1, Serpinb1a
**T and NK&NKT cells**		36.2	34.2	Cd3d, Il7r, Gzma, Klre1
**pDCs**		12.3	10.6	Ccr9, Siglech, Cox6a2, Bst2, Tcf4, Ly6d, Lair1, Runx2, Spib, Grn, Ctsl
**B cells**		7.4	8.9	Cd79a, Scd1 Ms4a1, Cd74, Cd79b, Cd83, H2Eb1, Ccr7, Satb1, Dusp2
**dividing cells**		4.2	4.5	Hist1h2ao, Top2a, Stmn1, Mki67, Hmgb2
**Plasma cells**		2.3	2.7	Jchain, Mzb1, Hsp90b1, Pdia4, Txndc5, Slpi, Xbp1

Supplementary Table 3

gene	p_val	avg_log2FC	pct.1	pct.2	p_val_adj	cluster
Cd3d	4.47E-282	1.620045685	0.3	0.092	5.98E-278	0-T
Il7r	7.55E-223	1.538063816	0.352	0.152	1.01E-218	0-T
Tcf7	8.76E-199	1.484138022	0.258	0.09	1.17E-194	0-T
Ms4a4b	6.01E-178	1.283834851	0.301	0.127	8.04E-174	0-T
Rps19	0	1.268179971	0.924	0.768	0	0-T
Rps24	0	1.238746658	0.874	0.705	0	0-T
Rplp1	0	1.207408555	0.993	0.932	0	0-T
Rps18	0	1.152127091	0.812	0.639	0	0-T
Gramd3	2.48E-118	1.115871571	0.328	0.183	3.32E-114	0-T
Rps13	0	1.105080062	0.733	0.567	0	0-T
Rps23	0	1.096532903	0.947	0.824	0	0-T
Thy1	9.42E-108	1.093533994	0.283	0.15	1.26E-103	0-T
Satb1	4.31E-207	1.082241718	0.578	0.377	5.77E-203	0-T
Rpl23a	0	1.072914178	0.935	0.795	0	0-T
Rps14	0	1.045676068	0.818	0.667	0	0-T
Rps15	0	1.041453123	0.901	0.765	0	0-T
LOC10086243:	0	1.038750151	0.903	0.769	0	0-T
Ccr7	1.40E-117	1.037101314	0.353	0.203	1.87E-113	0-T
Rpl17	0	1.036949407	0.847	0.718	0	0-T
Wdr89	0	1.022115093	0.997	0.961	0	0-T
Rpsa	0	1.015958403	0.793	0.643	0	0-T
Rps16	0	1.002097635	0.941	0.834	0	0-T
Gimap3	1.25E-92	0.99982044	0.308	0.181	1.68E-88	0-T
Eef1b2	2.46E-138	0.995413775	0.519	0.394	3.30E-134	0-T
Rpl32	0	0.989175922	0.894	0.757	0	0-T
Rnf125	3.80E-83	0.982983096	0.294	0.176	5.08E-79	0-T
Rps7	1.39E-65	0.981316518	0.265	0.168	1.86E-61	0-T
Rps20	0	0.968263243	0.921	0.825	0	0-T
Rps27rt	0	0.967478916	1	0.998	0	0-T
Emb	8.12E-65	0.964288824	0.334	0.238	1.09E-60	0-T
Rps5	1.45E-87	0.963856396	0.448	0.357	1.94E-83	0-T
Rps10	7.47E-100	0.960509486	0.431	0.329	1.00E-95	0-T
Rpl39	0	0.953060623	0.906	0.763	0	0-T
Rplp0	3.28E-236	0.95018319	0.756	0.641	4.39E-232	0-T
Ets1	2.26E-210	0.945692062	0.665	0.481	3.03E-206	0-T
Rpl13	0	0.941350244	0.945	0.849	0	0-T
Rpl22l1	3.22E-246	0.934450402	0.725	0.584	4.31E-242	0-T
Tpt1	0	0.932028275	0.977	0.894	0	0-T
Rps3	0	0.929011046	0.829	0.714	0	0-T
Rps8	0	0.924767525	0.797	0.654	0	0-T
Rpl18	6.43E-262	0.92206659	0.733	0.606	8.61E-258	0-T
Rpl5	6.88E-63	0.917797598	0.346	0.261	9.22E-59	0-T
Peli1	6.71E-74	0.915263579	0.391	0.287	8.98E-70	0-T
LOC10524420{	1.04E-189	0.91420643	0.721	0.613	1.39E-185	0-T
Rpl35	0	0.907438786	0.959	0.857	0	0-T
Saraf	5.79E-68	0.905622787	0.352	0.253	7.75E-64	0-T
Gm10045	1.75E-245	0.898901506	0.718	0.596	2.35E-241	0-T
Vps37b	9.69E-190	0.88390383	0.674	0.53	1.30E-185	0-T
Rps2	1.04E-84	0.876594898	0.46	0.374	1.39E-80	0-T
Rpl21	1.05E-167	0.875478804	0.624	0.519	1.41E-163	0-T
Rps4x	0	0.863635573	0.883	0.771	0	0-T
Gm25380	3.26E-168	0.831498995	0.674	0.584	4.37E-164	0-T
Hcst	1.49E-66	0.830565368	0.26	0.155	2.00E-62	0-T
Zc3hav1	4.42E-48	0.821898514	0.302	0.219	5.92E-44	0-T
Rps3a1	1.13E-301	0.819278021	0.844	0.736	1.52E-297	0-T

Rpl27a	2.80E-286	0.81748408	0.809	0.704	3.74E-282	0-T
Tmsb10	0	0.806153323	0.982	0.904	0	0-T
Rpl8	4.68E-292	0.799888182	0.839	0.753	6.26E-288	0-T
Rpl14	1.62E-185	0.790987852	0.749	0.655	2.17E-181	0-T
Rpl22	3.19E-243	0.788972029	0.793	0.673	4.27E-239	0-T
Rpl34-ps1	0	0.783611871	0.917	0.823	0	0-T
Rps6	3.61E-28	0.778051058	0.279	0.234	4.83E-24	0-T
Rpl37a	3.64E-87	0.77748518	0.536	0.448	4.87E-83	0-T
Uba52	4.73E-303	0.767089753	0.91	0.832	6.33E-299	0-T
Rpl19	2.65E-199	0.760943999	0.77	0.681	3.55E-195	0-T
Rpl15	3.13E-175	0.750607944	0.732	0.647	4.20E-171	0-T
Rpl6	3.57E-145	0.744280043	0.675	0.586	4.78E-141	0-T
Rpl10a	1.19E-161	0.740360703	0.701	0.623	1.59E-157	0-T
Rpl4	1.75E-184	0.730857038	0.733	0.645	2.34E-180	0-T
Eef1g	8.47E-59	0.729220365	0.441	0.37	1.13E-54	0-T
Rpl37rt	0	0.723686058	0.985	0.959	0	0-T
Rpl35a	2.97E-48	0.708946162	0.43	0.375	3.97E-44	0-T
Npm1	3.21E-45	0.706787314	0.404	0.345	4.30E-41	0-T
LOC10816792	0	0.696776285	0.934	0.857	0	0-T
Snrpg	1.94E-38	0.695449261	0.357	0.294	2.60E-34	0-T
Ccnd2	7.85E-42	0.690020433	0.256	0.174	1.05E-37	0-T
Gm4705	2.31E-28	0.688919693	0.317	0.269	3.09E-24	0-T
LOC10263818	3.33E-36	0.683820308	0.389	0.333	4.45E-32	0-T
Atp1b3	1.35E-49	0.681264033	0.472	0.406	1.81E-45	0-T
Rdm1	5.18E-23	0.671556105	0.312	0.266	6.93E-19	0-T
Rpl29	1.10E-78	0.664802628	0.55	0.499	1.48E-74	0-T
Rps11	7.10E-125	0.661508416	0.707	0.634	9.50E-121	0-T
Eef1a1	5.23E-282	0.653459911	0.912	0.834	7.00E-278	0-T
Elf1	2.11E-27	0.651432608	0.301	0.242	2.82E-23	0-T
Rpl26	7.19E-144	0.649583066	0.736	0.663	9.63E-140	0-T
Rps25	9.96E-133	0.631142389	0.746	0.667	1.33E-128	0-T
Rps26	7.05E-25	0.627852176	0.345	0.304	9.44E-21	0-T
P2ry10	3.43E-28	0.626205798	0.328	0.271	4.59E-24	0-T
Ifngr1	6.98E-32	0.602809461	0.405	0.351	9.35E-28	0-T
Rpl7	7.73E-100	0.598633741	0.655	0.6	1.03E-95	0-T
Rpl41	0	0.591536285	1	0.995	0	0-T
Ptpn18	1.39E-27	0.584960502	0.413	0.38	1.86E-23	0-T
Rps27a	6.06E-245	0.555402413	0.948	0.907	8.11E-241	0-T
Ncl	7.21E-20	0.531160546	0.378	0.346	9.64E-16	0-T
Cox7a2l	2.14E-20	0.525237499	0.386	0.361	2.87E-16	0-T
Tnfaip3	1.58E-51	0.519359339	0.587	0.522	2.12E-47	0-T
Jak1	2.25E-19	0.508285587	0.361	0.325	3.01E-15	0-T
**gene**	**p_val**	**avg_log2FC**	**pct.1**	**pct.2**	**p_val_adj**	**cluster**
S100a8	0	3.375136507	0.996	0.917	0	1-Granulocytes1
Retnlg	0	3.335387468	0.955	0.628	0	1-Granulocytes1
Wfdc21	0	3.304505424	0.799	0.223	0	1-Granulocytes1
Wfdc17	0	3.300787163	0.932	0.641	0	1-Granulocytes1
S100a9	0	3.107917457	1	0.96	0	1-Granulocytes1
G0s2	0	2.827732132	0.781	0.336	0	1-Granulocytes1
Ifitm1	0	2.763660451	0.686	0.319	0	1-Granulocytes1
Csf3r	0	2.661654603	0.717	0.209	0	1-Granulocytes1
Acod1	0	2.65626208	0.509	0.121	0	1-Granulocytes1
Cxcl2	0	2.517325078	0.821	0.444	0	1-Granulocytes1
Cxcr2	0	2.485068359	0.413	0.067	0	1-Granulocytes1
Il1b	0	2.468684817	0.958	0.599	0	1-Granulocytes1
Hdc	0	2.403955286	0.727	0.258	0	1-Granulocytes1
Mmp8	0	2.352849241	0.407	0.108	0	1-Granulocytes1
Mmp9	0	2.27355298	0.442	0.095	0	1-Granulocytes1

Il1f9	0	2.237295637	0.325	0.059	0	1-Granulocytes1
S100a11	0	2.223779137	0.853	0.473	0	1-Granulocytes1
Lcn2	0	2.203267458	0.546	0.154	0	1-Granulocytes1
Lrg1	0	2.192195347	0.396	0.117	0	1-Granulocytes1
Grina	0	2.098706612	0.666	0.282	0	1-Granulocytes1
Tnfaip2	0	2.080036898	0.445	0.145	0	1-Granulocytes1
Pglyrp1	0	2.067047398	0.476	0.165	0	1-Granulocytes1
Il1r2	0	1.995226891	0.35	0.094	0	1-Granulocytes1
Mxd1	0	1.92544307	0.669	0.306	0	1-Granulocytes1
Ccl6	0	1.910997358	0.594	0.308	0	1-Granulocytes1
C5ar1	1.66E-291	1.896326365	0.296	0.075	2.22E-287	1-Granulocytes1
Clec4d	1.65E-284	1.881624144	0.381	0.134	2.21E-280	1-Granulocytes1
Clec4e	4.79E-295	1.877446271	0.467	0.199	6.42E-291	1-Granulocytes1
Marcks	0	1.871428168	0.531	0.227	0	1-Granulocytes1
Fbxl5	1.03E-277	1.848838481	0.374	0.13	1.38E-273	1-Granulocytes1
Gadd45a	0	1.836036715	0.485	0.207	0	1-Granulocytes1
Trem1	1.24E-265	1.823925584	0.312	0.092	1.66E-261	1-Granulocytes1
Ccr1	2.08E-255	1.807944049	0.255	0.062	2.79E-251	1-Granulocytes1
Cd14	6.84E-240	1.794212869	0.469	0.234	9.16E-236	1-Granulocytes1
Slc16a3	6.72E-269	1.785200861	0.386	0.143	8.99E-265	1-Granulocytes1
Slfn4	2.30E-230	1.777493936	0.29	0.088	3.09E-226	1-Granulocytes1
Ptgs2	1.42E-212	1.772543585	0.34	0.128	1.89E-208	1-Granulocytes1
S100a6	0	1.753722851	0.89	0.643	0	1-Granulocytes1
Msrb1	0	1.740743087	0.685	0.381	0	1-Granulocytes1
Gda	0	1.733440739	0.529	0.226	0	1-Granulocytes1
Mcemp1	3.59E-242	1.730165361	0.339	0.119	4.81E-238	1-Granulocytes1
Lilr4b	2.91E-259	1.720521664	0.371	0.133	3.90E-255	1-Granulocytes1
Hp	0	1.678462967	0.627	0.334	0	1-Granulocytes1
Pla2g7	4.32E-211	1.670139894	0.357	0.145	5.78E-207	1-Granulocytes1
Cd300lf	5.08E-218	1.657079674	0.352	0.137	6.80E-214	1-Granulocytes1
Lmnb1	2.03E-268	1.609537787	0.519	0.262	2.72E-264	1-Granulocytes1
Cebpb	0	1.600841269	0.933	0.639	0	1-Granulocytes1
Nfkbiz	2.72E-259	1.598303627	0.582	0.334	3.64E-255	1-Granulocytes1
Slpi	0	1.591628437	0.575	0.295	0	1-Granulocytes1
Spi1	2.73E-217	1.58968061	0.436	0.215	3.65E-213	1-Granulocytes1
Cd9	2.14E-189	1.588911541	0.31	0.117	2.86E-185	1-Granulocytes1
Gsr	0	1.564196897	0.614	0.319	0	1-Granulocytes1
Ets2	4.21E-220	1.533500719	0.438	0.205	5.63E-216	1-Granulocytes1
Ifitm2	4.97E-245	1.454276351	0.614	0.389	6.65E-241	1-Granulocytes1
Ccrl2	3.72E-90	1.44500747	0.29	0.16	4.98E-86	1-Granulocytes1
Alox5ap	2.24E-220	1.438050545	0.557	0.352	2.99E-216	1-Granulocytes1
Anxa1	1.80E-151	1.410049895	0.317	0.14	2.41E-147	1-Granulocytes1
Mcl1	0	1.409055245	0.802	0.555	0	1-Granulocytes1
Lilrb4a	4.08E-129	1.381480199	0.325	0.162	5.46E-125	1-Granulocytes1
Adipor1	2.58E-112	1.372831103	0.293	0.146	3.45E-108	1-Granulocytes1
Marcksl1	6.68E-70	1.370968561	0.27	0.158	8.94E-66	1-Granulocytes1
Tpd52	4.74E-200	1.338027481	0.53	0.315	6.35E-196	1-Granulocytes1
Sorl1	7.03E-114	1.29031909	0.339	0.184	9.42E-110	1-Granulocytes1
Txn1	1.48E-177	1.287042666	0.539	0.36	1.99E-173	1-Granulocytes1
Zyx	1.46E-121	1.285059797	0.411	0.252	1.96E-117	1-Granulocytes1
Rnf149	2.08E-207	1.276140831	0.568	0.339	2.78E-203	1-Granulocytes1
Srgn	0	1.267608661	0.872	0.677	0	1-Granulocytes1
Clec7a	6.31E-82	1.252410312	0.25	0.129	8.45E-78	1-Granulocytes1
Ier3	1.50E-77	1.213998367	0.303	0.18	2.00E-73	1-Granulocytes1
Lcp1	8.76E-164	1.210455885	0.552	0.379	1.17E-159	1-Granulocytes1
Nfkbia	1.66E-210	1.202513738	0.743	0.622	2.22E-206	1-Granulocytes1
Osm	1.08E-69	1.157295516	0.288	0.172	1.44E-65	1-Granulocytes1
Prr13	1.37E-124	1.154080737	0.474	0.321	1.84E-120	1-Granulocytes1

Gcnt2	1.32E-92	1.140927554	0.352	0.212	1.77E-88	1-Granulocytes1
Atp6v1g1	2.43E-115	1.137494452	0.468	0.329	3.25E-111	1-Granulocytes1
Gpcpd1	4.36E-80	1.089319633	0.373	0.245	5.83E-76	1-Granulocytes1
Taldo1	8.77E-66	1.083553214	0.337	0.232	1.17E-61	1-Granulocytes1
R3hdm4	1.97E-63	1.066081663	0.281	0.172	2.64E-59	1-Granulocytes1
Picalm	1.17E-83	1.064692539	0.407	0.28	1.57E-79	1-Granulocytes1
Anxa2	1.72E-88	1.018287953	0.419	0.288	2.30E-84	1-Granulocytes1
Dusp1	2.99E-249	1.016593543	0.82	0.642	4.00E-245	1-Granulocytes1
Pirb	9.52E-47	1.009311818	0.252	0.162	1.27E-42	1-Granulocytes1
Ptafr	1.23E-39	1.007410914	0.251	0.17	1.65E-35	1-Granulocytes1
Mrpl33	5.89E-110	1.003163163	0.521	0.39	7.88E-106	1-Granulocytes1
2810474O19Ril	9.44E-57	0.987760315	0.254	0.149	1.26E-52	1-Granulocytes1
Tspo	4.73E-96	0.974329857	0.515	0.404	6.33E-92	1-Granulocytes1
Litaf	6.27E-57	0.937000201	0.357	0.255	8.39E-53	1-Granulocytes1
Samsn1	4.38E-60	0.90597434	0.37	0.261	5.87E-56	1-Granulocytes1
Emilin2	6.10E-41	0.897789329	0.281	0.195	8.16E-37	1-Granulocytes1
Trib1	7.37E-30	0.884424795	0.274	0.207	9.87E-26	1-Granulocytes1
Rhog	1.88E-32	0.872976809	0.267	0.195	2.51E-28	1-Granulocytes1
Gadd45b	9.83E-29	0.859250347	0.331	0.27	1.32E-24	1-Granulocytes1
Vasp	2.99E-47	0.858006362	0.357	0.266	4.00E-43	1-Granulocytes1
Csrnp1	1.05E-48	0.84727037	0.371	0.277	1.41E-44	1-Granulocytes1
Trim30a	4.35E-36	0.83567212	0.321	0.245	5.82E-32	1-Granulocytes1
Map1lc3b	1.94E-35	0.829318676	0.369	0.31	2.60E-31	1-Granulocytes1
Gmfg	2.19E-38	0.810740383	0.326	0.247	2.94E-34	1-Granulocytes1
St3gal4	9.33E-23	0.801852824	0.287	0.228	1.25E-18	1-Granulocytes1
Pde4b	6.86E-33	0.795460866	0.295	0.219	9.18E-29	1-Granulocytes1
Cd44	5.61E-121	0.793876374	0.674	0.527	7.51E-117	1-Granulocytes1
Plek	6.44E-27	0.789394988	0.335	0.278	8.62E-23	1-Granulocytes1
Tyrobp	5.17E-191	0.786571509	0.829	0.649	6.92E-187	1-Granulocytes1
Fxyd5	2.10E-79	0.786234847	0.541	0.442	2.82E-75	1-Granulocytes1
Lst1	4.51E-36	0.78621967	0.332	0.25	6.04E-32	1-Granulocytes1
Dgat1	1.06E-40	0.779664716	0.329	0.24	1.42E-36	1-Granulocytes1
Junb	2.96E-263	0.763097109	0.956	0.884	3.96E-259	1-Granulocytes1
Zfp36	4.48E-109	0.762530789	0.67	0.544	5.99E-105	1-Granulocytes1
Sdcbp	1.40E-36	0.757368249	0.415	0.353	1.87E-32	1-Granulocytes1
Lbr	5.28E-28	0.747958956	0.343	0.282	7.06E-24	1-Granulocytes1
Coq10b	6.81E-23	0.740801343	0.259	0.199	9.12E-19	1-Granulocytes1
Tgfbi	5.64E-35	0.73036815	0.291	0.209	7.55E-31	1-Granulocytes1
Pkm	3.26E-26	0.718474029	0.348	0.295	4.37E-22	1-Granulocytes1
Gapdh	2.32E-37	0.710993177	0.448	0.391	3.10E-33	1-Granulocytes1
Txnip	1.94E-16	0.707291722	0.289	0.248	2.60E-12	1-Granulocytes1
Gabarap	2.82E-27	0.671879712	0.394	0.349	3.77E-23	1-Granulocytes1
Nfe2l2	1.94E-13	0.655805022	0.284	0.251	2.59E-09	1-Granulocytes1
Ifitm3	3.59E-101	0.649401878	0.599	0.427	4.80E-97	1-Granulocytes1
Iqgap1	2.45E-23	0.647603582	0.381	0.337	3.28E-19	1-Granulocytes1
Kctd12	5.07E-14	0.644939316	0.301	0.265	6.78E-10	1-Granulocytes1
Sell	1.88E-14	0.633090991	0.264	0.223	2.52E-10	1-Granulocytes1
Ddx6	4.39E-31	0.63134537	0.427	0.366	5.88E-27	1-Granulocytes1
Mt-mt-Rnr2	6.08E-130	0.623193406	0.872	0.83	8.14E-126	1-Granulocytes1
Shfm1	2.42E-85	0.595103498	0.736	0.659	3.23E-81	1-Granulocytes1
Fth1	6.03E-125	0.586474801	0.941	0.892	8.07E-121	1-Granulocytes1
Selplg	1.24E-30	0.584549005	0.461	0.409	1.66E-26	1-Granulocytes1
Slfn2	9.23E-16	0.575403263	0.34	0.305	1.24E-11	1-Granulocytes1
Rab8b	8.91E-12	0.568652202	0.266	0.231	1.19E-07	1-Granulocytes1
Cdk2ap2	3.32E-07	0.558124998	0.296	0.287	0.0044474	1-Granulocytes1
Fos	9.21E-100	0.546849505	0.817	0.736	1.23E-95	1-Granulocytes1
Klf2	3.07E-80	0.541170735	0.737	0.671	4.11E-76	1-Granulocytes1
Cd52	8.79E-55	0.536065716	0.653	0.597	1.18E-50	1-Granulocytes1

Ptp4a1	8.54E-09	0.533156213	0.298	0.28	0.0001144	1-Granulocytes1
Cox17	6.03E-13	0.531695058	0.427	0.422	8.08E-09	1-Granulocytes1
Ier5	1.51E-25	0.527579609	0.533	0.511	2.02E-21	1-Granulocytes1
Kdm7a	2.00E-09	0.513313622	0.291	0.264	2.67E-05	1-Granulocytes1
Sat1	5.41E-32	0.511448858	0.465	0.401	7.24E-28	1-Granulocytes1
Plaur	4.92E-08	0.501090063	0.292	0.272	0.0006591	1-Granulocytes1
Dazap2	6.54E-13	0.500258155	0.417	0.409	8.76E-09	1-Granulocytes1
**gene**	**p_val**	**avg_log2FC**	**pct.1**	**pct.2**	**p_val_adj**	**cluster**
Ccr9	0	3.464534886	0.727	0.043	0	3-pDC
Siglech	0	3.321753865	0.752	0.052	0	3-pDC
Cox6a2	0	3.174488362	0.657	0.058	0	3-pDC
Bst2	0	3.150779014	0.945	0.214	0	3-pDC
Tcf4	0	3.03211957	0.891	0.143	0	3-pDC
Ly6d	0	2.733733305	0.822	0.223	0	3-pDC
Lair1	0	2.520413866	0.608	0.072	0	3-pDC
Runx2	0	2.299627343	0.651	0.114	0	3-pDC
Spib	0	2.298169828	0.481	0.053	0	3-pDC
Grn	0	2.264432765	0.851	0.238	0	3-pDC
Ctsl	0	2.193592154	0.518	0.083	0	3-pDC
Rnase6	0	2.187326704	0.507	0.082	0	3-pDC
Irf8	0	2.162612156	0.884	0.307	0	3-pDC
Bcl11a	0	2.145158897	0.492	0.064	0	3-pDC
Rell1	0	2.124591838	0.561	0.084	0	3-pDC
Ccnd1	0	2.052055629	0.37	0.028	0	3-pDC
Klk1	0	2.043618701	0.314	0.023	0	3-pDC
Zc3h12c	0	2.034409329	0.349	0.027	0	3-pDC
Cd300c	0	2.014467279	0.368	0.022	0	3-pDC
Ccl4	0	1.954265364	0.796	0.46	0	3-pDC
Atp1b1	0	1.88640536	0.484	0.082	0	3-pDC
Cyb561a3	0	1.886378918	0.428	0.058	0	3-pDC
Pltp	0	1.882186383	0.358	0.035	0	3-pDC
Mpeg1	0	1.819709112	0.698	0.209	0	3-pDC
Sdc4	0	1.81398765	0.429	0.064	0	3-pDC
Smim5	0	1.793642359	0.345	0.04	0	3-pDC
Ctsb	0	1.76290137	0.824	0.313	0	3-pDC
Pld4	0	1.755943664	0.635	0.185	0	3-pDC
Ptprs	0	1.736830774	0.39	0.053	0	3-pDC
Dnajc7	0	1.728795418	0.57	0.155	0	3-pDC
Irf2bp2	0	1.715945485	0.84	0.326	0	3-pDC
Sh3bgr	0	1.667061321	0.278	0.025	0	3-pDC
Tifa	0	1.627924053	0.398	0.082	0	3-pDC
Mctp2	0	1.564415309	0.333	0.046	0	3-pDC
Abhd17b	0	1.550349276	0.439	0.099	0	3-pDC
Ncf1	0	1.520031579	0.672	0.247	0	3-pDC
Fyn	0	1.498750273	0.547	0.146	0	3-pDC
Lgals1	0	1.489601408	0.722	0.303	0	3-pDC
Mef2c	0	1.482536828	0.614	0.17	0	3-pDC
Tspan13	0	1.479084183	0.662	0.234	0	3-pDC
Tsc22d1	0	1.473540431	0.361	0.076	0	3-pDC
Psap	0	1.457291859	0.998	0.726	0	3-pDC
Tex2	0	1.444701609	0.289	0.043	0	3-pDC
Snx5	0	1.434927289	0.6	0.204	0	3-pDC
Rpl31-ps12	0	1.396909806	0.969	0.668	0	3-pDC
Lag3	0	1.383300174	0.253	0.033	0	3-pDC
St8sia4	5.53E-305	1.364847338	0.552	0.184	7.41E-301	3-pDC
Syngr2	5.55E-276	1.347883412	0.459	0.145	7.43E-272	3-pDC
Ly6a	6.43E-181	1.328245944	0.344	0.116	8.61E-177	3-pDC
Ctsh	3.55E-239	1.321073671	0.451	0.16	4.75E-235	3-pDC

Fam46c	7.46E-207	1.306050348	0.331	0.095	9.98E-203	3-pDC
Rilpl2	3.29E-268	1.298617942	0.461	0.147	4.40E-264	3-pDC
Blnk	3.08E-283	1.236620725	0.266	0.046	4.12E-279	3-pDC
Cd47	2.39E-249	1.224760795	0.556	0.221	3.20E-245	3-pDC
Dclre1c	1.62E-233	1.219796468	0.31	0.075	2.17E-229	3-pDC
Xbp1	8.23E-248	1.208749684	0.545	0.208	1.10E-243	3-pDC
Fam174a	2.55E-217	1.19365313	0.402	0.132	3.42E-213	3-pDC
Cybb	4.82E-290	1.189498762	0.673	0.274	6.45E-286	3-pDC
Herpud1	8.49E-230	1.181239876	0.58	0.242	1.14E-225	3-pDC
Ifnar2	2.04E-205	1.16760564	0.333	0.098	2.72E-201	3-pDC
Dirc2	4.76E-239	1.16292762	0.287	0.063	6.37E-235	3-pDC
Ptms	1.75E-195	1.150465968	0.337	0.103	2.34E-191	3-pDC
Pgls	4.02E-175	1.129725616	0.323	0.105	5.38E-171	3-pDC
Unc93b1	7.21E-197	1.12959171	0.436	0.163	9.65E-193	3-pDC
Plac8	0	1.117444845	0.946	0.515	0	3-pDC
Ly6c2	1.28E-172	1.117120428	0.608	0.314	1.72E-168	3-pDC
Ly86	1.11E-171	1.116879045	0.295	0.09	1.48E-167	3-pDC
Tyrobp	0	1.100388511	0.964	0.642	0	3-pDC
Glcci1	1.03E-186	1.100071054	0.277	0.072	1.39E-182	3-pDC
Clic4	1.25E-157	1.064065223	0.291	0.091	1.67E-153	3-pDC
Gns	1.30E-184	1.061149055	0.274	0.072	1.74E-180	3-pDC
Clec12a	4.92E-134	1.023829634	0.276	0.095	6.59E-130	3-pDC
Fgfr1op2	1.16E-173	1.017116976	0.387	0.138	1.56E-169	3-pDC
Gltp	5.43E-162	1.011296126	0.254	0.071	7.27E-158	3-pDC
Mvb12a	1.15E-156	1.010308671	0.261	0.076	1.53E-152	3-pDC
Sec61b	1.31E-217	0.97097589	0.78	0.428	1.75E-213	3-pDC
Rnaset2b	2.10E-142	0.967981688	0.364	0.144	2.81E-138	3-pDC
Tmed3	5.09E-124	0.963148873	0.252	0.085	6.82E-120	3-pDC
Gnas	1.38E-146	0.957214377	0.5	0.242	1.84E-142	3-pDC
H2-T23	1.60E-167	0.952321456	0.471	0.195	2.14E-163	3-pDC
Ppia	2.27E-272	0.949868258	0.93	0.636	3.04E-268	3-pDC
Ramp1	3.42E-120	0.947812964	0.252	0.087	4.57E-116	3-pDC
Map3k8	9.85E-120	0.938770914	0.253	0.085	1.32E-115	3-pDC
Serinc3	1.90E-199	0.928909049	0.764	0.423	2.54E-195	3-pDC
Arhgap17	1.01E-127	0.928222164	0.263	0.086	1.35E-123	3-pDC
Stx7	5.21E-125	0.922088236	0.284	0.102	6.98E-121	3-pDC
Cd164	8.31E-174	0.912893269	0.501	0.207	1.11E-169	3-pDC
Tagln2	6.69E-148	0.892343876	0.579	0.296	8.95E-144	3-pDC
Zfp36l1	6.84E-111	0.885051633	0.651	0.386	9.16E-107	3-pDC
Hsp90aa1	1.11E-93	0.884040654	0.699	0.477	1.49E-89	3-pDC
D17H6S56E-5	6.94E-113	0.883562443	0.273	0.099	9.29E-109	3-pDC
Cyth4	1.94E-127	0.882670303	0.322	0.122	2.60E-123	3-pDC
Tagap	4.07E-99	0.880036814	0.3	0.122	5.44E-95	3-pDC
Cd7	2.77E-133	0.879193521	0.327	0.122	3.71E-129	3-pDC
Apobec3	9.17E-136	0.873529829	0.368	0.144	1.23E-131	3-pDC
Gng10	1.94E-109	0.870557453	0.358	0.16	2.59E-105	3-pDC
M6pr	1.74E-112	0.855739402	0.328	0.135	2.33E-108	3-pDC
Prkcd	1.20E-116	0.853183583	0.389	0.171	1.61E-112	3-pDC
Ivns1abp	2.01E-137	0.829939479	0.391	0.157	2.69E-133	3-pDC
Snx18	2.49E-134	0.826795761	0.554	0.275	3.33E-130	3-pDC
Ly6e	4.71E-183	0.82385787	0.852	0.556	6.31E-179	3-pDC
Serp1	1.37E-174	0.821222336	0.756	0.435	1.83E-170	3-pDC
Rabgap1l	3.10E-117	0.819932503	0.262	0.089	4.15E-113	3-pDC
Smim14	3.60E-113	0.816468051	0.348	0.148	4.82E-109	3-pDC
Prcp	8.48E-90	0.806606956	0.276	0.116	1.14E-85	3-pDC
Slc44a2	5.43E-115	0.794958949	0.358	0.148	7.26E-111	3-pDC
Rgs10	2.16E-81	0.792150979	0.274	0.122	2.89E-77	3-pDC
Hspe1	1.63E-119	0.790840134	0.619	0.35	2.18E-115	3-pDC

Smc6	8.01E-104	0.788384216	0.251	0.09	1.07E-99	3-pDC
Mtdh	1.99E-114	0.786900213	0.364	0.155	2.67E-110	3-pDC
Gm4184	1.72E-130	0.773492977	0.67	0.387	2.31E-126	3-pDC
Tram1	5.14E-95	0.7658138	0.302	0.129	6.87E-91	3-pDC
Plaur	2.43E-104	0.765527478	0.478	0.248	3.25E-100	3-pDC
Emp3	6.94E-82	0.763347505	0.373	0.193	9.29E-78	3-pDC
Rap1a	7.45E-98	0.763236491	0.416	0.206	9.97E-94	3-pDC
Smdt1	8.76E-107	0.755482739	0.493	0.26	1.17E-102	3-pDC
Nptn	4.45E-98	0.751989962	0.302	0.125	5.95E-94	3-pDC
Reep5	5.17E-77	0.74047804	0.276	0.126	6.92E-73	3-pDC
Cd69	2.38E-89	0.740363297	0.332	0.15	3.18E-85	3-pDC
Fyb	3.44E-105	0.739125187	0.373	0.166	4.61E-101	3-pDC
Rnf187	6.54E-74	0.734652656	0.267	0.121	8.75E-70	3-pDC
Rrbp1	1.02E-117	0.732469133	0.519	0.259	1.36E-113	3-pDC
Sec11c	1.73E-102	0.719845088	0.38	0.177	2.32E-98	3-pDC
Ubxn4	1.20E-87	0.717711353	0.25	0.099	1.61E-83	3-pDC
Rnaset2a	2.59E-95	0.705407793	0.372	0.177	3.46E-91	3-pDC
Cd74	1.21E-269	0.696854949	0.96	0.688	1.61E-265	3-pDC
Minos1	8.36E-107	0.690441579	0.592	0.336	1.12E-102	3-pDC
Hsp90b1	5.31E-208	0.688975686	0.753	0.393	7.11E-204	3-pDC
Grb2	6.96E-83	0.686041796	0.359	0.174	9.32E-79	3-pDC
Vimp	7.16E-75	0.683741417	0.28	0.128	9.58E-71	3-pDC
H2-Aa	1.66E-167	0.672276317	0.681	0.361	2.22E-163	3-pDC
H13	7.76E-85	0.66540779	0.378	0.188	1.04E-80	3-pDC
Sub1	3.41E-127	0.657078634	0.889	0.679	4.56E-123	3-pDC
Dnajb1	3.16E-49	0.656224542	0.452	0.29	4.23E-45	3-pDC
Kctd12	7.79E-101	0.652827948	0.48	0.243	1.04E-96	3-pDC
Ptpn6	3.39E-80	0.651591174	0.4	0.207	4.53E-76	3-pDC
Lims1	1.68E-63	0.630472195	0.272	0.13	2.25E-59	3-pDC
Hypk	1.98E-81	0.625821157	0.411	0.215	2.65E-77	3-pDC
Ahnak	1.80E-107	0.621172091	0.641	0.366	2.41E-103	3-pDC
Sec61g	1.06E-63	0.617772776	0.337	0.179	1.41E-59	3-pDC
Ptafr	1.95E-72	0.613889766	0.333	0.163	2.61E-68	3-pDC
Pip4k2a	1.68E-64	0.612988283	0.253	0.116	2.24E-60	3-pDC
Uqcr11	7.34E-56	0.604967034	0.283	0.147	9.83E-52	3-pDC
Trim30a	9.92E-80	0.602520904	0.442	0.233	1.33E-75	3-pDC
Rps27l	1.81E-67	0.601291932	0.372	0.2	2.42E-63	3-pDC
Cmah	4.08E-75	0.59904729	0.282	0.123	5.46E-71	3-pDC
Ywhae	4.43E-75	0.598910833	0.407	0.217	5.93E-71	3-pDC
Ctsz	5.50E-61	0.597819727	0.386	0.222	7.37E-57	3-pDC
Arpc5l	1.14E-65	0.594202802	0.257	0.119	1.53E-61	3-pDC
Dpm3	2.95E-80	0.589597968	0.426	0.225	3.95E-76	3-pDC
H2-DMa	1.43E-51	0.588393462	0.277	0.147	1.91E-47	3-pDC
Irf1	2.80E-81	0.586942399	0.398	0.199	3.75E-77	3-pDC
Fth1	6.40E-210	0.58691283	0.993	0.888	8.56E-206	3-pDC
Runx3	5.81E-68	0.583526462	0.376	0.198	7.78E-64	3-pDC
Ubc	1.76E-83	0.58112567	0.774	0.56	2.35E-79	3-pDC
Tmco1	1.00E-48	0.579946774	0.265	0.14	1.34E-44	3-pDC
Mapkapk2	1.43E-56	0.569158038	0.296	0.153	1.92E-52	3-pDC
H2afj	8.71E-60	0.564188147	0.578	0.375	1.17E-55	3-pDC
Slc3a2	1.92E-67	0.563545638	0.452	0.261	2.58E-63	3-pDC
Lsm6	1.84E-57	0.562904224	0.289	0.148	2.46E-53	3-pDC
Sell	2.59E-75	0.562177007	0.4	0.207	3.47E-71	3-pDC
Ptprcap	1.74E-67	0.560517612	0.36	0.189	2.33E-63	3-pDC
Psmb8	3.06E-53	0.553653274	0.404	0.245	4.10E-49	3-pDC
Tmed2	3.75E-58	0.551209073	0.354	0.195	5.01E-54	3-pDC
Psme1	4.71E-53	0.543735989	0.335	0.188	6.30E-49	3-pDC
Romo1	1.03E-55	0.543676647	0.289	0.149	1.38E-51	3-pDC

Rpl10	5.24E-139	0.542544746	0.963	0.774	7.02E-135	3-pDC
Tmem59	5.49E-53	0.538992013	0.267	0.137	7.34E-49	3-pDC
Prkcb	1.91E-79	0.537707987	0.387	0.19	2.55E-75	3-pDC
Spcs2	2.98E-51	0.537135876	0.325	0.183	3.99E-47	3-pDC
Selplg	3.26E-85	0.532402279	0.635	0.388	4.37E-81	3-pDC
Itm2b	2.72E-72	0.525295587	0.683	0.45	3.64E-68	3-pDC
Nrros	1.49E-42	0.516366764	0.262	0.143	2.00E-38	3-pDC
Trib1	9.49E-63	0.51436797	0.37	0.198	1.27E-58	3-pDC
Psme2b	6.18E-54	0.509072343	0.41	0.245	8.28E-50	3-pDC
Trim25	5.14E-50	0.500201199	0.336	0.189	6.88E-46	3-pDC
**gene**	**p_val**	**avg_log2FC**	**pct.1**	**pct.2**	**p_val_adj**	**cluster**
Gzma	0	3.677105195	0.703	0.188	0	4-NK&NKT
Klre1	0	2.429548979	0.405	0.058	0	4-NK&NKT
Ccl5	0	2.427128392	0.963	0.616	0	4-NK&NKT
Gzmb	0	2.382282974	0.366	0.074	0	4-NK&NKT
Xcl1	2.03E-275	2.367792428	0.272	0.045	2.72E-271	4-NK&NKT
Serpinb6b	0	2.366498922	0.431	0.093	0	4-NK&NKT
Nkg7	0	2.360681576	0.65	0.23	0	4-NK&NKT
Spry2	0	2.302525034	0.332	0.05	0	4-NK&NKT
Serpinb9	3.47E-245	2.10134932	0.398	0.113	4.65E-241	4-NK&NKT
Klrc1	2.69E-208	1.84309581	0.333	0.088	3.59E-204	4-NK&NKT
Il2rb	1.93E-258	1.83437827	0.571	0.229	2.59E-254	4-NK&NKT
Rgs1	1.68E-141	1.814562887	0.506	0.268	2.25E-137	4-NK&NKT
Nr4a2	2.30E-79	1.462949546	0.336	0.168	3.07E-75	4-NK&NKT
Pik3r1	1.08E-90	1.444344981	0.308	0.135	1.45E-86	4-NK&NKT
Nabp1	6.07E-83	1.434404834	0.355	0.182	8.12E-79	4-NK&NKT
Id2	2.88E-99	1.399437748	0.422	0.223	3.86E-95	4-NK&NKT
Ccnd2	2.48E-101	1.359684235	0.379	0.178	3.31E-97	4-NK&NKT
Klrd1	8.81E-82	1.351499922	0.289	0.127	1.18E-77	4-NK&NKT
Ugcg	1.65E-70	1.332878538	0.311	0.155	2.21E-66	4-NK&NKT
Bhlhe40	4.41E-83	1.328097669	0.388	0.208	5.91E-79	4-NK&NKT
Ifngr1	6.85E-94	1.174611166	0.535	0.348	9.16E-90	4-NK&NKT
Sh2d2a	2.33E-63	1.160033225	0.272	0.128	3.11E-59	4-NK&NKT
D16Ertd472e	2.55E-41	1.151208268	0.282	0.168	3.41E-37	4-NK&NKT
Zfp36l2	8.04E-116	1.149900281	0.689	0.518	1.08E-111	4-NK&NKT
Irf8	2.30E-71	1.088030822	0.526	0.362	3.07E-67	4-NK&NKT
Crem	4.92E-39	1.023016401	0.389	0.275	6.59E-35	4-NK&NKT
Nr4a3	6.26E-25	0.943061744	0.258	0.172	8.38E-21	4-NK&NKT
Gimap4	1.80E-36	0.942190971	0.28	0.167	2.40E-32	4-NK&NKT
Tnfaip3	2.10E-94	0.937117136	0.691	0.524	2.81E-90	4-NK&NKT
Vps37b	1.78E-93	0.923072994	0.701	0.555	2.38E-89	4-NK&NKT
Ms4a4b	2.56E-31	0.847512329	0.269	0.164	3.42E-27	4-NK&NKT
Dennd4a	1.89E-39	0.837077897	0.51	0.406	2.53E-35	4-NK&NKT
Rdm1	1.73E-18	0.833599608	0.343	0.272	2.32E-14	4-NK&NKT
Jak1	9.67E-29	0.831137419	0.418	0.326	1.29E-24	4-NK&NKT
Ubald2	1.01E-20	0.827979457	0.36	0.292	1.35E-16	4-NK&NKT
Hcst	5.77E-27	0.781108385	0.271	0.174	7.73E-23	4-NK&NKT
Itgal	1.01E-34	0.776027569	0.497	0.412	1.35E-30	4-NK&NKT
Zcchc11	1.66E-20	0.718282766	0.252	0.17	2.22E-16	4-NK&NKT
P2ry10	9.19E-18	0.717807939	0.349	0.28	1.23E-13	4-NK&NKT
Rgs2	1.71E-18	0.695406778	0.452	0.399	2.29E-14	4-NK&NKT
Ets1	8.94E-54	0.672074052	0.643	0.519	1.20E-49	4-NK&NKT
Runx3	5.45E-09	0.653147367	0.258	0.215	7.29E-05	4-NK&NKT
Fosl2	3.27E-21	0.6323614	0.537	0.497	4.38E-17	4-NK&NKT
Gimap3	9.93E-17	0.613813849	0.283	0.208	1.33E-12	4-NK&NKT
Bcl2l11	2.62E-09	0.597421826	0.382	0.356	3.51E-05	4-NK&NKT
H2-K1	3.05E-80	0.580593912	0.88	0.823	4.09E-76	4-NK&NKT
Ccl4	9.13E-37	0.579896752	0.592	0.492	1.22E-32	4-NK&NKT

Ahnak	9.99E-14	0.577695959	0.436	0.396	1.34E-09	4-NK&NKT
Rnf125	2.76E-09	0.575940088	0.251	0.203	3.70E-05	4-NK&NKT
LOC10263566	2.07E-07	0.566081855	0.338	0.321	0.0027719	4-NK&NKT
Myl6	4.09E-10	0.557279541	0.406	0.389	5.47E-06	4-NK&NKT
Dnaja1	0.0004469	0.520341822	0.341	0.34	1	4-NK&NKT
Ptprc	1.82E-25	0.515895463	0.659	0.632	2.44E-21	4-NK&NKT
**gene**	**p_val**	**avg_log2FC**	**pct.1**	**pct.2**	**p_val_adj**	**cluster**
Cd79a	0	2.665370209	0.516	0.103	0	5-B
Scd1	0	2.359928462	0.363	0.059	0	5-B
Ms4a1	0	2.309658018	0.291	0.027	0	5-B
Cd74	0	1.854130961	0.988	0.699	0	5-B
Mef2c	6.07E-191	1.791291725	0.516	0.2	8.13E-187	5-B
H2-Aa	7.35E-305	1.777679157	0.767	0.369	9.84E-301	5-B
Cd79b	2.51E-155	1.751306986	0.251	0.058	3.37E-151	5-B
H2-Eb1	1.81E-243	1.600171231	0.709	0.358	2.42E-239	5-B
Cd83	1.33E-98	1.519708309	0.417	0.201	1.78E-94	5-B
Ccr7	5.46E-151	1.462616072	0.511	0.221	7.31E-147	5-B
Rel	3.05E-84	1.400751131	0.278	0.107	4.08E-80	5-B
H2-Ab1	4.79E-174	1.267674168	0.743	0.475	6.42E-170	5-B
Foxp1	1.10E-74	1.197675754	0.498	0.305	1.48E-70	5-B
Satb1	6.10E-91	1.060887617	0.637	0.414	8.16E-87	5-B
Prkcb	1.42E-41	1.050631354	0.341	0.204	1.90E-37	5-B
Serp1	6.57E-53	0.861079263	0.606	0.463	8.79E-49	5-B
Zfp36l1	3.38E-35	0.824868528	0.527	0.41	4.53E-31	5-B
Fam107b	8.34E-24	0.818190838	0.383	0.288	1.12E-19	5-B
Ppp3ca	5.51E-13	0.75504695	0.264	0.201	7.37E-09	5-B
Map3k1	3.30E-16	0.753221716	0.286	0.209	4.41E-12	5-B
Dusp2	3.58E-13	0.745884053	0.319	0.257	4.80E-09	5-B
Ezr	2.39E-14	0.736912146	0.317	0.246	3.19E-10	5-B
Tcp11l2	3.79E-17	0.733960125	0.311	0.231	5.08E-13	5-B
Macf1	5.26E-13	0.704400211	0.255	0.188	7.04E-09	5-B
Rps19	7.00E-128	0.690321074	0.935	0.799	9.37E-124	5-B
Snx5	7.27E-13	0.690289996	0.31	0.247	9.74E-09	5-B
Ets1	3.27E-49	0.679799245	0.659	0.52	4.38E-45	5-B
Rpl21	3.89E-44	0.655549882	0.633	0.54	5.21E-40	5-B
Rps27rt	1.53E-255	0.647156051	1	0.998	2.05E-251	5-B
Crem	2.37E-14	0.641352096	0.352	0.28	3.17E-10	5-B
Ncl	1.20E-11	0.624861379	0.403	0.351	1.60E-07	5-B
Gm10045	4.65E-47	0.596864768	0.71	0.622	6.23E-43	5-B
Tsc22d3	2.69E-15	0.593264278	0.483	0.435	3.60E-11	5-B
Serinc3	1.13E-22	0.590731559	0.53	0.459	1.51E-18	5-B
Nr4a1	4.51E-07	0.566186561	0.48	0.47	0.0060381	5-B
Shisa5	4.29E-22	0.561666435	0.586	0.528	5.75E-18	5-B
Stk17b	1.28E-33	0.557372711	0.768	0.725	1.71E-29	5-B
Dennd4a	5.41E-12	0.543104983	0.458	0.412	7.24E-08	5-B
Ly6d	1.50E-18	0.542976895	0.377	0.289	2.01E-14	5-B
P2ry10	3.99E-10	0.540536374	0.338	0.282	5.34E-06	5-B
Cnbp	2.44E-07	0.5142055	0.296	0.252	0.0032702	5-B
**gene**	**p_val**	**avg_log2FC**	**pct.1**	**pct.2**	**p_val_adj**	**cluster**
Hist1h2ao	0	3.556729054	0.652	0.11	0	6-dividing
Top2a	0	3.049498603	0.645	0.049	0	6-dividing
2810417H13Ril	0	2.495441308	0.501	0.036	0	6-dividing
Stmn1	0	2.193778824	0.608	0.112	0	6-dividing
Mki67	0	2.13798641	0.408	0.032	0	6-dividing
Hmgb2	2.19E-178	2.109678893	0.861	0.519	2.93E-174	6-dividing
Tuba1b	3.57E-261	2.030692169	0.72	0.213	4.78E-257	6-dividing
Tubb5	4.23E-190	1.738831023	0.708	0.245	5.66E-186	6-dividing
Hist1h2ae	0	1.62858094	0.277	0.012	0	6-dividing

Nusap1	0	1.542956225	0.274	0.013	0	6-dividing
Birc5	8.26E-268	1.539239063	0.36	0.047	1.11E-263	6-dividing
Smc2	0	1.489928209	0.35	0.035	0	6-dividing
Ube2c	2.40E-213	1.436667841	0.28	0.034	3.22E-209	6-dividing
Rrm2	0	1.436298461	0.291	0.018	0	6-dividing
Cks1b	2.67E-235	1.40056552	0.327	0.043	3.58E-231	6-dividing
Dut	5.38E-223	1.378877308	0.385	0.062	7.21E-219	6-dividing
Ccna2	0	1.314027432	0.252	0.012	0	6-dividing
H2afy	3.02E-96	1.268905607	0.669	0.308	4.04E-92	6-dividing
Dnmt1	5.14E-221	1.252878323	0.398	0.064	6.88E-217	6-dividing
Nucks1	1.61E-163	1.238669408	0.542	0.144	2.15E-159	6-dividing
Mcm3	9.39E-256	1.238175469	0.321	0.037	1.26E-251	6-dividing
H2afv	8.12E-131	1.206735271	0.583	0.193	1.09E-126	6-dividing
Ptma	2.80E-192	1.164987858	0.978	0.815	3.75E-188	6-dividing
Anp32b	3.37E-129	1.163447143	0.58	0.197	4.52E-125	6-dividing
Tmpo	1.16E-128	1.136260154	0.467	0.132	1.56E-124	6-dividing
Dek	1.26E-141	1.134768118	0.46	0.121	1.69E-137	6-dividing
Cdk6	4.34E-141	1.117766349	0.252	0.041	5.80E-137	6-dividing
Hmgb1	1.53E-115	1.102367511	0.683	0.281	2.04E-111	6-dividing
Hmgn2	1.90E-116	1.102146577	0.497	0.159	2.54E-112	6-dividing
Alyref	6.89E-150	1.094685852	0.437	0.104	9.22E-146	6-dividing
Mcm6	2.15E-185	1.093335418	0.302	0.044	2.88E-181	6-dividing
Anp32e	1.85E-127	1.08538103	0.523	0.159	2.48E-123	6-dividing
Cbx5	8.91E-202	1.082737038	0.271	0.033	1.19E-197	6-dividing
Cbx3	1.79E-161	1.069839854	0.498	0.124	2.39E-157	6-dividing
Gm21596	2.62E-113	1.056280016	0.474	0.148	3.51E-109	6-dividing
Mcm5	1.39E-197	1.039858804	0.255	0.03	1.85E-193	6-dividing
Ran	3.51E-90	1.033339143	0.574	0.243	4.69E-86	6-dividing
Rrm1	5.46E-218	1.030781197	0.26	0.028	7.31E-214	6-dividing
H2afz	2.49E-81	1.015001772	0.794	0.518	3.33E-77	6-dividing
Pcna	3.07E-110	1.011589321	0.499	0.161	4.10E-106	6-dividing
Racgap1	6.50E-147	0.986983544	0.271	0.045	8.71E-143	6-dividing
Rpn1	2.69E-84	0.945822372	0.36	0.11	3.60E-80	6-dividing
Ube2s	4.05E-88	0.944034043	0.558	0.222	5.41E-84	6-dividing
D17H6S56E-5	3.47E-99	0.937378618	0.382	0.108	4.64E-95	6-dividing
Smc4	3.15E-121	0.904379059	0.335	0.075	4.22E-117	6-dividing
Arl6ip1	4.19E-66	0.89987937	0.561	0.254	5.61E-62	6-dividing
Tipin	8.73E-191	0.897546151	0.252	0.03	1.17E-186	6-dividing
Atpif1	2.50E-78	0.897472313	0.495	0.195	3.35E-74	6-dividing
Rad21	2.92E-100	0.893079042	0.46	0.144	3.91E-96	6-dividing
Cks2	9.00E-91	0.873817522	0.473	0.164	1.20E-86	6-dividing
Ranbp1	1.25E-99	0.849897945	0.318	0.082	1.67E-95	6-dividing
Set	3.57E-78	0.848019667	0.598	0.259	4.77E-74	6-dividing
Ssrp1	4.92E-106	0.834793804	0.324	0.079	6.59E-102	6-dividing
Nup210	1.23E-89	0.777278816	0.374	0.109	1.65E-85	6-dividing
Lgals1	5.03E-41	0.769381902	0.598	0.343	6.73E-37	6-dividing
Snrpd1	1.06E-90	0.767887904	0.357	0.103	1.42E-86	6-dividing
Cenpa	7.47E-74	0.760474813	0.342	0.108	1.00E-69	6-dividing
Rangap1	3.21E-93	0.754546705	0.289	0.07	4.30E-89	6-dividing
Suz12	9.44E-87	0.743041325	0.296	0.076	1.26E-82	6-dividing
Cmc2	1.29E-113	0.733493927	0.256	0.049	1.73E-109	6-dividing
Tubb4b	6.80E-88	0.733421166	0.38	0.114	9.10E-84	6-dividing
Erh	2.84E-60	0.730523946	0.458	0.193	3.80E-56	6-dividing
Cmtm7	8.90E-57	0.729636788	0.403	0.163	1.19E-52	6-dividing
2700094K13Ril	6.94E-87	0.725891095	0.274	0.069	9.28E-83	6-dividing
Baz1b	1.65E-72	0.718644588	0.327	0.1	2.21E-68	6-dividing
Rbbp7	4.25E-95	0.70246722	0.312	0.078	5.69E-91	6-dividing
Dnajc9	1.32E-94	0.687955021	0.282	0.067	1.77E-90	6-dividing

Hnrnpab	4.60E-63	0.675650808	0.534	0.231	6.15E-59	6-dividing
Cbx1	3.15E-80	0.673070751	0.291	0.078	4.22E-76	6-dividing
Smc3	2.98E-87	0.669311507	0.341	0.095	3.99E-83	6-dividing
Slbp	3.64E-71	0.662090554	0.342	0.109	4.87E-67	6-dividing
Hdgf	1.79E-71	0.659636838	0.312	0.094	2.40E-67	6-dividing
Hnrnpa1	3.13E-53	0.658973526	0.417	0.173	4.19E-49	6-dividing
Rdm1	5.81E-47	0.65657381	0.548	0.267	7.78E-43	6-dividing
Bub3	1.39E-65	0.650401243	0.288	0.088	1.86E-61	6-dividing
Ybx1	4.68E-53	0.650359846	0.498	0.226	6.26E-49	6-dividing
Calm3	1.07E-61	0.65004555	0.366	0.132	1.44E-57	6-dividing
Banf1	8.33E-73	0.649252629	0.314	0.095	1.11E-68	6-dividing
S100a4	7.49E-35	0.644394705	0.558	0.316	1.00E-30	6-dividing
Smc1a	2.07E-104	0.643498519	0.253	0.051	2.77E-100	6-dividing
Bcl2l11	2.56E-18	0.635977848	0.538	0.35	3.42E-14	6-dividing
Hpf1	5.05E-81	0.634657947	0.274	0.07	6.76E-77	6-dividing
Serbp1	1.92E-56	0.628551687	0.67	0.341	2.57E-52	6-dividing
Itgb1	2.28E-44	0.624444126	0.32	0.126	3.05E-40	6-dividing
Ppia	3.02E-43	0.623641607	0.85	0.663	4.04E-39	6-dividing
Larp7	1.43E-60	0.623418662	0.255	0.075	1.91E-56	6-dividing
S100a10	1.06E-34	0.620706377	0.59	0.34	1.42E-30	6-dividing
Itgb7	4.01E-47	0.618382871	0.364	0.15	5.37E-43	6-dividing
Pdia6	6.24E-52	0.611648146	0.381	0.152	8.36E-48	6-dividing
Ywhah	4.54E-58	0.610783122	0.391	0.149	6.08E-54	6-dividing
Snrpe	1.09E-46	0.602787164	0.595	0.309	1.46E-42	6-dividing
**gene**	**p_val**	**avg_log2FC**	**pct.1**	**pct.2**	**p_val_adj**	**cluster**
H2-Eb1	3.20E-187	2.561311854	0.833	0.371	4.28E-183	7-DC
Cst3	2.45E-159	2.556952261	0.892	0.468	3.28E-155	7-DC
H2-Ab1	3.48E-174	2.295244917	0.89	0.483	4.66E-170	7-DC
H2-Aa	1.89E-172	2.290161346	0.831	0.386	2.53E-168	7-DC
Cd74	7.82E-168	2.042966374	0.965	0.713	1.05E-163	7-DC
Cd209a	3.74E-179	1.976837498	0.285	0.034	5.00E-175	7-DC
Id3	8.03E-106	1.819055022	0.348	0.079	1.07E-101	7-DC
H2-DMb1	3.71E-142	1.619088476	0.457	0.103	4.97E-138	7-DC
Pmaip1	2.65E-88	1.511144678	0.551	0.193	3.55E-84	7-DC
Cd83	7.45E-97	1.510573201	0.585	0.206	9.98E-93	7-DC
Gm2a	2.61E-112	1.506633038	0.632	0.219	3.50E-108	7-DC
Ifi30	3.51E-99	1.447119402	0.457	0.134	4.69E-95	7-DC
H2-DMa	9.96E-101	1.383244658	0.498	0.152	1.33E-96	7-DC
Cbfa2t3	1.11E-113	1.382434379	0.39	0.087	1.49E-109	7-DC
Naaa	2.48E-59	1.377888386	0.256	0.068	3.32E-55	7-DC
Hk2	6.37E-73	1.374971127	0.319	0.084	8.52E-69	7-DC
Plbd1	8.34E-112	1.350819357	0.358	0.077	1.12E-107	7-DC
Ccdc88a	1.88E-102	1.2955251	0.325	0.068	2.52E-98	7-DC
Atox1	3.57E-69	1.120417234	0.508	0.191	4.78E-65	7-DC
Klf4	2.83E-65	1.100001836	0.435	0.149	3.79E-61	7-DC
Gng10	4.56E-61	1.081677833	0.461	0.176	6.11E-57	7-DC
Rps27l	5.17E-51	1.044089845	0.496	0.212	6.92E-47	7-DC
Id2	4.52E-33	1.029878915	0.467	0.234	6.05E-29	7-DC
Atf3	5.45E-39	1.026344044	0.409	0.18	7.30E-35	7-DC
Syngr2	1.35E-56	1.014736655	0.453	0.175	1.81E-52	7-DC
Clic4	6.27E-41	1.006593412	0.303	0.109	8.39E-37	7-DC
Ctnna1	1.79E-86	1.002516442	0.258	0.05	2.39E-82	7-DC
Lgals3	3.00E-49	0.955093584	0.626	0.315	4.01E-45	7-DC
S100a4	1.12E-44	0.94883535	0.614	0.317	1.50E-40	7-DC
Vim	8.87E-40	0.912311946	0.492	0.234	1.19E-35	7-DC
Tmem123	1.11E-18	0.901970732	0.289	0.146	1.48E-14	7-DC
Nfkb1	3.60E-46	0.886119888	0.417	0.165	4.82E-42	7-DC
St3gal4	3.17E-32	0.884936889	0.465	0.232	4.24E-28	7-DC

Bhlhe40	5.13E-33	0.843187931	0.449	0.218	6.87E-29	7-DC
Prcp	2.30E-43	0.840494616	0.346	0.129	3.08E-39	7-DC
Calm1	1.31E-34	0.838609361	0.667	0.396	1.76E-30	7-DC
Ywhah	1.34E-43	0.830376148	0.388	0.152	1.80E-39	7-DC
Jak2	3.74E-55	0.818100493	0.311	0.092	5.00E-51	7-DC
Gcnt2	3.25E-27	0.813519267	0.445	0.23	4.34E-23	7-DC
Spag9	4.49E-44	0.809558561	0.35	0.128	6.01E-40	7-DC
Nr4a3	1.76E-33	0.798383105	0.394	0.174	2.36E-29	7-DC
Atpif1	5.96E-34	0.798283811	0.425	0.201	7.97E-30	7-DC
H2afy	2.28E-42	0.790908379	0.622	0.314	3.05E-38	7-DC
Slfn5	1.04E-33	0.755765574	0.283	0.107	1.39E-29	7-DC
Skil	1.58E-33	0.755056311	0.378	0.163	2.12E-29	7-DC
Ptms	1.60E-40	0.753984387	0.335	0.125	2.14E-36	7-DC
Mt1	2.37E-18	0.750535763	0.295	0.149	3.17E-14	7-DC
Anxa5	1.15E-35	0.748604794	0.343	0.14	1.54E-31	7-DC
Napsa	4.97E-32	0.745997323	0.315	0.131	6.65E-28	7-DC
Cd300a	2.46E-30	0.724159773	0.268	0.105	3.30E-26	7-DC
Lrrc58	1.18E-31	0.71878728	0.354	0.156	1.58E-27	7-DC
Psmb8	3.69E-29	0.713991087	0.486	0.257	4.94E-25	7-DC
H2afz	7.31E-30	0.708651248	0.772	0.523	9.78E-26	7-DC
Lsp1	7.83E-38	0.702006603	0.687	0.392	1.05E-33	7-DC
Sh3bgrl	2.17E-37	0.701336488	0.309	0.115	2.90E-33	7-DC
Cdkn1a	3.09E-30	0.700111543	0.384	0.176	4.14E-26	7-DC
Erp29	3.86E-32	0.685159259	0.465	0.226	5.17E-28	7-DC
Rel	2.53E-21	0.682533413	0.26	0.116	3.39E-17	7-DC
Pitpna	1.03E-28	0.672366163	0.378	0.179	1.38E-24	7-DC
Actb	2.54E-63	0.671566207	1	0.973	3.40E-59	7-DC
Fam105a	6.87E-30	0.667695716	0.264	0.103	9.19E-26	7-DC
Myadm	1.46E-36	0.662584718	0.303	0.112	1.95E-32	7-DC
Gpx1	3.31E-35	0.65894438	0.713	0.424	4.43E-31	7-DC
Ctsh	3.18E-28	0.653767526	0.394	0.189	4.26E-24	7-DC
Tmsb4x	6.68E-45	0.648446073	1	0.998	8.94E-41	7-DC
Slk	3.49E-26	0.646522189	0.283	0.121	4.68E-22	7-DC
Ly86	1.25E-21	0.640018184	0.25	0.111	1.68E-17	7-DC
Ms4a6c	1.12E-34	0.638494726	0.449	0.207	1.50E-30	7-DC
Ctss	7.28E-34	0.623995574	0.6	0.319	9.74E-30	7-DC
Psme1	1.87E-29	0.619699668	0.415	0.199	2.51E-25	7-DC
Rtn4	8.33E-34	0.61893351	0.415	0.185	1.11E-29	7-DC
Psap	1.37E-31	0.618086274	0.931	0.753	1.84E-27	7-DC
Baz1a	6.33E-38	0.617049459	0.333	0.125	8.48E-34	7-DC
Hspa1a	6.63E-25	0.611967267	0.638	0.404	8.87E-21	7-DC
Marcksl1	7.91E-16	0.598397785	0.317	0.173	1.06E-11	7-DC
Psme2b	1.20E-23	0.597738681	0.474	0.259	1.61E-19	7-DC
Zfp36	3.05E-21	0.589567443	0.778	0.56	4.09E-17	7-DC
Efhd2	3.11E-26	0.582504324	0.431	0.217	4.16E-22	7-DC
Nr4a2	1.02E-19	0.576678383	0.346	0.179	1.36E-15	7-DC
Cycs	4.02E-25	0.575677298	0.465	0.246	5.38E-21	7-DC
Ifitm3	1.05E-25	0.575467194	0.711	0.45	1.40E-21	7-DC
Nap1l1	1.60E-24	0.572383597	0.303	0.135	2.14E-20	7-DC
Marcks	1.27E-23	0.57017308	0.5	0.275	1.70E-19	7-DC
Csf2rb	8.69E-20	0.568322777	0.28	0.132	1.16E-15	7-DC
Pip4k2a	3.14E-22	0.563117563	0.281	0.128	4.21E-18	7-DC
Rgs1	2.19E-15	0.562903743	0.459	0.285	2.93E-11	7-DC
Klf10	1.64E-18	0.556020089	0.329	0.169	2.19E-14	7-DC
Lgmn	3.56E-22	0.554802873	0.295	0.138	4.76E-18	7-DC
Csf2ra	1.36E-25	0.545383895	0.337	0.153	1.82E-21	7-DC
Psmb9	5.55E-19	0.534278597	0.307	0.156	7.43E-15	7-DC
Klrd1	2.54E-16	0.533989084	0.274	0.138	3.40E-12	7-DC

Tnfaip8	4.07E-23	0.532552935	0.266	0.116	5.45E-19	7-DC
Pomp	4.53E-22	0.5313548	0.49	0.275	6.06E-18	7-DC
Zfand5	2.23E-20	0.521403043	0.469	0.262	2.99E-16	7-DC
Smdt1	3.81E-27	0.516368148	0.526	0.281	5.10E-23	7-DC
Tmem256	1.15E-20	0.511101492	0.252	0.113	1.54E-16	7-DC
Gdi2	4.23E-26	0.510258277	0.48	0.25	5.67E-22	7-DC
Ccr2	9.04E-19	0.509763292	0.366	0.195	1.21E-14	7-DC
Actg1	6.73E-35	0.508734543	0.976	0.88	9.01E-31	7-DC
**gene**	**p_val**	**avg_log2FC**	**pct.1**	**pct.2**	**p_val_adj**	**cluster**
Jchain	0	5.487650835	0.958	0.277	0	8-plasma
Mzb1	0	3.894090497	0.825	0.123	0	8-plasma
Hsp90b1	1.16E-220	3.537453822	0.935	0.425	1.56E-216	8-plasma
Pdia4	0	3.287067885	0.772	0.09	0	8-plasma
Txndc5	0	2.904107988	0.672	0.073	0	8-plasma
Slpi	1.38E-44	2.592516612	0.627	0.338	1.85E-40	8-plasma
Trp53inp1	0	2.377820084	0.69	0.093	0	8-plasma
Rpn1	1.64E-232	2.298609954	0.63	0.108	2.19E-228	8-plasma
Xbp1	3.80E-186	2.242914412	0.802	0.235	5.09E-182	8-plasma
Creld2	3.23E-233	2.229917675	0.455	0.055	4.32E-229	8-plasma
Sec11c	1.07E-164	2.122129174	0.688	0.19	1.44E-160	8-plasma
Edem1	1.04E-204	2.060952549	0.682	0.141	1.39E-200	8-plasma
Sdf2l1	5.82E-180	2.045989548	0.468	0.076	7.79E-176	8-plasma
Hist1h2ao	4.28E-97	2.027559601	0.482	0.125	5.73E-93	8-plasma
Ckap4	0	1.98667386	0.547	0.057	0	8-plasma
Pdia6	9.94E-148	1.968903966	0.61	0.15	1.33E-143	8-plasma
Manf	2.97E-118	1.949722902	0.557	0.153	3.97E-114	8-plasma
Fkbp11	0	1.925749438	0.328	0.009	0	8-plasma
Ssr4	1.48E-148	1.90727513	0.53	0.116	1.99E-144	8-plasma
Fkbp2	7.14E-189	1.858957398	0.552	0.098	9.56E-185	8-plasma
Pycard	2.57E-83	1.858085353	0.59	0.219	3.44E-79	8-plasma
Fam46c	6.69E-214	1.847203987	0.635	0.111	8.96E-210	8-plasma
Derl3	0	1.828750457	0.348	0.013	0	8-plasma
Rexo2	7.06E-158	1.707054886	0.562	0.118	9.45E-154	8-plasma
Eaf2	0	1.702733679	0.335	0.01	0	8-plasma
Pou2af1	1.82E-264	1.661832989	0.41	0.037	2.44E-260	8-plasma
Mt1	1.76E-52	1.661217592	0.428	0.147	2.36E-48	8-plasma
H13	1.27E-119	1.60304581	0.665	0.2	1.70E-115	8-plasma
Spcs1	8.42E-105	1.602770067	0.565	0.168	1.13E-100	8-plasma
Spcs2	7.32E-110	1.539217329	0.627	0.19	9.79E-106	8-plasma
Sec61b	1.43E-85	1.518185951	0.797	0.463	1.92E-81	8-plasma
Vimp	8.24E-124	1.495346289	0.56	0.137	1.10E-119	8-plasma
St6gal1	4.36E-201	1.481319938	0.412	0.049	5.84E-197	8-plasma
Rrbp1	9.42E-100	1.479166848	0.75	0.28	1.26E-95	8-plasma
Calr	2.79E-95	1.46948351	0.765	0.304	3.74E-91	8-plasma
Ddost	1.96E-111	1.435157605	0.49	0.116	2.62E-107	8-plasma
Sel1l	2.55E-136	1.419192186	0.332	0.047	3.42E-132	8-plasma
Serp1	9.48E-90	1.413408021	0.848	0.465	1.27E-85	8-plasma
Ostc	8.74E-105	1.4101487	0.51	0.131	1.17E-100	8-plasma
Sec61a1	1.31E-140	1.392911611	0.452	0.081	1.75E-136	8-plasma
Lman1	1.22E-240	1.390743507	0.318	0.024	1.63E-236	8-plasma
Nme1	3.62E-88	1.386162108	0.64	0.231	4.85E-84	8-plasma
Manea	2.15E-165	1.366418555	0.27	0.026	2.87E-161	8-plasma
Ndufa1	5.06E-90	1.348174766	0.57	0.177	6.78E-86	8-plasma
Krtcap2	6.34E-85	1.345982894	0.515	0.156	8.49E-81	8-plasma
Mtdh	7.19E-95	1.337071146	0.578	0.171	9.63E-91	8-plasma
Spcs3	1.58E-143	1.33046848	0.392	0.062	2.12E-139	8-plasma
Edem2	5.69E-142	1.330292175	0.335	0.046	7.62E-138	8-plasma
Srp9	2.10E-79	1.324905198	0.72	0.319	2.81E-75	8-plasma

Hspa5	5.22E-62	1.321153323	0.848	0.517	6.99E-58	8-plasma
Ndufa4	3.30E-75	1.31162293	0.66	0.261	4.42E-71	8-plasma
Ell2	7.02E-130	1.306267131	0.405	0.07	9.40E-126	8-plasma
Cd24a	7.99E-90	1.303757023	0.517	0.144	1.07E-85	8-plasma
Herpud1	1.20E-69	1.287836944	0.677	0.274	1.61E-65	8-plasma
Ppib	1.74E-63	1.284243509	0.472	0.163	2.33E-59	8-plasma
Ssr2	5.24E-96	1.282932235	0.355	0.073	7.02E-92	8-plasma
Rpn2	3.65E-90	1.280178691	0.445	0.111	4.89E-86	8-plasma
Syvn1	7.53E-129	1.240026766	0.34	0.051	1.01E-124	8-plasma
Kcnn4	7.80E-124	1.227237882	0.352	0.056	1.04E-119	8-plasma
Dnajb11	1.09E-82	1.226703179	0.355	0.081	1.45E-78	8-plasma
Nucb1	3.44E-106	1.224088708	0.282	0.043	4.60E-102	8-plasma
Sec61g	6.13E-78	1.213891093	0.565	0.189	8.20E-74	8-plasma
Ssr3	7.04E-80	1.211065956	0.562	0.179	9.42E-76	8-plasma
Selk	3.70E-67	1.209461197	0.723	0.345	4.95E-63	8-plasma
Mef2c	1.52E-83	1.19052264	0.645	0.214	2.03E-79	8-plasma
Sub1	1.21E-68	1.173443237	0.912	0.7	1.62E-64	8-plasma
Eif5b	2.47E-73	1.15289542	0.6	0.207	3.31E-69	8-plasma
Rps27l	8.01E-53	1.148136464	0.53	0.213	1.07E-48	8-plasma
Cd79a	1.04E-97	1.143820989	0.5	0.126	1.39E-93	8-plasma
Hdlbp	2.71E-86	1.114811318	0.352	0.075	3.63E-82	8-plasma
Sec24d	3.89E-106	1.110920682	0.282	0.042	5.21E-102	8-plasma
Ube2j1	1.49E-95	1.108852521	0.405	0.088	1.99E-91	8-plasma
Bet1	3.20E-93	1.105712521	0.295	0.052	4.28E-89	8-plasma
Cd79b	4.34E-101	1.105193553	0.352	0.066	5.81E-97	8-plasma
Gm4184	6.94E-57	1.09276813	0.752	0.413	9.29E-53	8-plasma
Txndc11	5.95E-142	1.081623284	0.292	0.035	7.97E-138	8-plasma
P4hb	1.74E-74	1.078304944	0.468	0.135	2.33E-70	8-plasma
Atp5g1	2.53E-55	1.064403556	0.502	0.187	3.38E-51	8-plasma
Nme2	1.17E-52	1.059747796	0.645	0.3	1.56E-48	8-plasma
Rpl36al	3.02E-72	1.029286117	0.92	0.634	4.05E-68	8-plasma
Prdm1	9.45E-86	1.028814556	0.268	0.045	1.26E-81	8-plasma
Pdia3	4.14E-54	1.015130054	0.525	0.198	5.55E-50	8-plasma
Fbxw7	8.55E-89	0.997365481	0.278	0.047	1.14E-84	8-plasma
Magt1	3.59E-63	0.98572358	0.398	0.113	4.80E-59	8-plasma
Stt3a	1.51E-79	0.976092457	0.352	0.079	2.02E-75	8-plasma
Dnajc3	1.41E-66	0.975811614	0.322	0.077	1.89E-62	8-plasma
Tnfrsf13b	2.12E-66	0.970519249	0.28	0.06	2.84E-62	8-plasma
Cdv3	9.56E-77	0.967127081	0.432	0.113	1.28E-72	8-plasma
Tmed9	1.40E-51	0.955767669	0.465	0.167	1.87E-47	8-plasma
Mrpl57	2.04E-54	0.944396632	0.412	0.133	2.73E-50	8-plasma
Clptm1l	1.02E-90	0.937587835	0.362	0.075	1.37E-86	8-plasma
Irf4	1.24E-61	0.936916734	0.295	0.069	1.66E-57	8-plasma
Glipr1	6.29E-67	0.928516199	0.298	0.067	8.42E-63	8-plasma
Msi2	1.34E-69	0.927546854	0.328	0.075	1.79E-65	8-plasma
Vcp	6.04E-57	0.924807453	0.49	0.167	8.08E-53	8-plasma
Tmed10	8.06E-49	0.913013367	0.452	0.166	1.08E-44	8-plasma
Kdelr2	3.57E-78	0.912829642	0.27	0.05	4.78E-74	8-plasma
Tmed2	5.91E-56	0.900103343	0.552	0.206	7.91E-52	8-plasma
Erp44	7.53E-61	0.896100448	0.298	0.072	1.01E-56	8-plasma
Derl1	6.07E-58	0.888606037	0.372	0.107	8.13E-54	8-plasma
Rps29	8.00E-58	0.88458538	0.295	0.073	1.07E-53	8-plasma
Ly6a	2.39E-31	0.880031059	0.35	0.139	3.20E-27	8-plasma
1700017B05Ril	1.27E-53	0.879572129	0.29	0.074	1.70E-49	8-plasma
Uqcrq	1.08E-42	0.87379316	0.667	0.326	1.44E-38	8-plasma
Mlec	2.86E-64	0.861447151	0.295	0.067	3.83E-60	8-plasma
Surf4	1.80E-47	0.846343797	0.375	0.122	2.41E-43	8-plasma
Tmem167	4.20E-40	0.833934697	0.44	0.172	5.62E-36	8-plasma

Srp19	3.78E-41	0.807689585	0.278	0.082	5.06E-37	8-plasma
Snd1	4.09E-69	0.805631134	0.265	0.053	5.47E-65	8-plasma
Lrrc59	1.13E-66	0.805425068	0.295	0.065	1.51E-62	8-plasma
Rftn1	1.70E-48	0.800423416	0.258	0.065	2.28E-44	8-plasma
Cpeb2	7.02E-49	0.79367073	0.3	0.082	9.40E-45	8-plasma
Pafah1b3	2.54E-52	0.7920485	0.255	0.061	3.39E-48	8-plasma
Srpr	8.22E-57	0.786853415	0.252	0.056	1.10E-52	8-plasma
Lman2	3.59E-48	0.782172012	0.39	0.128	4.81E-44	8-plasma
Top1	2.59E-45	0.77305915	0.455	0.166	3.46E-41	8-plasma
Ufm1	3.18E-56	0.772540504	0.285	0.069	4.26E-52	8-plasma
Glcci1	2.72E-39	0.767309153	0.298	0.092	3.64E-35	8-plasma
Atp5j2	5.93E-38	0.764170847	0.603	0.284	7.94E-34	8-plasma
Ebp	1.80E-40	0.758211723	0.275	0.082	2.41E-36	8-plasma
Cd69	1.43E-41	0.755958032	0.445	0.165	1.91E-37	8-plasma
Nmt1	2.06E-40	0.754236136	0.435	0.163	2.75E-36	8-plasma
Gm42035	1.18E-32	0.748784185	0.292	0.101	1.57E-28	8-plasma
Egr1	6.93E-26	0.744765602	0.318	0.128	9.28E-22	8-plasma
Man1a	4.00E-59	0.731536491	0.332	0.085	5.35E-55	8-plasma
Mydgf	8.84E-42	0.725101881	0.25	0.069	1.18E-37	8-plasma
Wbp5	1.21E-51	0.721909349	0.365	0.108	1.62E-47	8-plasma
Morf4l2	3.49E-38	0.721205218	0.388	0.141	4.67E-34	8-plasma
Tmem248	8.10E-52	0.717551775	0.278	0.07	1.08E-47	8-plasma
Ssr1	4.37E-51	0.709723865	0.358	0.106	5.85E-47	8-plasma
Map2k2	1.93E-31	0.704251543	0.295	0.104	2.58E-27	8-plasma
Golga4	4.02E-33	0.703282841	0.258	0.081	5.39E-29	8-plasma
Bsg	9.18E-36	0.70207581	0.402	0.157	1.23E-31	8-plasma
Tmem160	2.07E-33	0.701318258	0.49	0.217	2.78E-29	8-plasma
Rap1a	2.50E-33	0.685553206	0.505	0.225	3.35E-29	8-plasma
Ndufb9	8.84E-32	0.667220034	0.415	0.174	1.18E-27	8-plasma
Canx	1.02E-32	0.666899718	0.62	0.308	1.37E-28	8-plasma
Nars	1.21E-44	0.665078355	0.28	0.078	1.63E-40	8-plasma
Ndufb11	2.24E-33	0.664388311	0.512	0.229	3.00E-29	8-plasma
Zfp706	6.25E-41	0.662047332	0.382	0.134	8.36E-37	8-plasma
Eif2s2	8.98E-28	0.661695193	0.67	0.375	1.20E-23	8-plasma
Tcf3	6.40E-38	0.657815131	0.26	0.076	8.56E-34	8-plasma
Cdc42se2	7.82E-51	0.657768628	0.36	0.106	1.05E-46	8-plasma
Pkig	1.52E-34	0.653548954	0.295	0.1	2.04E-30	8-plasma
Dhrsx	4.32E-44	0.652233175	0.255	0.067	5.79E-40	8-plasma
Ganab	2.44E-48	0.65116201	0.26	0.065	3.27E-44	8-plasma
Polr2l	2.11E-35	0.649250644	0.608	0.286	2.82E-31	8-plasma
Ndfip1	5.82E-35	0.648802523	0.44	0.177	7.79E-31	8-plasma
Cox17	4.26E-27	0.647695155	0.713	0.416	5.70E-23	8-plasma
Ube2s	4.69E-27	0.641397729	0.482	0.23	6.28E-23	8-plasma
Atp1b1	1.48E-24	0.640640334	0.312	0.126	1.98E-20	8-plasma
Ost4	9.25E-29	0.63407582	0.432	0.192	1.24E-24	8-plasma
Uqcr11	1.09E-30	0.633068797	0.388	0.158	1.46E-26	8-plasma
Pabpc4	2.82E-30	0.630470117	0.338	0.126	3.77E-26	8-plasma
Man2a1	1.13E-38	0.630466663	0.28	0.084	1.51E-34	8-plasma
Itgb7	2.56E-31	0.626293065	0.385	0.154	3.43E-27	8-plasma
Gorasp2	1.45E-49	0.624636497	0.27	0.068	1.94E-45	8-plasma
Tram1	7.47E-35	0.624203729	0.382	0.144	1.00E-30	8-plasma
Ndufs6	5.48E-35	0.62396549	0.388	0.147	7.33E-31	8-plasma
Cmah	1.98E-46	0.61230457	0.415	0.136	2.65E-42	8-plasma
Tm9sf3	9.65E-31	0.609479994	0.41	0.169	1.29E-26	8-plasma
Cd28	1.22E-42	0.608821757	0.378	0.124	1.64E-38	8-plasma
Psmb5	5.19E-28	0.608127565	0.378	0.159	6.94E-24	8-plasma
Prdx1	1.48E-23	0.606741717	0.547	0.294	1.98E-19	8-plasma
Erh	1.97E-24	0.593728556	0.425	0.198	2.64E-20	8-plasma

Tmco1	9.93E-26	0.593490947	0.355	0.151	1.33E-21	8-plasma
Tmem208	4.63E-42	0.59265621	0.3	0.09	6.20E-38	8-plasma
Anapc5	3.98E-34	0.591386238	0.302	0.102	5.33E-30	8-plasma
Tubb4b	2.02E-31	0.587959025	0.328	0.121	2.70E-27	8-plasma
Atp5g3	1.54E-31	0.586094748	0.525	0.238	2.06E-27	8-plasma
Sars	2.33E-33	0.574260282	0.308	0.106	3.12E-29	8-plasma
Swi5	3.20E-32	0.572704439	0.368	0.141	4.28E-28	8-plasma
Dnajb9	8.08E-35	0.572319135	0.29	0.094	1.08E-30	8-plasma
Preb	1.38E-39	0.569868933	0.332	0.108	1.85E-35	8-plasma
Edf1	9.60E-25	0.569479488	0.35	0.151	1.29E-20	8-plasma
Ndufs5	8.68E-35	0.568288838	0.362	0.133	1.16E-30	8-plasma
Rpl41	2.48E-53	0.568195104	1	0.996	3.32E-49	8-plasma
Ndufb7	2.82E-25	0.56390932	0.4	0.179	3.77E-21	8-plasma
Reep5	2.47E-28	0.562133758	0.348	0.14	3.31E-24	8-plasma
Mdh1	3.88E-32	0.55831515	0.31	0.11	5.19E-28	8-plasma
Hn1	5.73E-21	0.557604957	0.455	0.233	7.67E-17	8-plasma
Lat2	1.62E-28	0.547562099	0.26	0.09	2.17E-24	8-plasma
Ubxn4	2.42E-32	0.542571863	0.318	0.112	3.24E-28	8-plasma
Mrpl54	5.51E-24	0.537576229	0.29	0.117	7.37E-20	8-plasma
Dad1	7.69E-26	0.535222462	0.495	0.241	1.03E-21	8-plasma
Cd274	5.89E-33	0.533490283	0.265	0.084	7.89E-29	8-plasma
Prkcsh	4.22E-32	0.531875322	0.255	0.081	5.65E-28	8-plasma
Ptp4a2	9.27E-25	0.530492019	0.568	0.293	1.24E-20	8-plasma
Psmd8	5.80E-25	0.529733806	0.288	0.112	7.76E-21	8-plasma
D17H6S56E-5	3.03E-19	0.528336964	0.272	0.116	4.06E-15	8-plasma
Mrps14	9.34E-28	0.525961376	0.392	0.167	1.25E-23	8-plasma
Copa	2.28E-31	0.524300049	0.252	0.081	3.06E-27	8-plasma
Blnk	8.92E-46	0.523289885	0.262	0.068	1.19E-41	8-plasma
Spn	6.73E-24	0.523133243	0.308	0.125	9.00E-20	8-plasma
Ccnd2	6.47E-32	0.516891983	0.46	0.19	8.66E-28	8-plasma
Ier3ip1	3.24E-30	0.513966029	0.485	0.213	4.34E-26	8-plasma
Cox6a1	1.56E-21	0.512549307	0.765	0.491	2.08E-17	8-plasma
Smchd1	2.64E-31	0.511606195	0.342	0.126	3.53E-27	8-plasma
**gene**	**p_val**	**avg_log2FC**	**pct.1**	**pct.2**	**p_val_adj**	**cluster**
Camp	0	6.222808634	0.989	0.145	0	9-Granulocytes2
Ngp	1.58E-291	5.820298659	1	0.251	2.12E-287	9-Granulocytes2
Ltf	0	5.155157893	0.938	0.044	0	9-Granulocytes2
Lcn2	2.70E-292	3.709573461	0.982	0.212	3.62E-288	9-Granulocytes2
Ifitm6	0	3.289248795	0.882	0.13	0	9-Granulocytes2
Chil3	3.00E-199	3.239668771	0.761	0.15	4.01E-195	9-Granulocytes2
S100a8	2.61E-156	3.146578685	1	0.93	3.50E-152	9-Granulocytes2
Anxa1	4.49E-300	2.969273853	0.934	0.159	6.00E-296	9-Granulocytes2
Cd177	0	2.969144743	0.79	0.057	0	9-Granulocytes2
Wfdc21	5.54E-211	2.950551533	1	0.315	7.42E-207	9-Granulocytes2
S100a9	3.34E-154	2.819118811	1	0.967	4.47E-150	9-Granulocytes2
Pglyrp1	8.62E-218	2.585629481	0.923	0.209	1.15E-213	9-Granulocytes2
Mmp9	1.54E-161	2.382308889	0.728	0.147	2.06E-157	9-Granulocytes2
Adpgk	1.42E-268	2.283193891	0.647	0.07	1.90E-264	9-Granulocytes2
Mmp8	4.00E-102	2.174747646	0.61	0.154	5.36E-98	9-Granulocytes2
Dstn	9.96E-195	2.135955576	0.699	0.114	1.33E-190	9-Granulocytes2
G0s2	5.37E-114	2.115633289	0.952	0.406	7.19E-110	9-Granulocytes2
Syne1	0	1.965982888	0.504	0.034	0	9-Granulocytes2
Serpinb1a	2.78E-218	1.95458881	0.452	0.042	3.72E-214	9-Granulocytes2
Hmgn2	2.38E-90	1.916084783	0.607	0.166	3.19E-86	9-Granulocytes2
Ckap4	2.48E-187	1.912839055	0.511	0.061	3.32E-183	9-Granulocytes2
I830127L07Rik	9.06E-136	1.860766049	0.441	0.064	1.21E-131	9-Granulocytes2
Aldh2	8.72E-151	1.837705758	0.676	0.129	1.17E-146	9-Granulocytes2
AA467197	7.74E-195	1.73497129	0.39	0.034	1.04E-190	9-Granulocytes2

Tkt	1.70E-98	1.73306579	0.812	0.274	2.27E-94	9-Granulocytes2
Retnlg	1.17E-64	1.704884311	0.974	0.682	1.57E-60	9-Granulocytes2
St3gal5	2.49E-181	1.570367012	0.327	0.025	3.33E-177	9-Granulocytes2
Fpr2	1.08E-120	1.515946442	0.408	0.057	1.45E-116	9-Granulocytes2
Cd9	1.19E-104	1.503357611	0.629	0.144	1.59E-100	9-Granulocytes2
Gadd45a	1.21E-73	1.477327949	0.728	0.249	1.62E-69	9-Granulocytes2
Mcemp1	2.02E-96	1.461330709	0.621	0.151	2.70E-92	9-Granulocytes2
Mgst1	5.25E-81	1.43172016	0.562	0.144	7.03E-77	9-Granulocytes2
Plscr1	1.72E-125	1.415920874	0.375	0.047	2.30E-121	9-Granulocytes2
Hp	9.07E-76	1.380910927	0.871	0.378	1.21E-71	9-Granulocytes2
Ltb4r1	1.15E-108	1.371175982	0.357	0.049	1.55E-104	9-Granulocytes2
Mettl9	8.95E-81	1.307981599	0.382	0.072	1.20E-76	9-Granulocytes2
Trem3	5.89E-104	1.301038231	0.375	0.056	7.88E-100	9-Granulocytes2
Anxa3	2.33E-119	1.300714151	0.261	0.025	3.11E-115	9-Granulocytes2
Itgam	1.06E-84	1.29760898	0.54	0.125	1.41E-80	9-Granulocytes2
Osm	5.72E-54	1.253683739	0.566	0.186	7.65E-50	9-Granulocytes2
Ly6g	2.90E-91	1.248209903	0.305	0.042	3.88E-87	9-Granulocytes2
Sgms2	5.66E-76	1.203335607	0.331	0.056	7.57E-72	9-Granulocytes2
Pygl	1.24E-80	1.183452426	0.449	0.092	1.67E-76	9-Granulocytes2
Cpne3	4.22E-65	1.18156949	0.397	0.088	5.64E-61	9-Granulocytes2
Glrx	1.34E-56	1.177573152	0.36	0.083	1.79E-52	9-Granulocytes2
Lrg1	2.55E-69	1.171199436	0.574	0.16	3.42E-65	9-Granulocytes2
Mrgpra2b	1.40E-78	1.154966805	0.327	0.053	1.88E-74	9-Granulocytes2
Nfe2	1.29E-54	1.138658471	0.305	0.063	1.73E-50	9-Granulocytes2
Cybb	2.02E-57	1.129673168	0.779	0.315	2.71E-53	9-Granulocytes2
Hmgb2	1.94E-38	1.105920869	0.886	0.528	2.59E-34	9-Granulocytes2
Cd24a	6.22E-58	1.095176017	0.515	0.147	8.33E-54	9-Granulocytes2
Slfn4	1.89E-46	1.086549451	0.423	0.12	2.53E-42	9-Granulocytes2
Vasp	1.11E-45	1.084467217	0.688	0.275	1.48E-41	9-Granulocytes2
S100a6	8.60E-28	1.078900622	0.904	0.684	1.15E-23	9-Granulocytes2
Npepps	6.32E-58	1.077902314	0.335	0.071	8.46E-54	9-Granulocytes2
Arhgdib	2.91E-41	1.062969149	0.783	0.402	3.89E-37	9-Granulocytes2
Chil1	3.46E-70	1.040231634	0.276	0.043	4.63E-66	9-Granulocytes2
Arrb2	8.55E-49	1.023019897	0.441	0.126	1.14E-44	9-Granulocytes2
Ncf1	7.23E-55	1.006066239	0.754	0.291	9.67E-51	9-Granulocytes2
Degs1	1.02E-42	1.004283061	0.43	0.134	1.36E-38	9-Granulocytes2
C3	1.04E-54	0.95424713	0.32	0.069	1.39E-50	9-Granulocytes2
Pbx1	1.90E-42	0.942182833	0.25	0.053	2.55E-38	9-Granulocytes2
Lyz2	9.26E-67	0.91394789	0.857	0.329	1.24E-62	9-Granulocytes2
Clec4a2	3.16E-57	0.905699468	0.272	0.05	4.23E-53	9-Granulocytes2
Plbd1	3.99E-30	0.898477225	0.279	0.082	5.34E-26	9-Granulocytes2
Klf7	2.57E-40	0.892118364	0.316	0.081	3.45E-36	9-Granulocytes2
Pgd	3.66E-43	0.887215723	0.463	0.146	4.90E-39	9-Granulocytes2
Trib1	1.23E-31	0.884338209	0.522	0.214	1.65E-27	9-Granulocytes2
Hdc	2.46E-45	0.880695971	0.783	0.335	3.29E-41	9-Granulocytes2
Plaur	1.31E-36	0.872864218	0.647	0.27	1.76E-32	9-Granulocytes2
Txn1	1.06E-37	0.868908992	0.772	0.386	1.42E-33	9-Granulocytes2
Thbs1	5.22E-22	0.853626584	0.57	0.287	6.99E-18	9-Granulocytes2
Gsr	1.04E-38	0.851351469	0.776	0.365	1.39E-34	9-Granulocytes2
Pnkp	1.90E-54	0.849017306	0.265	0.049	2.54E-50	9-Granulocytes2
Slc2a3	8.89E-35	0.829239211	0.254	0.063	1.19E-30	9-Granulocytes2
Adam8	1.20E-33	0.827629844	0.342	0.104	1.61E-29	9-Granulocytes2
C5ar1	1.06E-35	0.827154935	0.364	0.111	1.41E-31	9-Granulocytes2
Pirb	4.32E-39	0.819739286	0.5	0.173	5.78E-35	9-Granulocytes2
S100a11	1.24E-42	0.813904251	0.908	0.535	1.67E-38	9-Granulocytes2
Lasp1	3.53E-30	0.813839184	0.353	0.116	4.73E-26	9-Granulocytes2
Shfm1	1.16E-41	0.808482204	0.978	0.668	1.56E-37	9-Granulocytes2
Lilr4b	2.30E-34	0.806246202	0.478	0.17	3.07E-30	9-Granulocytes2

Fam101b	1.62E-44	0.799954189	0.272	0.059	2.16E-40	9-Granulocytes2
Atxn10	4.99E-25	0.795324813	0.257	0.081	6.68E-21	9-Granulocytes2
Mapk3	1.47E-32	0.795094893	0.309	0.091	1.97E-28	9-Granulocytes2
Gpi1	5.01E-28	0.794227876	0.507	0.216	6.70E-24	9-Granulocytes2
Pilra	8.84E-36	0.792953301	0.29	0.078	1.18E-31	9-Granulocytes2
Dgat1	9.09E-35	0.792864804	0.614	0.25	1.22E-30	9-Granulocytes2
Ncf4	7.09E-32	0.784236897	0.386	0.132	9.49E-28	9-Granulocytes2
Alox5ap	8.26E-35	0.77397134	0.79	0.382	1.11E-30	9-Granulocytes2
Actn1	3.18E-36	0.757635159	0.309	0.083	4.26E-32	9-Granulocytes2
Trem1	2.47E-27	0.751520435	0.36	0.127	3.30E-23	9-Granulocytes2
Lims1	3.54E-33	0.749337309	0.419	0.143	4.74E-29	9-Granulocytes2
Ap3s1	6.93E-32	0.746704273	0.331	0.103	9.28E-28	9-Granulocytes2
Vsir	6.75E-29	0.746134581	0.496	0.201	9.04E-25	9-Granulocytes2
Cyba	2.19E-29	0.73918167	0.857	0.51	2.93E-25	9-Granulocytes2
Ccnd3	5.08E-31	0.731277115	0.441	0.161	6.80E-27	9-Granulocytes2
Gapdh	2.47E-27	0.718575221	0.757	0.395	3.30E-23	9-Granulocytes2
Gsn	1.09E-39	0.715718814	0.368	0.104	1.46E-35	9-Granulocytes2
Cebpd	1.31E-28	0.706308089	0.261	0.075	1.76E-24	9-Granulocytes2
Mpc2	7.53E-24	0.693507996	0.324	0.118	1.01E-19	9-Granulocytes2
Scp2	5.57E-24	0.688927098	0.449	0.19	7.45E-20	9-Granulocytes2
Itgb2	2.87E-27	0.684043478	0.364	0.129	3.84E-23	9-Granulocytes2
Flna	2.76E-25	0.677995681	0.504	0.217	3.69E-21	9-Granulocytes2
Slc16a3	3.10E-32	0.674251694	0.489	0.181	4.15E-28	9-Granulocytes2
1810037I17Rik	6.62E-20	0.662413854	0.662	0.365	8.86E-16	9-Granulocytes2
Fbxl5	1.05E-26	0.661549373	0.438	0.169	1.41E-22	9-Granulocytes2
Clec12a	3.35E-20	0.641690377	0.301	0.114	4.48E-16	9-Granulocytes2
Spi1	3.54E-27	0.63808312	0.577	0.25	4.74E-23	9-Granulocytes2
Mxd1	2.87E-24	0.627447592	0.71	0.365	3.84E-20	9-Granulocytes2
Pkm	1.03E-20	0.613595272	0.596	0.3	1.38E-16	9-Granulocytes2
Lst1	6.99E-28	0.5924129	0.596	0.259	9.36E-24	9-Granulocytes2
Gda	2.67E-30	0.589966326	0.64	0.274	3.57E-26	9-Granulocytes2
App	9.98E-23	0.582659767	0.368	0.141	1.34E-18	9-Granulocytes2
Lamtor4	1.14E-16	0.581261224	0.393	0.182	1.53E-12	9-Granulocytes2
Gyg	2.91E-26	0.580963229	0.287	0.091	3.89E-22	9-Granulocytes2
Lmnb1	1.10E-26	0.580742014	0.658	0.302	1.47E-22	9-Granulocytes2
Pfn1	3.87E-22	0.568857	0.919	0.654	5.19E-18	9-Granulocytes2
Tecr	4.89E-21	0.566496433	0.254	0.086	6.54E-17	9-Granulocytes2
Lmo4	4.60E-24	0.563005003	0.382	0.145	6.15E-20	9-Granulocytes2
Aprt	1.38E-20	0.556035588	0.382	0.159	1.85E-16	9-Granulocytes2
Pgk1	4.74E-17	0.537430531	0.386	0.176	6.35E-13	9-Granulocytes2
Arpc5	1.68E-16	0.535820826	0.688	0.397	2.25E-12	9-Granulocytes2
Grina	1.05E-23	0.533373183	0.688	0.345	1.40E-19	9-Granulocytes2
Pi16	5.97E-24	0.52876095	0.265	0.083	7.99E-20	9-Granulocytes2
Il1r2	2.71E-24	0.523637193	0.371	0.136	3.62E-20	9-Granulocytes2

Supplementary Table 4

gene	avg_log2FC	pct.1	pct.2	p_val_adj
Lyz2	2.507015568	0.958	0.246	9.29E-125
LOC1008624	2.378619883	0.978	0.36	2.56E-36
Ftl1	2.377513411	0.989	0.392	9.92E-175
Apoe	2.004497432	0.829	0.135	2.44E-135
Rps5	1.935833235	0.88	0.308	9.25E-178
Ifitm2	1.767374988	0.955	0.352	8.18E-75
Sh3bgrl3	1.754186318	0.968	0.345	3.38E-170
Rps2	1.714160962	0.887	0.326	8.05E-193
Fcer1g	1.600740288	0.97	0.46	1.52E-172
Lgals3	1.596886457	0.855	0.247	6.75E-164
Rps6	1.574398164	0.777	0.168	7.14E-94
Elane	1.545475423	0.437	0.015	1.43E-191
LOC1052442	1.541195856	0.965	0.595	1.58E-101
Jchain	1.532579223	0.954	0.197	9.78E-155
Ubb	1.530590196	0.998	0.641	2.21E-115
Ppib	1.497373235	0.68	0.095	2.47E-140
Rplp0	1.445391352	0.973	0.628	1.06E-189
Lgals1	1.406506655	0.84	0.283	1.06E-111
Ccl9	1.40469098	0.534	0.075	2.45E-79
Manf	1.395048544	0.637	0.093	8.64E-88
Alox5ap	1.386879412	0.889	0.315	7E-133
Ccl5	1.370983848	0.992	0.597	5.33E-147
Prtn3	1.370635223	0.438	0.021	1.13E-142
Mzb1	1.368412117	0.589	0.074	3.73E-199
Rpl14	1.275376466	0.985	0.636	1.46E-145
Gm13202	1.260546184	0.709	0.133	1.22E-50
Ifitm3	1.259093607	0.941	0.387	2.12E+41
Emd	1.230186192	0.918	0.308	5.22E-46
Slpi	1.222849739	0.857	0.27	1.78E-162
Uba52	1.211038959	0.998	0.832	1.12E-111
Ly6c2	1.204886216	0.842	0.277	9.14E-200
D8Ertd738e	1.193561649	0.84	0.21	108000000
Ly6d	1.176602938	0.781	0.225	1.15E-185
15-Sep	1.167295944	0.518	0.058	3.97E-103
Tspo	1.159163909	0.915	0.352	3.64E-73
Btf3	1.157583828	0.897	0.328	3.14E-184
Brk1	1.15752348	0.587	0.079	1.62E-115
Gm6745	1.150431977	0.447	0.036	1.75E-59
Ifitm1	1.143964454	0.885	0.311	3.61E-153
Ctsz	1.143467364	0.709	0.173	2.35E-135
Rpl29	1.129427067	0.947	0.449	5.5E-188
Cstb	1.118059799	0.622	0.12	8.53E-171
Cst3	1.111725972	0.938	0.414	9.98E-160
Ppp1ca	1.099746292	0.683	0.133	1.65E-184
H2-Ab1	1.095317821	0.936	0.431	2.13E-185
S100a10	1.09507092	0.856	0.277	3.09E-33
Eno1	1.094880008	0.466	0.065	1.98E-177
Cyba	1.084333177	0.947	0.452	3.94E-129
Gpx1	1.070646919	0.892	0.366	3.4E-176
Rps18	1.062381228	0.9	0.654	4.7E-161
Cd52	1.062136308	0.976	0.553	1.25E+16
Cd74	1.05389375	0.994	0.681	1.7E-145
Rpl15	1.053232233	0.983	0.624	2.43E-149
Fth1	1.039721903	1	0.887	1.61E-194
Ctss	1.035552143	0.75	0.265	1.16E-75

Ctsh	1.033995359	0.602	0.136	1.33E-104
Vamp8	1.016489148	0.749	0.189	1.4E-171
Rpl19	1.016189979	0.972	0.666	9.02E-82
Atp5d	1.01440357	0.683	0.145	4.6E-159
Arpc1b	1.008952189	0.792	0.251	9.68E-169
Sepw1	1.0033308	0.849	0.272	1.55E-158
Chil3	0.997617749	0.531	0.105	2.04E-172
Sdf2l1	0.992428266	0.37	0.044	3.22E-192
Gm25380	0.986383797	0.925	0.562	1.8518E-100
Eif5a	0.984962974	0.767	0.219	1.13E-124
Vim	0.983855983	0.648	0.182	1.47E-90
Prdx1	0.983453225	0.764	0.232	9.63E-179
Ccl6	0.981692163	0.84	0.289	4.79E-96
Cnbp	0.977843095	0.759	0.182	2.54E-86
Msrb1	0.977257078	0.885	0.369	3.78E-159
Myl12b	0.976554367	0.668	0.136	4.3E-132
Edf1	0.969877795	0.57	0.096	4.69E+65
Arhgdib	0.968015797	0.903	0.336	5.22E-09
Id3	0.963384587	0.294	0.057	1.01E-166
Hint1	0.9554233	0.822	0.263	1.1E-193
Ifi30	0.952094916	0.48	0.094	4.86E-33
Ctsg	0.950926352	0.199	0.006	8.13E-182
Atp6v1f	0.94275461	0.727	0.179	2.12E-80
Cdk2ap2	0.940442852	0.782	0.217	4.62E-190
Cd14	0.936743889	0.702	0.214	1.14E-194
Psmb8	0.933727198	0.733	0.196	2.48E-83
Ssr2	0.933718171	0.362	0.039	1.38E-190
Rps11	0.928247161	0.921	0.614	8.25E-171
Psmb2	0.924842849	0.46	0.067	5.51E-95
Park7	0.92285784	0.595	0.114	1.93E-167
Myl12a	0.918765116	0.755	0.208	5.85E-78
Ldha	0.918520209	0.645	0.143	2.76E-134
Psmb3	0.918419283	0.327	0.022	5.98E-84
Gpx4	0.918305584	0.645	0.14	3.61E-80
Ppia	0.917428227	0.979	0.626	4.4E-146
Fam96a	0.903494582	0.458	0.071	1.17E-134
Nme2	0.902741369	0.742	0.245	4.15E-151
Atp5c1	0.900313236	0.379	0.038	3.21E-171
H2-Aa	0.899910205	0.827	0.337	6.73E-194
Cycs	0.89972269	0.694	0.188	8.31E-159
Rps10	0.897688924	0.801	0.291	1.9E-103
Ssr4	0.89671343	0.451	0.078	3.24E-174
Gngt2	0.895500583	0.56	0.129	2.71E-146
Eno1b	0.8935374	0.267	0.01	2.28E-135
Aldoa	0.887704537	0.634	0.136	5.41E-153
Pfn1	0.882934788	0.978	0.611	2.44E-152
Emp3	0.880632563	0.608	0.158	1.28E-137
Nedd8	0.877583342	0.621	0.135	1.23E-95
Plac8	0.875663591	0.955	0.511	4.09E-106
LOC1026356(	0.871535259	0.786	0.255	1.64E+17
Fis1	0.865169028	0.699	0.167	1.45E+21
Uqcrfs1	0.860795534	0.551	0.108	8.81E-29
Rpl32	0.85769405	0.96	0.77	2.91E-128
Ramp1	0.854605969	0.419	0.061	4.76E-182
Rpl13	0.852705606	0.991	0.858	3.04E-178
Sumo2	0.851651029	0.646	0.149	3.86E-149
Rpl6	0.849076642	0.926	0.564	2.44E-188
Akr1a1	0.846344319	0.398	0.055	1.34E-145

Gm8210	0.845126097	0.232	0.003	6.02E-183
Slc25a5	0.842236252	0.715	0.219	3.75E-184
Npc2	0.841512182	0.874	0.38	1.48E-182
Rpl21	0.840775169	0.885	0.498	4.38E-177
Eif1	0.839801612	0.993	0.695	5.42E+43
Cox5a	0.838618912	0.513	0.096	3.99E-143
Stmn1	0.833081557	0.456	0.085	5.04E-21
H2-DMa	0.832086148	0.503	0.113	3.29E-188
Tyrobp	0.831120609	0.955	0.641	7E-153
Gng10	0.830377827	0.553	0.131	1.45E-65
Grpel1	0.829901734	0.307	0.022	8.46E-173
Hilpda	0.827016953	0.452	0.088	4.72E-132
Pfdn5	0.823513409	0.911	0.382	2.1E-71
Rgs10	0.822222707	0.495	0.089	1.64E-134
Itm2b	0.821169591	0.901	0.416	8.18E-115
Lrrc58	0.819309921	0.512	0.111	1.1E-193
Arpc3	0.814409449	0.841	0.298	2.61E-51
Rps14	0.812580073	0.93	0.675	3.67E-115
Gabarap	0.811910901	0.829	0.288	0.0097
Oaz1	0.810098985	0.98	0.576	3.71E-51
Prelid1	0.804324983	0.487	0.087	1.44E-83
Spcs1	0.802548375	0.564	0.121	1.93E+37
Gm4705	0.802479316	0.727	0.217	2.34E-183
Lamtor5	0.799984019	0.426	0.061	6.14E-136
Supt4b	0.799114014	0.387	0.05	3.47E-175
Dad1	0.797319685	0.693	0.182	1.49E-200
Tmsb4x	0.795713697	1	0.997	1.13E-186
Eef1d	0.79083931	0.57	0.119	2.47E-183
Napsa	0.790535263	0.457	0.089	1.47E-58
Mien1	0.7867172	0.45	0.074	3.37E-144
Prdx2	0.783554786	0.49	0.092	1.17E-86
Eif3h	0.781278193	0.712	0.195	5.04E-191
Gm5621	0.779919233	0.58	0.125	4.81E-56
Lyz1	0.779851453	0.189	0.01	2.43E-174
Erh	0.779119554	0.618	0.143	2.13E-150
Taldo1	0.777817415	0.657	0.191	1.46E-105
Myl6	0.777388636	0.835	0.326	3.04E-95
Serf2	0.777031541	0.975	0.585	1.11E-151
Eif4ebp1	0.773291599	0.309	0.032	3.16E-137
Sri	0.766964716	0.524	0.101	1.91E-139
Ly6a	0.766704575	0.456	0.098	8.45E-199
Rpl17	0.765432141	0.966	0.721	1.21E-128
Cox6b1	0.759471247	0.73	0.232	6.12E-88
Ms4a4c	0.758639433	0.395	0.078	5.65E-128
Nkg7	0.757984068	0.608	0.219	1.94E-198
Coa3	0.757473915	0.356	0.042	1.07139E+98
Clta	0.754766714	0.768	0.263	3.25E-166
Supt4a	0.753956897	0.709	0.185	1.44E-196
Med28	0.75211843	0.44	0.069	4.98E+34
Card19	0.752078155	0.355	0.046	1.71E-88
Camp	0.748553993	0.6	0.094	3.99E+73
Nt5c	0.745035194	0.425	0.064	3.56E-188
Ccdc12	0.744349069	0.608	0.139	5.41E-187
Pgls	0.743027494	0.444	0.086	2.24E-99
I830127L07Ri	0.742892416	0.289	0.038	7.32E-199
Rpl12	0.740669697	0.253	0.018	2.05E-192
Tmem134	0.739518724	0.352	0.043	2.21E-147
Ptprcap	0.738126309	0.595	0.154	1.11E-86

LOC1008624:	0.736747907	0.984	0.779	1.76E-132
Pycard	0.735961104	0.62	0.17	2.72E-126
Cd68	0.735765978	0.317	0.037	3.45E-179
Atp5f1	0.734948882	0.648	0.177	4.18E-177
Rnasek	0.734486116	0.557	0.126	3.09E-156
Rac2	0.733379365	0.858	0.327	2.25E+62
Hp	0.733212185	0.812	0.324	2.92E-161
Dynlrb1	0.732870477	0.529	0.107	4.29E-194
Rps3	0.732756664	0.955	0.714	3E-114
Rpl18	0.730089114	0.92	0.6	4.23E-126
Fabp5	0.727819081	0.267	0.041	5.4278E-110
Rpl18a	0.726046342	0.597	0.154	2.09E+39
Spcs2	0.725369555	0.582	0.145	5.18E-104
Higd2a	0.724170322	0.454	0.079	9.35E-75
Sys1	0.720653774	0.394	0.059	4.87E-157
H2-Eb1	0.72023836	0.796	0.324	1.06E-49
Clic1	0.71974325	0.718	0.214	5.23E-54
Saa3	0.712653142	0.272	0.037	7.6E-146
Ostf1	0.710947767	0.661	0.173	2.1E-39
Timm23	0.708821047	0.367	0.053	1.05E-180
Nupr1	0.708755707	0.312	0.041	5.67E-173
Reep5	0.708344079	0.461	0.098	1.35E-198
Ndufs4	0.708280564	0.345	0.044	1.61E-130
H2afx	0.704629083	0.244	0.023	2.16E-161
Mgst1	0.702161937	0.443	0.108	1.11E-108
Cmtm7	0.701376315	0.528	0.121	1.58E-169
Polr1d	0.700234422	0.646	0.165	1.12E-195
Ypel3	0.696532361	0.509	0.102	1.12E-185
Bax	0.695137914	0.518	0.108	7.69E-193
Ufc1	0.692952765	0.323	0.037	1.06E-143
H2afz	0.687248317	0.924	0.472	2.05E-170
H2-D1	0.686377578	0.989	0.71	3.93E-199
Selk	0.686174981	0.81	0.288	4.12E-170
Tmem176b	0.683812532	0.287	0.046	4.7E-97
B2m	0.680359267	0.997	0.854	9.67E-199
H3f3c	0.679797345	0.29	0.034	3.43E-199
Swi5	0.6794624	0.484	0.097	1.89E-139
Dynll1	0.676097548	0.769	0.284	1.19E-170
Igfbp6	0.673235104	0.266	0.035	5.57E+86
Sap18b	0.670224846	0.52	0.115	1.12E-139
Rpl7	0.669522629	0.937	0.567	4.59E-188
Sra1	0.666665792	0.319	0.039	4.9E-197
Pold4	0.665551843	0.33	0.045	1.38E-36
Lrg1	0.663900208	0.466	0.123	0.00389
Banf1	0.660432366	0.381	0.064	2.48E+49
Rnase6	0.660232193	0.402	0.095	9.29E-186
Timm13	0.658102591	0.678	0.194	1.37E-183
Bloc1s2	0.656240585	0.376	0.06	3.66E-123
Tnfaip8l2	0.653936852	0.276	0.028	1.75E-187
Birc5	0.652016151	0.25	0.032	9.22E-197
Ninj1	0.651274163	0.413	0.081	6.57E-194
Psenen	0.649132961	0.526	0.114	8.18E-162
Dctn3	0.646989003	0.453	0.086	1.83E-124
Sec11c	0.646689002	0.545	0.151	1.16E-184
Pkig	0.644699663	0.375	0.065	9.78E-158
Gng5	0.641590603	0.282	0.033	5.98E-192
Ybx1	0.64080306	0.627	0.18	1.13E-46
2410015M20F	0.63966606	0.484	0.098	1.02E-101

Rpl5	0.639600071	0.671	0.227	6.44E-179
Tmem59	0.639547776	0.483	0.105	1.17E-173
Elof1	0.638264919	0.217	0.015	2.41E-97
S100a11	0.636503057	0.896	0.489	3.58E-71
Tppp3	0.635191311	0.219	0.028	8.27E-170
Lamtor4	0.63395406	0.533	0.135	8.73E-145
Ap2s1	0.63209304	0.464	0.095	3.62E-178
Eif3f	0.628524668	0.793	0.308	3.76E-186
Srp14	0.62595897	0.647	0.176	1.57E-77
Rpl10a	0.62368681	0.919	0.603	4.82E-113
Atp6v0e	0.623586307	0.721	0.246	8.4312E+98
Ssna1	0.623258707	0.309	0.04	1.14E+44
Psmb5	0.622071121	0.51	0.114	5.06E-170
Bmyc	0.622030304	0.216	0.018	1.1944E-120
Ranbp1	0.620690653	0.33	0.057	5.77E-93
Ccdc124	0.618740822	0.313	0.043	1.47E-192
Mrpl20	0.61851288	0.379	0.068	6.68E-25
Ube2s	0.617847021	0.63	0.178	3.41E-15
Ly86	0.614383743	0.353	0.08	8.03E-38
Mrpl12	0.613820448	0.303	0.04	1.92E-103
Capg	0.613690896	0.313	0.05	6.31E-172
Gm10045	0.613392877	0.896	0.589	1.27E-90
Rps13	0.613066396	0.891	0.57	7.35E-104
Atp5b	0.612917144	0.677	0.213	1.73E-105
Nutf2	0.610597232	0.3	0.042	1.6E-136
Eif6	0.60998353	0.41	0.076	3.14E-100
Nhp2	0.608268201	0.332	0.05	2.68E-50
Tceb2	0.606202428	0.586	0.15	9.51E-108
Atp6v0c	0.606083841	0.814	0.35	2.83E-163
Snx3	0.604747033	0.452	0.092	4.51E-154
Tagln2	0.604567028	0.735	0.271	4.09E-135
Ppdpf	0.604438309	0.365	0.063	3.08E-188
1110008F13R	0.604405963	0.479	0.101	1.43E-114
Phf5a	0.60324953	0.396	0.071	6.92E-181
Mrfap1	0.600296197	0.554	0.135	7.16E-170
Lsm4	0.599813214	0.564	0.139	9.71E+12
Iscu	0.599750235	0.317	0.047	5.1E+58
Stra13	0.599100792	0.312	0.044	1.98E-139
Cfl1	0.597995776	0.93	0.52	1.52E-160
Cdkn2d	0.597768341	0.431	0.091	1.48E-123
Mydgf	0.597186328	0.298	0.041	1.5E-142
Rpl26	0.595508304	0.914	0.649	2.59E-66
Anapc11	0.592885181	0.34	0.052	1.53E-190
Cox7a2l	0.592189994	0.804	0.303	5.15E-167
Mpc1	0.592003064	0.472	0.108	1.02E-176
Anxa5	0.590278844	0.439	0.103	8.17E-187
Cyb5a	0.590203031	0.286	0.04	1.86E-189
Ndufv2	0.588737689	0.217	0.017	3.78E+94
Scand1	0.58793264	0.724	0.244	2.93E-195
Hmgn2	0.587617588	0.5	0.126	1.53E-144
Sumo1	0.587161131	0.551	0.13	2.74E-181
Sptssa	0.583896492	0.413	0.081	5.56E-80
2700060E02R	0.583179634	0.534	0.132	5.56E-72
Hcfc1r1	0.582816807	0.246	0.027	1.71E-139
Rps19	0.578712406	0.946	0.79	28.1
Cd300a	0.578410981	0.367	0.072	1.78E-186
Mrps14	0.577941264	0.513	0.122	1.39E-198
Ngp	0.577407974	0.801	0.184	1.75E-18

Commd1	0.574861393	0.254	0.028	6.65E-150
Rnaset2b	0.574332108	0.489	0.124	2.76E-101
Rpl3	0.573987052	0.182	0.01	1.49E-183
Hprt	0.573604464	0.374	0.069	3.74E-136
Sod2	0.573059815	0.377	0.08	2.18E-81
Ndufs8	0.572652126	0.318	0.049	3.58E-108
Rpl8	0.568917265	0.972	0.747	1.89E-89
Snrpd2	0.568177684	0.508	0.125	1.65E-153
Ndufa12	0.567185608	0.322	0.048	1.38E-82
Tmsb10	0.566615689	0.997	0.914	1.81E-60
Tmed9	0.566561014	0.51	0.125	3.67E-148
Rabac1	0.56566834	0.606	0.158	2.04E-200
Rgcc	0.565044439	0.369	0.079	357000000
Cks2	0.564695972	0.498	0.13	3.71E-114
Ran	0.564445591	0.641	0.201	1.63E-11
Rpl13a	0.563926565	0.586	0.156	4.15E-190
Rnh1	0.56167599	0.307	0.049	4.29E-64
Psmd8	0.557459735	0.4	0.075	2.13E+38
Mrps16	0.557060301	0.287	0.041	1.37E-108
Mdh2	0.555499793	0.452	0.096	9.8E-200
Nop10	0.554764211	0.653	0.209	4.59E-171
Gnas	0.547687683	0.648	0.218	5.71E-168
Creg1	0.547665595	0.393	0.085	1.6E-120
Cd79a	0.547415458	0.343	0.104	2.6E-176
Yif1b	0.547227308	0.184	0.013	1.21E-174
Spint2	0.547154607	0.271	0.038	1.22E-192
Tmed3	0.546748969	0.356	0.069	9.78E-36
Atraid	0.545917407	0.246	0.029	1.36E-157
Emc6	0.545769007	0.382	0.073	2.31E-186
Gemin7	0.545734899	0.253	0.031	6.28E-96
Mpo	0.542188177	0.134	0.009	4.62E-97
Gadd45g	0.540911492	0.32	0.062	2.3E-180
Ier2	0.539832796	0.801	0.316	1.5E-136
Hsd17b10	0.539201979	0.237	0.027	3.5E-163
Lsm3	0.538800058	0.296	0.047	9.18E-196
Ms4a6d	0.538418994	0.222	0.027	5.93E-205
Pdia3	0.537132089	0.566	0.153	7.08E-90
Mrps34	0.53700502	0.243	0.027	5.32E-169
Rps16	0.536641517	0.987	0.845	1.37E-52
Dynlt1f	0.535939575	0.229	0.026	4.5567E-130
Chchd2	0.535703763	0.852	0.381	4.27E-75
Khk	0.534360323	0.257	0.04	4.9E-124
Cd3d	0.533385728	0.373	0.115	4.1956E-141
Vdac3	0.533378676	0.278	0.039	1.8E-153
2700094K13F	0.53306181	0.281	0.048	4.65E-36
Bri3	0.532135257	0.531	0.143	5.71E-154
Atp6v0b	0.531664365	0.616	0.191	2.55E-187
Rpl10	0.530475999	0.974	0.771	2.75E-72
Timm17b	0.530193541	0.217	0.024	3.27E-206
Mrpl54	0.529611008	0.395	0.081	1.06E-148
Pfdn1	0.527777477	0.245	0.031	9.98E+12
Pomp	0.527686884	0.665	0.226	1.1E-106
Scp2	0.527510854	0.54	0.144	3.32E-187
Smim14	0.526624863	0.493	0.126	2.44E-191
Slc25a4	0.526493193	0.276	0.045	2.35E-114
Ifitm6	0.526030283	0.404	0.104	6.19E-57
Map1lc3b	0.525496135	0.747	0.259	4.53E-169
Rpl4	0.525178451	0.951	0.627	1.33E-146

Mrps12	0.524724634	0.274	0.041	1.78E-104
Ctsl	0.524294007	0.371	0.102	2.75E-133
BC031181	0.522601729	0.443	0.093	3.31E-170
Rheb	0.522194939	0.388	0.079	2.08E-158
Ost4	0.521641643	0.54	0.148	44400
Cxcl2	0.52134958	0.897	0.455	2.76E-123
Mrpl14	0.521224499	0.355	0.066	6.83E-124
Mrpl18	0.521058937	0.298	0.049	2.51E-195
Krtcap2	0.520897708	0.461	0.122	1.09E-63
Txn2	0.520650864	0.314	0.05	1.54E-181
Snrpb	0.520479971	0.613	0.183	6.64E-87
Pebp1	0.520365573	0.373	0.075	7.98E-172
Atp5j	0.520080364	0.27	0.037	1.01E-176
Dnajc15	0.519991203	0.38	0.076	9.55E-87
Sdhb	0.518887779	0.294	0.044	3.68E-144
Bag1	0.517171528	0.314	0.054	4.18E-156
Aurkaip1	0.51685939	0.337	0.061	6.94E-194
Cox5b	0.514094854	0.634	0.207	3.08E-193
Klk8	0.514021014	0.176	0.024	1.17E-126
Vps29	0.511919019	0.286	0.044	3.19E-174
Limd2	0.510092635	0.566	0.152	1.34E-82
Pglyrp1	0.509764011	0.541	0.174	1.13E-125
S100a1	0.509339366	0.167	0.012	2.53E-145
Rps24	0.509327312	0.922	0.725	9.16E-07
Timm10b	0.508174863	0.538	0.14	6.49E-120
1110008P14R	0.506571848	0.287	0.044	4.29E-181
Gm21188	0.506327182	0.231	0.047	6.46E-148
Psma1	0.506273982	0.388	0.08	6.17E-189
Clec4a3	0.505399797	0.284	0.054	1.13E-110
Atp5g1	0.504350349	0.52	0.147	6.31E-135
Cox8a	0.503410047	0.902	0.456	2.25E-87
Rac1	0.502315707	0.685	0.229	6.78E-176
Psma3	0.500708573	0.277	0.041	1.16E-142
Ms4a6c	0.50031626	0.504	0.171	3.37E-160
Ibtk	-0.500276289	0.103	0.079	3.85E-24
Mctp2	-0.501104906	0.107	0.077	5.14E-52
Xrn2	-0.501293575	0.138	0.092	5.56E-77
Setd1b	-0.50182615	0.118	0.08	5.18E-64
Pabpc1	-0.50226396	0.721	0.455	8.43E-162
Plekho2	-0.5031882	0.214	0.127	4.96E-200
Creb1	-0.504188257	0.145	0.096	1.81E-78
Canx	-0.50434635	0.487	0.29	2.38E-105
Isy1	-0.505299502	0.172	0.105	3.47E-118
Arfgef1	-0.505391251	0.122	0.082	1.9E-69
Pcna	-0.505680463	0.286	0.159	1.41E-60
Slc38a1	-0.505872449	0.156	0.1	7.78E-109
Xiap	-0.507421391	0.145	0.101	3.65E-84
Psap	-0.508140909	0.944	0.732	1.46E-166
Rtn4	-0.508144391	0.308	0.175	3.2E-152
Mfsd14b	-0.508155034	0.149	0.097	1.24E-86
Notch1	-0.508902125	0.157	0.103	3.61E-101
Ube2b	-0.509299038	0.649	0.388	1.7E-164
Sppl2a	-0.509394358	0.11	0.079	2.93E-64
Ccr5	-0.509495983	0.142	0.09	1.49E-71
Bach1	-0.509516309	0.147	0.099	1.11E-112
Elf2	-0.51005889	0.121	0.086	2.22E-59
Shisa5	-0.510174319	0.769	0.497	1.4E-174
Irf1	-0.510705495	0.36	0.203	1.04E-26

Noc2l	-0.510823192	0.115	0.079	2.28E-68
Zmiz1	-0.511656426	0.16	0.104	3.54E-117
Spib	-0.511872545	0.154	0.098	2.04E-80
Numa1	-0.511904246	0.14	0.091	5.31E-87
Atxn7	-0.512591649	0.1	0.075	2.18E-34
Jak2	-0.512932141	0.143	0.093	1.01E-99
Ptprs	-0.513047986	0.128	0.089	2.96E-89
Hnrnpr	-0.513216945	0.187	0.112	3.38E-154
Irf2	-0.513226994	0.188	0.116	1.17E-148
Sgk1	-0.51328927	0.183	0.108	4.02E-129
Atg2a	-0.51342012	0.124	0.086	1.03E-71
Otulin	-0.514380246	0.198	0.126	5.85E-138
Bod1l	-0.514540555	0.108	0.083	9.27E-33
Tm9sf3	-0.515439773	0.278	0.16	6.52E-183
Irak2	-0.515583013	0.128	0.087	9.78E-77
Mapk14	-0.515875879	0.158	0.101	3.6E-104
Adgre5	-0.515897776	0.489	0.282	2.32E-165
Emilin2	-0.516338013	0.348	0.19	6.76E-140
Tmc6	-0.516828166	0.124	0.086	4.47E-71
Adam19	-0.517218675	0.15	0.103	2.28E-83
Csnk1d	-0.517652198	0.184	0.112	1.43E-141
Ccr7	-0.517691132	0.373	0.224	2.64E-199
Ppp4r2	-0.518808012	0.193	0.122	2.38E-138
Picalm	-0.518815163	0.487	0.276	1.94E-93
Fnip1	-0.519726471	0.11	0.082	2.24E-50
Prdm2	-0.520176183	0.121	0.087	1.1E-67
Rps6ka3	-0.520695503	0.105	0.08	1.35E-34
Nampt	-0.522131946	0.177	0.105	2.3E-153
Klhl9	-0.522981905	0.121	0.084	7.12E-90
Lef1	-0.523756743	0.133	0.092	3.14E-49
Dclre1c	-0.523933928	0.143	0.098	4.9E-86
Kif5b	-0.524176118	0.254	0.152	4.61E-171
Rbm5	-0.524243119	0.161	0.103	6.25E-123
Safb2	-0.524823748	0.154	0.102	2.45E-101
Dennd5a	-0.525422302	0.178	0.121	8.19E-122
Sidt2	-0.525583877	0.174	0.107	9.39E-138
Smc6	-0.525853674	0.147	0.104	1.11E-74
Samd9l	-0.526132374	0.15	0.102	1.77E-74
Hsp90b1	-0.526185095	0.637	0.408	1.08E-68
Nfe2l2	-0.526290253	0.394	0.237	9.55E-139
Fam107b	-0.526595084	0.467	0.27	2.29E-159
Smek2	-0.526768869	0.166	0.107	7.27E-123
Zc3h7a	-0.526931404	0.153	0.1	2.62E-112
Tcf25	-0.527458132	0.3	0.172	2.14E-85
Cxcr2	-0.52803209	0.195	0.12	7.53E-105
Mef2d	-0.52823004	0.194	0.119	1.3E-156
Tes	-0.529392012	0.152	0.101	8.79E-85
Cdk11b	-0.530108582	0.12	0.087	4.49E-59
Smg7	-0.530146209	0.106	0.079	5.33E-34
Lasp1	-0.5307729	0.17	0.112	2.52E-116
Arhgap17	-0.533612766	0.146	0.102	7.3E-103
Sgms1	-0.534300364	0.125	0.092	9.33E-57
Hsp90aa1	-0.53462352	0.716	0.473	1.78E-73
Arhgef2	-0.534723119	0.113	0.086	1.01E-40
Tgfbr2	-0.535322047	0.164	0.107	5.64E-123
H2-Q4	-0.535326134	0.308	0.18	4.03E+42
Cpsf6	-0.535951453	0.159	0.106	3.96E-103
Clk4	-0.536027527	0.193	0.119	3.21E-162

Lyst	-0.536838267	0.117	0.088	6.5E-46
Nucks1	-0.537253208	0.24	0.15	1.66E-192
Baz1a	-0.538224556	0.184	0.124	1.52E-119
Pak2	-0.540335195	0.294	0.17	3.84E-133
Coq10b	-0.540751343	0.334	0.192	3.16E-175
Ppp2r5c	-0.541527643	0.214	0.133	1.31E-178
Mat2a	-0.541974926	0.239	0.144	7.13929E-59
Hnrnpu	-0.543121845	0.487	0.294	1.7E-158
Fmnl1	-0.543338609	0.317	0.188	1.73E-158
Cggbp1	-0.543429978	0.237	0.145	5.55E-192
Zc3h11a	-0.544080616	0.161	0.108	1.2E-114
Mafb	-0.544109043	0.186	0.094	4.96E-124
Klrc1	-0.546137631	0.146	0.105	5.57E-49
Phf3	-0.547203142	0.144	0.103	2.04E-74
Dcaf12	-0.548089779	0.115	0.082	5.09E-76
Serinc3	-0.548134122	0.666	0.435	1.89E-161
Fli1	-0.548217113	0.167	0.107	5.36E-123
Fbxo33	-0.54851608	0.109	0.088	5.86E-27
Nfkbid	-0.548604358	0.301	0.185	9.63E-42
Gnai3	-0.548670038	0.188	0.118	9.43E-150
Chka	-0.54880848	0.154	0.101	2.56E-90
Srsf11	-0.549257721	0.198	0.125	3.56E-150
Jmjd1c	-0.549505314	0.186	0.123	2.56E-127
Nxf1	-0.549980818	0.168	0.115	7.59E-121
Rpl41	-0.550122354	1	0.996	1.47E-42
Ptafr	-0.550421374	0.269	0.172	2.1E-196
Prrc2a	-0.551152498	0.135	0.096	8.15E-74
Rsbn1l	-0.55193302	0.211	0.132	7.98E-170
Pabpn1	-0.552109326	0.254	0.152	1.54E-126
Arid4b	-0.552309286	0.119	0.089	4.42E-44
Pdcd6ip	-0.552354304	0.234	0.143	4.01E-203
Mapk6	-0.552409367	0.124	0.086	3.93E-84
Eef2	-0.552928101	0.872	0.679	3.25E-100
Camk2d	-0.553220775	0.127	0.09	3.52E-75
Pbxip1	-0.553253957	0.202	0.124	1.34E-168
Ptp4a2	-0.554943169	0.471	0.275	6.88E-94
Evl	-0.555453749	0.239	0.144	2.0829E-155
Scaf11	-0.555835651	0.153	0.105	2.7E-101
Dnajc5	-0.556073574	0.207	0.13	6.48E-170
Hnrnpk	-0.556279798	0.613	0.391	5.04E-188
Nup153	-0.556831289	0.1	0.077	1.01E-56
Syk	-0.557032076	0.323	0.193	2.98E-191
Kras	-0.558243858	0.24	0.15	1.11E-204
Hnrnpa3	-0.559052243	0.565	0.347	1.12E-105
Cnbd2	-0.559305084	0.176	0.114	2.61E-137
Ss18	-0.559326741	0.118	0.087	2.93E-56
Tiparp	-0.559809771	0.196	0.122	4.57E-160
Rnf44	-0.560761647	0.135	0.098	1.2E-84
Ap2a2	-0.561003712	0.13	0.098	2.67E-80
Zbtb7a	-0.561045803	0.172	0.114	1.43E-135
Rplp1	-0.561348497	0.986	0.942	6.19E-49
Mapk1ip1l	-0.561834254	0.125	0.092	2.3E-72
Top2b	-0.56215052	0.183	0.119	4.59E-138
Kdm6b	-0.564526452	0.31	0.195	2.56E-159
Herpud1	-0.564699904	0.428	0.262	4.21E-114
Asxl2	-0.565861622	0.135	0.099	3.75E-67
Arf3	-0.566741728	0.209	0.13	1.48E-195
Scd1	-0.567414446	0.1	0.079	86.2

Crlf3	-0.56884062	0.258	0.156	3.97E-166
Glcci1	-0.56941152	0.119	0.094	3.25E-41
Wac	-0.570110092	0.201	0.132	1.29E-153
Slc44a2	-0.571200908	0.26	0.161	3.95E-191
Chd3	-0.572474053	0.175	0.119	4.94E-110
Ncor1	-0.57344446	0.243	0.15	1.46E-154
Laptm5	-0.573520552	0.801	0.56	1.36E-107
Irf2bpl	-0.573815045	0.105	0.085	5.32E-40
Atf7ip	-0.574363859	0.196	0.123	2.97E-177
Tle3	-0.574600111	0.245	0.152	4E-85
Ivns1abp	-0.576171631	0.282	0.172	4.32E-195
Arhgap30	-0.577189199	0.305	0.186	1.62E-51
Aff1	-0.578445898	0.105	0.089	3.62E-34
Actr2	-0.579124512	0.418	0.247	0.000158
Zfp292	-0.579353566	0.149	0.107	3.82E-99
Cnot1	-0.580659527	0.116	0.091	2.14E-56
Mfsd14a	-0.582032548	0.102	0.088	1.93E-33
Gnb1	-0.582894827	0.4	0.237	8.1E-24
Med13	-0.583720878	0.102	0.086	6.01E-35
Sorl1	-0.58557871	0.319	0.196	2.98E-81
Malt1	-0.585734348	0.184	0.123	8.27E-133
Adnp	-0.586608133	0.128	0.099	2.08E-66
Icam1	-0.586975475	0.198	0.127	1.95E-145
Nisch	-0.587211265	0.199	0.129	4E-161
Tatdn2	-0.587385104	0.118	0.092	1.28E-54
G3bp2	-0.587857036	0.224	0.139	1.51E-197
Gpatch8	-0.587880326	0.1	0.089	1.5E-21
Akap13	-0.588558469	0.363	0.211	4.51E-153
Pik3cd	-0.58891342	0.275	0.169	1.93E-138
Usp25	-0.590885935	0.172	0.119	7.18E-136
Cnot6	-0.591421944	0.148	0.104	2.84E-94
Adrbk1	-0.592341827	0.434	0.259	2.79E-94
Mbd2	-0.592449384	0.21	0.141	5.69E-163
Lilr4b	-0.593244334	0.263	0.162	1.5E-203
Cyth1	-0.593596216	0.202	0.131	1.27E-172
Ptp4a1	-0.594699111	0.42	0.263	4.82E-114
Fryl	-0.596265833	0.141	0.109	3.54E-67
H2-K1	-0.596368224	0.943	0.811	1.18E-165
Rpl37a	-0.596427804	0.669	0.443	3E-12
Ptk2b	-0.596775301	0.264	0.16	2.74E-96
Wdr89	-0.596812141	0.997	0.966	2.83E-65
Uqcrh	-0.597381184	0.881	0.695	3.81E-199
Tor1aip1	-0.597421089	0.221	0.148	6.24E-184
Ino80d	-0.597738775	0.117	0.094	4.56E-57
Spata13	-0.598440591	0.172	0.117	1.26E-136
Ddx18	-0.598678886	0.126	0.099	1.15E-60
Mepce	-0.600720489	0.146	0.107	3.45E-96
Ubl3	-0.600942346	0.38	0.219	2.57E-169
Rassf3	-0.60129477	0.167	0.12	1.18E-112
Pias1	-0.603717409	0.105	0.088	3.76E-45
Whsc1l1	-0.604103256	0.27	0.164	5.1E-134
Trp53inp1	-0.604406909	0.148	0.102	2.32E-100
Tmem30a	-0.604561459	0.234	0.15	7.42E-190
Leng8	-0.60481371	0.147	0.109	4.99E-90
Kctd12	-0.604817564	0.412	0.251	1.66E-143
Gm21811	-0.605293094	0.187	0.123	1.83E-154
Eif2s3x	-0.605668216	0.147	0.112	1.46E-87
Pitpnc1	-0.606000598	0.153	0.108	1.26E-102

Il7r	-0.606326504	0.304	0.191	2.25E-144
Rad21	-0.607632902	0.221	0.148	4.65E-172
Far1	-0.607703659	0.145	0.108	2.73E-105
Cd274	-0.608638404	0.106	0.085	3.22E-31
Atp2a3	-0.610174109	0.155	0.115	7.55E-96
Mmp9	-0.610301623	0.233	0.146	2.62E-144
Kmt2c	-0.610479016	0.105	0.087	4.02E-50
Arid4a	-0.61145936	0.132	0.104	2.04E-68
Nfkb1	-0.611689909	0.257	0.16	2.64E-180
Smek1	-0.611748104	0.112	0.094	7.19E-52
Ccng2	-0.612431396	0.158	0.117	1.08E-97
Tcf7	-0.612960766	0.202	0.125	2.64E-172
Cdkn1b	-0.613003854	0.184	0.132	2.8E-128
Tpr	-0.613006664	0.161	0.115	3.01E-109
2810474O19F	-0.61321181	0.248	0.156	1.43E-154
Kpna4	-0.613313315	0.205	0.133	2.64E-180
Tnfrsf1b	-0.61352653	0.307	0.194	1.9E-74
Ttc14	-0.614124833	0.154	0.11	5.28E-117
Usp19	-0.615269047	0.127	0.102	2.5E-78
Bcl11b	-0.616411093	0.106	0.089	7.19E-23
Itk	-0.617503375	0.163	0.117	1.88E-103
Abcf1	-0.617820949	0.162	0.121	8.73E-103
Smg1	-0.618038998	0.136	0.102	4.75E-94
Cpeb2	-0.618262704	0.1	0.085	1.28E-32
Stat3	-0.618964793	0.375	0.23	2.46E-145
Nfatc1	-0.619490899	0.164	0.117	1.18E-110
Ppp1r15a	-0.620348528	0.588	0.368	1.68E-162
Slfn5	-0.621324185	0.151	0.106	6.27E-101
Gem	-0.621691528	0.103	0.086	1.09E-07
Birc2	-0.621812598	0.132	0.108	3.46E-55
Wnk1	-0.623021807	0.288	0.181	8.11E-60
Baz1b	-0.623770545	0.14	0.106	4.37E-88
Atp11b	-0.62470822	0.116	0.091	1.26E-75
Trip12	-0.624928295	0.205	0.139	5.35E-167
Ctnnb1	-0.625145799	0.188	0.134	6.98E-158
Trim12c	-0.625928778	0.138	0.106	1.53E-80
Fam134b	-0.627073854	0.187	0.122	3.24E-156
Epb41	-0.629025666	0.102	0.088	5.3E-38
Gna13	-0.630529689	0.319	0.2	1.7E-158
Dync1h1	-0.631246708	0.145	0.109	7.31E-107
Cox17	-0.632933566	0.61	0.395	1.1E-192
Nup98	-0.63375517	0.113	0.094	2.16E-58
Lrrfip1	-0.634503936	0.311	0.194	3.13E-147
Cebpz	-0.635037741	0.197	0.135	1.05E-168
Nktr	-0.635533348	0.122	0.102	7E-65
Nab1	-0.63652589	0.167	0.122	1.06E-128
Myo9b	-0.636884802	0.188	0.132	1.13E-155
Icos	-0.636886119	0.114	0.089	3.05E-32
Arl8a	-0.637490652	0.127	0.102	6.01E-84
Bcl11a	-0.638484474	0.149	0.111	1.98E-70
Il17ra	-0.638616818	0.327	0.204	1.57E+31
Dck	-0.638980911	0.174	0.12	5.39E-126
Gda	-0.640496338	0.429	0.258	6.44E-182
Rock1	-0.641013562	0.177	0.127	5.93E-139
Kmt2e	-0.641206013	0.275	0.178	3.78E-118
Aplp2	-0.641686197	0.187	0.134	2.02E-148
Spty2d1	-0.642880998	0.14	0.111	1.64E-81
Pcbp1	-0.643453232	0.51	0.318	2.7E+36

Kmt2a	-0.643456124	0.117	0.1	2.21E-53
Klf4	-0.644051918	0.231	0.147	1.32E-184
Arid5a	-0.644335777	0.314	0.201	4.13E-168
Phip	-0.646172285	0.1	0.091	8.42E-36
Slk	-0.64629809	0.155	0.121	3.04E-114
Elovl5	-0.646555781	0.199	0.135	3.68E-174
Rgs2	-0.647645844	0.579	0.378	2.63E-102
Tbl1xr1	-0.648612289	0.17	0.128	5.42E-130
Pnn	-0.648762409	0.152	0.117	2.29E-99
Dhx9	-0.650293597	0.125	0.102	3.64E-80
Cab39	-0.651927222	0.207	0.146	8.34E-165
Elmsan1	-0.652196083	0.124	0.103	4.64E-60
Mycbp2	-0.652315681	0.266	0.179	2.82E-60
Pde4b	-0.654344011	0.342	0.217	4.15E-151
Gimap3	-0.654678538	0.308	0.202	3.21E-171
Birc3	-0.655074149	0.234	0.158	1.41E-207
Larp4b	-0.655178416	0.269	0.178	6.73E-194
Prex1	-0.655730358	0.184	0.131	4.88E-146
Klf10	-0.657036318	0.243	0.164	7.21E-183
U2af2	-0.657254134	0.233	0.155	8.31E-159
Gpbp1	-0.66062357	0.28	0.183	1.9E-103
Hnrnpul2	-0.661593577	0.27	0.179	3.24E-174
Sod1	-0.663065863	0.193	0.139	1.05E-168
Lmo4	-0.663094813	0.206	0.14	2.43E-166
Aff4	-0.663504838	0.118	0.099	1.37E-67
Serinc1	-0.663980703	0.228	0.15	2.71E-146
Wdr43	-0.664230077	0.101	0.098	5.16E-36
Wipf1	-0.66460818	0.291	0.191	2.28E-135
Smad4	-0.664836507	0.17	0.125	6.58E-139
Arid1a	-0.664950283	0.237	0.157	2.5826E-152
Dgat1	-0.665730075	0.351	0.242	5.41E-153
Il21r	-0.666021484	0.226	0.16	5.89E-164
Ube2h	-0.666141811	0.203	0.149	2.97E-148
Pura	-0.66751568	0.136	0.107	1.24E-87
Ppp1r12a	-0.668812348	0.2	0.142	4.5E-154
N4bp1	-0.669108502	0.143	0.107	8.28E-98
Tob1	-0.672362418	0.175	0.129	8.23E-136
Lmnb1	-0.67267865	0.423	0.291	2.44E-152
Arhgef3	-0.673305774	0.124	0.104	2.31E-62
Zeb2	-0.673808684	0.224	0.149	1.1949E-152
Cyld	-0.67421615	0.127	0.109	4.89E-74
Pbrm1	-0.674528848	0.191	0.141	1.08E-150
Zcchc6	-0.675926962	0.148	0.121	1.08E-100
Ywhaz	-0.67629928	0.54	0.352	1.28E-137
Rbm25	-0.677417018	0.253	0.168	1.23E-95
Neurl3	-0.67827411	0.247	0.174	1.07E-187
Hk2	-0.678687935	0.1	0.09	9.86E-23
Serpinb6b	-0.678855178	0.161	0.119	4.85E-63
Azin1	-0.679291323	0.221	0.153	1.63E-206
Cd300lf	-0.679645093	0.234	0.167	8.45E-162
Csnk1g3	-0.681409287	0.107	0.098	1.67E-41
Rrbp1	-0.681955616	0.424	0.271	4.09E-106
Gm1966	-0.682437984	0.154	0.119	2.94E-113
Fbxl5	-0.683421264	0.247	0.163	2.72E-183
Rell1	-0.683492229	0.186	0.136	8.36E-150
Hivep2	-0.684442527	0.136	0.114	1.07E-70
Pcmtd1	-0.685017368	0.113	0.1	1.68E-52
Tgif1	-0.685775995	0.33	0.21	1.64E+17

Ier5	-0.686445659	0.713	0.486	1.45E+21
Cytip	-0.688793289	0.707	0.504	8.81E-29
Zfp36	-0.690867469	0.766	0.538	4.76E-182
Skil	-0.692831648	0.223	0.162	9.87E-195
Mapk1	-0.694507946	0.268	0.185	3.86E-149
Il18rap	-0.695194038	0.114	0.103	3.98E-42
Slc6a6	-0.695510644	0.197	0.146	1.76E-173
Smchd1	-0.696077351	0.157	0.127	1.89E-98
Safb	-0.69617807	0.168	0.127	1.41E-133
Jun	-0.696745736	0.686	0.473	1.34E-145
Cd164	-0.69975238	0.346	0.227	6.02E-183
Per1	-0.701549208	0.251	0.177	3.75E-184
Fbxl3	-0.70248396	0.119	0.103	4.01E-71
G3bp1	-0.702616153	0.296	0.194	1.48E-182
Hp1bp3	-0.703416056	0.204	0.144	9.18E-185
Sf3b1	-0.704346443	0.447	0.296	5.42E+43
Slc15a3	-0.705275624	0.208	0.148	4.8E-170
Phf21a	-0.706347107	0.151	0.118	9E-124
Zcchc7	-0.706461937	0.127	0.114	1.83E-75
Spag9	-0.706474136	0.161	0.13	7.19E-129
Klhl24	-0.70650268	0.197	0.153	3.43E-153
Ankrd44	-0.706801708	0.173	0.135	1.22E-124
Nfatc3	-0.707207447	0.14	0.113	7.99E-89
Ythdf3	-0.707948973	0.152	0.123	5.6E-113
Zbtb20	-0.70917512	0.173	0.131	1.92E-115
Ppig	-0.710760825	0.251	0.173	3.99E-143
Trim25	-0.711679266	0.282	0.196	5.04E-21
Stk4	-0.713781028	0.227	0.16	3.29E-188
Sfpq	-0.71383892	0.336	0.221	1.45E-65
Tkt	-0.714929877	0.399	0.266	8.46E-173
Sf1	-0.715251444	0.317	0.216	4.72E-132
Ppp1r16b	-0.715590753	0.129	0.116	5.24E-67
Jarid2	-0.716056303	0.152	0.124	1.1E-120
Ezr	-0.716279951	0.366	0.234	2.1E-71
Prkcb	-0.716691348	0.305	0.201	1.64E-134
Pafah1b1	-0.717425625	0.286	0.195	8.18E-115
Sh2d2a	-0.718770033	0.173	0.137	7.56E-96
Runx1	-0.718789444	0.19	0.144	7.03E-154
Dazap2	-0.719547264	0.595	0.383	1.1E-193
Eif2s2	-0.721186016	0.532	0.36	2.61E-51
Macf1	-0.724643774	0.259	0.184	0.0097
Fnbp1	-0.725757735	0.187	0.138	6.68E-174
Tardbp	-0.726177788	0.196	0.15	2.87E-160
Kdm5a	-0.729278404	0.134	0.12	3.8E-72
Rtf1	-0.730848339	0.245	0.176	3.71E-51
Msl2	-0.731933141	0.159	0.127	6.09E-126
Odc1	-0.732874068	0.267	0.177	1.44E-83
Pum1	-0.732965291	0.135	0.121	3.68E-81
Map4k4	-0.734569941	0.17	0.135	1.83E-139
Hdc	-0.734956529	0.455	0.326	1.93E+37
Gatad2b	-0.736182607	0.141	0.122	1.03E-97
Nipbl	-0.739241492	0.197	0.154	2.12E-162
Zdhhc18	-0.740549154	0.145	0.122	8.66E-93
S1pr1	-0.741892392	0.173	0.124	1.98E-124
Pdpk1	-0.742111598	0.102	0.101	1.72E-46
Elavl1	-0.742958301	0.26	0.19	2.34E-183
Hif1a	-0.743154581	0.274	0.192	6.14E-136
Mxd1	-0.744612715	0.522	0.349	3.47E-175

Rbms1	-0.745550718	0.308	0.211	1.49E-200
Abi1	-0.745619577	0.265	0.183	1.13E-186
Pik3r1	-0.750055793	0.191	0.145	2.52E-134
Etnk1	-0.750414174	0.155	0.127	2.36E-137
Grina	-0.751238512	0.492	0.33	2.47E-183
Ppp6r1	-0.751549248	0.15	0.128	1.56E-109
Trib1	-0.752482821	0.291	0.208	1.47E-58
Eif5b	-0.753898892	0.29	0.206	3.37E-144
Suco	-0.754115618	0.107	0.107	1.34E-45
Chd7	-0.754747842	0.202	0.152	1.69E-173
Sp3	-0.7554368	0.134	0.12	4.33E-96
Fyn	-0.761175304	0.249	0.187	1.7724E-151
Tnrc6a	-0.761639873	0.133	0.127	1.58E-74
Slfn4	-0.762284989	0.148	0.121	1.09E-70
Ssh2	-0.762718925	0.375	0.253	1.17E-86
Tab2	-0.763629212	0.194	0.152	2.54E-164
Kat6a	-0.764046365	0.105	0.105	3.01E-70
Vps4b	-0.76632379	0.188	0.148	1.52E-169
Ddx3x	-0.766491844	0.364	0.256	5.04E-191
Tagap	-0.767196176	0.18	0.138	1.27E-135
Cdk17	-0.772366329	0.109	0.106	8.03E-57
Ets2	-0.773174566	0.338	0.234	4.81E-56
Sp110	-0.774405664	0.245	0.177	2.13E-150
Cd28	-0.774993266	0.156	0.126	4.49E-107
Wdr26	-0.779559055	0.268	0.199	1.46E-105
Rsrc2	-0.780725429	0.325	0.229	3.04E-95
Ewsr1	-0.781893018	0.329	0.227	1.11E-151
D16Ertd472e	-0.782238045	0.233	0.171	1.64E-195
Phf20l1	-0.783235171	0.162	0.136	1.56E-131
Ccnd2	-0.788502441	0.253	0.188	1.13E-182
Nr3c1	-0.790555148	0.237	0.172	3.75E-207
Iqgap1	-0.793350152	0.466	0.327	3.16E-137
St3gal4	-0.793392912	0.34	0.224	1.91E-139
Nufip2	-0.793740055	0.215	0.167	8.34E-204
Ccdc88c	-0.794105237	0.167	0.139	2.39E-142
Rsrp1	-0.794444078	0.488	0.33	8.45E-199
Zc3hav1	-0.796432529	0.33	0.228	6.12E-88
Dusp10	-0.801147462	0.139	0.119	5.08E-80
Hsph1	-0.801495427	0.321	0.214	5.65E-128
Myh9	-0.802598707	0.291	0.21	1.94E-198
Pde7a	-0.807694364	0.162	0.138	1.41E-124
Pmaip1	-0.808495917	0.273	0.194	1.07139E+98
Ikzf1	-0.809784577	0.377	0.26	3.25E-166
Gpcpd1	-0.810053794	0.362	0.254	1.44E-196
Aebp2	-0.811463563	0.125	0.124	5.39E-65
Cmah	-0.813043127	0.155	0.141	5.52E-111
Ankrd12	-0.813270709	0.124	0.122	1.28E-62
Taok1	-0.813425626	0.119	0.114	4.22E-94
Rpl28	-0.816364704	0.1	0.11	1.02E-57
Fam65b	-0.816864755	0.227	0.182	2.69E-191
Srsf6	-0.817601756	0.345	0.241	4.98E+34
Hnrnpdl	-0.818393779	0.374	0.261	1.71E-88
Ncf1	-0.819178843	0.411	0.282	3.99E+73
Ddit3	-0.821225712	0.391	0.27	3.56E-188
Matr3	-0.822215599	0.219	0.175	6.2E-196
Clint1	-0.822962447	0.218	0.172	8.9466E-150
Dhx15	-0.823655676	0.19	0.159	1.5E-162
Son	-0.825493734	0.502	0.353	5.41E-187

Ugcg	-0.825526031	0.218	0.162	1.41E-195
Srrm2	-0.825583708	0.451	0.322	2.24E-99
Arih1	-0.82604165	0.213	0.17	1.14E-196
4932438A13F	-0.828022455	0.126	0.126	3.22E-74
Stk38	-0.829471978	0.207	0.171	1.83E-185
Fos	-0.833388577	0.894	0.729	7.32E-199
Hipk1	-0.834173484	0.259	0.2	2.05E-192
Pum2	-0.834182927	0.271	0.204	2.21E-147
St8sia4	-0.838147167	0.293	0.22	1.11E-86
Dock10	-0.838432724	0.224	0.183	8.316E-149
Mef2c	-0.84302061	0.285	0.215	4.49E-197
Eif4g2	-0.843696154	0.531	0.381	2.72E-126
Serpinb9	-0.844296395	0.165	0.136	3.34E-85
Susd6	-0.845508457	0.147	0.145	3.38E-104
Impact	-0.846712512	0.115	0.119	2.16E-76
Ist1	-0.848093572	0.264	0.2	3.45E-179
Samsn1	-0.848822317	0.368	0.268	4.18E-177
Ep300	-0.850827154	0.111	0.122	2.7E-65
Elf1	-0.851031163	0.337	0.246	3.09E-156
Csf3r	-0.851989482	0.4	0.285	2.25E+62
Tle4	-0.852032526	0.128	0.128	7.44E-81
Clec2d	-0.85499964	0.169	0.149	1E-144
Strn3	-0.857927223	0.127	0.132	1.53E-83
Tgoln1	-0.858398326	0.293	0.224	2.92E-161
Csnk1a1	-0.858410673	0.357	0.261	4.29E-194
Rel	-0.861323064	0.105	0.122	1.33E-43
Ifrd1	-0.862552266	0.49	0.366	2.09E+39
Rnf125	-0.863376998	0.259	0.2	2.447E-148
Atrx	-0.866917981	0.201	0.169	1.3E-190
Ip6k1	-0.868704146	0.113	0.123	5.25E-70
Mgea5	-0.872089559	0.18	0.16	3.2E-158
Foxp1	-0.872133132	0.414	0.306	5.18E-104
Ncl	-0.872396184	0.464	0.339	9.35E-75
Nrip1	-0.873518313	0.128	0.129	1.25E-64
Atp2b1	-0.874394379	0.327	0.243	4.87E-157
Jak1	-0.8768237	0.444	0.319	1.06E-49
Polr2a	-0.877672794	0.258	0.195	5.23E-54
Rnf149	-0.878796282	0.515	0.36	7.6E-146
Il4ra	-0.879324622	0.291	0.22	2.1E-39
Tpt1	-0.880034201	0.964	0.909	1.05E-180
Junb	-0.880822188	0.969	0.887	5.67E-173
Mt-mt-Rnr2	-0.881133062	0.944	0.822	1.35E-198
Nmt1	-0.881515705	0.185	0.167	9.15E-194
Ppp3ca	-0.884984441	0.249	0.199	9.29E-125
Tnrc6b	-0.89441532	0.149	0.148	8.79E-107
Snrnp70	-0.89648451	0.347	0.256	2.56E-36
Esyt2	-0.898715374	0.167	0.155	6.88E-133
Rassf5	-0.899860005	0.22	0.181	9.92E-175
Tnks2	-0.903433015	0.159	0.154	1.99E-141
Zc3h13	-0.90683708	0.103	0.121	6.36E-59
Gcnt2	-0.907994175	0.295	0.228	2.44E-135
Ogt	-0.912281367	0.151	0.15	5.52E-122
Cmip	-0.915585386	0.196	0.18	7.69E-166
Atf4	-0.915693435	0.56	0.427	9.25E-178
Heca	-0.917887307	0.153	0.147	8.86E-114
Ddx3y	-0.918969571	0.258	0.207	8.18E-75
Eif3j2	-0.924277245	0.161	0.162	2.85E-142
Wsb1	-0.925007078	0.265	0.21	3.38E-170

Ddx17	-0.925270728	0.307	0.241	8.05E-193
Runx2	-0.927075512	0.211	0.175	3.94E-175
Map3k1	-0.927307729	0.248	0.21	1.52E-172
Pnisr	-0.92969985	0.222	0.194	9.8389E-147
Ptgs2	-0.931396863	0.221	0.158	1.47E-119
Lbr	-0.933105191	0.361	0.283	6.75E-164
Runx3	-0.93392421	0.256	0.214	7.14E-94
Csrnp1	-0.934611809	0.373	0.282	1.43E-191
Trim30a	-0.937736652	0.306	0.252	1.58E-101
Mcl1	-0.93780687	0.752	0.576	9.78E-155
Hexim1	-0.938502976	0.232	0.202	9.259E-146
Rab8b	-0.943051446	0.285	0.231	2.21E-115
Tra2a	-0.944449394	0.224	0.198	3.5521E-145
Celf1	-0.94462292	0.206	0.183	6.78E-191
Gramd3	-0.945904741	0.267	0.216	2.47E-140
Nabp1	-0.957203452	0.22	0.195	1.98E-159
Purb	-0.962749039	0.194	0.178	6.06E-186
Sp100	-0.967027395	0.349	0.276	1.06E-189
Egr1	-0.968160271	0.143	0.131	4.62E-52
Il2rb	-0.969845421	0.324	0.252	1.06E-111
Prpf38b	-0.975129381	0.303	0.246	2.45E-79
Thbs1	-0.976034894	0.453	0.268	8.64E-88
Ccr9	-0.980614723	0.146	0.123	4.11E-69
Crebrf	-0.983294417	0.147	0.157	3.35E-128
Atp1b3	-0.984330287	0.553	0.405	7E-133
Eif3a	-0.984756867	0.353	0.282	5.33E-147
Rora	-0.988255504	0.088	0.107	3.1E-20
Ptbp3	-0.988836739	0.409	0.324	1.13E-142
Hnrnpm	-0.995927568	0.42	0.329	3.73E-199
Gpr132	-1.002740505	0.409	0.327	1.46E-145
Cnot6l	-1.012252131	0.2	0.187	5.81E-198
Rdm1	-1.013276735	0.338	0.27	1.22E-50
Pabpc4	-1.013585413	0.108	0.135	8.6E-72
Nr4a3	-1.013644926	0.187	0.179	1.9E-150
Tnfaip2	-1.02068465	0.229	0.194	3.02E-158
Nfkbiz	-1.023439005	0.48	0.364	2.12E+41
Ythdc1	-1.02736413	0.289	0.247	5.22E-46
Hnrnph1	-1.028520643	0.395	0.312	1.78E-162
Vps37b	-1.031602705	0.657	0.556	1.12E-111
Ddx21	-1.033571471	0.248	0.224	9.14E-200
Amd1	-1.053896803	0.118	0.142	5.07E-75
Tob2	-1.055422305	0.192	0.181	8.34E-198
Mbnl1	-1.058584129	0.533	0.424	1.24E-173
Clk1	-1.059244634	0.463	0.374	108000000
Ahnak	-1.061295528	0.497	0.385	1.15E-185
Tcf4	-1.069655457	0.268	0.229	8.42E-158
Ankrd11	-1.072875583	0.212	0.205	5.16E-203
Luc7l2	-1.077407489	0.469	0.371	3.97E-103
Fus	-1.085684237	0.318	0.281	3.64E-73
Kdm7a	-1.086414879	0.299	0.265	3.14E-184
Pcbp2	-1.094071055	0.586	0.48	1.62E-115
Gls	-1.097224097	0.226	0.22	1.75E-59
Tcp11l2	-1.10176068	0.254	0.234	3.61E-153
Snx18	-1.108821066	0.35	0.303	2.35E-135
Akna	-1.112535721	0.196	0.2	6.59E-199
Hnrnpa0	-1.114247463	0.317	0.289	5.5E-188
Dusp1	-1.119008155	0.789	0.657	8.53E-171
Nr4a1	-1.11997327	0.566	0.456	9.98E-160

Bhlhe40	-1.12369017	0.244	0.222	6.6136E-143
Ptprc	-1.124245368	0.732	0.62	1.65E-184
Zcchc11	-1.13091744	0.156	0.181	2.74E-134
Itgal	-1.145090702	0.488	0.41	2.13E-185
Zfp36l2	-1.146813018	0.632	0.52	3.09E-33
Marcks	-1.148263279	0.332	0.274	1.98E-177
Bcl2l11	-1.156645073	0.405	0.351	3.94E-129
Nr4a2	-1.162859513	0.183	0.184	4.09E-146
Prrc2c	-1.166127376	0.2	0.219	1.01E-197
Cd44	-1.199275188	0.64	0.541	3.4E-176
Prpf4b	-1.20266044	0.229	0.249	1.25E+16
Klf6	-1.224874391	0.647	0.553	1.7E-145
Celf2	-1.253802995	0.4	0.379	2.43E-149
Slc38a2	-1.25518749	0.386	0.361	1.61E-194
Cxcr4	-1.282984626	0.58	0.526	1.16E-75
Ets1	-1.289199941	0.579	0.523	1.33E-104
Ccnl1	-1.294642492	0.434	0.405	1.4E-171
Stk17b	-1.300743156	0.789	0.719	9.02E-82
P2ry10	-1.302224475	0.28	0.287	4.6E-159
Ddx5	-1.302487126	0.908	0.86	9.68E-169
Tra2b	-1.315095056	0.552	0.509	1.55E-158
Btg1	-1.329235855	0.978	0.946	2.04E-172
Ddx6	-1.333083313	0.382	0.376	3.22E-192
Rps28	-1.352347779	0.067	0.164	3.94E-56
Peli1	-1.35705794	0.31	0.315	1.13E-124
Fosl2	-1.390990625	0.546	0.494	1.47E-90
Mt-mt-Rnr1	-1.405919987	0.176	0.23	2.95E-193
Dennd4a	-1.423192513	0.414	0.416	9.63E-179
Satb1	-1.426454673	0.445	0.429	4.79E-96
Zfp36l1	-1.430311244	0.431	0.417	2.54E-86
Hspa1b	-1.49359165	0.4	0.358	1.85E-188
Irf2bp2	-1.599169737	0.369	0.391	3.78E-159
Tnfaip3	-1.619505679	0.528	0.541	4.3E-132
Hbb-bs	-2.414883884	0	0.144	1.08E-32

Supplementary Table 5

gene	p_val	avg_log2FC	pct.1	pct.2	p_val_adj	cluster
Thbs1	3.64E-102	1.513873113	0.768	0.463	4.88E-98	0-Mφ-Thbs1
Fn1	8.29E-87	1.241385333	0.628	0.302	1.11E-82	0-Mφ-Thbs1
Lyz2	1.87E-145	1.206668319	0.98	0.675	2.50E-141	0-Mφ-Thbs1
F13a1	4.67E-65	1.130731663	0.537	0.266	6.25E-61	0-Mφ-Thbs1
Mafb	1.36E-50	1.092822933	0.528	0.281	1.82E-46	0-Mφ-Thbs1
Mgst1	1.11E-46	0.988733464	0.472	0.251	1.48E-42	0-Mφ-Thbs1
Chil3	8.61E-43	0.976318784	0.515	0.298	1.15E-38	0-Mφ-Thbs1
Vcan	1.31E-34	0.889460804	0.348	0.158	1.75E-30	0-Mφ-Thbs1
Ifitm6	4.81E-32	0.855654137	0.4	0.221	6.45E-28	0-Mφ-Thbs1
Gm9733	1.14E-29	0.842533544	0.31	0.144	1.53E-25	0-Mφ-Thbs1
Gda	4.59E-59	0.84109113	0.696	0.43	6.15E-55	0-Mφ-Thbs1
Ifitm3	2.19E-108	0.789078757	0.973	0.797	2.93E-104	0-Mφ-Thbs1
Gsr	8.52E-62	0.782401031	0.779	0.524	1.14E-57	0-Mφ-Thbs1
Smpdl3a	1.89E-30	0.768143899	0.455	0.279	2.52E-26	0-Mφ-Thbs1
Ccr2	3.48E-53	0.744131927	0.692	0.452	4.66E-49	0-Mφ-Thbs1
Klra2	5.40E-20	0.694441152	0.294	0.162	7.23E-16	0-Mφ-Thbs1
Nfil3	5.81E-26	0.689028315	0.508	0.341	7.77E-22	0-Mφ-Thbs1
Emilin2	1.09E-36	0.687954976	0.609	0.406	1.46E-32	0-Mφ-Thbs1
Fcgr3	6.99E-21	0.684868958	0.373	0.238	9.36E-17	0-Mφ-Thbs1
Anxa1	6.15E-20	0.675305893	0.382	0.251	8.23E-16	0-Mφ-Thbs1
Ms4a6c	2.60E-52	0.669957395	0.806	0.6	3.48E-48	0-Mφ-Thbs1
Dbi	2.75E-38	0.667095021	0.669	0.499	3.68E-34	0-Mφ-Thbs1
Plac8	5.79E-81	0.64204999	0.978	0.838	7.75E-77	0-Mφ-Thbs1
Ccl6	9.58E-34	0.630689994	0.697	0.54	1.28E-29	0-Mφ-Thbs1
Ifi27l2a	4.57E-44	0.622266178	0.861	0.716	6.11E-40	0-Mφ-Thbs1
Tpd52	3.24E-39	0.617377514	0.709	0.507	4.33E-35	0-Mφ-Thbs1
Ahnak	8.23E-36	0.612808077	0.747	0.578	1.10E-31	0-Mφ-Thbs1
Tgfbi	1.88E-38	0.604756361	0.716	0.501	2.52E-34	0-Mφ-Thbs1
Emb	1.02E-17	0.591665665	0.412	0.29	1.36E-13	0-Mφ-Thbs1
Ms4a4c	1.57E-16	0.585803512	0.421	0.299	2.11E-12	0-Mφ-Thbs1
Sat1	5.64E-39	0.583135417	0.808	0.628	7.55E-35	0-Mφ-Thbs1
Klf13	1.73E-19	0.57829909	0.453	0.316	2.32E-15	0-Mφ-Thbs1
Mt1	1.40E-09	0.566791318	0.426	0.341	1.88E-05	0-Mφ-Thbs1
Vsir	1.26E-16	0.561868366	0.42	0.297	1.68E-12	0-Mφ-Thbs1
Rnf149	5.87E-32	0.561577021	0.798	0.632	7.86E-28	0-Mφ-Thbs1
Aprt	2.41E-15	0.544660098	0.377	0.261	3.22E-11	0-Mφ-Thbs1
Pla2g7	2.81E-20	0.531868501	0.474	0.324	3.76E-16	0-Mφ-Thbs1
Fos	2.70E-39	0.522110827	0.968	0.892	3.62E-35	0-Mφ-Thbs1
S100a4	1.19E-34	0.514080192	0.839	0.673	1.60E-30	0-Mφ-Thbs1
Ccl9	8.71E-16	0.506344776	0.45	0.333	1.17E-11	0-Mφ-Thbs1
**gene**	**p_val**	**avg_log2FC**	**pct.1**	**pct.2**	**p_val_adj**	**cluster**
Cd209a	2.52E-91	2.171890889	0.319	0.042	3.37E-87	1-DC
H2-Eb1	8.63E-118	2.139337557	0.85	0.459	1.15E-113	1-DC
H2-Ab1	1.79E-123	1.954424897	0.923	0.591	2.39E-119	1-DC
H2-Aa	4.06E-104	1.910796589	0.841	0.48	5.43E-100	1-DC
Cd74	5.79E-117	1.729860319	0.969	0.801	7.75E-113	1-DC
Klrd1	1.30E-41	1.475617649	0.296	0.086	1.74E-37	1-DC
H2-DMb1	4.71E-50	1.311847076	0.5	0.204	6.30E-46	1-DC
Cst3	1.29E-26	1.271365611	0.889	0.842	1.73E-22	1-DC
Bcl11a	1.95E-28	1.175264107	0.257	0.085	2.61E-24	1-DC
Bhlhe40	3.60E-19	1.137049976	0.427	0.253	4.82E-15	1-DC
Ckb	1.81E-45	1.116543559	0.257	0.057	2.42E-41	1-DC
H2-DMa	1.21E-33	1.033902524	0.551	0.298	1.62E-29	1-DC
Tcf4	2.86E-12	1.019659679	0.352	0.219	3.83E-08	1-DC
Cbfa2t3	3.45E-28	1.002866262	0.385	0.168	4.62E-24	1-DC

Cd83	2.90E-19	0.95474439	0.564	0.381	3.89E-15	1-DC
Gm2a	2.59E-29	0.905313943	0.697	0.465	3.47E-25	1-DC
Ywhah	1.82E-19	0.902017792	0.387	0.205	2.43E-15	1-DC
P2ry10	1.79E-26	0.858239052	0.403	0.183	2.39E-22	1-DC
Bst2	1.12E-09	0.841753137	0.473	0.357	1.49E-05	1-DC
Jak2	1.44E-20	0.815440778	0.285	0.12	1.93E-16	1-DC
Nr4a3	1.06E-16	0.804476167	0.376	0.208	1.42E-12	1-DC
Cd24a	6.92E-14	0.788393839	0.277	0.143	9.27E-10	1-DC
H2afy	2.40E-21	0.786956806	0.659	0.46	3.21E-17	1-DC
Pmaip1	2.22E-10	0.771500223	0.538	0.402	2.98E-06	1-DC
Ccdc88a	5.93E-16	0.755529153	0.312	0.155	7.93E-12	1-DC
Gcnt2	1.55E-06	0.754339049	0.454	0.371	0.020767114	1-DC
Nfkb1	1.90E-13	0.752390748	0.425	0.269	2.54E-09	1-DC
Set	7.11E-20	0.751438661	0.513	0.308	9.52E-16	1-DC
Dpysl2	3.90E-16	0.746008835	0.27	0.125	5.22E-12	1-DC
Id3	5.56E-08	0.729213535	0.369	0.265	0.000744632	1-DC
Syngr2	3.05E-13	0.728637858	0.416	0.268	4.08E-09	1-DC
Gng10	1.01E-15	0.68686121	0.511	0.338	1.35E-11	1-DC
Skil	2.56E-10	0.684548107	0.372	0.238	3.42E-06	1-DC
Ptms	3.77E-14	0.679091498	0.303	0.159	5.05E-10	1-DC
Hk2	6.55E-06	0.670129581	0.338	0.243	0.087741315	1-DC
Mef2c	3.16E-10	0.634201668	0.305	0.18	4.23E-06	1-DC
Plbd1	4.75E-11	0.626893226	0.412	0.273	6.36E-07	1-DC
Eef1g	2.56E-12	0.613667137	0.527	0.388	3.43E-08	1-DC
Dock10	1.33E-11	0.604917732	0.381	0.232	1.78E-07	1-DC
Nap1l1	4.43E-09	0.580514417	0.316	0.2	5.93E-05	1-DC
Dennd4a	5.51E-07	0.578610893	0.489	0.381	0.007370693	1-DC
Nr4a2	8.26E-06	0.573571906	0.358	0.27	0.110555796	1-DC
Gsn	3.85E-11	0.570028912	0.261	0.14	5.15E-07	1-DC
Baz1a	8.99E-13	0.56689872	0.35	0.197	1.20E-08	1-DC
St3gal4	6.30E-05	0.566625838	0.473	0.4	0.843388471	1-DC
Lgals1	1.34E-05	0.564950654	0.482	0.395	0.179523339	1-DC
Tnfaip8	1.42E-07	0.564881401	0.265	0.169	0.001899419	1-DC
Ptma	3.27E-38	0.555467984	0.969	0.908	4.37E-34	1-DC
Mbnl1	3.97E-12	0.553552957	0.617	0.472	5.32E-08	1-DC
Hnrnpa1	3.01E-08	0.538617193	0.365	0.252	0.000403214	1-DC
Lsp1	5.45E-15	0.535030265	0.684	0.534	7.29E-11	1-DC
Anp32e	9.89E-08	0.531896822	0.27	0.171	0.001324484	1-DC
Hspe1	3.64E-09	0.520114384	0.555	0.451	4.87E-05	1-DC
Psmb8	2.41E-08	0.51272069	0.48	0.37	0.000322583	1-DC
Herpud1	5.40E-09	0.509674572	0.442	0.311	7.23E-05	1-DC

**gene**	**p_val**	**avg_log2FC**	**pct.1**	**pct.2**	**p_val_adj**	**cluster**
Cxcl9	5.87E-91	2.714603714	0.289	0.029	7.86E-87	2-Mφ-Cxcl9
Lpl	1.86E-101	2.635973939	0.347	0.04	2.50E-97	2-Mφ-Cxcl9
Cxcl10	2.54E-48	2.591232918	0.329	0.087	3.39E-44	2-Mφ-Cxcl9
Il1rn	1.25E-74	2.154590336	0.554	0.176	1.68E-70	2-Mφ-Cxcl9
Gbp2	1.41E-61	1.883181774	0.297	0.054	1.89E-57	2-Mφ-Cxcl9
Bcl2a1b	5.36E-88	1.864375787	0.576	0.162	7.18E-84	2-Mφ-Cxcl9
Isg15	1.85E-32	1.675744855	0.501	0.249	2.47E-28	2-Mφ-Cxcl9
Mmp14	4.10E-101	1.663714131	0.282	0.021	5.49E-97	2-Mφ-Cxcl9
Sdc4	4.18E-45	1.630553883	0.327	0.09	5.60E-41	2-Mφ-Cxcl9
Apoe	7.19E-11	1.57373028	0.584	0.459	9.63E-07	2-Mφ-Cxcl9
Bcl2a1d	8.32E-59	1.371036041	0.406	0.106	1.11E-54	2-Mφ-Cxcl9
Cstb	7.18E-31	1.308449568	0.599	0.361	9.61E-27	2-Mφ-Cxcl9
Aif1	2.35E-43	1.274876002	0.411	0.14	3.14E-39	2-Mφ-Cxcl9
Cxcl16	2.89E-55	1.239212064	0.347	0.079	3.87E-51	2-Mφ-Cxcl9
Nampt	2.97E-31	1.199264421	0.436	0.192	3.97E-27	2-Mφ-Cxcl9
Rgs1	3.91E-14	1.167170643	0.491	0.324	5.24E-10	2-Mφ-Cxcl9

Clic4	9.54E-35	1.138280972	0.439	0.178	1.28E-30	2-Mφ-Cxcl9
Sod2	5.39E-27	1.110850905	0.434	0.21	7.21E-23	2-Mφ-Cxcl9
Marcksl1	2.49E-24	1.105901486	0.486	0.258	3.34E-20	2-Mφ-Cxcl9
Cd274	6.58E-26	1.096818037	0.259	0.086	8.81E-22	2-Mφ-Cxcl9
Ccl4	2.13E-06	1.083347558	0.676	0.558	0.028506265	2-Mφ-Cxcl9
Mif	3.08E-27	1.081847199	0.469	0.237	4.12E-23	2-Mφ-Cxcl9
LOC10086	1.36E-15	1.078980318	0.788	0.656	1.82E-11	2-Mφ-Cxcl9
Ccrl2	5.44E-24	1.042889244	0.536	0.303	7.28E-20	2-Mφ-Cxcl9
Clec4e	6.94E-15	1.028046256	0.559	0.386	9.28E-11	2-Mφ-Cxcl9
Ftl1	2.87E-14	1.0239848	0.82	0.708	3.84E-10	2-Mφ-Cxcl9
Malt1	7.80E-22	1.012327383	0.337	0.149	1.04E-17	2-Mφ-Cxcl9
H2-Aa	9.14E-31	1.005505072	0.748	0.501	1.22E-26	2-Mφ-Cxcl9
H2-Eb1	4.70E-27	0.986024966	0.723	0.486	6.29E-23	2-Mφ-Cxcl9
Cd74	2.85E-39	0.979574641	0.955	0.806	3.81E-35	2-Mφ-Cxcl9
Cd9	1.78E-15	0.94177745	0.292	0.144	2.38E-11	2-Mφ-Cxcl9
Ptgs2	9.63E-11	0.936609933	0.431	0.289	1.29E-06	2-Mφ-Cxcl9
H2-Ab1	9.78E-29	0.931367829	0.808	0.615	1.31E-24	2-Mφ-Cxcl9
Clec7a	1.70E-23	0.930547354	0.561	0.321	2.28E-19	2-Mφ-Cxcl9
Tgm2	1.50E-29	0.929239699	0.344	0.124	2.01E-25	2-Mφ-Cxcl9
Tpi1	9.33E-19	0.902670744	0.322	0.149	1.25E-14	2-Mφ-Cxcl9
Acod1	2.52E-14	0.882251385	0.309	0.157	3.38E-10	2-Mφ-Cxcl9
Fth1	3.47E-36	0.874017989	0.988	0.974	4.65E-32	2-Mφ-Cxcl9
Tnfaip2	1.57E-13	0.86487849	0.499	0.324	2.10E-09	2-Mφ-Cxcl9
Cflar	1.63E-14	0.861689268	0.362	0.197	2.18E-10	2-Mφ-Cxcl9
Mnda	1.72E-14	0.838204097	0.317	0.163	2.31E-10	2-Mφ-Cxcl9
Cxcl2	2.22E-08	0.779186922	0.741	0.6	0.000296583	2-Mφ-Cxcl9
Ctsb	2.36E-18	0.777566676	0.758	0.629	3.16E-14	2-Mφ-Cxcl9
Psme2b	1.07E-21	0.769594251	0.608	0.374	1.43E-17	2-Mφ-Cxcl9
Prdx1	4.56E-06	0.728760348	0.589	0.501	0.060986968	2-Mφ-Cxcl9
Slc15a3	1.11E-13	0.722820079	0.464	0.292	1.49E-09	2-Mφ-Cxcl9
Ccl3	0.00288867	0.721198457	0.374	0.307	1	2-Mφ-Cxcl9
Prkcd	2.69E-15	0.712539736	0.551	0.347	3.60E-11	2-Mφ-Cxcl9
Ctsz	5.63E-17	0.680292808	0.611	0.438	7.54E-13	2-Mφ-Cxcl9
Pgk1	2.90E-14	0.673777513	0.456	0.276	3.89E-10	2-Mφ-Cxcl9
Gadd45b	3.82E-15	0.669254002	0.571	0.372	5.11E-11	2-Mφ-Cxcl9
Adam17	2.25E-12	0.657902326	0.264	0.131	3.01E-08	2-Mφ-Cxcl9
Icam1	4.47E-08	0.633096385	0.327	0.209	0.000598979	2-Mφ-Cxcl9
Ptafr	6.11E-12	0.617785579	0.481	0.31	8.18E-08	2-Mφ-Cxcl9
H2-DMa	1.01E-08	0.612162516	0.446	0.319	0.000134742	2-Mφ-Cxcl9
Ifi203	4.96E-09	0.60499328	0.262	0.147	6.64E-05	2-Mφ-Cxcl9
Atp6v0c	5.96E-17	0.602998736	0.786	0.634	7.97E-13	2-Mφ-Cxcl9
Ctsc	1.01E-11	0.600973305	0.633	0.474	1.35E-07	2-Mφ-Cxcl9
Dusp2	2.35E-07	0.599211818	0.494	0.365	0.003151248	2-Mφ-Cxcl9
H2-DMb1	4.37E-10	0.591242701	0.367	0.229	5.84E-06	2-Mφ-Cxcl9
Gngt2	1.38E-10	0.590143175	0.579	0.437	1.85E-06	2-Mφ-Cxcl9
B2m	5.64E-42	0.58749411	0.993	0.956	7.55E-38	2-Mφ-Cxcl9
Clec12a	8.05E-14	0.586716518	0.379	0.21	1.08E-09	2-Mφ-Cxcl9
C3	2.82E-12	0.577536498	0.354	0.199	3.78E-08	2-Mφ-Cxcl9
Sqstm1	1.05E-06	0.574399721	0.294	0.193	0.014050404	2-Mφ-Cxcl9
Gna13	1.67E-11	0.549381226	0.399	0.236	2.23E-07	2-Mφ-Cxcl9
Csf1r	5.44E-13	0.544641661	0.606	0.405	7.29E-09	2-Mφ-Cxcl9
Ifi204	4.00E-07	0.54383929	0.367	0.252	0.005358932	2-Mφ-Cxcl9
F10	7.53E-06	0.536321945	0.389	0.286	0.100824421	2-Mφ-Cxcl9
Tgif1	8.64E-08	0.531976561	0.451	0.323	0.00115654	2-Mφ-Cxcl9
Irf8	3.22E-05	0.524855051	0.529	0.413	0.430948231	2-Mφ-Cxcl9
Gapdh	1.06E-10	0.523263504	0.676	0.532	1.42E-06	2-Mφ-Cxcl9
Cd83	4.64E-11	0.51848771	0.566	0.384	6.21E-07	2-Mφ-Cxcl9
Anxa5	1.77E-09	0.515568006	0.509	0.36	2.37E-05	2-Mφ-Cxcl9

Id2	2.75E-08	0.514342457	0.501	0.358	0.000368362	2-Mφ-Cxcl9
Lgmn	6.91E-07	0.510554731	0.489	0.372	0.009249981	2-Mφ-Cxcl9
**gene**	**p_val**	**avg_log2FC**	**pct.1**	**pct.2**	**p_val_adj**	**cluster**
Ear2	1.56E-16	1.519583375	0.705	0.219	2.08E-12	3-Mφ-Gngt2
Ace	1.61E-27	1.201138881	0.614	0.078	2.15E-23	3-Mφ-Gngt2
Gngt2	5.05E-12	1.098237354	0.526	0.449	6.76E-08	3-Mφ-Gngt2
Itgal	7.60E-06	0.871344973	0.548	0.529	0.101667128	3-Mφ-Gngt2
Adgre4	1.47E-08	0.792515827	0.455	0.149	0.000197089	3-Mφ-Gngt2
Hes1	0.0000206	0.676264953	0.523	0.268	0.275988256	3-Mφ-Gngt2
Pglyrp1	0.005416995	0.638626847	0.682	0.567	1	3-Mφ-Gngt2
Mt-mt-Rnr2	3.36E-05	0.616777988	0.79	0.861	0.450005118	3-Mφ-Gngt2
Cd300c2	0.0000847	0.566591301	0.659	0.506	1	3-Mφ-Gngt2
Pou2f2	0.010452931	0.559771184	0.25	0.126	1	3-Mφ-Gngt2
Hspa1a	0.013189501	0.532872903	0.864	0.84	1	3-Mφ-Gngt2
Clec4a1	0.0000195	0.530218535	0.773	0.526	0.260787235	3-Mφ-Gngt2
**gene**	**p_val**	**avg_log2FC**	**pct.1**	**pct.2**	**p_val_adj**	**cluster**
Saa3	3.02E-43	2.980229449	0.5	0.129	4.04E-39	4-Mφ-Cxcl2
Cxcl2	2.16E-63	2.498442113	0.95	0.6	2.89E-59	4-Mφ-Cxcl2
Tnfsf9	1.42E-74	2.218130974	0.519	0.08	1.90E-70	4-Mφ-Cxcl2
Il6	1.49E-82	2.201257675	0.394	0.037	1.99E-78	4-Mφ-Cxcl2
Nfkbia	8.61E-70	1.886406043	0.994	0.734	1.15E-65	4-Mφ-Cxcl2
Tnf	1.04E-50	1.832512376	0.506	0.108	1.39E-46	4-Mφ-Cxcl2
Nlrp3	4.19E-55	1.804597934	0.769	0.23	5.61E-51	4-Mφ-Cxcl2
Nfkbiz	1.22E-47	1.797827486	0.887	0.421	1.63E-43	4-Mφ-Cxcl2
Ptgs2	7.54E-42	1.78219853	0.769	0.282	1.01E-37	4-Mφ-Cxcl2
Clec4e	2.95E-58	1.724265092	0.9	0.382	3.94E-54	4-Mφ-Cxcl2
Ccrl2	2.38E-46	1.657380707	0.794	0.308	3.19E-42	4-Mφ-Cxcl2
Cd14	6.24E-40	1.546094857	0.869	0.488	8.36E-36	4-Mφ-Cxcl2
Tnfaip3	1.55E-37	1.494215807	0.925	0.489	2.08E-33	4-Mφ-Cxcl2
Ccl3	7.83E-24	1.374144765	0.662	0.297	1.05E-19	4-Mφ-Cxcl2
Marcksl1	7.21E-29	1.350565005	0.656	0.267	9.65E-25	4-Mφ-Cxcl2
Chil3	3.46E-19	1.312748555	0.688	0.395	4.63E-15	4-Mφ-Cxcl2
F10	1.09E-35	1.308112394	0.725	0.276	1.46E-31	4-Mφ-Cxcl2
Arl5c	1.20E-35	1.29250732	0.494	0.132	1.61E-31	4-Mφ-Cxcl2
Sod2	8.21E-29	1.25931304	0.6	0.22	1.10E-24	4-Mφ-Cxcl2
Cflar	1.74E-28	1.227876941	0.569	0.199	2.32E-24	4-Mφ-Cxcl2
Gadd45b	1.06E-28	1.210304402	0.769	0.377	1.42E-24	4-Mφ-Cxcl2
Il1b	4.84E-33	1.180670519	0.975	0.826	6.48E-29	4-Mφ-Cxcl2
Lsmem1	2.89E-33	1.13722963	0.275	0.046	3.87E-29	4-Mφ-Cxcl2
Ehd1	3.43E-24	1.106271869	0.45	0.145	4.59E-20	4-Mφ-Cxcl2
Cxcl10	8.14E-25	1.096166161	0.375	0.105	1.09E-20	4-Mφ-Cxcl2
Ptafr	3.88E-18	1.045624362	0.644	0.315	5.20E-14	4-Mφ-Cxcl2
Egr1	1.19E-14	1.045101182	0.419	0.174	1.60E-10	4-Mφ-Cxcl2
Dusp2	5.29E-18	1.041122426	0.688	0.365	7.08E-14	4-Mφ-Cxcl2
Slpi	5.68E-16	1.006887146	0.669	0.397	7.61E-12	4-Mφ-Cxcl2
Tnfaip2	1.37E-18	0.969591892	0.688	0.328	1.83E-14	4-Mφ-Cxcl2
Ifrd1	3.39E-18	0.952676781	0.863	0.608	4.54E-14	4-Mφ-Cxcl2
Icam1	2.74E-19	0.92497148	0.519	0.208	3.67E-15	4-Mφ-Cxcl2
Slfn2	1.31E-17	0.921732284	0.8	0.518	1.76E-13	4-Mφ-Cxcl2
Ier3	5.47E-14	0.909462905	0.637	0.365	7.32E-10	4-Mφ-Cxcl2
Pde4b	8.98E-20	0.908324625	0.725	0.353	1.20E-15	4-Mφ-Cxcl2
Hilpda	1.65E-11	0.901650805	0.388	0.179	2.21E-07	4-Mφ-Cxcl2
Tlr2	5.40E-20	0.89325161	0.55	0.222	7.23E-16	4-Mφ-Cxcl2
Spata13	3.58E-16	0.884011153	0.319	0.104	4.79E-12	4-Mφ-Cxcl2
Wfdc21	4.57E-11	0.857612523	0.5	0.262	6.12E-07	4-Mφ-Cxcl2
Bcl2l11	4.90E-13	0.857243662	0.762	0.458	6.56E-09	4-Mφ-Cxcl2
Socs3	6.27E-12	0.851268709	0.488	0.242	8.39E-08	4-Mφ-Cxcl2
Mmp8	3.69E-15	0.846109329	0.469	0.197	4.93E-11	4-Mφ-Cxcl2

Il1rn	2.21E-13	0.839200204	0.469	0.212	2.95E-09	4-Mφ-Cxcl2
Slc7a11	1.09E-16	0.816307801	0.312	0.097	1.46E-12	4-Mφ-Cxcl2
Hp	9.30E-15	0.799393434	0.819	0.547	1.25E-10	4-Mφ-Cxcl2
Arg2	5.69E-14	0.795592319	0.3	0.102	7.62E-10	4-Mφ-Cxcl2
Ppp1r15a	1.67E-11	0.785391973	0.769	0.505	2.24E-07	4-Mφ-Cxcl2
Bcl2a1b	9.26E-10	0.778535268	0.412	0.206	1.24E-05	4-Mφ-Cxcl2
Mapk6	9.17E-16	0.758702693	0.412	0.156	1.23E-11	4-Mφ-Cxcl2
Maff	6.14E-14	0.755762095	0.288	0.096	8.22E-10	4-Mφ-Cxcl2
Trem1	3.25E-19	0.750549559	0.419	0.141	4.35E-15	4-Mφ-Cxcl2
Tgif1	1.48E-12	0.733796898	0.619	0.325	1.97E-08	4-Mφ-Cxcl2
Csrnp1	2.06E-10	0.723701038	0.7	0.41	2.75E-06	4-Mφ-Cxcl2
Clec4d	2.54E-11	0.719007033	0.494	0.244	3.40E-07	4-Mφ-Cxcl2
Plek	8.07E-12	0.710181808	0.744	0.464	1.08E-07	4-Mφ-Cxcl2
Gadd45a	2.77E-08	0.701850608	0.5	0.289	0.000370749	4-Mφ-Cxcl2
Tnip1	7.01E-13	0.696704678	0.294	0.104	9.39E-09	4-Mφ-Cxcl2
Kdm6b	4.37E-12	0.69483487	0.625	0.333	5.84E-08	4-Mφ-Cxcl2
Txnrd1	2.00E-12	0.688495583	0.35	0.139	2.68E-08	4-Mφ-Cxcl2
Nupr1	6.30E-10	0.684574822	0.425	0.209	8.44E-06	4-Mφ-Cxcl2
C5ar1	1.23E-15	0.68219958	0.369	0.132	1.65E-11	4-Mφ-Cxcl2
Mgst1	6.66E-13	0.654378896	0.656	0.349	8.91E-09	4-Mφ-Cxcl2
Sowahc	8.59E-09	0.637579639	0.431	0.222	0.000114932	4-Mφ-Cxcl2
Rab20	1.29E-06	0.603473473	0.306	0.157	0.017308159	4-Mφ-Cxcl2
Acod1	2.43E-06	0.596641666	0.325	0.169	0.032528132	4-Mφ-Cxcl2
Marcks	4.30E-06	0.57590333	0.744	0.537	0.057518718	4-Mφ-Cxcl2
Ninj1	5.58E-10	0.560774293	0.475	0.243	7.47E-06	4-Mφ-Cxcl2
Smpdl3a	4.26E-09	0.559707689	0.606	0.356	5.70E-05	4-Mφ-Cxcl2
Prdx6	9.94E-08	0.556952212	0.475	0.274	0.00133078	4-Mφ-Cxcl2
Tnfrsf1b	8.47E-07	0.544519106	0.562	0.35	0.011337891	4-Mφ-Cxcl2
Cited2	4.29E-08	0.542103336	0.388	0.199	0.000573723	4-Mφ-Cxcl2
Zfp36	4.90E-08	0.518897597	0.956	0.814	0.000656243	4-Mφ-Cxcl2
Trem3	9.86E-12	0.518303691	0.262	0.091	1.32E-07	4-Mφ-Cxcl2
Ccng2	1.91E-08	0.516344992	0.306	0.136	0.000255347	4-Mφ-Cxcl2
Sgk1	1.02E-07	0.513261166	0.412	0.217	0.001360558	4-Mφ-Cxcl2
Batf	4.52E-09	0.512025254	0.269	0.11	6.04E-05	4-Mφ-Cxcl2
Nfe2l2	3.59E-08	0.510761326	0.762	0.515	0.000480084	4-Mφ-Cxcl2
Lrg1	9.23E-06	0.509677585	0.294	0.159	0.123504478	4-Mφ-Cxcl2

**gene**	**p_val**	**avg_log2FC**	**pct.1**	**pct.2**	**p_val_adj**	**cluster**
Ccr7	1.02E-48	4.104841845	0.863	0.181	1.37E-44	5-Mφ-Fscn1
Fscn1	7.86E-245	3.548562684	0.608	0.007	1.05E-240	5-Mφ-Fscn1
Tmem123	1.86E-54	3.431634747	0.902	0.17	2.50E-50	5-Mφ-Fscn1
Tbc1d4	8.17E-83	3.095382835	0.588	0.036	1.09E-78	5-Mφ-Fscn1
Il12b	4.06E-96	3.040543683	0.294	0.005	5.44E-92	5-Mφ-Fscn1
Basp1	1.49E-43	2.982146394	0.686	0.104	2.00E-39	5-Mφ-Fscn1
Fabp5	9.03E-30	2.902068712	0.608	0.121	1.21E-25	5-Mφ-Fscn1
Mreg	8.03E-145	2.788854079	0.451	0.008	1.07E-140	5-Mφ-Fscn1
Ccl22	3.33E-81	2.538466672	0.314	0.008	4.45E-77	5-Mφ-Fscn1
Cd200	6.42E-59	2.533663657	0.412	0.024	8.59E-55	5-Mφ-Fscn1
Tspan3	5.11E-63	2.531843874	0.49	0.033	6.85E-59	5-Mφ-Fscn1
Cacnb3	1.25E-208	2.443969294	0.431	0.003	1.67E-204	5-Mφ-Fscn1
Relb	1.66E-33	2.384929847	0.588	0.093	2.22E-29	5-Mφ-Fscn1
Cd63	4.49E-54	2.381342216	0.392	0.024	6.01E-50	5-Mφ-Fscn1
Cxcl16	2.19E-32	2.331648899	0.608	0.106	2.94E-28	5-Mφ-Fscn1
Id2	8.43E-24	2.324904648	0.882	0.368	1.13E-19	5-Mφ-Fscn1
Zmynd15	2.17E-157	2.322985528	0.412	0.005	2.91E-153	5-Mφ-Fscn1
Apol7c	2.47E-109	2.240873439	0.255	0.002	3.31E-105	5-Mφ-Fscn1
Rgs1	2.74E-17	2.229567694	0.784	0.338	3.67E-13	5-Mφ-Fscn1
Serpinb9	3.22E-27	2.214807617	0.588	0.115	4.31E-23	5-Mφ-Fscn1
Clic4	5.96E-25	2.128454671	0.745	0.203	7.98E-21	5-Mφ-Fscn1

Nr4a3	1.26E-14	2.121088086	0.627	0.226	1.68E-10	5-Mφ-Fscn1
Marcksl1	1.91E-23	2.112062633	0.824	0.279	2.56E-19	5-Mφ-Fscn1
Anxa3	4.92E-33	2.078951546	0.51	0.071	6.58E-29	5-Mφ-Fscn1
Socs2	1.78E-60	2.071307381	0.275	0.009	2.39E-56	5-Mφ-Fscn1
Rogdi	1.17E-36	2.059110698	0.49	0.059	1.57E-32	5-Mφ-Fscn1
H2-Eb2	4.96E-70	2.003202151	0.333	0.012	6.64E-66	5-Mφ-Fscn1
Serpinb6b	1.27E-15	2.00231032	0.392	0.086	1.71E-11	5-Mφ-Fscn1
Epsti1	3.45E-10	1.996449289	0.529	0.222	4.62E-06	5-Mφ-Fscn1
Etv3	9.13E-25	1.981059593	0.49	0.084	1.22E-20	5-Mφ-Fscn1
Plxnc1	1.20E-34	1.953612803	0.392	0.039	1.61E-30	5-Mφ-Fscn1
Map4k4	3.87E-19	1.91096464	0.667	0.195	5.18E-15	5-Mφ-Fscn1
Tmem176a	1.50E-15	1.897927769	0.471	0.12	2.00E-11	5-Mφ-Fscn1
Bcl2a1b	2.33E-12	1.872762281	0.569	0.21	3.12E-08	5-Mφ-Fscn1
Zfp36l1	8.27E-15	1.861859727	0.804	0.394	1.11E-10	5-Mφ-Fscn1
Cst3	1.35E-13	1.856668136	0.922	0.848	1.81E-09	5-Mφ-Fscn1
Birc2	2.35E-21	1.829227938	0.549	0.116	3.14E-17	5-Mφ-Fscn1
Calm1	2.38E-14	1.812779558	0.843	0.585	3.19E-10	5-Mφ-Fscn1
Net1	4.60E-30	1.810253575	0.294	0.025	6.16E-26	5-Mφ-Fscn1
Swap70	3.01E-21	1.806803252	0.412	0.069	4.03E-17	5-Mφ-Fscn1
Rel	1.46E-15	1.796011914	0.588	0.173	1.95E-11	5-Mφ-Fscn1
Bcl2a1d	2.19E-15	1.775662617	0.51	0.14	2.94E-11	5-Mφ-Fscn1
H2-Eb1	1.34E-12	1.765170437	0.843	0.511	1.80E-08	5-Mφ-Fscn1
Tmem39a	4.19E-22	1.741647932	0.373	0.054	5.61E-18	5-Mφ-Fscn1
Marcks	1.56E-17	1.727377877	0.961	0.541	2.08E-13	5-Mφ-Fscn1
Cblb	6.54E-16	1.714354851	0.412	0.087	8.75E-12	5-Mφ-Fscn1
H2-Aa	4.59E-14	1.684281423	0.902	0.527	6.15E-10	5-Mφ-Fscn1
Spint2	5.92E-17	1.677456009	0.333	0.059	7.93E-13	5-Mφ-Fscn1
Zc3h12c	1.92E-18	1.648207397	0.314	0.047	2.57E-14	5-Mφ-Fscn1
Tbc1d8	1.05E-15	1.641973795	0.353	0.066	1.41E-11	5-Mφ-Fscn1
Syngr2	1.51E-16	1.63956409	0.725	0.282	2.02E-12	5-Mφ-Fscn1
Arl5a	1.99E-20	1.638316219	0.471	0.09	2.66E-16	5-Mφ-Fscn1
Ccl5	2.10E-10	1.635000023	0.863	0.616	2.81E-06	5-Mφ-Fscn1
Psme2b	1.42E-13	1.634585364	0.784	0.398	1.90E-09	5-Mφ-Fscn1
Pcgf5	2.01E-13	1.628035973	0.412	0.103	2.69E-09	5-Mφ-Fscn1
Rps27l	4.30E-09	1.517928498	0.745	0.458	5.75E-05	5-Mφ-Fscn1
Cd40	3.54E-07	1.515427579	0.275	0.08	0.004742224	5-Mφ-Fscn1
Jak2	1.86E-13	1.473308801	0.49	0.139	2.48E-09	5-Mφ-Fscn1
Gfpt1	2.01E-13	1.471370832	0.392	0.089	2.70E-09	5-Mφ-Fscn1
Hmgcs1	2.98E-11	1.46332836	0.294	0.063	3.99E-07	5-Mφ-Fscn1
Ptms	2.56E-09	1.439225242	0.471	0.176	3.42E-05	5-Mφ-Fscn1
Samsn1	7.85E-12	1.43425182	0.765	0.369	1.05E-07	5-Mφ-Fscn1
Gtf2a1	7.94E-10	1.418016381	0.333	0.091	1.06E-05	5-Mφ-Fscn1
Klf6	2.55E-08	1.41237914	0.863	0.688	0.000341089	5-Mφ-Fscn1
Gadd45b	6.63E-14	1.402418663	0.804	0.391	8.87E-10	5-Mφ-Fscn1
Csrp1	5.42E-14	1.39813579	0.255	0.041	7.25E-10	5-Mφ-Fscn1
Fam32a	5.88E-11	1.380520281	0.588	0.233	7.87E-07	5-Mφ-Fscn1
Tuba1a	1.81E-07	1.343455149	0.275	0.081	0.002427697	5-Mφ-Fscn1
Mmd	4.90E-07	1.339715379	0.255	0.073	0.006560757	5-Mφ-Fscn1
Bcl2a1a	1.67E-15	1.333172019	0.333	0.06	2.24E-11	5-Mφ-Fscn1
Fbrsl1	3.33E-12	1.331949176	0.373	0.089	4.45E-08	5-Mφ-Fscn1
Cd74	2.13E-11	1.327924705	0.98	0.823	2.85E-07	5-Mφ-Fscn1
Glipr2	8.17E-09	1.322941989	0.412	0.139	0.000109378	5-Mφ-Fscn1
Ccnd2	6.26E-11	1.319497095	0.412	0.116	8.38E-07	5-Mφ-Fscn1
Tmem176b	2.73E-08	1.309594802	0.51	0.217	0.000365389	5-Mφ-Fscn1
Ptger4	2.80E-06	1.304055185	0.333	0.123	0.037507638	5-Mφ-Fscn1
Lactb	5.80E-12	1.298585797	0.294	0.06	7.76E-08	5-Mφ-Fscn1
Bmp2k	4.07E-19	1.296020121	0.255	0.029	5.45E-15	5-Mφ-Fscn1
Psme1	2.14E-09	1.288629356	0.647	0.31	2.86E-05	5-Mφ-Fscn1

Il21r	1.44E-08	1.282518501	0.412	0.137	0.000192701	5-Mφ-Fscn1
Cbfa2t3	9.71E-07	1.250232206	0.451	0.195	0.012996641	5-Mφ-Fscn1
Ramp3	2.10E-09	1.230337239	0.255	0.057	2.81E-05	5-Mφ-Fscn1
Traf1	4.44E-10	1.216390836	0.412	0.124	5.94E-06	5-Mφ-Fscn1
Papss2	8.43E-23	1.212122247	0.255	0.025	1.13E-18	5-Mφ-Fscn1
Clec2d	4.32E-06	1.198332548	0.412	0.172	0.0578911	5-Mφ-Fscn1
Rasa2	4.57E-14	1.195654661	0.294	0.052	6.11E-10	5-Mφ-Fscn1
Aebp2	5.62E-07	1.189175106	0.373	0.135	0.00752333	5-Mφ-Fscn1
Uap1	3.19E-08	1.188386957	0.275	0.072	0.000426794	5-Mφ-Fscn1
Sav1	2.17E-07	1.177050615	0.275	0.079	0.002899471	5-Mφ-Fscn1
Pik3r1	8.20E-07	1.173241926	0.392	0.145	0.010980115	5-Mφ-Fscn1
Cd83	1.73E-09	1.167603712	0.745	0.402	2.32E-05	5-Mφ-Fscn1
Iscu	9.62E-10	1.161493991	0.451	0.156	1.29E-05	5-Mφ-Fscn1
Avpi1	1.75E-06	1.123655135	0.314	0.109	0.023371191	5-Mφ-Fscn1
Zfand6	7.15E-05	1.119265058	0.392	0.188	0.957181723	5-Mφ-Fscn1
Dpp4	4.47E-19	1.115743763	0.275	0.034	5.99E-15	5-Mφ-Fscn1
Rassf2	4.95E-07	1.11288324	0.373	0.135	0.006619487	5-Mφ-Fscn1
Rabgap1l	3.99E-11	1.108694696	0.294	0.063	5.34E-07	5-Mφ-Fscn1
Selplg	6.70E-06	1.100601549	0.667	0.425	0.089688532	5-Mφ-Fscn1
Arl5c	5.42E-08	1.095080216	0.412	0.147	0.000725803	5-Mφ-Fscn1
Dynll2	3.72E-08	1.082037264	0.275	0.073	0.00049778	5-Mφ-Fscn1
Arl4c	6.25E-05	1.068598186	0.412	0.197	0.835985864	5-Mφ-Fscn1
Txndc17	0.00084372	1.0648742	0.392	0.222	1	5-Mφ-Fscn1
Cnn2	3.75E-06	1.061638854	0.431	0.197	0.050234183	5-Mφ-Fscn1
Psmb8	5.46E-05	1.058308548	0.588	0.383	0.731103209	5-Mφ-Fscn1
D930015E(	8.23E-11	1.04936621	0.275	0.057	1.10E-06	5-Mφ-Fscn1
H2-T23	2.14E-05	1.039395863	0.549	0.313	0.287082393	5-Mφ-Fscn1
Bhlhe40	2.09E-09	1.035670541	0.647	0.273	2.80E-05	5-Mφ-Fscn1
Fam46c	0.001311951	1.029512515	0.255	0.114	1	5-Mφ-Fscn1
Ccdc88a	1.85E-05	1.022914197	0.392	0.175	0.247767488	5-Mφ-Fscn1
Cd274	1.60E-05	1.007070347	0.294	0.106	0.21451918	5-Mφ-Fscn1
Pmaip1	4.01E-06	1.003061507	0.725	0.417	0.053720211	5-Mφ-Fscn1
Itgb1	1.60E-06	0.994093711	0.431	0.179	0.021472037	5-Mφ-Fscn1
Batf3	1.09E-08	0.991727675	0.275	0.068	0.00014559	5-Mφ-Fscn1
Cflar	3.12E-06	0.977241864	0.471	0.214	0.041825597	5-Mφ-Fscn1
Lsp1	3.05E-06	0.97099999	0.745	0.553	0.040853477	5-Mφ-Fscn1
Tmsb4x	3.84E-15	0.966721872	1	0.999	5.14E-11	5-Mφ-Fscn1
Rftn1	5.45E-08	0.963760414	0.314	0.09	0.000729591	5-Mφ-Fscn1
Psmb9	4.70E-05	0.955793636	0.471	0.241	0.629147317	5-Mφ-Fscn1
Grasp	1.10E-07	0.949918258	0.255	0.065	0.001475965	5-Mφ-Fscn1
Scpep1	0.00011018	0.94367071	0.275	0.108	1	5-Mφ-Fscn1
Mbtd1	0.000196535	0.943423512	0.314	0.14	1	5-Mφ-Fscn1
H2-K1	7.25E-09	0.935099912	0.98	0.86	9.70E-05	5-Mφ-Fscn1
Ctnna1	3.24E-08	0.934558717	0.392	0.127	0.00043408	5-Mφ-Fscn1
Got1	0.001501117	0.925104471	0.333	0.167	1	5-Mφ-Fscn1
Rala	1.85E-07	0.923896756	0.373	0.127	0.002475481	5-Mφ-Fscn1
Arf4	1.48E-06	0.921769964	0.627	0.333	0.019825323	5-Mφ-Fscn1
Galnt1	9.22E-05	0.914949226	0.294	0.119	1	5-Mφ-Fscn1
Rassf3	2.37E-05	0.907668457	0.431	0.199	0.317501776	5-Mφ-Fscn1
Phf21a	4.23E-05	0.907157694	0.333	0.136	0.565689427	5-Mφ-Fscn1
Nfkb2	1.67E-07	0.906018068	0.314	0.094	0.002235974	5-Mφ-Fscn1
Fnbp1	0.001198785	0.898442228	0.333	0.174	1	5-Mφ-Fscn1
Ube2z	1.01E-06	0.896713914	0.255	0.072	0.013478696	5-Mφ-Fscn1
H2-Ab1	0.002134611	0.890267367	0.745	0.638	1	5-Mφ-Fscn1
Rnf115	0.000172857	0.888051036	0.333	0.149	1	5-Mφ-Fscn1
Dnm1l	2.53E-12	0.88675852	0.275	0.05	3.38E-08	5-Mφ-Fscn1
Tubb5	0.000101205	0.88554983	0.529	0.302	1	5-Mφ-Fscn1
H2-Q4	0.000276178	0.843218709	0.353	0.172	1	5-Mφ-Fscn1

Ktn1	8.16E-09	0.834315413	0.255	0.058	0.000109246	5-Mφ-Fscn1
Wnk1	0.001069475	0.83357897	0.412	0.221	1	5-Mφ-Fscn1
Hspa1a	0.000211891	0.830617451	0.804	0.578	1	5-Mφ-Fscn1
Prex1	0.000251643	0.824106899	0.373	0.182	1	5-Mφ-Fscn1
P2ry10	0.003672872	0.814651776	0.373	0.213	1	5-Mφ-Fscn1
Nfkbib	0.001469677	0.813197455	0.255	0.116	1	5-Mφ-Fscn1
Gramd3	0.000264186	0.805755644	0.353	0.161	1	5-Mφ-Fscn1
Gnb1	0.00095336	0.800914771	0.549	0.379	1	5-Mφ-Fscn1
Apobec3	0.000191996	0.794107357	0.353	0.162	1	5-Mφ-Fscn1
Il7r	0.001947492	0.789446925	0.275	0.128	1	5-Mφ-Fscn1
Ifi30	0.001148783	0.780759504	0.588	0.402	1	5-Mφ-Fscn1
B2m	5.40E-10	0.751637348	1	0.96	7.23E-06	5-Mφ-Fscn1
Kmt2a	0.000470645	0.748453928	0.255	0.103	1	5-Mφ-Fscn1
Pfkfb3	6.17E-06	0.747232389	0.255	0.079	0.082619524	5-Mφ-Fscn1
Adam8	0.001200263	0.744255766	0.275	0.125	1	5-Mφ-Fscn1
Ptprs	1.55E-05	0.738916618	0.255	0.083	0.20740696	5-Mφ-Fscn1
Rab8b	0.003368632	0.738274264	0.529	0.358	1	5-Mφ-Fscn1
Skil	8.26E-05	0.725844699	0.49	0.254	1	5-Mφ-Fscn1
Dapp1	0.000512086	0.72468004	0.314	0.143	1	5-Mφ-Fscn1
Rap2a	2.92E-05	0.722348935	0.255	0.086	0.390832366	5-Mφ-Fscn1
Rnf19b	0.001482284	0.713732733	0.333	0.168	1	5-Mφ-Fscn1
Rab21	0.000503502	0.711762085	0.255	0.105	1	5-Mφ-Fscn1
Hn1	0.004692238	0.710783641	0.471	0.313	1	5-Mφ-Fscn1
Fam168b	0.004893749	0.710056278	0.294	0.153	1	5-Mφ-Fscn1
Sumo2	0.000518616	0.709441681	0.471	0.268	1	5-Mφ-Fscn1
Hivep2	1.16E-05	0.706811013	0.333	0.123	0.154901714	5-Mφ-Fscn1
Rab8a	0.001023795	0.693922764	0.314	0.15	1	5-Mφ-Fscn1
Myo1g	0.000972992	0.689659592	0.294	0.134	1	5-Mφ-Fscn1
Rtn4	0.000653441	0.687284069	0.549	0.335	1	5-Mφ-Fscn1
Tob2	0.004728531	0.684989355	0.412	0.233	1	5-Mφ-Fscn1
Eif4b	0.006846487	0.682951639	0.333	0.191	1	5-Mφ-Fscn1
Hsp90ab1	1.24E-05	0.677472033	0.922	0.754	0.166491378	5-Mφ-Fscn1
Nrip1	0.008720134	0.668579365	0.275	0.147	1	5-Mφ-Fscn1
Arpp19	0.006228274	0.658453629	0.51	0.33	1	5-Mφ-Fscn1
Selk	0.000502647	0.653609676	0.706	0.464	1	5-Mφ-Fscn1
Ogfrl1	0.002764779	0.651118783	0.333	0.175	1	5-Mφ-Fscn1
Cd24a	0.000758941	0.648290656	0.333	0.16	1	5-Mφ-Fscn1
Rps19	8.80E-08	0.647790469	1	0.925	0.001177719	5-Mφ-Fscn1
Ddx6	0.000200033	0.64447576	0.647	0.404	1	5-Mφ-Fscn1
H2-D1	4.34E-06	0.635678241	0.902	0.841	0.058057128	5-Mφ-Fscn1
Ostf1	0.007153047	0.635579829	0.49	0.341	1	5-Mφ-Fscn1
Actb	3.99E-08	0.628463447	1	0.999	0.000533818	5-Mφ-Fscn1
Edf1	0.005990913	0.627278969	0.373	0.23	1	5-Mφ-Fscn1
Tagln2	0.001635726	0.623534943	0.627	0.452	1	5-Mφ-Fscn1
Odc1	0.006241969	0.620222229	0.373	0.217	1	5-Mφ-Fscn1
Suco	0.001733721	0.61193802	0.255	0.111	1	5-Mφ-Fscn1
Edem1	0.004053783	0.611555964	0.412	0.241	1	5-Mφ-Fscn1
Pbxip1	0.001084765	0.610761274	0.294	0.135	1	5-Mφ-Fscn1
Maff	0.000492247	0.603814967	0.255	0.103	1	5-Mφ-Fscn1
Dip2b	6.55E-05	0.601388417	0.255	0.088	0.876424212	5-Mφ-Fscn1
Arrb2	0.003839408	0.596688783	0.314	0.166	1	5-Mφ-Fscn1
H2afz	0.002470859	0.594217458	0.824	0.769	1	5-Mφ-Fscn1
Nampt	0.007245047	0.590954414	0.373	0.222	1	5-Mφ-Fscn1
Ssu72	0.00733889	0.588411417	0.333	0.187	1	5-Mφ-Fscn1
Birc3	0.001185001	0.583925618	0.471	0.257	1	5-Mφ-Fscn1
Tap1	0.000511707	0.583147312	0.392	0.194	1	5-Mφ-Fscn1
2700060E(	0.004391349	0.578477855	0.431	0.275	1	5-Mφ-Fscn1
Cggbp1	0.001078649	0.57730049	0.412	0.216	1	5-Mφ-Fscn1

Herpud1	0.008690157	0.568287757	0.49	0.327	1	5-Mφ-Fscn1
Gpbp1	0.000883349	0.567511716	0.431	0.227	1	5-Mφ-Fscn1
Rad21	0.000746507	0.558229823	0.353	0.166	1	5-Mφ-Fscn1
Actg1	2.19E-06	0.55242176	1	0.971	0.029330298	5-Mφ-Fscn1
Mt-mt-Rnr2	0.000656722	0.537019818	0.961	0.853	1	5-Mφ-Fscn1
Ak2	0.005949964	0.533920196	0.275	0.142	1	5-Mφ-Fscn1
Vamp8	0.005484874	0.533012316	0.588	0.406	1	5-Mφ-Fscn1
Zfp207	0.004635594	0.514111156	0.294	0.151	1	5-Mφ-Fscn1

Supplementary Table 6

gene	p_val	avg_log2FC	pct.1	pct.2	p_val_adj
LOC100862446	0	2.599497027	1	0.609	0
Ftl1	0	2.459398188	0.998	0.669	0
Jchain	4.04E-301	2.270290551	0.992	0.204	5.41E-297
Lyz2	1.01E-216	2.093109489	1	0.799	1.35E-212
Rps5	1.15E-218	1.736189983	0.988	0.38	1.54E-214
Sh3bgrl3	1.78E-244	1.686811242	0.994	0.512	2.39E-240
Apoe	2.66E-152	1.681044346	0.966	0.378	3.55E-148
Ifitm2	4.08E-239	1.627043248	0.996	0.593	5.46E-235
Lyz1	6.90E-246	1.610680497	0.688	0.046	9.23E-242
Rps2	2.84E-183	1.546407216	0.986	0.404	3.80E-179
Lgals1	3.09E-181	1.513841723	0.955	0.3	4.14E-177
Ubb	1.02E-254	1.501839748	1	0.714	1.37E-250
Mzb1	7.67E-273	1.482385152	0.721	0.046	1.03E-268
Ppib	9.69E-250	1.470930488	0.887	0.156	1.30E-245
Eno1	2.01E-236	1.396560933	0.814	0.128	2.70E-232
Lgals3	1.44E-167	1.367962029	0.988	0.635	1.93E-163
Rplp0	1.08E-238	1.35201253	1	0.777	1.44E-234
LOC105244208	1.80E-219	1.348781675	1	0.719	2.42E-215
Elane	1.98E-228	1.339519833	0.569	0.017	2.66E-224
Fcer1g	2.48E-217	1.333227395	1	0.779	3.31E-213
Manf	6.32E-230	1.326208367	0.789	0.123	8.46E-226
Rps6	2.46E-208	1.323127146	0.907	0.223	3.30E-204
Prtn3	4.29E-233	1.320929651	0.601	0.032	5.75E-229
Ngp	1.77E-246	1.296073657	0.921	0.196	2.37E-242
Rpl14	3.79E-203	1.279095946	1	0.712	5.07E-199
Ccl5	1.19E-166	1.265402386	1	0.544	1.59E-162
Eno1b	2.02E-196	1.232244751	0.514	0.02	2.70E-192
Gm13202	2.55E-220	1.228700443	0.862	0.186	3.42E-216
D8Ertd738e	1.14E-204	1.221605277	0.943	0.266	1.52E-200
Brk1	2.19E-265	1.185076462	0.846	0.141	2.92E-261
Alox5ap	1.16E-136	1.182539129	0.99	0.561	1.56E-132
Ly6c2	4.59E-146	1.175198055	0.955	0.374	6.14E-142
Ccl9	1.70E-145	1.15623019	0.881	0.297	2.28E-141
Camp	1.98E-243	1.151343196	0.737	0.098	2.65E-239
Emd	8.25E-149	1.127202305	0.953	0.366	1.10E-144
Ly6d	1.55E-261	1.124449596	0.868	0.163	2.08E-257
Uba52	2.40E-206	1.121281063	1	0.911	3.21E-202
Rpl29	1.03E-133	1.117698819	0.988	0.537	1.39E-129
15-Sep	9.18E-234	1.110740511	0.704	0.092	1.23E-229
Ppp1ca	7.69E-254	1.10137157	0.897	0.197	1.03E-249
Gm6745	1.91E-208	1.100395359	0.579	0.045	2.55E-204
Tspo	1.42E-130	1.097324994	0.992	0.526	1.90E-126
Psmb3	3.78E-188	1.090293199	0.532	0.037	5.07E-184
Cnbp	7.35E-250	1.085282051	0.866	0.179	9.84E-246
S100a10	2.86E-123	1.068159832	0.964	0.447	3.83E-119
Aldoa	5.00E-221	1.067291159	0.826	0.178	6.69E-217
Btf3	4.70E-127	1.063654458	0.984	0.493	6.29E-123
Atp5c1	7.63E-225	1.062965386	0.65	0.075	1.02E-220
Cd68	2.36E-200	1.054826929	0.646	0.09	3.16E-196
Ifitm1	2.76E-196	1.04684184	0.957	0.305	3.69E-192
Gpx4	1.07E-214	1.041333419	0.856	0.208	1.44E-210
Slpi	1.89E-176	1.037361931	0.939	0.307	2.53E-172
Atp5d	1.57E-252	1.033372641	0.897	0.205	2.10E-248
Psmb2	5.72E-225	1.026899704	0.706	0.108	7.66E-221
Ctsz	4.50E-175	1.023460219	0.972	0.359	6.03E-171

Akr1a1	3.69E-229	1.01631414	0.729	0.117	4.94E-225
Cstb	1.01E-175	1.016059798	0.901	0.291	1.35E-171
Cyba	7.17E-124	1.009971292	0.998	0.715	9.60E-120
Id3	5.53E-93	1.009050405	0.634	0.211	7.40E-89
Sdf2l1	1.50E-181	0.993960405	0.569	0.07	2.00E-177
Ppia	1.00E-129	0.987885541	1	0.724	1.34E-125
Cd52	8.21E-121	0.986216052	0.998	0.734	1.10E-116
Edf1	4.77E-236	0.984380151	0.753	0.129	6.38E-232
Atp6v1f	3.96E-204	0.983365809	0.901	0.258	5.30E-200
Msrb1	3.11E-110	0.981934597	0.992	0.579	4.16E-106
Pfn1	2.03E-120	0.979388833	0.996	0.728	2.72E-116
LOC102635661	1.08E-169	0.978098829	0.945	0.34	1.45E-165
Myl12b	3.25E-221	0.971470979	0.818	0.184	4.35E-217
Prdx1	2.44E-145	0.961676473	0.976	0.421	3.27E-141
Arpc1b	7.28E-135	0.959343558	0.982	0.451	9.75E-131
Eif5a	1.36E-178	0.958506422	0.915	0.3	1.82E-174
Ssr2	1.94E-182	0.954400044	0.536	0.052	2.59E-178
Rpl15	1.26E-128	0.952375751	1	0.761	1.68E-124
Arpc3	3.91E-152	0.951373827	0.958	0.382	5.24E-148
Arhgdib	2.15E-121	0.949896717	0.978	0.478	2.87E-117
Sepw1	1.47E-173	0.936838436	0.937	0.329	1.96E-169
Park7	4.51E-221	0.93572933	0.739	0.14	6.04E-217
Cdk2ap2	2.12E-171	0.929717806	0.903	0.302	2.84E-167
Nedd8	2.62E-229	0.921438237	0.862	0.214	3.51E-225
Card19	3.97E-192	0.918016678	0.615	0.092	5.31E-188
Capg	5.70E-181	0.916814979	0.609	0.096	7.63E-177
Sumo2	3.70E-225	0.913504661	0.789	0.169	4.95E-221
Gabarap	3.96E-158	0.912306082	0.972	0.396	5.30E-154
Rpl19	5.32E-124	0.912192063	0.996	0.77	7.11E-120
Vamp8	4.32E-204	0.898561237	0.941	0.304	5.78E-200
Ssr4	2.15E-208	0.888444283	0.676	0.119	2.88E-204
Cox5a	3.38E-236	0.88344277	0.789	0.161	4.53E-232
Fis1	8.65E-213	0.882934867	0.858	0.229	1.16E-208
Rps10	8.43E-172	0.877633251	0.937	0.342	1.13E-167
Myl12a	8.20E-175	0.873188389	0.905	0.311	1.10E-170
Ctsh	8.78E-199	0.869466335	0.901	0.282	1.17E-194
Stmn1	7.39E-190	0.867575291	0.581	0.079	9.90E-186
Rac2	4.24E-194	0.864855988	0.907	0.289	5.67E-190
Supt4b	2.94E-186	0.860637085	0.549	0.068	3.93E-182
Emp3	2.31E-181	0.857094338	0.879	0.282	3.09E-177
Erh	8.90E-218	0.853123702	0.806	0.192	1.19E-213
Grpel1	3.80E-156	0.852396199	0.441	0.032	5.09E-152
Nme2	2.97E-130	0.850903323	0.953	0.42	3.97E-126
Mien1	6.95E-204	0.849381857	0.619	0.092	9.30E-200
Gm25380	3.81E-105	0.845016831	1	0.682	5.09E-101
Myl6	1.05E-123	0.842028987	0.962	0.448	1.40E-119
Ostf1	1.88E-209	0.838543977	0.862	0.241	2.52E-205
H2-Ab1	2.89E-104	0.837273934	0.994	0.57	3.87E-100
Ifitm3	2.16E-82	0.832595251	0.998	0.866	2.89E-78
Ldha	4.45E-199	0.832125083	0.806	0.212	5.96E-195
Fth1	3.51E-96	0.832119035	1	0.971	4.70E-92
Lrrc58	4.75E-199	0.831124896	0.794	0.203	6.36E-195
Spcs1	1.09E-240	0.827490228	0.781	0.164	1.46E-236
Psmb8	2.14E-200	0.827288239	0.901	0.284	2.86E-196
Ramp1	2.47E-214	0.822941888	0.623	0.087	3.31E-210
Napsa	3.32E-213	0.819088865	0.816	0.211	4.44E-209
Lamtor5	8.84E-213	0.815875838	0.642	0.104	1.18E-208
Dad1	1.96E-231	0.814981937	0.86	0.225	2.63E-227

Taldo1	1.17E-178	0.814090739	0.919	0.322	1.57E-174
Tnfaip8l2	4.17E-156	0.81301955	0.443	0.039	5.59E-152
Tyrobp	2.33E-107	0.812338049	0.994	0.883	3.11E-103
Fam96a	3.44E-214	0.810893401	0.757	0.171	4.60E-210
Rps18	1.81E-93	0.804612954	0.994	0.769	2.43E-89
Hint1	1.43E-137	0.799147332	0.953	0.415	1.92E-133
Tppp3	3.60E-113	0.797556796	0.445	0.081	4.82E-109
I830127L07Rik	2.05E-151	0.797033339	0.534	0.096	2.75E-147
Bax	7.12E-215	0.795110217	0.765	0.175	9.54E-211
Eif4ebp1	4.30E-184	0.79235693	0.524	0.062	5.75E-180
Nt5c	5.75E-203	0.790141837	0.607	0.088	7.69E-199
Uqcrfs1	4.37E-205	0.788243927	0.749	0.177	5.86E-201
Oaz1	2.17E-96	0.787209415	1	0.7	2.91E-92
Ifi30	4.86E-177	0.786098702	0.895	0.307	6.50E-173
Ndufs4	9.51E-183	0.785763912	0.528	0.065	1.27E-178
Chil3	2.99E-119	0.784797608	0.818	0.329	4.00E-115
Higd2a	2.26E-206	0.783439036	0.656	0.12	3.03E-202
Hilpda	1.86E-148	0.781630269	0.565	0.116	2.49E-144
Dynlrb1	2.35E-232	0.781037296	0.759	0.163	3.15E-228
Tmem59	8.15E-209	0.778289231	0.694	0.14	1.09E-204
Hp	3.88E-105	0.777634018	0.955	0.483	5.19E-101
Cycs	2.72E-167	0.774805343	0.907	0.335	3.64E-163
Sra1	3.46E-151	0.772228282	0.457	0.05	4.63E-147
Cd74	2.45E-56	0.771756616	1	0.791	3.28E-52
Sys1	9.79E-191	0.770415357	0.569	0.083	1.31E-186
Rgs10	1.20E-213	0.768389524	0.704	0.141	1.61E-209
Vim	1.73E-112	0.766855596	0.955	0.474	2.32E-108
Cox6b1	1.23E-155	0.765284435	0.941	0.382	1.65E-151
Slc25a5	2.11E-147	0.7613306	0.937	0.389	2.83E-143
Polr1d	1.11E-221	0.758040368	0.838	0.224	1.49E-217
Spcs2	1.06E-230	0.757980252	0.775	0.176	1.42E-226
Gm5621	6.95E-229	0.755502102	0.789	0.188	9.30E-225
Rnasek	1.39E-200	0.754612798	0.721	0.167	1.86E-196
Tmem176b	4.61E-137	0.75252091	0.593	0.148	6.18E-133
Pfdn5	4.78E-121	0.752077935	0.98	0.492	6.40E-117
Gpx1	2.94E-82	0.748935898	0.994	0.743	3.94E-78
Rnh1	2.24E-198	0.747785209	0.662	0.133	3.00E-194
Pgls	2.11E-189	0.744858289	0.621	0.114	2.82E-185
Gngt2	4.19E-140	0.744847853	0.899	0.367	5.62E-136
H2-D1	7.79E-87	0.743930798	0.998	0.811	1.04E-82
Sri	2.44E-221	0.742670562	0.694	0.135	3.26E-217
Eif3h	9.04E-188	0.742043202	0.87	0.28	1.21E-183
Rps11	1.97E-81	0.739007068	0.992	0.749	2.64E-77
Tmem134	5.72E-172	0.738150269	0.488	0.058	7.66E-168
Rpl32	4.44E-89	0.738130829	0.994	0.882	5.94E-85
Med28	4.32E-195	0.737951147	0.573	0.082	5.79E-191
Bloc1s2	2.06E-169	0.735343176	0.52	0.074	2.75E-165
Rnase6	6.99E-179	0.73507574	0.561	0.086	9.35E-175
H3f3c	4.74E-164	0.734340275	0.486	0.063	6.35E-160
Coa3	1.19E-166	0.73409276	0.484	0.056	1.59E-162
Prelid1	3.05E-223	0.731371861	0.708	0.141	4.09E-219
Ctss	3.90E-81	0.730979105	0.998	0.714	5.22E-77
Ninj1	2.12E-171	0.727392863	0.682	0.17	2.83E-167
Clic1	9.23E-196	0.725922653	0.915	0.314	1.24E-191
Ccdc12	5.82E-223	0.725810048	0.834	0.225	7.79E-219
Ufc1	6.68E-161	0.724967985	0.476	0.06	8.95E-157
Reep5	1.32E-202	0.723612994	0.719	0.17	1.77E-198
Serf2	2.48E-87	0.721794301	0.996	0.762	3.32E-83

Itm2b	3.53E-77	0.72076888	0.984	0.648	4.72E-73
Cmtm7	6.24E-220	0.720597149	0.761	0.174	8.36E-216
Lrg1	1.43E-153	0.719675051	0.53	0.094	1.92E-149
Tmem176a	3.63E-114	0.716577536	0.415	0.068	4.86E-110
Eif1	2.13E-84	0.712624041	0.998	0.813	2.85E-80
Ly6a	3.01E-152	0.712553003	0.496	0.072	4.03E-148
Nupr1	4.16E-175	0.711143702	0.632	0.138	5.57E-171
Ear2	1.00E-68	0.706274589	0.261	0.033	1.34E-64
Hmgn2	2.09E-200	0.705965339	0.611	0.107	2.80E-196
BC028528	1.54E-139	0.705177781	0.46	0.071	2.06E-135
Stra13	2.13E-148	0.704964987	0.445	0.054	2.85E-144
H2-DMa	5.85E-166	0.704693231	0.783	0.247	7.83E-162
Clta	8.57E-134	0.703416003	0.951	0.434	1.15E-129
Fabp5	2.60E-81	0.703177279	0.366	0.083	3.47E-77
Ccl6	3.74E-97	0.698999128	0.978	0.55	5.01E-93
Rps14	6.34E-79	0.697561357	0.994	0.783	8.49E-75
Psmb5	3.93E-230	0.696710655	0.753	0.169	5.26E-226
Timm23	3.16E-165	0.69558257	0.512	0.077	4.22E-161
Swi5	4.55E-200	0.689490585	0.632	0.121	6.09E-196
Nhp2	7.10E-147	0.689298401	0.453	0.058	9.50E-143
Ypel3	4.86E-208	0.683584704	0.666	0.134	6.50E-204
Eef1d	4.23E-209	0.683017334	0.652	0.13	5.66E-205
Commd1	1.67E-139	0.682568593	0.409	0.044	2.24E-135
H2-Aa	6.17E-134	0.682312114	0.941	0.453	8.27E-130
Mydgf	1.56E-154	0.6786471	0.453	0.055	2.09E-150
Sdhb	5.22E-172	0.676949491	0.492	0.064	6.98E-168
Gng10	4.14E-201	0.676057605	0.852	0.267	5.54E-197
Psenen	4.73E-204	0.675631344	0.66	0.137	6.33E-200
Supt4a	3.00E-228	0.67406318	0.856	0.246	4.02E-224
Cfl1	4.18E-78	0.67291483	0.996	0.675	5.59E-74
Prdx2	1.77E-225	0.672608935	0.771	0.192	2.37E-221
Timm13	2.55E-222	0.665251531	0.858	0.255	3.42E-218
Tmsb4x	3.52E-114	0.664163018	1	0.998	4.71E-110
Pkig	7.79E-170	0.664050085	0.51	0.072	1.04E-165
Birc5	3.01E-106	0.663005709	0.314	0.022	4.03E-102
Rpl13	1.74E-96	0.662423937	1	0.96	2.32E-92
Dctn3	1.12E-189	0.661036898	0.617	0.121	1.50E-185
Rpl18a	1.14E-197	0.656386317	0.709	0.176	1.52E-193
Cox7a2l	1.56E-148	0.655325343	0.925	0.389	2.09E-144
Vdac3	1.38E-153	0.654603482	0.474	0.07	1.85E-149
B2m	1.19E-79	0.651737472	1	0.953	1.60E-75
Iscu	4.43E-176	0.650905287	0.534	0.087	5.92E-172
Ndufs8	8.13E-175	0.650812628	0.524	0.08	1.09E-170
Ube2s	2.32E-221	0.650571093	0.767	0.191	3.11E-217
Fxyd5	1.16E-80	0.65051288	0.97	0.583	1.56E-76
Rpl6	1.08E-71	0.650029616	0.992	0.716	1.45E-67
Pycard	1.64E-186	0.649569082	0.84	0.276	2.19E-182
Gm8210	1.19E-95	0.648813646	0.257	0.006	1.59E-91
Sumo1	1.07E-240	0.648228424	0.745	0.163	1.43E-236
Ptprcap	3.52E-174	0.646843837	0.549	0.092	4.71E-170
Ndufa12	2.25E-156	0.646689014	0.443	0.049	3.01E-152
Ap2s1	6.51E-210	0.645018916	0.696	0.155	8.72E-206
2410015M20Rik	7.92E-193	0.644674555	0.615	0.118	1.06E-188
Cst3	8.28E-70	0.644475134	0.998	0.819	1.11E-65
Rnaset2b	1.22E-186	0.641790708	0.621	0.128	1.64E-182
Banf1	3.50E-182	0.63984322	0.532	0.083	4.68E-178
Ms4a4c	1.26E-164	0.639746199	0.804	0.274	1.68E-160
Mdh2	4.58E-188	0.638497871	0.623	0.127	6.13E-184

Atp5j	1.12E-139	0.638433184	0.439	0.06	1.50E-135
Ifitm6	4.34E-178	0.637153146	0.753	0.226	5.80E-174
Ndufv2	3.00E-116	0.636271675	0.332	0.026	4.01E-112
Ppdpf	3.87E-157	0.636078035	0.472	0.065	5.18E-153
Mpc1	6.38E-210	0.635826715	0.719	0.168	8.54E-206
Mrps14	5.66E-246	0.635375569	0.773	0.177	7.58E-242
Elof1	2.65E-113	0.635215076	0.32	0.023	3.55E-109
Eif3f	8.71E-144	0.633850355	0.957	0.434	1.17E-139
Mrpl12	1.20E-141	0.632041322	0.419	0.049	1.61E-137
Sap18b	9.62E-222	0.631227742	0.708	0.159	1.29E-217
Snx3	3.88E-200	0.629765897	0.642	0.134	5.19E-196
Spp1	4.81E-56	0.627736914	0.229	0.034	6.43E-52
1110008F13Rik	4.26E-228	0.625868324	0.725	0.165	5.70E-224
Atp5f1	6.86E-196	0.625814902	0.856	0.288	9.19E-192
Hsd17b10	8.94E-124	0.621949584	0.37	0.042	1.20E-119
Cope	2.67E-113	0.620738161	0.34	0.031	3.57E-109
Anapc11	2.40E-162	0.620161134	0.48	0.069	3.22E-158
Ybx1	1.64E-217	0.619908724	0.842	0.259	2.20E-213
Saa3	3.00E-90	0.618036104	0.423	0.094	4.02E-86
Pold4	9.43E-158	0.616641221	0.484	0.074	1.26E-153
Tceb2	4.67E-221	0.616027881	0.8	0.219	6.26E-217
Ccdc124	4.38E-159	0.615161134	0.455	0.06	5.86E-155
Eif6	2.05E-195	0.614418107	0.591	0.109	2.75E-191
Cdkn2d	4.89E-168	0.613283482	0.557	0.112	6.54E-164
BC031181	8.17E-185	0.61016371	0.561	0.103	1.09E-180
Psmd8	3.49E-202	0.609973114	0.636	0.129	4.67E-198
Pkm	3.70E-159	0.609893564	0.923	0.385	4.96E-155
Gnas	2.14E-225	0.609393208	0.838	0.247	2.87E-221
Selk	4.89E-175	0.609151077	0.935	0.375	6.54E-171
Gpsm3	1.28E-202	0.608752448	0.678	0.159	1.71E-198
Atp6v0e	2.19E-156	0.607742774	0.895	0.365	2.94E-152
Ndufs3	1.06E-172	0.607292411	0.472	0.059	1.42E-168
Krtcap2	1.56E-179	0.6060292	0.607	0.132	2.09E-175
Cyb5a	1.13E-145	0.605785704	0.455	0.069	1.52E-141
Sec11c	6.78E-190	0.605227977	0.715	0.195	9.08E-186
Mrpl20	8.19E-168	0.604673717	0.532	0.1	1.10E-163
Ran	6.72E-217	0.604061725	0.836	0.253	9.00E-213
Phf5a	1.54E-174	0.603746422	0.532	0.092	2.07E-170
Rpl12	2.42E-93	0.60365876	0.273	0.016	3.25E-89
H2afx	7.66E-106	0.602877021	0.3	0.02	1.03E-101
Cd48	4.49E-204	0.602658702	0.67	0.149	6.01E-200
Ctsg	2.33E-92	0.600855113	0.241	0.004	3.12E-88
C1qb	6.79E-37	0.599408268	0.158	0.022	9.09E-33
1110008P14Rik	6.27E-145	0.598655541	0.417	0.051	8.39E-141
LOC100862433	7.52E-72	0.597347404	0.998	0.868	1.01E-67
Rpl13a	1.68E-211	0.596560999	0.727	0.179	2.25E-207
Atp5b	2.65E-200	0.596328452	0.881	0.305	3.54E-196
Trem3	3.58E-102	0.595921437	0.348	0.051	4.80E-98
Csnk2b	6.25E-171	0.593763857	0.538	0.097	8.37E-167
Rpl18	7.52E-64	0.593424143	0.99	0.721	1.01E-59
Rpl21	4.30E-84	0.593313032	0.976	0.587	5.76E-80
Nutf2	3.71E-151	0.592822713	0.443	0.063	4.96E-147
Dnajc15	2.67E-177	0.592239577	0.526	0.086	3.57E-173
Tpgs1	7.13E-127	0.591247076	0.358	0.035	9.54E-123
Susd3	2.47E-122	0.59027558	0.381	0.049	3.31E-118
Trappc6a	2.90E-126	0.590112539	0.381	0.046	3.88E-122
Bmyc	4.20E-90	0.588253482	0.283	0.026	5.62E-86
Bloc1s1	2.79E-145	0.588126565	0.457	0.073	3.73E-141

Gm4705	6.37E-204	0.586817283	0.862	0.295	8.53E-200
Dynlt1f	3.10E-118	0.586454316	0.36	0.04	4.15E-114
Tpi1	4.51E-146	0.585962466	0.514	0.104	6.03E-142
Cd7	1.81E-119	0.585154973	0.419	0.076	2.42E-115
2700060E02Rik	7.65E-198	0.584307427	0.721	0.19	1.02E-193
Mgst1	2.43E-136	0.584119316	0.767	0.285	3.25E-132
Ssna1	6.65E-148	0.58408737	0.435	0.06	8.90E-144
2700094K13Rik	1.82E-127	0.583518205	0.403	0.057	2.44E-123
Hprt	1.58E-197	0.583170369	0.603	0.122	2.11E-193
Yif1b	6.45E-94	0.582956456	0.277	0.02	8.64E-90
Rab4b	6.46E-136	0.582811085	0.399	0.049	8.65E-132
Lamtor4	1.48E-198	0.58223685	0.794	0.239	1.98E-194
Rpl17	4.06E-64	0.581605862	0.996	0.825	5.44E-60
Gm21188	3.69E-170	0.581279152	0.698	0.206	4.94E-166
Clec4n	1.03E-73	0.57982584	0.292	0.052	1.38E-69
Tmsb10	1.03E-60	0.579792284	1	0.976	1.38E-56
Emc6	2.89E-174	0.579475966	0.54	0.101	3.86E-170
Ndufb8	3.11E-189	0.579232549	0.557	0.098	4.16E-185
Aurkaip1	1.15E-168	0.578324821	0.52	0.096	1.54E-164
Tagln2	1.13E-167	0.578125234	0.909	0.365	1.51E-163
Rps3	3.14E-68	0.577575064	1	0.826	4.21E-64
Zmat5	7.01E-108	0.576761628	0.31	0.027	9.39E-104
Npc2	1.39E-69	0.576650505	0.992	0.688	1.86E-65
Snapc5	7.30E-118	0.575453213	0.362	0.043	9.77E-114
Blvrb	6.63E-108	0.574479289	0.318	0.029	8.88E-104
Gng5	4.83E-160	0.574316036	0.455	0.064	6.46E-156
Ethe1	3.82E-131	0.570792274	0.387	0.049	5.11E-127
Limd2	1.14E-194	0.570225074	0.636	0.141	1.53E-190
Hcfc1r1	7.05E-136	0.568710126	0.389	0.045	9.44E-132
S100a1	3.14E-101	0.568361129	0.31	0.032	4.20E-97
Ssu72	1.24E-177	0.566534959	0.567	0.114	1.66E-173
Atraid	2.47E-117	0.563321197	0.358	0.043	3.31E-113
Ict1	7.18E-144	0.561794442	0.423	0.059	9.61E-140
Tmed9	2.07E-195	0.561438816	0.674	0.165	2.77E-191
Srp14	6.78E-230	0.560589649	0.83	0.245	9.07E-226
Lsm3	5.29E-125	0.559997896	0.397	0.06	7.07E-121
Atp6v0b	2.58E-201	0.559708325	0.897	0.322	3.45E-197
Mrps12	1.32E-127	0.558729917	0.383	0.05	1.77E-123
Ranbp1	7.57E-137	0.555124915	0.441	0.074	1.01E-132
Pglyrp1	9.67E-174	0.554602387	0.577	0.119	1.29E-169
Psma2	2.70E-226	0.553931552	0.783	0.212	3.62E-222
Rpl8	9.81E-65	0.552520856	1	0.835	1.31E-60
Gadd45g	3.80E-128	0.552091425	0.512	0.122	5.09E-124
Txn2	9.61E-174	0.551627486	0.486	0.072	1.29E-169
Rack1	5.07E-124	0.551446691	0.379	0.051	6.79E-120
Khk	8.23E-121	0.550971542	0.362	0.045	1.10E-116
Ms4a6d	8.90E-177	0.550014955	0.567	0.119	1.19E-172
Arpc5l	1.29E-163	0.548552102	0.512	0.096	1.73E-159
Bag1	5.34E-142	0.547417986	0.435	0.069	7.15E-138
Vps29	3.73E-154	0.547377796	0.447	0.068	4.99E-150
Id1	4.94E-89	0.546097516	0.328	0.059	6.62E-85
Atp6v1e1	7.93E-179	0.545818415	0.587	0.129	1.06E-174
Bri3	7.56E-199	0.544220685	0.787	0.241	1.01E-194
Rer1	1.35E-165	0.544082216	0.518	0.098	1.80E-161
Bcap31	1.51E-169	0.543779547	0.498	0.083	2.02E-165
Cks1b	7.16E-93	0.543218854	0.277	0.022	9.58E-89
Retnlg	3.67E-83	0.542033278	0.994	0.657	4.91E-79
Uqcc2	3.62E-133	0.540908114	0.423	0.071	4.85E-129

Mrps16	4.85E-128	0.539882239	0.393	0.056	6.50E-124
Fcgr3	1.61E-193	0.537534888	0.755	0.219	2.16E-189
Mrpl18	1.32E-135	0.537381673	0.417	0.063	1.77E-131
Lsm4	9.44E-212	0.536635366	0.709	0.177	1.26E-207
Psma4	2.11E-155	0.536212775	0.484	0.085	2.83E-151
Cyc1	5.64E-140	0.534599621	0.409	0.057	7.54E-136
Znhit1	6.30E-170	0.534091146	0.506	0.09	8.44E-166
Glipr1	7.77E-112	0.533093518	0.375	0.063	1.04E-107
S100a11	1.81E-65	0.532495166	0.978	0.654	2.42E-61
Rpl7	4.81E-61	0.532426497	0.988	0.712	6.44E-57
Atp6v1g1	2.87E-171	0.532331001	0.917	0.377	3.84E-167
Psma6	1.05E-184	0.531427775	0.555	0.108	1.40E-180
Snrpd2	1.54E-188	0.530952823	0.638	0.152	2.07E-184
Trappc1	3.32E-113	0.530095822	0.344	0.043	4.44E-109
Pomp	2.85E-184	0.53001756	0.929	0.374	3.82E-180
Cd63	1.10E-31	0.529847296	0.123	0.012	1.47E-27
Ier2	3.12E-154	0.529585155	0.941	0.424	4.18E-150
Lrrc25	2.11E-148	0.528494958	0.417	0.056	2.83E-144
Psma3	2.08E-144	0.528087084	0.437	0.07	2.78E-140
Pfdn1	4.70E-113	0.527479331	0.342	0.043	6.29E-109
Trem2	1.99E-96	0.525406986	0.298	0.034	2.67E-92
Ech1	5.26E-85	0.525269312	0.271	0.027	7.04E-81
Uqcrc1	6.82E-154	0.524288923	0.47	0.077	9.12E-150
Pebp1	7.24E-163	0.521839645	0.5	0.09	9.70E-159
Prr13	2.64E-188	0.520607401	0.881	0.33	3.53E-184
Ccdc109b	1.04E-152	0.520296665	0.462	0.08	1.39E-148
Gm9733	9.99E-129	0.519884728	0.565	0.163	1.34E-124
Rnaset2a	3.27E-221	0.519639388	0.739	0.193	4.37E-217
Rheb	2.40E-171	0.517722887	0.555	0.119	3.21E-167
Mrpl54	1.76E-186	0.517701014	0.601	0.132	2.36E-182
Rpl3	3.84E-71	0.516990726	0.213	0.011	5.14E-67
H2afz	5.30E-62	0.515059442	0.996	0.725	7.09E-58
Rps13	3.56E-64	0.514581565	0.972	0.645	4.77E-60
Scand1	1.11E-171	0.513928572	0.893	0.364	1.49E-167
Ndufa8	6.57E-183	0.513345886	0.579	0.128	8.79E-179
Psma1	5.88E-193	0.512545627	0.597	0.124	7.88E-189
Nxt1	2.20E-97	0.511900032	0.289	0.027	2.95E-93
Rgcc	4.79E-128	0.509773453	0.421	0.076	6.41E-124
Ddit4	1.04E-64	0.509740234	0.253	0.04	1.39E-60
Timm17b	3.42E-92	0.508785415	0.273	0.022	4.58E-88
Cotl1	3.10E-158	0.508579321	0.891	0.371	4.15E-154
Cd8b1	5.00E-134	0.508310609	0.401	0.055	6.70E-130
Dirc2	4.47E-77	-0.505485875	0.221	0.124	5.98E-73
Rgl2	4.16E-55	-0.505691908	0.154	0.099	5.57E-51
Gimap3	3.06E-81	-0.506493039	0.247	0.146	4.09E-77
Dcaf12	3.65E-63	-0.506503885	0.174	0.112	4.88E-59
Efr3a	1.03E-58	-0.506892824	0.18	0.117	1.38E-54
Epc1	3.72E-85	-0.507057774	0.235	0.136	4.98E-81
Acap2	1.23E-45	-0.507112893	0.14	0.104	1.64E-41
Rnf44	2.89E-50	-0.507286389	0.162	0.119	3.87E-46
Smox	3.18E-107	-0.507927156	0.291	0.155	4.26E-103
Otulin	1.78E-76	-0.508167933	0.241	0.157	2.39E-72
Pyhin1	7.10E-144	-0.50822607	0.457	0.241	9.51E-140
Rbm47	2.15E-64	-0.508853313	0.178	0.108	2.88E-60
Nop56	3.96E-69	-0.509023611	0.194	0.121	5.31E-65
Eif3j1	1.24E-82	-0.509919299	0.241	0.142	1.66E-78
Trim12c	8.86E-53	-0.510066588	0.172	0.119	1.19E-48
Csf2ra	4.07E-194	-0.510416734	0.672	0.357	5.45E-190

Sltm	8.29E-74	-0.512040345	0.208	0.124	1.11E-69
Pak2	6.72E-135	-0.512140977	0.389	0.214	8.99E-131
Scaf11	8.06E-73	-0.512353018	0.204	0.124	1.08E-68
Usp8	2.11E-46	-0.514001773	0.142	0.105	2.82E-42
Arhgef2	2.03E-65	-0.514224779	0.192	0.123	2.72E-61
Magt1	3.35E-109	-0.51469503	0.32	0.176	4.49E-105
Cmtm6	4.30E-84	-0.515240345	0.255	0.152	5.76E-80
Arhgef3	1.86E-54	-0.515588889	0.16	0.112	2.49E-50
Parp8	1.30E-66	-0.516519591	0.19	0.123	1.74E-62
Wdr43	2.01E-38	-0.516662732	0.113	0.1	2.69E-34
Lims1	3.01E-128	-0.517620244	0.391	0.211	4.03E-124
Cdk11b	4.44E-50	-0.518124185	0.144	0.101	5.95E-46
Sgk3	6.17E-101	-0.518538401	0.267	0.152	8.26E-97
Lcor	1.15E-36	-0.518769084	0.113	0.097	1.54E-32
Luc7l3	3.08E-62	-0.520402537	0.204	0.137	4.13E-58
Csnk1g3	2.79E-39	-0.52044238	0.119	0.098	3.73E-35
Top2b	1.06E-81	-0.520733157	0.227	0.137	1.42E-77
Etv6	8.58E-57	-0.521784254	0.166	0.11	1.15E-52
Rtn4	2.65E-184	-0.522013022	0.547	0.297	3.54E-180
Phf3	3.52E-58	-0.522171453	0.172	0.122	4.71E-54
Atp6v1b2	9.80E-164	-0.522185083	0.47	0.238	1.31E-159
Hipk2	1.28E-49	-0.522310148	0.166	0.119	1.71E-45
Kat2b	1.75E-48	-0.522678271	0.15	0.114	2.34E-44
Shoc2	3.11E-35	-0.522957174	0.107	0.093	4.17E-31
Pan3	4.06E-56	-0.523061389	0.166	0.116	5.43E-52
Irf1	1.27E-133	-0.523905362	0.455	0.235	1.71E-129
Arhgef10l	1.03E-39	-0.524037146	0.115	0.087	1.38E-35
Ctnnb1	2.17E-92	-0.524087145	0.283	0.177	2.91E-88
Tgfbr2	7.23E-55	-0.524326803	0.162	0.111	9.67E-51
Eps15	7.90E-66	-0.525516892	0.18	0.118	1.06E-61
Zc3h11a	3.47E-74	-0.526209821	0.213	0.133	4.64E-70
Safb	2.69E-84	-0.526713445	0.245	0.144	3.60E-80
Tardbp	1.27E-87	-0.527718189	0.265	0.169	1.70E-83
Msl2	2.24E-77	-0.528739716	0.223	0.141	3.00E-73
Rps27a	6.15E-47	-0.529082169	1	0.978	8.23E-43
Pias1	5.37E-48	-0.52916126	0.148	0.113	7.18E-44
Gnai3	3.03E-96	-0.529211472	0.265	0.156	4.06E-92
Cnot2	2.47E-36	-0.529347148	0.107	0.092	3.31E-32
Limd1	2.34E-88	-0.531632562	0.231	0.14	3.13E-84
Fam102b	4.59E-46	-0.531680961	0.13	0.096	6.14E-42
Icam1	2.86E-102	-0.531805607	0.354	0.199	3.83E-98
Ino80d	1.68E-44	-0.533056451	0.13	0.103	2.25E-40
Fosb	4.11E-129	-0.533537487	0.498	0.305	5.50E-125
Dhx9	4.13E-51	-0.534748843	0.16	0.114	5.53E-47
Rpl22	1.90E-113	-0.535013873	0.98	0.804	2.55E-109
Pcna	6.97E-125	-0.535275494	0.354	0.195	9.33E-121
Gramd3	2.37E-83	-0.53614915	0.245	0.148	3.17E-79
Tnrc6c	3.68E-48	-0.536482902	0.142	0.109	4.92E-44
Cd2ap	1.18E-65	-0.537237627	0.204	0.13	1.58E-61
Ppp1r15b	1.12E-82	-0.538904831	0.229	0.142	1.50E-78
Usp19	1.98E-57	-0.539002683	0.17	0.126	2.65E-53
Zfp703	5.11E-62	-0.540776416	0.176	0.11	6.84E-58
Kctd12	2.07E-163	-0.540871787	0.549	0.315	2.77E-159
Fryl	3.73E-44	-0.541070791	0.134	0.11	4.99E-40
Gpatch8	1.39E-38	-0.54116107	0.109	0.092	1.86E-34
Ddx18	3.39E-41	-0.541261041	0.134	0.11	4.54E-37
Gpr141	4.42E-104	-0.542615733	0.316	0.19	5.91E-100
Ptpn12	2.50E-83	-0.542999791	0.247	0.157	3.34E-79

Rps25	2.40E-104	-0.543034291	0.978	0.814	3.22E-100
Adrbk1	8.21E-180	-0.54313304	0.579	0.324	1.10E-175
Sp1	2.16E-44	-0.545192038	0.126	0.101	2.89E-40
Hnrnpa3	1.24E-194	-0.545625683	0.763	0.464	1.65E-190
Ctnna1	6.42E-62	-0.545632447	0.178	0.122	8.59E-58
Eef2	2.57E-117	-0.547500121	0.97	0.778	3.44E-113
Med13	4.41E-41	-0.547855199	0.117	0.096	5.91E-37
Pid1	3.33E-102	-0.548112435	0.291	0.168	4.45E-98
Prpf18	1.31E-37	-0.548509496	0.117	0.098	1.75E-33
Arl5b	3.27E-36	-0.548899891	0.105	0.093	4.38E-32
Csgalnact2	6.98E-71	-0.54926541	0.198	0.124	9.34E-67
Atp6v1a	2.37E-106	-0.549691675	0.285	0.162	3.17E-102
Zfp869	2.41E-33	-0.550542612	0.105	0.098	3.23E-29
Pou2f2	1.31E-41	-0.550674467	0.136	0.102	1.75E-37
Aff4	3.10E-58	-0.550997457	0.17	0.119	4.15E-54
Il2rb	9.57E-99	-0.551146557	0.306	0.165	1.28E-94
Prkx	1.42E-81	-0.551186228	0.247	0.152	1.90E-77
Zcchc7	1.13E-41	-0.551428746	0.128	0.11	1.51E-37
Srsf11	1.89E-84	-0.552557045	0.251	0.163	2.53E-80
Irak2	1.59E-75	-0.552651271	0.219	0.146	2.12E-71
Selt	1.10E-155	-0.552672018	0.47	0.269	1.47E-151
Cblb	2.03E-33	-0.553184629	0.103	0.091	2.71E-29
Zdhhc18	1.63E-50	-0.553223189	0.134	0.1	2.18E-46
Cdk17	2.54E-41	-0.553761521	0.121	0.102	3.39E-37
Ifi204	2.06E-137	-0.555195242	0.433	0.234	2.75E-133
Wac	6.52E-83	-0.556180742	0.249	0.152	8.73E-79
Asxl2	2.87E-49	-0.556620806	0.144	0.107	3.85E-45
Rplp1	5.43E-42	-0.556651459	1	0.984	7.27E-38
Qk	3.29E-122	-0.556935892	0.348	0.204	4.40E-118
Irf2	4.03E-98	-0.557451837	0.289	0.179	5.39E-94
Serpinb9	9.73E-56	-0.557600934	0.172	0.114	1.30E-51
Laptm5	2.69E-153	-0.557847838	0.966	0.715	3.60E-149
Pdcd6ip	3.07E-126	-0.558393895	0.346	0.204	4.11E-122
Grina	1.18E-181	-0.558581647	0.583	0.322	1.57E-177
Ankrd44	2.94E-76	-0.559767762	0.223	0.144	3.94E-72
Nono	1.08E-139	-0.560200981	0.407	0.228	1.44E-135
Top1	2.08E-126	-0.560259957	0.372	0.215	2.78E-122
Fbxl3	1.34E-57	-0.560827237	0.164	0.121	1.79E-53
Nfkbid	7.91E-113	-0.561172376	0.332	0.195	1.06E-108
Mafk	7.11E-65	-0.563985379	0.176	0.123	9.52E-61
Mapk1ip1l	3.03E-54	-0.564329113	0.154	0.116	4.06E-50
Phf20l1	1.86E-71	-0.564548466	0.198	0.132	2.49E-67
Rgs2	7.38E-135	-0.56463934	0.804	0.547	9.87E-131
Alyref	1.70E-82	-0.567874706	0.235	0.154	2.27E-78
Shfm1	4.39E-66	-0.567922775	0.984	0.888	5.88E-62
Prdm1	2.66E-37	-0.569416016	0.109	0.087	3.56E-33
Ankrd12	4.70E-42	-0.569784425	0.128	0.105	6.29E-38
Ehbp1l1	5.40E-100	-0.569784684	0.292	0.175	7.23E-96
Vcpip1	3.30E-43	-0.569787107	0.126	0.104	4.42E-39
Matr3	8.10E-85	-0.569871226	0.271	0.174	1.08E-80
Tnrc18	1.74E-51	-0.569920536	0.138	0.106	2.33E-47
Dgat1	7.77E-135	-0.570530475	0.375	0.222	1.04E-130
Fbrsl1	3.29E-37	-0.570798751	0.101	0.093	4.41E-33
Cybb	1.66E-137	-0.570895003	0.927	0.682	2.22E-133
Rassf2	4.82E-65	-0.571528077	0.186	0.129	6.45E-61
Prcp	5.07E-150	-0.571540139	0.458	0.258	6.78E-146
Mfsd14b	1.64E-72	-0.571581334	0.221	0.147	2.20E-68
Mbd2	1.65E-78	-0.571647946	0.227	0.153	2.21E-74

Hsp90ab1	6.59E-130	-0.572167025	0.945	0.72	8.82E-126
Noc2l	4.45E-50	-0.57332496	0.136	0.105	5.95E-46
Ncf1	1.89E-176	-0.573380936	0.538	0.294	2.53E-172
Zbtb7a	8.11E-83	-0.573523443	0.249	0.162	1.09E-78
Tet2	1.65E-39	-0.573841626	0.123	0.102	2.21E-35
Smad4	2.98E-71	-0.574127037	0.206	0.145	3.99E-67
Myo9b	2.52E-102	-0.574217069	0.281	0.175	3.37E-98
Aff1	1.32E-37	-0.57433974	0.117	0.109	1.77E-33
Pik3cd	9.54E-118	-0.574348584	0.35	0.215	1.28E-113
Adgre5	1.03E-177	-0.575044857	0.702	0.427	1.38E-173
Ezr	1.08E-146	-0.575223229	0.447	0.248	1.45E-142
Elf1	8.05E-109	-0.57576905	0.322	0.192	1.08E-104
Pik3r1	1.75E-72	-0.575832694	0.213	0.137	2.34E-68
Klhl9	1.69E-79	-0.575883066	0.208	0.138	2.26E-75
G3bp2	7.48E-103	-0.576046546	0.291	0.173	1.00E-98
Sorl1	2.13E-136	-0.576106148	0.435	0.257	2.85E-132
Dhx40	2.40E-65	-0.576924632	0.202	0.134	3.21E-61
Setd2	2.90E-29	-0.577035577	0.095	0.105	3.88E-25
Fndc3b	9.16E-48	-0.577169727	0.13	0.103	1.23E-43
Sfpq	1.84E-143	-0.577710646	0.441	0.26	2.46E-139
Akap13	1.61E-168	-0.578388574	0.506	0.275	2.16E-164
Mapkapk2	1.22E-154	-0.578865111	0.482	0.272	1.64E-150
Mat2a	8.00E-123	-0.57969793	0.356	0.208	1.07E-118
Fbxl5	3.43E-118	-0.580121374	0.336	0.191	4.59E-114
Tgm2	7.64E-81	-0.580281518	0.235	0.136	1.02E-76
Myeov2	1.01E-194	-0.581257861	0.862	0.576	1.35E-190
Ptk2b	1.17E-136	-0.582427945	0.36	0.199	1.56E-132
Ube2h	2.42E-74	-0.582558873	0.211	0.137	3.24E-70
Eif2s3x	2.58E-62	-0.582831943	0.19	0.141	3.45E-58
Flcn	1.85E-47	-0.584112057	0.134	0.108	2.47E-43
Atf7ip	5.21E-95	-0.584227702	0.243	0.137	6.98E-91
Zcchc11	6.30E-53	-0.58432906	0.162	0.126	8.43E-49
Nktr	6.92E-56	-0.5845454	0.168	0.124	9.26E-52
Zmiz1	1.47E-97	-0.585021985	0.285	0.187	1.96E-93
H2-K1	2.35E-99	-0.585204696	0.992	0.836	3.15E-95
Hivep2	6.01E-52	-0.586226056	0.158	0.12	8.05E-48
Man2a1	2.38E-75	-0.586675017	0.204	0.138	3.19E-71
Kpna4	9.35E-107	-0.587051815	0.296	0.175	1.25E-102
Rnd3	1.69E-45	-0.588112845	0.16	0.108	2.26E-41
Cers6	1.26E-141	-0.588515677	0.401	0.222	1.69E-137
Wdr89	2.39E-67	-0.588533152	1	0.991	3.20E-63
Sf3b1	3.80E-183	-0.589412001	0.619	0.375	5.09E-179
Pura	3.94E-52	-0.589534238	0.146	0.116	5.28E-48
Hsd17b11	7.74E-35	-0.589607827	0.105	0.1	1.04E-30
Usp25	4.45E-100	-0.590553287	0.281	0.178	5.96E-96
Olfr1033	2.57E-42	-0.590887529	0.219	0.163	3.44E-38
Adnp	7.71E-52	-0.593457152	0.148	0.121	1.03E-47
Ppp2r5c	1.46E-117	-0.593932816	0.34	0.204	1.95E-113
Gnb1	1.29E-182	-0.595101414	0.589	0.341	1.73E-178
Abcg1	3.79E-56	-0.595717125	0.174	0.124	5.08E-52
Etnk1	1.98E-71	-0.59602042	0.194	0.134	2.65E-67
Foxn3	3.05E-124	-0.59674922	0.372	0.232	4.09E-120
Cnbd2	1.33E-79	-0.59786036	0.211	0.143	1.78E-75
Runx2	1.85E-61	-0.597968039	0.192	0.127	2.47E-57
Pbrm1	1.20E-83	-0.599722265	0.261	0.175	1.61E-79
Wnk1	1.99E-107	-0.599790129	0.328	0.204	2.66E-103
Eif3c	2.21E-89	-0.602098752	0.255	0.157	2.96E-85
Ugcg	2.44E-69	-0.603332391	0.206	0.141	3.27E-65

Actr2	6.01E-199	-0.60360842	0.65	0.39	8.04E-195
Malt1	4.85E-71	-0.60433914	0.249	0.159	6.49E-67
Mbtd1	2.24E-61	-0.604425154	0.182	0.136	3.00E-57
Trip12	5.92E-105	-0.605019665	0.296	0.19	7.92E-101
Adam17	4.79E-67	-0.60554703	0.198	0.138	6.41E-63
Ppp1r12a	1.27E-81	-0.606985447	0.245	0.165	1.70E-77
Hes1	8.16E-66	-0.607277072	0.291	0.174	1.09E-61
Arid1a	1.91E-115	-0.607290622	0.306	0.182	2.56E-111
Sf1	2.97E-151	-0.607485331	0.443	0.263	3.98E-147
Rad21	5.31E-91	-0.608068974	0.255	0.152	7.11E-87
Prkcb	4.55E-121	-0.609388095	0.338	0.188	6.08E-117
Sdcbp	1.75E-182	-0.61088144	0.83	0.553	2.35E-178
Atrx	1.47E-96	-0.611684621	0.263	0.166	1.97E-92
Ccng2	2.52E-66	-0.612579225	0.19	0.136	3.37E-62
Ppp4r2	1.47E-93	-0.612745061	0.259	0.172	1.97E-89
Cggbp1	8.77E-106	-0.612904971	0.306	0.202	1.17E-101
Klhl2	5.94E-50	-0.614185635	0.132	0.11	7.95E-46
D16Ertd472e	8.37E-89	-0.614230742	0.259	0.17	1.12E-84
N4bp1	4.76E-77	-0.616590713	0.2	0.134	6.37E-73
Fbxo33	3.63E-49	-0.61688203	0.142	0.112	4.86E-45
Rsad2	5.75E-23	-0.617062416	0.113	0.087	7.70E-19
Tpd52	1.73E-177	-0.617606167	0.84	0.566	2.31E-173
Arhgap30	3.14E-146	-0.619003577	0.451	0.269	4.21E-142
Phip	3.13E-38	-0.619522973	0.105	0.099	4.19E-34
Nr3c1	1.30E-90	-0.619547345	0.253	0.165	1.74E-86
Syk	2.34E-155	-0.621395072	0.474	0.282	3.14E-151
Far1	8.44E-92	-0.621714676	0.247	0.163	1.13E-87
Gatad2b	5.06E-68	-0.624040728	0.168	0.13	6.78E-64
Osm	1.96E-130	-0.624485621	0.54	0.317	2.63E-126
Lamp2	4.87E-196	-0.625932314	0.619	0.359	6.52E-192
Ppp6r1	4.16E-66	-0.626058918	0.186	0.137	5.56E-62
Tab2	5.22E-99	-0.626060897	0.3	0.203	6.99E-95
Cyld	5.95E-54	-0.627249609	0.146	0.123	7.96E-50
Cyth1	4.03E-90	-0.63041599	0.263	0.173	5.40E-86
Tnrc6b	2.01E-47	-0.631381576	0.138	0.119	2.69E-43
Gpbp1l1	2.32E-37	-0.632427881	0.109	0.107	3.10E-33
Slc44a2	5.99E-107	-0.634744227	0.312	0.196	8.02E-103
Kdm5a	9.62E-50	-0.635294452	0.152	0.129	1.29E-45
B4galt5	1.49E-78	-0.635728764	0.213	0.149	2.00E-74
Elovl5	5.99E-95	-0.636018722	0.271	0.183	8.02E-91
Fam46a	3.03E-64	-0.636127662	0.198	0.143	4.06E-60
Cab39	9.85E-88	-0.636152566	0.253	0.175	1.32E-83
Xiap	2.92E-79	-0.636873701	0.219	0.161	3.90E-75
G3bp1	1.32E-135	-0.636922791	0.403	0.247	1.77E-131
Rdm1	1.31E-126	-0.637336626	0.387	0.221	1.75E-122
Cpeb2	2.94E-46	-0.637606607	0.125	0.11	3.93E-42
Sppl2a	2.34E-95	-0.637838028	0.253	0.165	3.14E-91
Add3	3.83E-100	-0.638238887	0.267	0.165	5.12E-96
Suco	3.88E-41	-0.638464922	0.115	0.114	5.19E-37
Nab1	5.82E-87	-0.638812312	0.253	0.176	7.79E-83
Ip6k1	2.05E-50	-0.638828964	0.152	0.136	2.74E-46
Pabpc4	2.40E-34	-0.639707452	0.111	0.111	3.21E-30
Cbfa2t3	1.69E-77	-0.640414414	0.269	0.186	2.26E-73
Dusp3	4.90E-116	-0.640441286	0.35	0.197	6.56E-112
Birc2	1.49E-50	-0.641559962	0.142	0.12	2.00E-46
Elf2	7.07E-55	-0.642694756	0.146	0.127	9.47E-51
Dync1h1	7.33E-76	-0.643240224	0.213	0.144	9.82E-72
Rpl41	1.06E-127	-0.643398404	1	1	1.42E-123

Kat6a	1.77E-61	-0.64364494	0.166	0.134	2.37E-57
4932438A13Rik	2.01E-34	-0.643849787	0.101	0.108	2.69E-30
Baz1a	4.41E-107	-0.643917508	0.306	0.202	5.90E-103
Ddhd1	6.79E-46	-0.644347849	0.13	0.112	9.09E-42
St8sia4	7.39E-130	-0.644604178	0.366	0.225	9.90E-126
Gpbp1	6.75E-127	-0.644731312	0.334	0.21	9.03E-123
Zcchc6	1.91E-78	-0.645141865	0.219	0.155	2.55E-74
Pip4k2a	1.55E-120	-0.645378809	0.34	0.215	2.07E-116
Slfn4	1.55E-44	-0.645957062	0.126	0.114	2.07E-40
Ptprj	7.61E-104	-0.646288228	0.306	0.199	1.02E-99
Zc3h13	1.48E-42	-0.646801202	0.136	0.125	1.98E-38
Ogt	7.72E-58	-0.647333378	0.168	0.136	1.03E-53
Sdc3	3.26E-52	-0.647416845	0.164	0.132	4.36E-48
Smek1	3.83E-47	-0.649498888	0.134	0.122	5.13E-43
Mgea5	4.91E-75	-0.649901523	0.223	0.166	6.57E-71
Csf2rb	7.94E-135	-0.650137887	0.425	0.257	1.06E-130
Prex1	1.07E-88	-0.650140345	0.243	0.173	1.43E-84
Arhgap17	4.03E-83	-0.650917834	0.211	0.148	5.40E-79
Alcam	2.77E-60	-0.65106942	0.16	0.12	3.71E-56
Vps4b	1.82E-89	-0.651115413	0.265	0.182	2.44E-85
Ppig	8.02E-114	-0.652125517	0.34	0.218	1.07E-109
Fam49a	4.16E-102	-0.652701782	0.298	0.199	5.56E-98
Nfil3	9.35E-177	-0.652813412	0.634	0.386	1.25E-172
Ccdc88a	1.03E-77	-0.653863882	0.239	0.166	1.38E-73
Odc1	2.02E-107	-0.654093843	0.33	0.198	2.70E-103
Cd300lf	4.07E-105	-0.654988694	0.306	0.205	5.45E-101
Neurl3	5.32E-96	-0.65581403	0.302	0.206	7.12E-92
Tgif1	3.60E-156	-0.657765598	0.514	0.306	4.81E-152
Spty2d1	2.41E-68	-0.658317904	0.18	0.14	3.22E-64
Klhl24	1.07E-83	-0.658837513	0.253	0.177	1.44E-79
Gsr	3.24E-163	-0.658881672	0.868	0.613	4.34E-159
Srsf6	8.21E-177	-0.659654997	0.502	0.298	1.10E-172
Bcl11a	8.38E-36	-0.661188095	0.121	0.108	1.12E-31
Pcbp1	9.37E-217	-0.662644844	0.779	0.483	1.25E-212
Pafah1b1	4.12E-141	-0.665241815	0.389	0.244	5.51E-137
Birc3	1.92E-123	-0.666305599	0.372	0.238	2.57E-119
Serinc1	2.20E-116	-0.666493773	0.316	0.197	2.94E-112
Mef2a	1.18E-113	-0.667364403	0.32	0.206	1.58E-109
Tgfbi	2.45E-144	-0.667367108	0.798	0.575	3.28E-140
Tbl1xr1	3.01E-86	-0.668044265	0.245	0.173	4.03E-82
Ssh2	1.88E-167	-0.668939789	0.5	0.309	2.52E-163
Ptger4	1.18E-51	-0.669304003	0.148	0.122	1.58E-47
Heca	3.92E-58	-0.670341775	0.166	0.132	5.24E-54
Tnfrsf1b	7.43E-145	-0.671415518	0.516	0.331	9.95E-141
Efhd2	2.62E-173	-0.672060321	0.648	0.418	3.51E-169
Mepce	8.15E-50	-0.673694179	0.13	0.122	1.09E-45
Ibtk	1.33E-59	-0.673918709	0.172	0.139	1.78E-55
Sh3bgrl	7.08E-180	-0.674168384	0.498	0.288	9.47E-176
Rnf19b	4.44E-78	-0.674973635	0.223	0.16	5.95E-74
Nlrp3	4.25E-108	-0.675419347	0.377	0.235	5.69E-104
Bcl6	4.82E-61	-0.676024873	0.164	0.136	6.45E-57
Rbm39	2.07E-170	-0.677754803	0.939	0.711	2.77E-166
Larp4b	1.45E-136	-0.677852279	0.391	0.248	1.94E-132
Celf1	4.36E-85	-0.678434322	0.255	0.188	5.84E-81
Sp110	1.27E-126	-0.678928317	0.364	0.226	1.70E-122
Ppp1r15a	1.96E-171	-0.679293559	0.751	0.473	2.63E-167
Mapk1	1.18E-127	-0.681600869	0.37	0.244	1.58E-123
Smchd1	5.20E-62	-0.681633038	0.192	0.157	6.96E-58

Zc3hav1	1.40E-144	-0.681681937	0.383	0.237	1.87E-140
Cdkn1b	3.17E-68	-0.682029638	0.208	0.156	4.25E-64
Ikzf1	5.58E-155	-0.682239171	0.439	0.267	7.47E-151
Uqcrh	1.56E-113	-0.683005703	0.964	0.834	2.09E-109
Arid5a	1.05E-118	-0.683314853	0.379	0.256	1.40E-114
Ubl3	2.91E-187	-0.683433393	0.563	0.346	3.89E-183
Tle3	2.84E-115	-0.683680457	0.35	0.234	3.80E-111
Eif2s2	3.16E-201	-0.683982566	0.706	0.453	4.23E-197
Gnaq	2.15E-38	-0.685045306	0.107	0.112	2.88E-34
Clint1	1.40E-109	-0.686208041	0.312	0.208	1.88E-105
Nup98	2.88E-57	-0.687977434	0.156	0.131	3.86E-53
Zfand5	2.08E-166	-0.68823863	0.706	0.458	2.79E-162
Itgam	5.44E-155	-0.688930828	0.468	0.291	7.28E-151
Tgoln1	2.97E-132	-0.688984171	0.389	0.254	3.97E-128
Ddx3x	2.77E-160	-0.691393612	0.502	0.315	3.71E-156
Ccdc88c	1.72E-77	-0.69200381	0.229	0.163	2.31E-73
Tob1	1.98E-70	-0.69251421	0.2	0.152	2.65E-66
Myh9	2.07E-124	-0.69300564	0.37	0.239	2.78E-120
Hsp90aa1	1.49E-143	-0.693649537	0.84	0.559	1.99E-139
Mxd1	7.14E-172	-0.695119772	0.589	0.362	9.56E-168
Pdpk1	2.54E-43	-0.695208602	0.117	0.118	3.40E-39
Fnip1	3.07E-59	-0.695323819	0.152	0.127	4.11E-55
Il4ra	2.85E-134	-0.69558699	0.411	0.266	3.82E-130
Mt-mt-Rnr2	1.69E-90	-0.698007103	0.978	0.831	2.26E-86
Lbr	5.22E-143	-0.698487422	0.445	0.29	6.98E-139
Rsrc2	1.59E-139	-0.699856786	0.413	0.275	2.12E-135
Slc20a1	2.01E-43	-0.699880359	0.119	0.118	2.69E-39
Ptafr	3.82E-124	-0.701534158	0.455	0.308	5.11E-120
Tkt	8.89E-184	-0.702375856	0.654	0.428	1.19E-179
Esyt2	4.89E-53	-0.702861129	0.142	0.133	6.54E-49
Lrrfip1	2.72E-168	-0.706634765	0.476	0.298	3.65E-164
Psap	1.09E-53	-0.706679202	0.996	0.98	1.46E-49
Arid4a	3.13E-58	-0.707462604	0.174	0.149	4.19E-54
Kdm6b	7.05E-144	-0.707548088	0.506	0.317	9.44E-140
Cebpz	5.59E-101	-0.708400391	0.294	0.197	7.49E-97
Mef2c	1.17E-89	-0.70940649	0.283	0.182	1.56E-85
Snrnp70	1.20E-159	-0.709605307	0.455	0.285	1.60E-155
Stk38	7.97E-98	-0.710754458	0.281	0.207	1.07E-93
Hsph1	7.26E-132	-0.710853321	0.401	0.24	9.72E-128
Chd7	6.57E-89	-0.712229312	0.251	0.175	8.79E-85
Rpl37a	2.38E-197	-0.714719777	0.808	0.565	3.19E-193
Eif5b	6.73E-144	-0.714907061	0.429	0.276	9.01E-140
Sp3	5.24E-68	-0.715558732	0.176	0.143	7.02E-64
Impact	1.81E-58	-0.716549068	0.16	0.139	2.42E-54
Il1rn	1.51E-80	-0.716749226	0.36	0.199	2.02E-76
Pum1	2.76E-60	-0.717812912	0.174	0.151	3.70E-56
Elavl1	6.90E-124	-0.718346039	0.354	0.244	9.23E-120
Rassf5	6.76E-120	-0.722513772	0.31	0.216	9.05E-116
Mapk6	7.38E-88	-0.723352251	0.223	0.158	9.88E-84
Csf3r	6.11E-151	-0.727183988	0.472	0.302	8.18E-147
Tle4	1.30E-63	-0.728922298	0.166	0.144	1.74E-59
Nabp1	4.21E-106	-0.729704337	0.287	0.206	5.63E-102
Sgms1	1.49E-73	-0.733217267	0.2	0.155	1.99E-69
Wipf1	8.04E-134	-0.733368864	0.383	0.257	1.08E-129
Taok1	7.20E-66	-0.73465988	0.178	0.152	9.63E-62
Per1	6.60E-114	-0.734954623	0.322	0.225	8.84E-110
Sod1	4.67E-100	-0.73878524	0.285	0.211	6.25E-96
Ywhaz	1.55E-213	-0.739296375	0.745	0.499	2.07E-209

Abcf1	2.96E-70	-0.73959314	0.2	0.161	3.96E-66
Dennd5a	2.66E-98	-0.739836132	0.251	0.19	3.56E-94
Polr2a	3.60E-128	-0.741180887	0.336	0.218	4.83E-124
Plekho2	8.01E-133	-0.741990694	0.387	0.256	1.07E-128
Stat3	1.33E-172	-0.743651265	0.514	0.317	1.78E-168
Pnisr	4.45E-96	-0.743732606	0.283	0.208	5.95E-92
Dck	6.87E-104	-0.745158317	0.273	0.197	9.19E-100
Cytip	8.66E-161	-0.747307368	0.875	0.669	1.16E-156
Pim1	3.11E-165	-0.749272909	0.571	0.372	4.17E-161
Sgk1	7.65E-107	-0.749476261	0.33	0.206	1.02E-102
Lmnb1	3.06E-161	-0.752289578	0.49	0.315	4.10E-157
Aplp2	1.56E-119	-0.752467368	0.35	0.259	2.09E-115
Ddit3	1.97E-164	-0.752601766	0.496	0.313	2.63E-160
Cnot6l	3.49E-89	-0.753729964	0.235	0.172	4.67E-85
Cd274	2.80E-38	-0.754612995	0.109	0.109	3.75E-34
Hnrnpul2	5.31E-120	-0.75506273	0.32	0.224	7.10E-116
Rnf213	9.09E-103	-0.755246882	0.3	0.208	1.22E-98
Morc3	2.32E-51	-0.757388834	0.13	0.128	3.11E-47
Il17ra	2.94E-142	-0.759383872	0.49	0.315	3.94E-138
Tnf	6.56E-45	-0.763142641	0.215	0.111	8.78E-41
Skil	1.97E-104	-0.763239896	0.34	0.242	2.64E-100
Map4k4	1.42E-86	-0.763552304	0.263	0.192	1.90E-82
Il1b	1.93E-91	-0.764314593	0.986	0.803	2.59E-87
Dazap2	9.36E-216	-0.7659228	0.783	0.514	1.25E-211
Picalm	1.08E-208	-0.765987282	0.664	0.423	1.45E-204
Ewsr1	7.03E-163	-0.766482859	0.457	0.308	9.41E-159
Jdp2	3.05E-39	-0.770289265	0.095	0.113	4.08E-35
Eif4g2	1.22E-226	-0.770892692	0.725	0.468	1.63E-222
Ythdf3	1.09E-99	-0.771861066	0.269	0.193	1.46E-95
Ier5	7.69E-182	-0.773210405	0.854	0.621	1.03E-177
Cflar	1.32E-102	-0.773419533	0.302	0.202	1.77E-98
Vcan	1.10E-111	-0.773535457	0.358	0.235	1.47E-107
Hnrnpdl	4.39E-170	-0.775329226	0.5	0.322	5.87E-166
Synj1	8.52E-91	-0.776187438	0.237	0.177	1.14E-86
Nampt	5.01E-104	-0.777278531	0.306	0.208	6.71E-100
Arl8a	8.10E-94	-0.778541965	0.243	0.191	1.08E-89
Camk2d	3.20E-86	-0.778972649	0.223	0.171	4.28E-82
Csnk1a1	5.70E-173	-0.780480381	0.532	0.368	7.63E-169
Ddx3y	4.69E-116	-0.78161447	0.336	0.233	6.28E-112
Tnrc6a	9.17E-48	-0.782667046	0.128	0.139	1.23E-43
Dhx15	6.22E-87	-0.784068693	0.243	0.2	8.32E-83
St3gal4	2.06E-138	-0.784533199	0.597	0.374	2.76E-134
Hdc	4.29E-169	-0.786538582	0.488	0.292	5.74E-165
Lpl	9.11E-24	-0.7888592	0.123	0.072	1.22E-19
Tnks2	4.28E-75	-0.7891246	0.194	0.183	5.74E-71
Samhd1	1.33E-178	-0.791797961	0.652	0.447	1.77E-174
Nipbl	4.35E-106	-0.791880112	0.273	0.208	5.83E-102
Rpl28	1.54E-56	-0.79364485	0.136	0.142	2.05E-52
Abi1	4.26E-159	-0.793714473	0.435	0.292	5.70E-155
Arih1	2.76E-96	-0.796219659	0.281	0.215	3.69E-92
Wdr26	1.47E-146	-0.79647534	0.407	0.28	1.97E-142
Myadm	1.27E-140	-0.798107329	0.385	0.257	1.70E-136
Nufip2	1.43E-98	-0.798378176	0.251	0.192	1.91E-94
Nfkb1	1.07E-138	-0.798681689	0.409	0.269	1.43E-134
Iqgap1	1.35E-186	-0.799388013	0.621	0.431	1.81E-182
Tor1aip1	1.25E-131	-0.800772107	0.383	0.269	1.68E-127
Foxp1	1.36E-151	-0.801016816	0.466	0.309	1.83E-147
Cox17	1.48E-171	-0.801765413	0.739	0.544	1.97E-167

Hipk1	1.69E-117	-0.802849639	0.314	0.229	2.26E-113
Macf1	1.58E-108	-0.803048999	0.285	0.211	2.12E-104
P2ry10	2.59E-97	-0.803535618	0.265	0.206	3.47E-93
Ncl	1.57E-168	-0.804943342	0.538	0.362	2.11E-164
App	2.67E-153	-0.805607723	0.455	0.309	3.57E-149
Rassf3	2.60E-99	-0.806340698	0.255	0.192	3.49E-95
Prkcd	4.14E-170	-0.807467895	0.538	0.342	5.54E-166
Gm1966	7.00E-94	-0.811208045	0.245	0.188	9.37E-90
Aebp2	7.40E-50	-0.811259744	0.126	0.141	9.91E-46
Clec2d	1.89E-73	-0.811337632	0.204	0.17	2.53E-69
Zswim6	4.99E-52	-0.811408814	0.136	0.145	6.67E-48
Lyn	4.20E-192	-0.812676429	0.636	0.428	5.62E-188
Jak1	5.60E-174	-0.814882718	0.5	0.338	7.50E-170
Clic4	4.28E-89	-0.817186253	0.285	0.197	5.74E-85
Lmo4	9.93E-123	-0.818218887	0.377	0.261	1.33E-118
Crebrf	3.79E-53	-0.820407713	0.13	0.146	5.07E-49
Azin1	2.67E-111	-0.829097225	0.298	0.222	3.57E-107
Samsn1	1.70E-165	-0.831002949	0.522	0.347	2.27E-161
Ptp4a1	1.70E-169	-0.832379441	0.611	0.434	2.27E-165
Gpcpd1	1.24E-141	-0.832874654	0.494	0.363	1.66E-137
Cd83	2.21E-127	-0.834297959	0.565	0.377	2.96E-123
Rtf1	1.67E-118	-0.835293973	0.291	0.219	2.24E-114
Nrip1	2.17E-52	-0.837326146	0.14	0.151	2.91E-48
Clk1	2.63E-188	-0.838989459	0.573	0.389	3.53E-184
Pum2	5.72E-136	-0.843604666	0.35	0.244	7.65E-132
Mycbp2	6.91E-118	-0.846423219	0.336	0.253	9.25E-114
Purb	3.02E-86	-0.847012864	0.231	0.201	4.05E-82
Dock10	5.90E-105	-0.849736476	0.306	0.244	7.90E-101
AI607873	5.14E-147	-0.850333899	0.466	0.315	6.88E-143
Trim25	1.35E-147	-0.854234903	0.439	0.32	1.80E-143
Ep300	5.98E-66	-0.854254972	0.162	0.167	8.00E-62
Ppp3ca	2.63E-141	-0.871984136	0.36	0.264	3.52E-137
Vegfa	3.65E-43	-0.87477295	0.13	0.123	4.88E-39
Pde4b	8.68E-156	-0.87632247	0.502	0.347	1.16E-151
Slk	7.55E-109	-0.877126604	0.281	0.233	1.01E-104
Thbd	1.47E-52	-0.883070378	0.172	0.149	1.97E-48
Amd1	1.37E-55	-0.8833271	0.138	0.15	1.83E-51
Tcf4	1.22E-90	-0.883679754	0.298	0.226	1.64E-86
Tcp11l2	8.67E-118	-0.884004732	0.328	0.26	1.16E-113
Slc6a6	2.18E-116	-0.887859584	0.326	0.268	2.91E-112
Eif3a	2.26E-171	-0.891571236	0.526	0.375	3.03E-167
Trps1	7.59E-107	-0.891999291	0.277	0.211	1.02E-102
Emilin2	1.02E-198	-0.892794168	0.698	0.474	1.37E-194
Srrm2	8.73E-180	-0.893111732	0.526	0.377	1.17E-175
Chka	4.92E-71	-0.893618448	0.239	0.191	6.59E-67
Hnrnph1	1.36E-167	-0.894188665	0.492	0.345	1.82E-163
Klf10	1.04E-117	-0.894593862	0.415	0.302	1.39E-113
Ap2a2	2.36E-92	-0.899120545	0.265	0.23	3.16E-88
Bhlhe40	9.91E-94	-0.901913271	0.35	0.265	1.33E-89
Rrbp1	2.84E-199	-0.902368686	0.676	0.468	3.80E-195
Spag9	8.57E-102	-0.906493675	0.287	0.248	1.15E-97
Son	2.39E-214	-0.909094205	0.638	0.442	3.20E-210
Strn3	5.20E-62	-0.913362132	0.142	0.167	6.96E-58
Ptbp3	3.96E-179	-0.913580309	0.567	0.414	5.30E-175
Hif1a	2.19E-143	-0.913601344	0.383	0.285	2.93E-139
Atf4	1.45E-188	-0.916603354	0.706	0.526	1.94E-184
Junb	2.65E-86	-0.918127645	0.996	0.952	3.55E-82
Prpf38b	6.02E-143	-0.918389713	0.415	0.306	8.05E-139

Rbms1	7.15E-175	-0.920558493	0.534	0.378	9.57E-171
Atp2b1	7.50E-186	-0.920617829	0.617	0.437	1.00E-181
Mbnl1	7.23E-223	-0.923191906	0.67	0.459	9.67E-219
Sowahc	6.63E-108	-0.92370334	0.292	0.221	8.88E-104
Hnrnpm	4.38E-207	-0.924428773	0.589	0.417	5.87E-203
Trib1	2.10E-147	-0.929461726	0.427	0.324	2.81E-143
Runx1	3.28E-120	-0.930938953	0.346	0.266	4.39E-116
Bach1	1.84E-125	-0.931001292	0.326	0.264	2.46E-121
Map3k1	1.15E-123	-0.936026627	0.32	0.255	1.53E-119
Satb1	6.64E-118	-0.937761908	0.354	0.262	8.89E-114
Tiparp	3.02E-120	-0.938912281	0.332	0.251	4.05E-116
Trim30a	1.37E-116	-0.942560183	0.415	0.341	1.84E-112
Nfe2l2	2.36E-170	-0.944330218	0.662	0.501	3.16E-166
Akna	2.33E-103	-0.947171763	0.263	0.233	3.12E-99
Tob2	1.82E-110	-0.950635948	0.279	0.227	2.44E-106
Luc7l2	6.42E-205	-0.951046035	0.577	0.407	8.59E-201
Runx3	9.27E-111	-0.95496876	0.318	0.266	1.24E-106
Ankrd11	1.07E-94	-0.955516374	0.259	0.236	1.43E-90
Ist1	6.49E-164	-0.955625816	0.458	0.338	8.68E-160
Nr4a3	1.50E-79	-0.960163364	0.253	0.228	2.01E-75
Susd6	3.75E-83	-0.961101975	0.2	0.201	5.02E-79
Rab8b	3.30E-165	-0.971838525	0.455	0.343	4.42E-161
Cmip	4.00E-107	-0.974197565	0.312	0.284	5.36E-103
Ythdc1	4.09E-125	-0.977367755	0.346	0.288	5.48E-121
Nmt1	2.29E-127	-0.983712116	0.316	0.273	3.06E-123
Vps37b	1.43E-202	-0.983853794	0.65	0.472	1.91E-198
Gcnt2	1.75E-164	-0.98427557	0.514	0.358	2.34E-160
Atp1b3	3.41E-226	-0.984674986	0.735	0.521	4.57E-222
Sp100	1.02E-169	-0.988577625	0.49	0.368	1.36E-165
Tpt1	8.62E-146	-0.991463929	0.996	0.978	1.15E-141
Jarid2	3.84E-107	-0.993272821	0.285	0.247	5.14E-103
Wsb1	1.14E-148	-0.994176527	0.429	0.33	1.53E-144
Fus	1.58E-140	-0.994461001	0.391	0.308	2.12E-136
Gpr132	2.82E-168	-1.000792745	0.514	0.389	3.77E-164
Tra2a	8.71E-113	-1.001489721	0.287	0.259	1.17E-108
Ddx21	6.98E-117	-1.008304945	0.312	0.282	9.34E-113
Slc15a3	9.10E-121	-1.012721929	0.377	0.302	1.22E-116
Csrnp1	2.82E-172	-1.018013382	0.565	0.397	3.77E-168
Rel	1.41E-61	-1.018876309	0.17	0.182	1.89E-57
Ifrd1	1.00E-140	-1.020251086	0.737	0.599	1.34E-136
Gls	3.93E-120	-1.024955918	0.291	0.262	5.26E-116
Ets1	1.14E-158	-1.02505549	0.441	0.342	1.53E-154
Prrc2c	2.95E-82	-1.026018231	0.217	0.235	3.95E-78
Nfkbiz	3.79E-149	-1.026070546	0.593	0.416	5.08E-145
Ddx17	3.11E-155	-1.02764045	0.405	0.323	4.17E-151
Jun	2.78E-146	-1.034347859	0.84	0.66	3.72E-142
Mt-mt-Rnr1	2.09E-68	-1.041377765	0.188	0.211	2.80E-64
Gda	3.66E-198	-1.048193171	0.713	0.537	4.90E-194
Ets2	2.88E-162	-1.061365776	0.431	0.34	3.85E-158
Hexim1	6.21E-117	-1.06286681	0.271	0.256	8.31E-113
Zfp36	5.33E-137	-1.064485844	0.927	0.801	7.14E-133
Snx18	2.47E-165	-1.071452142	0.453	0.369	3.30E-161
Zfp36l2	6.31E-198	-1.078979386	0.753	0.577	8.44E-194
Eif3j2	2.46E-93	-1.080122761	0.229	0.246	3.29E-89
Hnrnpa0	5.96E-155	-1.085412602	0.399	0.343	7.98E-151
Celf2	1.57E-160	-1.089129682	0.498	0.434	2.10E-156
Egr1	1.46E-49	-1.100324089	0.2	0.184	1.96E-45
Mcl1	1.03E-182	-1.101417319	0.899	0.774	1.38E-178

Prpf4b	1.55E-112	-1.110624832	0.294	0.29	2.08E-108
Ptprc	3.52E-194	-1.123394937	0.872	0.75	4.71E-190
Nr4a1	1.06E-164	-1.124753135	0.804	0.655	1.42E-160
Itgal	2.85E-190	-1.133759786	0.652	0.506	3.82E-186
Bcl2l11	1.98E-158	-1.13987597	0.553	0.458	2.65E-154
Fos	1.56E-110	-1.142944679	0.992	0.919	2.08E-106
Zeb2	2.62E-164	-1.151331278	0.48	0.401	3.50E-160
Pcbp2	1.14E-227	-1.152623901	0.779	0.636	1.52E-223
Dennd4a	3.80E-148	-1.168175973	0.441	0.389	5.08E-144
Stk17b	4.40E-205	-1.175191636	0.86	0.724	5.89E-201
Hk2	2.09E-81	-1.176530924	0.259	0.257	2.80E-77
Ddx5	2.47E-216	-1.195373335	0.976	0.893	3.31E-212
Peli1	2.54E-155	-1.199151325	0.383	0.345	3.40E-151
Klf4	3.53E-139	-1.205316951	0.48	0.41	4.73E-135
Rps28	5.35E-50	-1.210601267	0.073	0.182	7.16E-46
Zfp36l1	5.07E-142	-1.22042614	0.458	0.389	6.79E-138
Nr4a2	7.29E-95	-1.232547978	0.271	0.285	9.77E-91
Ddx6	1.60E-187	-1.235643581	0.458	0.398	2.14E-183
Tra2b	1.90E-209	-1.239491707	0.638	0.541	2.54E-205
Slfn5	7.01E-136	-1.24111793	0.401	0.366	9.38E-132
Tnfaip2	7.26E-129	-1.242596667	0.397	0.337	9.72E-125
Pmaip1	1.11E-136	-1.250874845	0.482	0.41	1.49E-132
Rnf149	5.97E-182	-1.260050377	0.812	0.699	7.99E-178
Kdm7a	1.29E-174	-1.265753894	0.468	0.42	1.73E-170
Ahnak	1.41E-216	-1.283521819	0.794	0.639	1.89E-212
Slc38a2	1.76E-167	-1.313407136	0.435	0.414	2.36E-163
Ccnl1	2.53E-215	-1.340740431	0.652	0.599	3.38E-211
Klf6	1.45E-198	-1.369260917	0.775	0.675	1.94E-194
Cxcr4	3.89E-184	-1.37086435	0.781	0.696	5.21E-180
Dusp1	7.94E-144	-1.372859845	0.947	0.893	1.06E-139
Ptgs2	1.49E-86	-1.409060964	0.362	0.297	2.00E-82
Btg1	1.26E-181	-1.436497714	0.992	0.967	1.69E-177
Mafb	6.28E-127	-1.448963102	0.462	0.398	8.41E-123
Tnfaip3	6.74E-170	-1.449979191	0.571	0.5	9.02E-166
Fosl2	5.25E-212	-1.452065624	0.731	0.648	7.02E-208
Marcks	4.82E-178	-1.462773614	0.593	0.539	6.45E-174
Cd44	6.73E-225	-1.46846343	0.889	0.795	9.00E-221
Irf2bp2	3.26E-194	-1.624312363	0.49	0.505	4.37E-190
Hspa1b	2.87E-82	-1.626382908	0.563	0.486	3.84E-78
Hbb-bs	3.53E-41	-1.807839436	0	0.181	4.72E-37
Thbs1	8.02E-148	-1.840110856	0.717	0.6	1.07E-143

Supplementary Table 7

gene	p_val	avg_log2FC	pct.1	pct.2	p_val_adj	cluster
Wfdc17	7.81E-210	1.812758304	0.961	0.852	1.04E-205	0-Gran-Wfdc17
Ifitm1	5.78E-95	1.562063477	0.74	0.557	7.74E-91	0-Gran-Wfdc17
Ccl6	2.65E-72	1.302762046	0.656	0.457	3.54E-68	0-Gran-Wfdc17
Retnlg	1.37E-186	1.266424996	0.985	0.915	1.83E-182	0-Gran-Wfdc17
Ifitm2	1.31E-74	1.245699758	0.681	0.519	1.75E-70	0-Gran-Wfdc17
Cxcl2	5.54E-101	1.214105081	0.853	0.716	7.42E-97	0-Gran-Wfdc17
Cd14	4.17E-45	1.20709872	0.54	0.371	5.58E-41	0-Gran-Wfdc17
Slpi	7.08E-50	1.070385981	0.63	0.505	9.47E-46	0-Gran-Wfdc17
Ftl1	1.49E-25	1.055597285	0.526	0.461	2.00E-21	0-Gran-Wfdc17
Alox5ap	2.79E-49	1.010523674	0.627	0.504	3.73E-45	0-Gran-Wfdc17
Marcksl1	6.85E-14	1.006626849	0.305	0.212	9.16E-10	0-Gran-Wfdc17
S100a6	1.61E-120	1.002007846	0.941	0.82	2.15E-116	0-Gran-Wfdc17
S100a11	2.94E-113	0.988737112	0.901	0.795	3.93E-109	0-Gran-Wfdc17
Clec4e	2.28E-33	0.986678762	0.529	0.369	3.05E-29	0-Gran-Wfdc17
Lrg1	1.79E-29	0.985561421	0.467	0.329	2.40E-25	0-Gran-Wfdc17
Acod1	1.80E-34	0.974054982	0.551	0.399	2.40E-30	0-Gran-Wfdc17
LOC100862	1.14E-17	0.964481631	0.472	0.429	1.53E-13	0-Gran-Wfdc17
Ifitm3	1.21E-51	0.961476647	0.675	0.538	1.62E-47	0-Gran-Wfdc17
Tspo	2.07E-39	0.926339542	0.583	0.453	2.77E-35	0-Gran-Wfdc17
Il1b	1.16E-112	0.910975522	0.967	0.912	1.55E-108	0-Gran-Wfdc17
Msrb1	2.84E-59	0.892793805	0.729	0.63	3.81E-55	0-Gran-Wfdc17
Wfdc21	4.54E-94	0.875072034	0.905	0.688	6.08E-90	0-Gran-Wfdc17
Nfkbiz	4.21E-26	0.86367421	0.612	0.521	5.64E-22	0-Gran-Wfdc17
Ccrl2	3.26E-11	0.863286304	0.313	0.233	4.37E-07	0-Gran-Wfdc17
Clec4d	4.15E-23	0.855631337	0.425	0.29	5.56E-19	0-Gran-Wfdc17
Atp6v1g1	1.22E-29	0.84605618	0.53	0.404	1.63E-25	0-Gran-Wfdc17
Nfkbia	1.89E-35	0.809039745	0.755	0.699	2.53E-31	0-Gran-Wfdc17
Cxcr2	4.63E-22	0.802077065	0.46	0.332	6.20E-18	0-Gran-Wfdc17
Marcks	6.91E-26	0.798426426	0.564	0.426	9.25E-22	0-Gran-Wfdc17
Il1f9	1.17E-12	0.791938016	0.356	0.267	1.57E-08	0-Gran-Wfdc17
Grina	3.98E-41	0.79162027	0.717	0.597	5.33E-37	0-Gran-Wfdc17
Csf3r	2.95E-44	0.774826834	0.752	0.608	3.94E-40	0-Gran-Wfdc17
Mmp8	4.43E-23	0.765604882	0.486	0.335	5.93E-19	0-Gran-Wfdc17
Mrpl33	3.49E-27	0.764674221	0.569	0.47	4.68E-23	0-Gran-Wfdc17
C5ar1	1.94E-13	0.738327589	0.341	0.245	2.59E-09	0-Gran-Wfdc17
Spi1	8.07E-19	0.730439275	0.489	0.39	1.08E-14	0-Gran-Wfdc17
S100a8	2.10E-127	0.724372928	0.999	0.991	2.81E-123	0-Gran-Wfdc17
Arg2	8.03E-14	0.720613744	0.259	0.154	1.07E-09	0-Gran-Wfdc17
Gda	5.43E-21	0.718335239	0.572	0.488	7.27E-17	0-Gran-Wfdc17
Trem1	1.05E-11	0.716322831	0.351	0.266	1.41E-07	0-Gran-Wfdc17
Hp	5.74E-37	0.716176879	0.689	0.588	7.68E-33	0-Gran-Wfdc17
Tyrobp	1.71E-60	0.70908868	0.849	0.812	2.29E-56	0-Gran-Wfdc17
S100a9	1.57E-144	0.706691731	1	0.999	2.11E-140	0-Gran-Wfdc17
Fcer1g	7.65E-22	0.698482905	0.591	0.538	1.02E-17	0-Gran-Wfdc17
Srgn	3.52E-68	0.697738543	0.889	0.834	4.72E-64	0-Gran-Wfdc17
App	1.52E-07	0.696070501	0.281	0.224	0.0020309	0-Gran-Wfdc17
Fbxl5	3.41E-14	0.693883251	0.415	0.328	4.56E-10	0-Gran-Wfdc17
Txn1	3.74E-26	0.687108531	0.598	0.502	5.00E-22	0-Gran-Wfdc17
Prr13	4.85E-21	0.681919721	0.523	0.422	6.49E-17	0-Gran-Wfdc17
G0s2	1.93E-32	0.673652578	0.822	0.756	2.58E-28	0-Gran-Wfdc17
Tgfbi	1.38E-11	0.673565356	0.321	0.233	1.85E-07	0-Gran-Wfdc17
Fth1	5.42E-49	0.668677985	0.934	0.955	7.25E-45	0-Gran-Wfdc17
St3gal4	2.95E-06	0.657601912	0.301	0.25	0.0395392	0-Gran-Wfdc17
Ier3	3.04E-09	0.652976067	0.33	0.258	4.07E-05	0-Gran-Wfdc17
Nlrp3	6.63E-07	0.634704391	0.252	0.191	0.00887349	0-Gran-Wfdc17

Fcgr3	1.54E-06	0.629345043	0.25	0.194	0.02065265	0-Gran-Wfdc17
Zyx	8.96E-12	0.626385799	0.446	0.37	1.20E-07	0-Gran-Wfdc17
Pygl	5.01E-06	0.61950493	0.257	0.207	0.06702458	0-Gran-Wfdc17
Ptafr	1.60E-06	0.613883029	0.269	0.214	0.02148359	0-Gran-Wfdc17
Tpd52	1.04E-21	0.609718614	0.568	0.454	1.40E-17	0-Gran-Wfdc17
R3hdm4	2.87E-07	0.60765939	0.314	0.258	0.00384269	0-Gran-Wfdc17
Hdc	1.08E-32	0.607038525	0.77	0.677	1.45E-28	0-Gran-Wfdc17
Pla2g7	1.30E-09	0.588051866	0.374	0.298	1.74E-05	0-Gran-Wfdc17
Lilrb4a	1.41E-08	0.584556194	0.355	0.291	0.00018911	0-Gran-Wfdc17
Trim30a	4.36E-06	0.58272873	0.336	0.297	0.05839549	0-Gran-Wfdc17
Taldo1	1.32E-09	0.581734568	0.371	0.305	1.77E-05	0-Gran-Wfdc17
Cebpb	8.35E-59	0.576450978	0.942	0.918	1.12E-54	0-Gran-Wfdc17
Slc16a3	4.75E-09	0.568287902	0.418	0.36	6.35E-05	0-Gran-Wfdc17
Lcp1	1.28E-14	0.55674989	0.584	0.534	1.71E-10	0-Gran-Wfdc17
Cd300lf	5.20E-07	0.549562015	0.378	0.335	0.00695492	0-Gran-Wfdc17
Gsr	1.96E-17	0.544105211	0.651	0.593	2.63E-13	0-Gran-Wfdc17
Mxd1	2.39E-17	0.5289851	0.689	0.649	3.20E-13	0-Gran-Wfdc17
Lilr4b	5.12E-08	0.525383174	0.402	0.349	0.00068478	0-Gran-Wfdc17
Lmnb1	1.00E-10	0.523162285	0.547	0.507	1.34E-06	0-Gran-Wfdc17
Gadd45a	5.48E-09	0.515332799	0.522	0.482	7.33E-05	0-Gran-Wfdc17
Pde4b	3.16E-05	0.512601591	0.31	0.27	0.42280234	0-Gran-Wfdc17
Il1r2	3.18E-07	0.511076215	0.377	0.316	0.00426046	0-Gran-Wfdc17
Lgals3	8.66E-07	0.510650071	0.326	0.274	0.01159103	0-Gran-Wfdc17
Adipor1	2.42E-05	0.509415611	0.315	0.267	0.32414755	0-Gran-Wfdc17
Sat1	4.79E-11	0.502008486	0.496	0.442	6.42E-07	0-Gran-Wfdc17
**gene**	**p_val**	**avg_log2FC**	**pct.1**	**pct.2**	**p_val_adj**	**cluster**
Gzma	3.65E-33	1.870460507	0.287	0.131	4.88E-29	1-Gran-Ets1
Hspa1b	1.21E-38	1.591840208	0.327	0.146	1.62E-34	1-Gran-Ets1
Satb1	3.32E-65	1.515202338	0.446	0.186	4.44E-61	1-Gran-Ets1
Ets1	4.61E-83	1.386577367	0.584	0.269	6.17E-79	1-Gran-Ets1
Gm25380	2.34E-62	1.38453932	0.45	0.204	3.13E-58	1-Gran-Ets1
Tpt1	6.31E-132	1.293855151	0.873	0.647	8.45E-128	1-Gran-Ets1
Wdr89	1.51E-180	1.271031774	0.956	0.837	2.02E-176	1-Gran-Ets1
Foxp1	1.17E-47	1.259152956	0.297	0.1	1.56E-43	1-Gran-Ets1
Il2rb	4.76E-39	1.194279814	0.307	0.125	6.37E-35	1-Gran-Ets1
Hsp90aa1	1.16E-39	1.171627165	0.419	0.216	1.56E-35	1-Gran-Ets1
Hspe1	1.31E-29	1.116883014	0.257	0.112	1.76E-25	1-Gran-Ets1
Ncl	1.12E-34	1.094701199	0.273	0.108	1.50E-30	1-Gran-Ets1
P2ry10	1.21E-38	1.086733024	0.289	0.108	1.62E-34	1-Gran-Ets1
Ahnak	5.26E-30	1.077200972	0.29	0.134	7.04E-26	1-Gran-Ets1
Rpl34-ps1	1.10E-71	1.061508624	0.75	0.523	1.47E-67	1-Gran-Ets1
Rpl35a	1.40E-31	1.056145038	0.274	0.117	1.87E-27	1-Gran-Ets1
Vps37b	1.87E-54	1.054862059	0.599	0.353	2.51E-50	1-Gran-Ets1
Rpl37a	3.85E-38	1.050316422	0.361	0.166	5.16E-34	1-Gran-Ets1
Rpl10	1.14E-54	1.043032231	0.652	0.427	1.52E-50	1-Gran-Ets1
Tmsb10	4.57E-104	1.028681886	0.888	0.704	6.12E-100	1-Gran-Ets1
Rpl8	7.80E-71	1.0193858	0.729	0.473	1.04E-66	1-Gran-Ets1
LOC105244	1.52E-38	1.003423968	0.479	0.275	2.04E-34	1-Gran-Ets1
Eef1b2	1.69E-27	1.00089997	0.298	0.148	2.26E-23	1-Gran-Ets1
Ccl5	9.94E-26	0.990348841	0.657	0.52	1.33E-21	1-Gran-Ets1
Rpl41	3.73E-202	0.986813099	0.997	0.976	4.99E-198	1-Gran-Ets1
Rpl19	5.68E-43	0.981134806	0.563	0.355	7.60E-39	1-Gran-Ets1
Rpl6	3.69E-30	0.97243112	0.416	0.248	4.94E-26	1-Gran-Ets1
Elf1	8.22E-34	0.971588955	0.253	0.093	1.10E-29	1-Gran-Ets1
Ptma	1.07E-68	0.961211618	0.762	0.518	1.44E-64	1-Gran-Ets1
Peli1	2.45E-30	0.951750781	0.313	0.146	3.28E-26	1-Gran-Ets1
Eef1a1	2.52E-66	0.949993504	0.765	0.538	3.37E-62	1-Gran-Ets1
Hsp90ab1	2.15E-38	0.948120118	0.5	0.29	2.88E-34	1-Gran-Ets1

LOC108167	7.52E-77	0.932393146	0.829	0.607	1.01E-72	1-Gran-Ets1
Zfp36l2	4.15E-39	0.92206721	0.552	0.334	5.55E-35	1-Gran-Ets1
Rgs1	7.15E-17	0.918191183	0.254	0.143	9.57E-13	1-Gran-Ets1
Rpl31-ps12	3.43E-36	0.905948043	0.535	0.343	4.60E-32	1-Gran-Ets1
Rpl4	1.30E-32	0.887059564	0.537	0.367	1.74E-28	1-Gran-Ets1
Rpl37rt	1.21E-100	0.862581538	0.943	0.866	1.62E-96	1-Gran-Ets1
Hspa1a	1.66E-14	0.860057457	0.36	0.251	2.23E-10	1-Gran-Ets1
Eef2	1.70E-47	0.85103153	0.661	0.436	2.28E-43	1-Gran-Ets1
H2-K1	2.32E-61	0.834518916	0.813	0.612	3.10E-57	1-Gran-Ets1
Pabpc1	8.49E-26	0.816406767	0.429	0.265	1.14E-21	1-Gran-Ets1
Eef1g	3.94E-22	0.815961878	0.294	0.156	5.27E-18	1-Gran-Ets1
Rpl15	4.50E-30	0.810581845	0.525	0.349	6.02E-26	1-Gran-Ets1
Eif2s2	1.23E-19	0.809810037	0.252	0.129	1.64E-15	1-Gran-Ets1
Rpl36al	5.81E-28	0.809350953	0.487	0.32	7.78E-24	1-Gran-Ets1
Irf2bp2	1.48E-20	0.809275195	0.317	0.177	1.99E-16	1-Gran-Ets1
Hspa8	1.12E-45	0.799995598	0.735	0.547	1.50E-41	1-Gran-Ets1
Rplp0	7.38E-27	0.791284642	0.518	0.348	9.88E-23	1-Gran-Ets1
Slc38a2	2.00E-22	0.789755912	0.371	0.217	2.68E-18	1-Gran-Ets1
Rpl7	3.55E-22	0.779822942	0.453	0.312	4.75E-18	1-Gran-Ets1
Luc7l2	3.49E-24	0.77438629	0.376	0.212	4.67E-20	1-Gran-Ets1
Ifngr1	3.42E-21	0.773051128	0.363	0.215	4.58E-17	1-Gran-Ets1
Hnrnph1	1.73E-19	0.771382944	0.312	0.178	2.32E-15	1-Gran-Ets1
Crem	1.97E-15	0.770949893	0.253	0.145	2.63E-11	1-Gran-Ets1
Irf8	7.59E-12	0.767189923	0.27	0.176	1.02E-07	1-Gran-Ets1
Hnrnpf	3.94E-24	0.764629497	0.395	0.232	5.27E-20	1-Gran-Ets1
Uba52	1.22E-43	0.729607869	0.781	0.581	1.63E-39	1-Gran-Ets1
Pcbp1	6.95E-15	0.724896063	0.26	0.152	9.30E-11	1-Gran-Ets1
Hsp90b1	7.02E-17	0.72467231	0.325	0.2	9.40E-13	1-Gran-Ets1
Ppia	3.14E-26	0.721319799	0.52	0.342	4.20E-22	1-Gran-Ets1
Atp1b3	9.83E-20	0.71656393	0.366	0.224	1.32E-15	1-Gran-Ets1
Cd74	4.91E-16	0.715459028	0.706	0.584	6.57E-12	1-Gran-Ets1
Shisa5	4.79E-22	0.696876946	0.489	0.339	6.41E-18	1-Gran-Ets1
Srrm2	3.59E-16	0.692043453	0.296	0.174	4.81E-12	1-Gran-Ets1
Uqcrh	1.35E-28	0.686948318	0.624	0.465	1.80E-24	1-Gran-Ets1
Jak1	1.30E-20	0.677459768	0.361	0.209	1.73E-16	1-Gran-Ets1
Rbm39	6.20E-28	0.660139077	0.588	0.403	8.30E-24	1-Gran-Ets1
Rps27a	3.23E-49	0.658640852	0.871	0.771	4.33E-45	1-Gran-Ets1
H2-Eb1	1.65E-08	0.65573919	0.313	0.241	0.00022088	1-Gran-Ets1
Gpr132	4.38E-16	0.649176293	0.314	0.19	5.87E-12	1-Gran-Ets1
Ifrd1	2.88E-13	0.641038355	0.351	0.237	3.86E-09	1-Gran-Ets1
Hnrnpdl	2.50E-11	0.640096572	0.26	0.166	3.34E-07	1-Gran-Ets1
Cxcr4	5.53E-28	0.63273836	0.623	0.432	7.40E-24	1-Gran-Ets1
H2-Aa	0.000108896	0.629393678	0.278	0.235	1	1-Gran-Ets1
Dnaja1	4.35E-12	0.622543619	0.293	0.191	5.83E-08	1-Gran-Ets1
Dnajb1	1.11E-08	0.620639535	0.257	0.181	0.00014903	1-Gran-Ets1
Dennd4a	6.67E-23	0.609672247	0.534	0.35	8.93E-19	1-Gran-Ets1
Rpl14	9.00E-18	0.6047471	0.537	0.402	1.20E-13	1-Gran-Ets1
Pcbp2	4.80E-18	0.602850047	0.434	0.287	6.42E-14	1-Gran-Ets1
Hnrnpa3	1.86E-10	0.591756162	0.282	0.19	2.49E-06	1-Gran-Ets1
Atp5h	9.89E-12	0.589116773	0.344	0.245	1.32E-07	1-Gran-Ets1
Atp2b1	2.56E-13	0.578020171	0.252	0.145	3.43E-09	1-Gran-Ets1
Fam107b	1.62E-09	0.570360013	0.27	0.185	2.16E-05	1-Gran-Ets1
Ptpn18	1.11E-10	0.562284047	0.291	0.201	1.49E-06	1-Gran-Ets1
Jun	1.28E-13	0.556986719	0.459	0.339	1.72E-09	1-Gran-Ets1
Ezr	4.25E-12	0.553711994	0.274	0.17	5.69E-08	1-Gran-Ets1
Nr4a1	6.61E-14	0.535955667	0.479	0.352	8.85E-10	1-Gran-Ets1
Srsf3	4.81E-10	0.531997124	0.319	0.224	6.44E-06	1-Gran-Ets1
Rpl29	9.82E-11	0.504779496	0.382	0.281	1.32E-06	1-Gran-Ets1

Fus	5.80E-09	0.501027909	0.262	0.177	7.77E-05	1-Gran-Ets1
**gene**	**p_val**	**avg_log2FC**	**pct.1**	**pct.2**	**p_val_adj**	**cluster**
Camp	0	6.024771078	0.956	0.117	0	2-Gran-Camp
Ngp	1.55E-251	5.325600716	0.99	0.246	2.07E-247	2-Gran-Camp
Ltf	0	4.9152343	0.879	0.038	0	2-Gran-Camp
Chil3	5.03E-244	3.908792505	0.717	0.069	6.73E-240	2-Gran-Camp
Cd177	1.51E-202	2.34750646	0.761	0.088	2.02E-198	2-Gran-Camp
Ifitm6	2.25E-135	2.213032705	0.879	0.234	3.02E-131	2-Gran-Camp
Lyz2	5.66E-109	2.087901842	0.845	0.244	7.58E-105	2-Gran-Camp
Cybb	6.83E-146	2.073187354	0.737	0.127	9.15E-142	2-Gran-Camp
Adpgk	4.95E-169	2.06174612	0.626	0.069	6.63E-165	2-Gran-Camp
Lcn2	8.61E-123	1.943212622	0.983	0.543	1.15E-118	2-Gran-Camp
Serpinb1a	8.13E-155	1.895281365	0.431	0.029	1.09E-150	2-Gran-Camp
I830127L07I	2.05E-131	1.857587894	0.428	0.038	2.74E-127	2-Gran-Camp
Anxa1	5.92E-103	1.733274059	0.896	0.316	7.92E-99	2-Gran-Camp
Syne1	8.87E-151	1.719764631	0.475	0.038	1.19E-146	2-Gran-Camp
Ckap4	2.38E-118	1.67997066	0.498	0.062	3.18E-114	2-Gran-Camp
Tkt	1.81E-92	1.595150648	0.815	0.235	2.42E-88	2-Gran-Camp
Aldh2	1.99E-109	1.565906572	0.643	0.116	2.67E-105	2-Gran-Camp
Hmgn2	1.30E-61	1.559774832	0.559	0.155	1.74E-57	2-Gran-Camp
Dstn	2.20E-91	1.476586468	0.673	0.15	2.95E-87	2-Gran-Camp
AA467197	9.77E-66	1.25841637	0.37	0.062	1.31E-61	2-Gran-Camp
St3gal5	2.52E-63	1.146846849	0.303	0.042	3.37E-59	2-Gran-Camp
Pglyrp1	2.55E-53	1.102024406	0.919	0.473	3.41E-49	2-Gran-Camp
Cpne3	1.52E-64	1.090543666	0.37	0.062	2.04E-60	2-Gran-Camp
Arhgdib	5.57E-43	1.080145761	0.768	0.338	7.46E-39	2-Gran-Camp
S100a8	5.28E-89	1.057069995	1	0.996	7.07E-85	2-Gran-Camp
Lims1	2.79E-64	1.055469681	0.401	0.072	3.73E-60	2-Gran-Camp
H2afz	6.67E-26	0.959305475	0.673	0.345	8.93E-22	2-Gran-Camp
Npepps	5.84E-49	0.957175562	0.313	0.058	7.82E-45	2-Gran-Camp
Clec12a	4.21E-51	0.946347718	0.286	0.047	5.63E-47	2-Gran-Camp
Aprt	1.98E-53	0.943347807	0.357	0.069	2.66E-49	2-Gran-Camp
Plbd1	1.03E-40	0.936840786	0.273	0.054	1.38E-36	2-Gran-Camp
Ly6c2	6.94E-39	0.906934829	0.62	0.226	9.29E-35	2-Gran-Camp
Wfdc21	4.71E-54	0.901408667	1	0.797	6.30E-50	2-Gran-Camp
Scp2	4.27E-44	0.89806812	0.424	0.109	5.72E-40	2-Gran-Camp
Ltb4r1	2.11E-47	0.889782203	0.34	0.07	2.82E-43	2-Gran-Camp
Degs1	1.84E-44	0.886924052	0.411	0.102	2.47E-40	2-Gran-Camp
Mpc2	1.03E-45	0.875730946	0.293	0.055	1.37E-41	2-Gran-Camp
Clec4a2	2.94E-54	0.865862426	0.263	0.037	3.93E-50	2-Gran-Camp
Thbs1	6.79E-20	0.863521639	0.569	0.281	9.10E-16	2-Gran-Camp
C3	3.88E-50	0.850127203	0.3	0.052	5.19E-46	2-Gran-Camp
Hmgb2	8.26E-20	0.842864493	0.879	0.563	1.11E-15	2-Gran-Camp
S100a9	2.65E-85	0.842564024	1	1	3.55E-81	2-Gran-Camp
Mmp9	3.23E-21	0.837467711	0.741	0.438	4.33E-17	2-Gran-Camp
Mettl9	1.29E-39	0.831911349	0.364	0.091	1.73E-35	2-Gran-Camp
Plscr1	6.25E-40	0.816234921	0.357	0.086	8.37E-36	2-Gran-Camp
Mgst1	3.34E-34	0.795833976	0.549	0.195	4.47E-30	2-Gran-Camp
1810037I17I	2.16E-27	0.737996381	0.636	0.271	2.89E-23	2-Gran-Camp
Itgam	7.49E-38	0.725323258	0.522	0.163	1.00E-33	2-Gran-Camp
Cyba	3.56E-27	0.724456643	0.848	0.459	4.76E-23	2-Gran-Camp
Ncf1	1.59E-33	0.702943931	0.737	0.305	2.12E-29	2-Gran-Camp
Arrb2	8.47E-29	0.659909806	0.418	0.136	1.13E-24	2-Gran-Camp
Lamtor4	2.66E-27	0.646826034	0.37	0.118	3.56E-23	2-Gran-Camp
Flna	2.71E-27	0.638494866	0.492	0.181	3.63E-23	2-Gran-Camp
Mmp8	2.65E-13	0.628689575	0.633	0.403	3.55E-09	2-Gran-Camp
Cd24a	5.97E-26	0.590198223	0.529	0.202	7.99E-22	2-Gran-Camp
Glrx	1.61E-21	0.578305777	0.364	0.132	2.15E-17	2-Gran-Camp

LOC102635	6.65E-25	0.569588546	0.599	0.246	8.90E-21	2-Gran-Camp
Lasp1	2.01E-22	0.566626528	0.343	0.116	2.69E-18	2-Gran-Camp
Uqcrb	3.52E-28	0.564888163	0.266	0.067	4.71E-24	2-Gran-Camp
Ndufv3	4.19E-28	0.556423302	0.32	0.091	5.61E-24	2-Gran-Camp
Ndufa1	1.55E-28	0.542787733	0.283	0.074	2.08E-24	2-Gran-Camp
Cox7a2	1.39E-23	0.542743067	0.438	0.162	1.86E-19	2-Gran-Camp
Fam101b	3.94E-23	0.528789386	0.256	0.073	5.27E-19	2-Gran-Camp
Ap3s1	1.18E-27	0.511415635	0.306	0.086	1.59E-23	2-Gran-Camp
Ndufb7	8.17E-31	0.506804457	0.343	0.095	1.09E-26	2-Gran-Camp

Supplementary Table 8

gene	avg_log2FC	pct.1	pct.2	p_val_adj
LOC1008(	2.405264193	1	0.392	1.25E-229
Ftl1	2.340705307	0.997	0.443	8.72E-234
Lyz2	2.309961118	0.943	0.225	3.26E-157
Jchain	2.002884576	0.902	0.17	2.65E-160
Apoe	1.946959917	0.738	0.085	1.02E-158
Ifitm2	1.885156354	1	0.571	3.52E-183
Fcer1g	1.780677373	1	0.52	1.78E-167
Sh3bgrl3	1.734277193	0.952	0.296	3.06E-129
Ubb	1.529371533	0.994	0.595	1.10E-143
Alox5ap	1.497026779	0.991	0.529	1.87E-119
Lgals3	1.493916279	0.875	0.239	1.20E-116
Slpi	1.467857576	0.982	0.533	1.77E-92
Ifitm1	1.440631471	0.967	0.63	2.13E-66
Elane	1.437457558	0.307	0.013	3.51E-77
Ly6d	1.410820683	0.652	0.112	2.41E-117
Ppib	1.379302199	0.446	0.045	1.12E-93
Ccl9	1.358615804	0.354	0.027	2.50E-78
Plac8	1.344395021	0.917	0.317	2.64E-101
Hilpda	1.313469269	0.432	0.091	8.94E-54
Emd	1.292890444	0.836	0.269	4.34E-91
Mzb1	1.292676547	0.42	0.043	1.00E-91
D8Ertd738	1.27358818	0.771	0.191	1.78E-116
Prtn3	1.26947104	0.286	0.018	2.62E-63
Igfbp6	1.264499824	0.429	0.064	3.78E-78
Lgals1	1.24118333	0.667	0.136	2.87E-109
Fth1	1.23752829	1	0.936	4.38E-105
Manf	1.233061981	0.372	0.041	3.09E-71
Cd14	1.199436841	0.896	0.422	4.67E-62
Ly6c2	1.168045932	0.744	0.206	4.36E-95
Tspo	1.167103452	0.952	0.481	4.86E-74
Cst3	1.148951786	0.896	0.322	4.04E-95
Ctss	1.14580051	0.568	0.102	1.27E-105
Ifitm3	1.134831475	0.994	0.576	3.50E-81
Myl12b	1.116907215	0.646	0.137	1.75E-109
Vamp8	1.112092222	0.696	0.175	8.56E-102
S100a10	1.10683691	0.646	0.147	6.31E-102
Arpc1b	1.086738146	0.738	0.235	1.05E-80
Cyba	1.080107415	0.946	0.443	1.38E-75
Cd52	1.066995527	0.988	0.635	3.16E-76
Cycs	1.061573647	0.464	0.086	4.23E-73
Rpl14	1.060028579	0.908	0.393	1.02E-74
Ppp1ca	1.04751004	0.482	0.097	4.25E-76
Rpl29	1.047368546	0.786	0.26	3.68E-87
Fabp5	1.034696975	0.375	0.065	9.16E-64
Msrb1	1.032987035	0.994	0.653	1.45E-70
Rgs10	1.01873785	0.31	0.032	2.77E-64
Ctsz	1.016582512	0.512	0.106	4.93E-81
Btf3	1.009227709	0.682	0.177	6.26E-104
Ccl6	1.008103578	0.958	0.53	8.40E-60
Eno1	1.005389759	0.393	0.065	3.37E-67
Ppia	1.004884537	0.896	0.342	1.60E-92
Ifi30	1.00407237	0.274	0.027	6.33E-55
Npc2	1.003440556	0.723	0.204	3.94E-102
Brk1	0.999414239	0.366	0.05	1.21E-72
Rpl15	0.994240711	0.911	0.348	3.28E-97

LOC1052	0.988085676	0.792	0.289	1.38E-78
Eif4ebp1	0.983788984	0.375	0.054	9.72E-76
Ctsh	0.982549814	0.402	0.055	5.54E-81
Lrg1	0.975864924	0.872	0.358	9.50E-77
Vim	0.970658391	0.488	0.099	1.85E-80
Gm13202	0.967334212	0.396	0.071	1.35E-63
Tyrobp	0.965948997	1	0.815	8.76E-80
Gpx1	0.964879234	0.848	0.314	8.43E-87
H3f3c	0.954492492	0.286	0.034	2.96E-56
Cstb	0.949135944	0.455	0.087	9.93E-81
Sepw1	0.945721448	0.622	0.16	3.67E-94
Prdx1	0.943544051	0.5	0.106	3.29E-83
H2-Ab1	0.939731863	0.845	0.319	5.48E-83
Arhgdib	0.934704513	0.863	0.322	4.06E-95
Taldo1	0.93031205	0.818	0.29	1.74E-92
Pfdn5	0.920810767	0.812	0.303	1.01E-81
Oaz1	0.917467301	0.952	0.439	1.03E-81
Cdk2ap2	0.910550399	0.732	0.257	6.48E-76
Atp6v1f	0.906754683	0.548	0.126	8.27E-89
Uba52	0.903791498	0.988	0.606	8.58E-64
Ldha	0.898245477	0.53	0.134	3.24E-74
Supt4b	0.897545845	0.298	0.043	1.89E-52
Gpx4	0.895723677	0.497	0.115	8.81E-83
S100a11	0.892746398	1	0.841	1.44E-64
Card19	0.884335505	0.351	0.059	1.04E-66
Arpc3	0.884191144	0.845	0.324	1.36E-85
Myl12a	0.8820645	0.595	0.16	1.77E-83
Ramp1	0.881665934	0.274	0.033	1.97E-52
Rplp0	0.878605253	0.854	0.351	6.88E-77
Psmb8	0.878544178	0.485	0.103	1.26E-82
15-Sep	0.871155989	0.265	0.033	1.57E-47
Eif1	0.870750133	0.988	0.649	5.67E-58
Ccl5	0.857292348	0.982	0.516	3.30E-74
Gabarap	0.856976251	0.893	0.353	9.97E-92
Hint1	0.855688541	0.56	0.143	4.26E-84
Supt4a	0.85480796	0.628	0.172	2.24E-94
Fis1	0.854759909	0.631	0.169	4.11E-98
Cmtm7	0.850186433	0.366	0.076	3.42E-55
Bcl2a1b	0.848079972	0.286	0.072	2.87E-33
Ly6g	0.843749796	0.411	0.105	5.29E-55
Cxcl2	0.840987622	0.979	0.776	2.82E-27
Atp5d	0.831785623	0.44	0.091	1.23E-77
Dynll1	0.83100574	0.518	0.151	2.25E-61
Rnasek	0.830079276	0.435	0.1	6.20E-69
Tmsb4x	0.825851014	1	0.998	1.51E-127
Gm5150	0.821564107	0.274	0.042	9.63E-52
Ssr2	0.818842763	0.199	0.017	4.51E-38
Prdx2	0.806456532	0.265	0.038	3.62E-47
Cd74	0.804502545	0.979	0.583	4.19E-61
Map1lc3b	0.802729517	0.818	0.318	2.55E-87
Pfn1	0.801686954	0.982	0.619	3.29E-54
H2afz	0.797846071	0.789	0.328	3.98E-68
Psmb2	0.794337812	0.22	0.029	1.52E-37
Sod2	0.794113347	0.33	0.062	1.33E-53
LOC1026	0.787244109	0.667	0.234	1.01E-68
Uqcrfs1	0.784273633	0.271	0.051	1.11E-37
Rac2	0.781161466	0.833	0.381	1.21E-62
Eif5a	0.780337853	0.518	0.129	7.39E-83

Park7	0.776277037	0.369	0.067	4.77E-66
Ypel3	0.774091011	0.497	0.132	6.57E-75
Myl6	0.771968675	0.753	0.295	8.84E-73
Camp	0.771423418	0.494	0.158	1.85E-59
Pycard	0.770501776	0.369	0.08	1.57E-58
Chil3	0.768793842	0.381	0.099	2.33E-46
Cnbp	0.765201025	0.482	0.12	8.94E-76
Pglyrp1	0.761464395	0.911	0.468	3.45E-59
Itm2b	0.756377127	0.836	0.367	2.44E-70
Fam96a	0.755973377	0.241	0.03	8.64E-43
Slc25a5	0.752959976	0.449	0.108	8.25E-70
Sumo2	0.752298505	0.336	0.071	4.98E-54
Edf1	0.751511395	0.307	0.054	6.13E-54
Nedd8	0.751064181	0.387	0.083	6.16E-64
Clta	0.743651407	0.562	0.168	3.40E-76
Gngt2	0.741459216	0.333	0.06	3.66E-60
Ms4a6c	0.739826604	0.324	0.057	1.84E-58
Prelid1	0.73933762	0.345	0.075	6.79E-54
Cdkn2d	0.73353788	0.518	0.128	6.03E-89
Rps2	0.732051365	0.423	0.117	3.91E-54
Retnlg	0.730280863	1	0.951	3.65E-28
Sdf2l1	0.729708527	0.17	0.013	5.13E-32
Lamtor5	0.727702235	0.247	0.035	9.11E-43
Eno1b	0.725527356	0.164	0.011	1.70E-33
Serf2	0.724767623	0.943	0.514	2.53E-58
Ccdc12	0.723411665	0.461	0.116	9.78E-77
Banf1	0.722827412	0.211	0.033	4.45E-32
Atp5f1	0.721376949	0.443	0.12	1.20E-59
Ctsb	0.720793207	0.589	0.191	4.42E-76
Hcfc1r1	0.717189244	0.19	0.024	1.80E-32
Mien1	0.716246718	0.265	0.046	1.63E-43
Med28	0.715581351	0.247	0.036	6.78E-45
Ube2s	0.714798545	0.461	0.12	2.65E-69
Ctsg	0.714589403	0.119	0.003	3.65E-29
Sri	0.712650353	0.435	0.109	2.68E-64
Tmem134	0.711995725	0.223	0.031	2.61E-38
Cox6b1	0.710334631	0.551	0.158	7.42E-78
Hp	0.707470897	0.961	0.612	1.21E-43
Spcs1	0.70633087	0.31	0.07	9.58E-43
Higd2a	0.703793095	0.292	0.055	1.27E-49
Gng10	0.703671997	0.289	0.051	5.00E-46
Clic1	0.702240795	0.622	0.208	2.33E-77
Dynlt1f	0.700640457	0.176	0.015	8.81E-35
Dctn3	0.697557015	0.315	0.07	3.57E-45
Aldoa	0.69364295	0.625	0.188	4.23E-97
I830127L(	0.68816356	0.223	0.056	1.45E-20
Psenen	0.686697528	0.381	0.086	2.01E-67
Timm10b	0.683330488	0.458	0.121	1.97E-69
Rac1	0.682594274	0.598	0.194	3.58E-81
Cks2	0.682507549	0.443	0.146	1.54E-47
Pdia3	0.680693861	0.36	0.078	3.65E-60
Ly6a	0.680364065	0.268	0.046	1.67E-46
Nutf2	0.680106126	0.152	0.011	1.55E-29
Marcksl1	0.679576867	0.571	0.232	4.35E-47
Swi5	0.679537469	0.277	0.051	5.25E-45
Mrpl14	0.677714219	0.271	0.05	1.42E-42
Ssna1	0.674052446	0.176	0.023	1.85E-27
Lsm10	0.671118096	0.149	0.014	3.85E-27

Gng5	0.665224614	0.17	0.022	1.18E-26
Hmgn2	0.665055683	0.476	0.159	3.94E-55
Ostf1	0.662423429	0.661	0.219	4.45E-89
Ninj1	0.661579689	0.39	0.099	4.79E-66
H2afx	0.659500729	0.14	0.012	8.12E-24
Mrfap1	0.651927559	0.354	0.083	4.15E-55
Gmfg	0.651687477	0.759	0.295	1.05E-85
Stmn1	0.651456281	0.244	0.049	1.13E-33
Ms4a4c	0.647630295	0.176	0.021	1.78E-31
Sys1	0.641763521	0.244	0.039	1.16E-41
Birc5	0.640039578	0.152	0.017	1.42E-23
Sec11c	0.638620185	0.342	0.078	2.27E-53
Lrrc58	0.637488708	0.235	0.042	2.59E-35
Asnsd1	0.631418761	0.226	0.041	5.94E-34
1110008F	0.630907171	0.39	0.102	2.32E-58
Lsm4	0.629900452	0.324	0.073	6.61E-51
Coa3	0.627456448	0.196	0.025	3.64E-36
Atp6v0e	0.626439329	0.56	0.188	5.66E-66
Saa3	0.625211925	0.161	0.033	5.01E-19
S100a1	0.622916026	0.131	0.009	8.50E-24
Stra13	0.622566458	0.179	0.022	1.95E-30
Atpif1	0.61936974	0.357	0.084	2.72E-55
Erh	0.614969207	0.33	0.075	1.88E-51
Dad1	0.614699373	0.375	0.088	1.11E-63
Ccdc124	0.614663742	0.211	0.032	5.68E-37
Nt5c	0.611296981	0.205	0.029	2.43E-36
Rgcc	0.607548427	0.241	0.048	7.51E-37
B2m	0.606687428	0.982	0.693	1.41E-39
Rpl19	0.604097299	0.833	0.375	1.34E-76
Spcs2	0.602868754	0.298	0.068	2.40E-43
Cdc37	0.600586488	0.223	0.046	1.34E-31
Rab24	0.600341518	0.321	0.072	3.39E-55
Snapc5	0.600018404	0.149	0.016	6.36E-26
Tceb2	0.596554346	0.408	0.107	2.24E-61
H2-D1	0.595846849	0.979	0.688	9.23E-38
Phospho1	0.594270241	0.185	0.029	6.46E-35
Phf5a	0.594111731	0.232	0.049	2.11E-33
Creg1	0.593397025	0.259	0.057	5.18E-40
Bst2	0.592181673	0.387	0.102	2.24E-60
H2-Aa	0.590361901	0.625	0.206	8.16E-83
Selk	0.585950556	0.664	0.256	3.26E-72
H2-Q10	0.585827233	0.292	0.083	1.41E-39
Iscu	0.585188077	0.247	0.049	1.15E-41
Tmem176	0.584099106	0.134	0.014	8.28E-22
Sra1	0.58324038	0.214	0.034	1.81E-39
1110008P	0.582302297	0.196	0.032	5.85E-32
Cuedc2	0.579313099	0.158	0.022	3.40E-23
Amica1	0.578198882	0.298	0.075	8.04E-48
Pkig	0.577866597	0.182	0.026	3.64E-31
Srp14	0.576772059	0.414	0.111	8.09E-61
Ap2s1	0.576659113	0.318	0.075	1.09E-50
Pold4	0.576447463	0.211	0.037	8.46E-35
Napsa	0.575877439	0.265	0.051	9.08E-46
Dph3	0.574615809	0.277	0.057	1.06E-48
Esd	0.572718765	0.295	0.08	3.69E-39
Rps6	0.572624248	0.247	0.065	1.20E-28
Dynlrb1	0.571831847	0.289	0.065	1.32E-43
Upp1	0.571197827	0.193	0.039	7.73E-29

S100a4	0.57092648	0.509	0.134	3.93E-92
Sptssa	0.570050955	0.229	0.041	2.58E-35
G0s2	0.569548645	0.982	0.773	2.36E-23
Eif3h	0.566092515	0.372	0.099	9.44E-53
Cxcl3	0.565741765	0.131	0.028	1.31E-16
Ndufs4	0.562351507	0.176	0.025	4.39E-27
Mdh2	0.562224883	0.289	0.064	1.10E-42
Rheb	0.559788379	0.247	0.055	1.93E-38
Pomp	0.559658108	0.429	0.132	9.53E-55
Dhrs7	0.559239874	0.342	0.086	2.89E-54
Cd63	0.559090208	0.137	0.017	1.64E-19
Gm5621	0.557545934	0.208	0.047	1.61E-25
Tagln2	0.557092326	0.542	0.193	4.25E-68
Cfl1	0.556984602	0.896	0.458	1.11E-65
Mgst1	0.551475935	0.515	0.194	3.89E-51
Tm2d2	0.550526441	0.176	0.022	7.76E-32
Ppdpf	0.548234143	0.164	0.025	2.53E-25
Dbi	0.547841281	0.307	0.079	8.92E-40
Cks1b	0.547526786	0.143	0.016	2.14E-23
Id1	0.545061348	0.167	0.036	2.79E-21
Emp3	0.544785215	0.304	0.078	1.11E-42
Mcemp1	0.544389555	0.741	0.319	1.26E-74
Plp2	0.54398017	0.348	0.085	2.30E-60
Nxt1	0.541173075	0.14	0.018	1.24E-20
Mrpl20	0.541139252	0.232	0.048	7.18E-35
Ctsl	0.541070519	0.244	0.046	1.72E-42
Ybx1	0.540968526	0.292	0.076	2.50E-38
Ifi27l2a	0.539806561	0.786	0.381	1.21E-55
Unc119	0.53912614	0.259	0.066	8.12E-36
Pilrb2	0.538505509	0.173	0.029	1.83E-28
Pfdn1	0.53802742	0.131	0.016	3.43E-20
Psmb5	0.537356417	0.277	0.065	5.45E-41
Cib1	0.537033921	0.193	0.038	2.20E-27
Tnfaip8l2	0.532842936	0.152	0.021	1.67E-24
Timm13	0.532193623	0.435	0.126	5.90E-61
Chil1	0.531970493	0.369	0.109	1.83E-48
Akr1a1	0.531491157	0.128	0.019	4.42E-16
Anxa5	0.530642876	0.211	0.045	8.88E-30
Metrnl	0.529351259	0.307	0.078	1.36E-49
Cox8a	0.528766034	0.786	0.352	2.43E-77
Bax	0.527129782	0.271	0.057	2.21E-45
Bloc1s2	0.525387543	0.214	0.04	7.32E-38
Trappc5	0.525049812	0.196	0.038	8.16E-30
Gadd45g	0.524974163	0.247	0.061	2.76E-34
Glipr1	0.523983679	0.244	0.049	2.13E-43
Timm17b	0.52382438	0.143	0.019	2.46E-22
Limd2	0.523532138	0.473	0.148	5.72E-65
Rnase6	0.523471218	0.164	0.025	1.72E-25
Gpsm3	0.522227644	0.571	0.2	1.06E-75
Gemin7	0.520032754	0.164	0.023	1.23E-27
Sumo1	0.518094262	0.354	0.096	3.82E-54
Clec4a3	0.517621739	0.128	0.013	1.22E-21
Ostc	0.51485348	0.217	0.053	1.41E-28
Rabac1	0.514774437	0.438	0.137	3.07E-64
Cox5a	0.512678781	0.211	0.048	5.50E-27
Lamtor4	0.506539113	0.372	0.115	6.76E-47
Gm9733	0.506464189	0.137	0.022	2.70E-20
Snrpd2	0.504565708	0.241	0.053	3.34E-37

Atraid	0.503781735	0.122	0.015	6.90E-20
Gng12	0.503665591	0.28	0.063	7.04E-50
Snrpc	0.501257142	0.19	0.032	6.13E-32
Syk	-0.510625758	0.321	0.232	6.24E-39
Iqsec1	-0.51320316	0.101	0.086	8.61E-14
Diaph1	-0.514839625	0.173	0.121	1.05E-25
Dmxl2	-0.514953088	0.122	0.097	1.13E-14
Coq10b	-0.516817159	0.378	0.247	9.19E-59
Tes	-0.518019684	0.119	0.095	9.67E-15
Myadm	-0.518262193	0.199	0.135	3.83E-31
Ppp1cb	-0.519255987	0.205	0.15	5.32E-24
Mrpl52	-0.51934659	0.14	0.114	1.49E-15
Mapk1	-0.519373006	0.155	0.119	1.23E-21
Hnrnpf	-0.521297488	0.372	0.274	5.62E-49
LOC1026:	-0.521798945	0.161	0.126	6.91E-20
Puf60	-0.522933693	0.116	0.099	6.34E-14
Skil	-0.523296762	0.134	0.114	3.95E-15
Brd4	-0.526283967	0.11	0.089	5.22E-14
Slc40a1	-0.526340981	0.107	0.091	8.28E-11
Ptafr	-0.528391268	0.321	0.238	2.51E-34
Adam8	-0.53142474	0.324	0.221	3.51E-44
Hspd1	-0.534856817	0.101	0.087	1.69E-13
Etf1	-0.535086953	0.128	0.102	2.45E-16
Ptges3	-0.535130643	0.11	0.09	1.71E-13
Arhgap30	-0.535212351	0.199	0.155	3.24E-24
Pla2g7	-0.535815579	0.461	0.329	4.92E-59
Pbxip1	-0.535968084	0.179	0.132	9.59E-24
Adgre5	-0.537011868	0.417	0.293	2.54E-53
Akap13	-0.538041954	0.241	0.18	1.18E-33
Arl6ip1	-0.541068587	0.25	0.18	2.59E-36
Pak2	-0.541363381	0.214	0.146	9.70E-34
Ctnnb1	-0.541448074	0.122	0.101	1.20E-16
Icam1	-0.541465652	0.11	0.089	1.60E-11
Slc44a2	-0.54327064	0.152	0.122	4.71E-18
Rps15	-0.54418157	0.679	0.476	7.31E-78
Dclre1c	-0.546810573	0.104	0.084	5.05E-12
Itgam	-0.54698226	0.262	0.188	3.91E-34
Larp4b	-0.548246259	0.205	0.152	1.16E-27
Ppp2r5c	-0.548578946	0.11	0.095	3.78E-13
Trim25	-0.550876827	0.193	0.139	1.68E-25
Herpud1	-0.553203065	0.274	0.184	4.62E-41
Ywhab	-0.553470116	0.182	0.138	1.25E-26
Srsf2	-0.554188159	0.247	0.172	6.91E-36
Tgif1	-0.555484844	0.196	0.146	3.45E-26
Mmp8	-0.556101592	0.577	0.406	2.64E-56
Rnf114	-0.556637922	0.161	0.125	2.42E-21
Cox17	-0.556793393	0.604	0.431	7.09E-67
Gnai3	-0.557708452	0.173	0.131	4.37E-26
Ckap4	-0.558355594	0.107	0.1	1.22E-10
Ptp4a1	-0.560712643	0.396	0.29	3.25E-50
Rapgef6	-0.560998578	0.122	0.108	1.66E-12
Tm9sf3	-0.561455016	0.158	0.116	1.39E-22
Uqcrh	-0.56146667	0.637	0.503	1.52E-64
Srsf3	-0.562369201	0.333	0.245	8.10E-44
Plekho2	-0.563165504	0.161	0.121	1.44E-25
Itprip	-0.565874383	0.137	0.114	9.62E-17
Prok2	-0.566105122	0.179	0.15	1.32E-13
Hbp1	-0.567415552	0.11	0.101	5.78E-12

Ppp1r15a	-0.56788123	0.476	0.375	3.09E-44
Lrrfip1	-0.568726372	0.247	0.175	5.17E-36
Canx	-0.569062319	0.22	0.167	1.95E-27
Snrpg	-0.569152917	0.128	0.111	6.89E-14
Cmtm6	-0.569708877	0.211	0.167	1.94E-25
Pabpn1	-0.571624989	0.101	0.102	4.34E-10
Ube2b	-0.572630154	0.586	0.422	9.09E-71
Dnajb6	-0.572760467	0.223	0.163	1.58E-33
Rassf3	-0.575303269	0.196	0.152	4.06E-27
Cers6	-0.575881207	0.149	0.11	4.28E-23
Sun2	-0.578970776	0.113	0.098	2.06E-16
Elavl1	-0.578977627	0.146	0.125	2.16E-17
Inpp5d	-0.5793154	0.113	0.095	2.93E-15
Dnajc5	-0.579337798	0.128	0.106	4.46E-17
Rock1	-0.579554525	0.116	0.103	1.97E-13
Osm	-0.584003061	0.423	0.298	1.14E-49
Sf1	-0.584565896	0.185	0.157	1.44E-21
Top1	-0.587575126	0.125	0.107	6.47E-13
Rps19	-0.589055995	0.658	0.43	2.49E-73
Lilr4b	-0.59212724	0.527	0.363	2.11E-72
Kmt2e	-0.592159796	0.176	0.138	2.40E-23
Crk	-0.592259944	0.116	0.094	5.94E-19
Rtn4	-0.593147422	0.182	0.142	8.32E-27
Rrbp1	-0.593338098	0.202	0.147	3.90E-31
Sfpq	-0.593898098	0.176	0.142	1.05E-22
Cxcr2	-0.594136535	0.557	0.391	3.07E-73
Hnrnpa3	-0.595363709	0.28	0.213	7.47E-42
Tnfrsf1b	-0.59638654	0.259	0.208	1.46E-28
Adrbk1	-0.597086892	0.336	0.253	9.23E-44
Serbp1	-0.598044393	0.155	0.137	7.34E-18
Nfe2	-0.599050057	0.185	0.143	1.45E-27
2810474C	-0.599911427	0.345	0.24	1.50E-55
Kras	-0.600470703	0.173	0.14	1.27E-23
Gna13	-0.600758576	0.173	0.142	3.10E-22
Ptpn12	-0.600781826	0.131	0.11	1.10E-18
Gnb1	-0.600843008	0.229	0.171	2.13E-31
Cd164	-0.601813606	0.22	0.169	2.30E-33
Arid1a	-0.603544255	0.122	0.108	7.39E-15
Irf2	-0.608139788	0.116	0.107	1.44E-13
Grina	-0.60814611	0.848	0.647	9.49E-79
Thy1	-0.608907169	0.119	0.104	5.24E-15
Atf7ip	-0.609042615	0.101	0.096	1.43E-12
Cnot6l	-0.611701879	0.107	0.109	2.04E-10
Rad21	-0.612781113	0.146	0.126	5.88E-20
Prex1	-0.613236973	0.131	0.109	6.82E-20
Cd84	-0.613258048	0.161	0.135	5.23E-24
Thbs1	-0.613720121	0.449	0.291	3.80E-42
Crlf3	-0.61516616	0.116	0.107	3.90E-13
Gsr	-0.61711779	0.783	0.61	2.75E-77
Steap4	-0.617947954	0.134	0.116	1.05E-18
Irf1	-0.618427592	0.256	0.211	7.30E-33
Gpbp1	-0.618772256	0.193	0.148	2.96E-31
Mxd1	-0.620377462	0.845	0.653	1.21E-76
Lasp1	-0.62420237	0.152	0.135	3.82E-18
Ptk2b	-0.626556133	0.176	0.139	2.70E-29
Rela	-0.627995286	0.107	0.098	3.35E-15
Wnk1	-0.628446752	0.167	0.13	5.52E-28
Cyth1	-0.6302632	0.134	0.108	5.92E-22

Zfp36	-0.630741377	0.824	0.649	2.95E-67
Tle3	-0.631223352	0.214	0.162	2.12E-29
Atrx	-0.631526774	0.095	0.1	1.04E-09
Hsph1	-0.63225026	0.17	0.134	1.07E-22
H2-K1	-0.632778024	0.798	0.663	4.34E-65
Rnf144a	-0.633219553	0.188	0.143	2.35E-32
Cd53	-0.637535296	0.628	0.462	2.33E-80
Hnrnpdl	-0.637640106	0.25	0.19	1.08E-34
Serinc1	-0.640162511	0.11	0.103	3.79E-17
Tor1aip1	-0.64103333	0.134	0.119	4.30E-18
Hnrnpu	-0.6448138	0.235	0.186	2.32E-33
Kctd12	-0.645947287	0.426	0.291	9.19E-65
Clk4	-0.647784549	0.113	0.104	4.66E-15
Spata13	-0.648621451	0.176	0.143	1.92E-30
Set	-0.648664587	0.119	0.114	3.53E-15
Zc3hav1	-0.648977629	0.179	0.157	2.81E-21
Actn1	-0.649831573	0.161	0.131	6.25E-23
Hp1bp3	-0.650341673	0.107	0.105	3.48E-13
Pafah1b1	-0.650844734	0.188	0.155	1.73E-25
Eif3a	-0.652093405	0.122	0.121	1.59E-15
Ncor1	-0.65212702	0.128	0.119	2.90E-15
Pde4b	-0.653550735	0.378	0.284	7.37E-54
Tcf25	-0.654229608	0.155	0.131	1.02E-22
Zdhhc18	-0.654526951	0.137	0.125	3.21E-15
Whsc1l1	-0.655958853	0.193	0.146	9.53E-36
Hectd1	-0.656397964	0.095	0.101	4.64E-09
Rbm39	-0.656656961	0.58	0.449	5.90E-74
Stat3	-0.659352991	0.241	0.2	5.77E-32
Midn	-0.660739902	0.146	0.125	3.70E-20
Pabpc1	-0.660844384	0.393	0.309	1.79E-51
Lamp2	-0.660964257	0.339	0.24	1.45E-56
Trip12	-0.661691144	0.107	0.107	4.68E-12
Dedd2	-0.661946001	0.107	0.098	1.97E-15
Cd177	-0.662747689	0.161	0.148	7.71E-16
Eif2s2	-0.662906417	0.193	0.166	3.46E-26
Trim30d	-0.664046286	0.107	0.098	6.65E-14
Ywhaz	-0.664372467	0.399	0.294	5.35E-62
Arf3	-0.664715707	0.113	0.112	2.59E-14
Rasgrp2	-0.667090714	0.185	0.159	1.29E-23
Wipf1	-0.667857665	0.211	0.177	8.64E-32
Ythdf3	-0.671344175	0.107	0.104	1.00E-12
Sdcbp	-0.672415685	0.554	0.408	9.92E-78
Hnrnpa2b	-0.674965739	0.19	0.161	1.28E-22
Sidt2	-0.675078618	0.131	0.119	1.85E-17
Mmp9	-0.675584404	0.613	0.449	1.39E-84
Rpl41	-0.675693532	1	0.981	1.72E-23
Rnf125	-0.675733207	0.22	0.169	2.52E-35
Rsrc2	-0.67624239	0.247	0.19	7.19E-40
Rps20	-0.676831006	0.804	0.584	2.99E-97
Phf20l1	-0.677712838	0.113	0.11	1.65E-12
Samd9l	-0.677938766	0.19	0.159	5.06E-25
Rpsa	-0.679012967	0.521	0.378	1.41E-70
Rsrp1	-0.680506332	0.488	0.357	1.57E-68
Bcl2l11	-0.680550566	0.321	0.263	6.29E-35
St8sia4	-0.68231562	0.199	0.163	7.59E-31
Rpl35a	-0.683429576	0.182	0.166	1.87E-18
Sorl1	-0.684713624	0.44	0.33	2.25E-64
Tiparp	-0.685211706	0.158	0.139	6.13E-21

Basp1	-0.685583206	0.119	0.11	8.19E-15
Rps24	-0.688258637	0.539	0.39	6.39E-67
Ptp4a2	-0.68830779	0.214	0.163	2.06E-38
Fbxl3	-0.688737226	0.11	0.114	1.12E-11
Cmah	-0.689072481	0.101	0.111	3.48E-12
Kpna4	-0.690320347	0.128	0.124	6.19E-15
Cd300lf	-0.691039818	0.458	0.349	2.18E-57
Shisa5	-0.693445355	0.509	0.374	9.13E-80
Gm1966	-0.693577934	0.107	0.114	2.35E-10
Pmaip1	-0.69369205	0.125	0.118	8.08E-16
D16Ertd47	-0.693822485	0.122	0.12	4.14E-16
Matr3	-0.694128768	0.098	0.106	2.02E-11
Ugcg	-0.694737253	0.119	0.111	1.78E-15
Oas3	-0.69617145	0.143	0.133	1.80E-17
Ets2	-0.696281113	0.577	0.426	2.93E-79
Hmha1	-0.696905905	0.387	0.29	9.09E-61
Klf10	-0.699315489	0.113	0.11	6.32E-15
Trim12c	-0.700595167	0.176	0.156	1.70E-22
Lmo4	-0.701115221	0.173	0.15	6.22E-27
Mfsd14b	-0.70228877	0.161	0.141	2.94E-21
Rara	-0.702859011	0.146	0.139	2.67E-20
Il17ra	-0.703566594	0.268	0.221	1.91E-40
Rpl22l1	-0.704842538	0.384	0.298	3.35E-51
Hif1a	-0.70640115	0.259	0.208	1.92E-35
Phf21a	-0.70688965	0.116	0.126	1.31E-11
Zcchc6	-0.707443007	0.128	0.131	1.26E-15
Klhl2	-0.711560709	0.122	0.121	2.65E-16
Msl2	-0.712053051	0.11	0.113	1.23E-15
App	-0.712474602	0.333	0.249	9.94E-52
Il18rap	-0.712812065	0.143	0.14	1.16E-16
Hnrnpk	-0.715813888	0.363	0.291	2.26E-55
Slc15a3	-0.715981364	0.259	0.216	9.97E-36
Jun	-0.717197676	0.488	0.365	2.48E-66
Rpl35	-0.718057933	0.804	0.581	5.12E-88
Gimap3	-0.718976989	0.149	0.138	3.42E-24
Gzma	-0.719182537	0.327	0.165	1.72E-48
4932438A	-0.722111751	0.098	0.106	5.42E-15
Ube2h	-0.72526116	0.217	0.191	4.11E-28
Ipcef1	-0.727337823	0.092	0.104	2.76E-12
Hspa8	-0.727967807	0.756	0.591	6.47E-88
Samsn1	-0.72903808	0.461	0.375	1.36E-59
Rps3a1	-0.729522088	0.562	0.433	6.32E-73
Pbx1	-0.730885597	0.134	0.126	1.80E-19
Rpl37a	-0.732383361	0.25	0.226	2.98E-33
Sgms2	-0.732800216	0.149	0.142	1.16E-18
Wdr26	-0.733382814	0.158	0.156	6.18E-20
Rbms1	-0.734856491	0.304	0.237	1.22E-48
Ncf1	-0.736921891	0.414	0.336	7.26E-53
H2-Q4	-0.737851403	0.116	0.12	2.62E-16
AI467606	-0.741378329	0.131	0.129	4.04E-19
Ikzf1	-0.743304836	0.185	0.172	6.74E-23
Gda	-0.743744215	0.673	0.522	1.06E-84
Stk4	-0.744313696	0.095	0.105	8.28E-14
Per1	-0.74959976	0.232	0.2	5.83E-35
Hsp90ab1	-0.74974606	0.438	0.348	3.15E-64
Osgin1	-0.751674213	0.101	0.121	2.45E-10
Azin1	-0.753683074	0.146	0.138	3.23E-23
Rbm25	-0.754773268	0.074	0.101	1.28E-08

Prkcb	-0.755096101	0.188	0.171	1.35E-29
Sh2d2a	-0.757079133	0.08	0.101	7.75E-09
Myo9b	-0.757533857	0.113	0.118	1.07E-14
Birc3	-0.758453892	0.217	0.181	2.80E-31
Tab2	-0.759793428	0.122	0.125	2.66E-16
Mapk14	-0.760482681	0.086	0.103	3.03E-08
Sf3b1	-0.761554708	0.315	0.254	1.67E-46
Rps26	-0.772074192	0.092	0.11	1.40E-14
Stk38	-0.773722011	0.179	0.171	1.47E-23
Arih1	-0.773990617	0.11	0.122	6.43E-14
Celf1	-0.778109935	0.083	0.106	6.77E-11
Eif4g2	-0.778513788	0.321	0.287	2.43E-44
Chd7	-0.778942836	0.122	0.126	3.92E-20
Wdr89	-0.780590931	0.982	0.863	3.93E-56
Themis2	-0.784930559	0.182	0.158	8.34E-33
Cpeb2	-0.785433626	0.146	0.131	3.50E-27
Safb	-0.785679188	0.089	0.107	5.26E-11
Macf1	-0.786958783	0.104	0.11	4.06E-19
Odc1	-0.787429644	0.107	0.11	2.04E-16
Nampt	-0.788049437	0.107	0.11	1.36E-17
Lyst	-0.789318458	0.161	0.16	2.28E-23
Csf3r	-0.789335494	0.824	0.678	4.30E-77
Fnbp1	-0.790520664	0.083	0.105	2.34E-13
Ppp1r12a	-0.791812566	0.131	0.129	1.53E-21
Suco	-0.792023942	0.089	0.114	1.04E-10
Tmcc1	-0.792652549	0.188	0.174	1.45E-28
Ssh2	-0.795016331	0.307	0.245	6.08E-50
Iqgap1	-0.7958229	0.455	0.38	4.43E-66
Ncl	-0.796405466	0.167	0.161	2.38E-23
Cytip	-0.796862246	0.565	0.437	2.45E-83
Ezr	-0.797262709	0.226	0.201	1.23E-34
Hdac4	-0.800193596	0.149	0.136	2.38E-27
Rpl22	-0.800757418	0.476	0.379	4.99E-64
Polr2a	-0.804782243	0.17	0.151	4.21E-29
Rgs2	-0.806728975	0.423	0.348	1.39E-55
Egr1	-0.808640888	0.092	0.1	1.89E-10
Heca	-0.810795879	0.122	0.136	9.71E-16
Son	-0.811210644	0.286	0.255	2.10E-41
Ankrd44	-0.812620964	0.101	0.119	2.37E-14
Dck	-0.814590418	0.223	0.176	7.32E-44
Neurl3	-0.820276916	0.158	0.157	9.64E-22
Serinc3	-0.820277698	0.44	0.337	8.72E-73
Pcbp1	-0.822051081	0.205	0.185	3.54E-34
Rdm1	-0.825710463	0.164	0.164	1.94E-18
Klf7	-0.827559727	0.101	0.135	1.39E-08
Myh9	-0.828639425	0.208	0.195	3.35E-30
Klhl24	-0.828789497	0.122	0.136	8.95E-19
Pik3cd	-0.829824701	0.137	0.139	3.88E-23
Fam134b	-0.832118606	0.185	0.172	1.97E-28
Cab39	-0.834440241	0.092	0.118	4.94E-13
Hipk1	-0.838351838	0.223	0.203	4.41E-35
Sp110	-0.839018036	0.098	0.115	1.06E-18
Tkt	-0.839073168	0.336	0.282	3.38E-56
Susd6	-0.840890654	0.101	0.133	3.75E-09
Ewsr1	-0.840986307	0.14	0.143	1.01E-23
Ddx3x	-0.847931048	0.274	0.258	1.69E-35
Emilin2	-0.849245883	0.33	0.273	5.60E-58
Tnks2	-0.850472314	0.068	0.107	1.27E-08

Ier5	-0.852001378	0.652	0.517	1.24E-87
Rps25	-0.853185055	0.429	0.349	3.84E-70
Pag1	-0.854645145	0.089	0.117	6.83E-13
Tgoln1	-0.855936956	0.211	0.199	2.42E-33
Atp1b3	-0.860091516	0.315	0.265	2.00E-51
Nufip2	-0.860534336	0.101	0.115	1.22E-19
Nr3c1	-0.861637883	0.167	0.157	1.28E-27
Clec2d	-0.863690883	0.122	0.142	3.12E-15
Tob1	-0.864434561	0.152	0.158	2.29E-23
Ddx17	-0.866129238	0.149	0.164	2.41E-22
Nfkbiz	-0.86699004	0.667	0.564	8.44E-61
Trib1	-0.867071404	0.336	0.29	1.91E-49
Ppp1r3b	-0.867833033	0.152	0.161	4.64E-23
Ahnak	-0.868569019	0.188	0.184	1.02E-27
Il1rap	-0.871597705	0.22	0.209	5.60E-34
Sh2d3c	-0.8721117	0.137	0.147	7.50E-25
Ifrd1	-0.873323767	0.31	0.269	2.50E-44
Txnip	-0.874744525	0.348	0.288	1.84E-50
Pum2	-0.876965318	0.149	0.157	7.88E-24
Nr4a2	-0.87768528	0.086	0.103	1.44E-12
Csrnp1	-0.881723591	0.411	0.36	2.26E-56
Ccr7	-0.882792055	0.128	0.137	5.47E-21
Pnisr	-0.88335993	0.11	0.133	1.47E-14
Rab8b	-0.884097715	0.304	0.275	1.44E-46
Ccdc88c	-0.887432121	0.083	0.114	1.93E-14
Hnrnpm	-0.887570923	0.196	0.184	2.08E-33
Fos	-0.889481651	0.875	0.821	7.02E-53
Fbxl5	-0.890393517	0.423	0.374	3.86E-55
Junb	-0.893616173	0.979	0.95	2.49E-53
Ddit3	-0.893937811	0.31	0.269	5.37E-48
Ep300	-0.895710786	0.074	0.114	5.42E-10
Abi1	-0.900272692	0.14	0.155	4.07E-24
Il4ra	-0.906242923	0.214	0.212	3.46E-33
Tnrc6b	-0.907217719	0.113	0.138	2.87E-17
Map3k1	-0.909197682	0.08	0.121	1.09E-10
Snrnp70	-0.910698724	0.202	0.197	1.13E-36
Mgea5	-0.913433207	0.128	0.152	4.34E-17
Atf4	-0.918483241	0.396	0.363	3.42E-60
Igf1r	-0.921102498	0.158	0.169	1.22E-26
Clint1	-0.921787151	0.104	0.131	3.87E-20
Ddx3y	-0.922830701	0.199	0.183	9.34E-40
Crebrf	-0.925008974	0.119	0.152	2.20E-17
Ist1	-0.930296035	0.193	0.202	1.85E-28
Eef2	-0.934506115	0.58	0.5	4.09E-86
Gpcpd1	-0.937112907	0.452	0.357	2.20E-84
Lbr	-0.943814016	0.399	0.362	1.78E-54
Srsf6	-0.944593534	0.143	0.158	5.14E-26
Rnf149	-0.945154251	0.649	0.551	9.38E-79
Slc6a6	-0.949839183	0.19	0.193	5.53E-35
Rpl23a	-0.952397595	0.619	0.489	8.97E-83
Hsp90aa1	-0.955379609	0.31	0.278	5.16E-44
Csnk1a1	-0.957464548	0.116	0.153	2.18E-18
Prpf38b	-0.959817225	0.119	0.144	2.50E-19
Nr4a3	-0.96202301	0.089	0.118	2.39E-18
Ythdc1	-0.96366106	0.161	0.171	5.10E-28
Arid5a	-0.964447974	0.182	0.192	4.48E-30
Ip6k1	-0.968586001	0.113	0.139	1.82E-23
Vps4b	-0.96986259	0.104	0.143	9.03E-18

Ogt	-0.971118469	0.104	0.138	4.61E-16
Jak1	-0.971430263	0.262	0.257	5.67E-45
Foxp1	-0.988770082	0.134	0.167	1.29E-18
Gls	-0.989329224	0.122	0.153	1.86E-22
Arhgef3	-0.990507248	0.065	0.112	5.61E-12
Sp100	-0.990729605	0.205	0.213	5.91E-34
Bhlhe40	-0.991344604	0.092	0.139	1.96E-11
Nabp1	-0.999660785	0.134	0.155	2.86E-22
Purb	-0.999730704	0.062	0.109	3.48E-14
Snx18	-0.999904149	0.378	0.339	1.43E-66
Trim30a	-1.003169874	0.307	0.321	1.82E-38
Esyt2	-1.006240079	0.101	0.135	2.04E-19
Clk1	-1.008842625	0.384	0.38	1.54E-57
Elf1	-1.008883831	0.098	0.149	2.82E-17
Fam65b	-1.010363135	0.226	0.236	1.07E-40
Rassf5	-1.015853561	0.199	0.211	2.77E-40
Taok1	-1.022660725	0.048	0.101	1.65E-11
Rplp1	-1.027537592	0.917	0.785	5.30E-79
Ptgs2	-1.029907093	0.396	0.322	5.35E-48
Kdm7a	-1.039311398	0.307	0.312	3.10E-42
Rpl39	-1.039763494	0.577	0.467	1.56E-101
Mcl1	-1.039872842	0.866	0.794	2.04E-81
Cmip	-1.04668554	0.214	0.242	4.26E-35
Fus	-1.048281763	0.173	0.208	1.09E-28
Gramd3	-1.049788276	0.101	0.141	1.63E-20
Wsb1	-1.053062568	0.182	0.21	5.30E-33
Rsad2	-1.055529913	0.071	0.103	1.80E-09
Dazap2	-1.058972791	0.476	0.422	6.95E-82
Srrm2	-1.05958473	0.185	0.217	1.30E-32
Gcnt2	-1.061840282	0.339	0.349	1.14E-51
Mt-mt-Rnr	-1.062770564	0.92	0.872	7.45E-74
Gpr132	-1.063622148	0.223	0.23	1.30E-37
Hexim1	-1.066096917	0.131	0.179	8.96E-19
Slfn4	-1.066824102	0.31	0.3	9.21E-48
Hnrnpa0	-1.068682643	0.134	0.179	1.59E-25
Prpf4b	-1.075422467	0.054	0.118	6.26E-13
St3gal4	-1.083898648	0.318	0.276	2.36E-48
Ankrd11	-1.087127927	0.089	0.142	2.21E-15
Dusp1	-1.087134737	0.869	0.811	2.55E-67
Celf2	-1.089103443	0.307	0.31	6.72E-51
Il2rb	-1.090790773	0.164	0.185	2.44E-32
Hnrnph1	-1.095645529	0.199	0.224	3.61E-39
Mbnl1	-1.096731893	0.205	0.222	1.31E-41
Itgal	-1.098109385	0.42	0.411	2.06E-64
Tra2a	-1.09956264	0.125	0.168	4.95E-22
N4bp1	-1.100058813	0.161	0.198	3.83E-27
Zcchc11	-1.100349473	0.057	0.113	7.21E-15
Luc7l2	-1.114827742	0.241	0.268	3.56E-43
Pcbp2	-1.116986614	0.321	0.336	2.81E-63
Atp2b1	-1.12539667	0.14	0.184	7.55E-26
Nr4a1	-1.14387267	0.387	0.394	2.33E-58
Tpt1	-1.14750081	0.804	0.71	1.83E-99
Tnfaip2	-1.156003267	0.426	0.431	1.23E-52
Rps8	-1.168528189	0.283	0.321	1.15E-43
Irf2bp2	-1.16888918	0.182	0.227	9.91E-36
Zfp36l2	-1.171810455	0.423	0.402	1.49E-70
Peli1	-1.173320413	0.152	0.205	4.55E-26
Prrc2c	-1.184572701	0.042	0.122	4.94E-14

Klf6	-1.187435789	0.527	0.511	9.83E-86
Ppp3ca	-1.188098094	0.125	0.186	1.85E-25
Ptprc	-1.207723302	0.625	0.645	9.95E-85
Tnfaip3	-1.208540549	0.494	0.514	2.19E-51
Fosl2	-1.208596856	0.545	0.543	3.72E-76
Btg1	-1.22600213	0.982	0.96	4.58E-100
Tcp11l2	-1.227690882	0.167	0.23	3.65E-35
Cd44	-1.247430383	0.673	0.68	1.80E-95
Slc38a2	-1.263804275	0.226	0.271	2.17E-42
Stk17b	-1.335588453	0.744	0.742	3.64E-108
Zfp36l1	-1.336613314	0.348	0.374	1.02E-60
Ddx6	-1.337060524	0.363	0.441	5.32E-59
Marcks	-1.341250751	0.479	0.51	2.97E-70
Ptbp3	-1.342994262	0.244	0.326	4.66E-51
Akna	-1.359105479	0.119	0.208	2.59E-26
Vps37b	-1.37901621	0.411	0.434	6.53E-77
P2ry10	-1.415937977	0.08	0.176	1.41E-24
Ddx5	-1.441335497	0.81	0.842	2.38E-128
Ccnl1	-1.459008981	0.312	0.377	2.09E-67
Hspa1b	-1.475060888	0.167	0.208	7.81E-26
Tra2b	-1.483802836	0.188	0.28	2.13E-46
Mt-mt-Rnr	-1.485154042	0.092	0.189	4.39E-26
Cxcr4	-1.489393092	0.423	0.502	9.18E-65
Ets1	-1.534385722	0.295	0.379	1.15E-57
Satb1	-1.589579722	0.193	0.278	5.09E-46
Dennd4a	-1.609769115	0.304	0.421	2.24E-64
Hbb-bs	-2.260597333	0	0.14	3.97E-18

Supplementary Table 9

gene	p_val	avg_log2FC	pct.1	pct.2	p_val_adj	cluster
Ccr7	1.42E-58	1.2862	0.43	0.29	2.84E-55	0-CD8+T naive
Igfbp4	5.82E-19	1.2281	0.171	0.132	1.16E-15	0-CD8+T naive
Lef1	6.94E-32	1.1823	0.265	0.189	1.39E-28	0-CD8+T naive
S1pr1	7.98E-12	1.0155	0.259	0.237	1.60E-08	0-CD8+T naive
Fam101b	0.0038315	0.9817	0.128	0.148	1	0-CD8+T naive
Dusp10	0.0016349	0.8694	0.244	0.256	1	0-CD8+T naive
Rgcc	2.17E-09	0.5093	0.103	0.119	4.34E-06	0-CD8+T naive
Rps2	5.64E-05	0.4869	0.407	0.43	0.1128185	0-CD8+T naive
Cd55	3.19E-05	0.4523	0.075	0.156	0.0637214	0-CD8+T naive
Cd8b1	4.98E-08	0.4402	0.187	0.197	9.96E-05	0-CD8+T naive
Lcn4	0.0003232	0.2920	0.049	0.132	0.6464005	0-CD8+T naive
Cks2	0.0020875	0.2883	0.091	0.135	1	0-CD8+T naive
Dnajc9	0.0001085	0.2714	0.069	0.11	0.2169797	0-CD8+T naive
F420015M19Ri	0.0057543	0.2681	0.076	0.157	1	0-CD8+T naive
Pmepa1	8.37E-07	0.2629	0.072	0.164	0.0016741	0-CD8+T naive
**gene**	**p_val**	**avg_log2FC**	**pct.1**	**pct.2**	**p_val_adj**	**cluster**
Gzma	0	3.1755	0.86	0.206	0	1-NK
Irf8	1.50E-269	2.5638	0.721	0.21	3.00E-266	1-NK
Klra4	2.87E-128	2.4469	0.322	0.06	5.75E-125	1-NK
Klra8	7.46E-135	2.3823	0.329	0.057	1.49E-131	1-NK
Spry2	1.65E-128	2.0522	0.463	0.151	3.30E-125	1-NK
Serpinb6b	2.37E-133	1.9642	0.536	0.194	4.74E-130	1-NK
Klri2	2.52E-64	1.8884	0.276	0.076	5.04E-61	1-NK
Klre1	1.11E-130	1.8325	0.456	0.123	2.21E-127	1-NK
Vegfa	1.80E-57	1.8248	0.292	0.118	3.59E-54	1-NK
Serpinb9	1.71E-113	1.7564	0.514	0.194	3.41E-110	1-NK
Ccl3	4.51E-65	1.7358	0.337	0.115	9.02E-62	1-NK
Klrb1c	6.82E-80	1.7091	0.292	0.075	1.36E-76	1-NK
Gzmb	6.84E-77	1.5980	0.4	0.141	1.37E-73	1-NK
Ccl4	1.07E-74	1.5290	0.633	0.381	2.13E-71	1-NK
Eomes	1.12E-57	1.4996	0.282	0.107	2.24E-54	1-NK
Gem	7.03E-43	1.4732	0.331	0.155	1.41E-39	1-NK
Ccl5	2.24E-218	1.4520	0.971	0.688	4.48E-215	1-NK
Klra9	3.45E-37	1.3840	0.192	0.049	6.89E-34	1-NK
Nkg7	2.39E-97	1.3533	0.666	0.363	4.77E-94	1-NK
Klrb1b	1.44E-51	1.3178	0.218	0.063	2.89E-48	1-NK
Bhlhe40	1.18E-68	1.2989	0.501	0.249	2.36E-65	1-NK
Nr4a2	2.33E-59	1.2203	0.458	0.232	4.66E-56	1-NK
Cma1	5.74E-21	1.2025	0.14	0.023	1.15E-17	1-NK
Itgam	1.20E-29	1.1650	0.189	0.064	2.41E-26	1-NK
Klra7	9.85E-19	1.1260	0.151	0.041	1.97E-15	1-NK
St3gal4	3.34E-33	1.1168	0.348	0.187	6.67E-30	1-NK
Ncr1	6.46E-28	1.1104	0.153	0.032	1.29E-24	1-NK
Ccr5	1.92E-17	1.0990	0.25	0.131	3.83E-14	1-NK
Anxa2	2.26E-34	1.0640	0.326	0.158	4.52E-31	1-NK
Khdc1a	1.49E-34	1.0238	0.152	0.031	2.99E-31	1-NK
Klrb1f	4.36E-16	0.9849	0.162	0.069	8.72E-13	1-NK
Mmd	1.60E-24	0.9845	0.257	0.142	3.20E-21	1-NK
Dhrs3	1.50E-09	0.9817	0.127	0.035	3.00E-06	1-NK
Car2	1.94E-25	0.9751	0.159	0.051	3.89E-22	1-NK
Klrc1	4.55E-34	0.9735	0.366	0.2	9.09E-31	1-NK
Atp1b1	1.14E-45	0.9639	0.241	0.088	2.27E-42	1-NK
Camk2n1	1.79E-05	0.9628	0.146	0.08	0.0358695	1-NK
Klrk1	4.45E-30	0.9475	0.265	0.125	8.89E-27	1-NK
Gm6637	3.24E-14	0.9377	0.144	0.058	6.49E-11	1-NK

Sgk1	1.32E-10	0.9332	0.233	0.148	2.65E-07	1-NK
Metrnl	3.57E-14	0.9216	0.174	0.079	7.14E-11	1-NK
Id2	5.76E-38	0.9181	0.454	0.257	1.15E-34	1-NK
Zeb2	2.23E-35	0.9103	0.233	0.105	4.46E-32	1-NK
Klrg1	1.44E-15	0.8618	0.146	0.049	2.89E-12	1-NK
Rgs1	1.50E-38	0.8243	0.494	0.295	2.99E-35	1-NK
Nr4a3	2.55E-09	0.8131	0.329	0.239	5.10E-06	1-NK
Nfkbiz	4.07E-28	0.8038	0.432	0.274	8.13E-25	1-NK
Klf10	6.37E-25	0.7736	0.265	0.149	1.27E-21	1-NK
Itgax	1.35E-14	0.7636	0.165	0.085	2.70E-11	1-NK
Ifng	0.001519	0.7477	0.198	0.156	1	1-NK
Bcl2l11	2.60E-23	0.7464	0.489	0.346	5.20E-20	1-NK
Ptger4	4.65E-09	0.7463	0.233	0.163	9.29E-06	1-NK
Cd9	1.10E-14	0.7279	0.169	0.083	2.19E-11	1-NK
Zbtb20	4.97E-16	0.7021	0.308	0.204	9.95E-13	1-NK
Gimap5	4.98E-23	0.6837	0.275	0.153	9.96E-20	1-NK
Klrd1	3.23E-13	0.6749	0.281	0.178	6.46E-10	1-NK
Icam1	3.12E-13	0.6487	0.257	0.175	6.25E-10	1-NK
H2afz	4.36E-20	0.6481	0.594	0.473	8.71E-17	1-NK
Kit	7.05E-20	0.6415	0.156	0.064	1.41E-16	1-NK
Xcl1	3.68E-08	0.6406	0.207	0.131	7.35E-05	1-NK
Fyn	1.00E-09	0.6175	0.33	0.244	2.01E-06	1-NK
Ccr2	1.33E-12	0.6122	0.196	0.11	2.66E-09	1-NK
Prdm1	4.99E-21	0.6015	0.176	0.09	9.98E-18	1-NK
Olfm1	2.15E-23	0.5994	0.123	0.024	4.29E-20	1-NK
Sema4c	1.87E-15	0.5881	0.123	0.039	3.74E-12	1-NK
Ppp1r3b	8.76E-22	0.5859	0.175	0.089	1.75E-18	1-NK
Gpr171	1.72E-08	0.5724	0.133	0.071	3.44E-05	1-NK
Ccnd2	7.97E-12	0.5631	0.416	0.317	1.59E-08	1-NK
Spn	0.000473	0.5480	0.25	0.199	0.9459077	1-NK
Nfkbia	7.21E-15	0.5268	0.638	0.539	1.44E-11	1-NK
Ifrd1	1.44E-16	0.5242	0.49	0.361	2.87E-13	1-NK
Il18r1	1.16E-16	0.5216	0.274	0.178	2.33E-13	1-NK
Gadd45b	8.78E-07	0.5079	0.267	0.198	0.0017556	1-NK
**gene**	**p_val**	**avg_log2FC**	**pct.1**	**pct.2**	**p_val_adj**	**cluster**
Hspa1b	2.51E-143	1.8559	0.722	0.371	5.02E-140	2-CD8+T-Hspa1b
Hspa1a	1.11E-125	1.7926	0.658	0.321	2.21E-122	2-CD8+T-Hspa1b
Dnajb1	7.05E-74	1.3461	0.529	0.273	1.41E-70	2-CD8+T-Hspa1b
Hsp90aa1	1.93E-65	1.0550	0.687	0.46	3.86E-62	2-CD8+T-Hspa1b
Jun	1.13E-58	1.0541	0.609	0.376	2.25E-55	2-CD8+T-Hspa1b
Hsph1	1.79E-26	1.0476	0.379	0.25	3.58E-23	2-CD8+T-Hspa1b
Phlda1	4.13E-06	0.8791	0.151	0.129	0.0082562	2-CD8+T-Hspa1b
Hspe1	1.78E-29	0.8358	0.508	0.356	3.56E-26	2-CD8+T-Hspa1b
Fam46a	2.79E-07	0.7770	0.153	0.127	0.0005579	2-CD8+T-Hspa1b
Tagap	0.0032391	0.7699	0.235	0.219	1	2-CD8+T-Hspa1b
Hspd1	1.06E-15	0.7186	0.253	0.178	2.13E-12	2-CD8+T-Hspa1b
Cd69	2.03E-08	0.6364	0.227	0.185	4.06E-05	2-CD8+T-Hspa1b
Amd1	0.0001992	0.6122	0.3	0.269	0.3983249	2-CD8+T-Hspa1b
Dnaja4	7.51E-06	0.5555	0.115	0.105	0.0150177	2-CD8+T-Hspa1b
**gene**	**p_val**	**avg_log2FC**	**pct.1**	**pct.2**	**p_val_adj**	**cluster**
Ccl5	1.08E-88	0.9567	0.898	0.702	2.16E-85	3-CD8+ccl5
Klrc1	4.02E-17	0.9221	0.312	0.21	8.05E-14	3-CD8+ccl5
Rora	2.20E-17	0.9050	0.323	0.226	4.40E-14	3-CD8+ccl5
Bcl2a1b	9.43E-13	0.8141	0.211	0.144	1.89E-09	3-CD8+ccl5
Nkg7	2.41E-28	0.6561	0.54	0.386	4.81E-25	3-CD8+ccl5
Hopx	2.70E-09	0.6558	0.174	0.125	5.40E-06	3-CD8+ccl5
Cd8a	5.69E-07	0.6438	0.173	0.129	0.0011385	3-CD8+ccl5
Rpa2	6.12E-11	0.6410	0.118	0.072	1.22E-07	3-CD8+ccl5

Icos	4.93E-05	0.6086	0.246	0.211	0.0986361	3-CD8+ccl5
Il18r1	5.19E-09	0.5985	0.239	0.185	1.04E-05	3-CD8+ccl5
Bcl2a1d	9.89E-08	0.5644	0.131	0.092	0.0001978	3-CD8+ccl5
Serpinb9	2.82E-07	0.5395	0.287	0.234	0.000565	3-CD8+ccl5
Klrk1	0.0011757	0.5241	0.165	0.143	1	3-CD8+ccl5
Lgals1	2.91E-09	0.5218	0.328	0.268	5.82E-06	3-CD8+ccl5
Krtcap2	3.43E-05	0.5127	0.17	0.141	0.0686123	3-CD8+ccl5
Ms4a4b	4.84E-05	0.5001	0.33	0.296	0.0967779	3-CD8+ccl5
Cx3cr1	3.51E-10	0.4897	0.1	0.064	7.01E-07	3-CD8+ccl5
Ly6c2	0.0001592	0.4739	0.297	0.262	0.3184265	3-CD8+ccl5
Cd28	0.0013318	0.4495	0.284	0.259	1	3-CD8+ccl5
Klrg1	4.46E-11	0.4315	0.108	0.056	8.92E-08	3-CD8+ccl5
S100a6	4.88E-16	0.4297	0.596	0.488	9.76E-13	3-CD8+ccl5
Cdk6	4.07E-05	0.4155	0.117	0.098	0.081438	3-CD8+ccl5
Ctla2a	0.0007345	0.4139	0.209	0.182	1	3-CD8+ccl5
Bhlhe40	0.0001327	0.4133	0.315	0.282	0.2654174	3-CD8+ccl5
Itgb1	0.0002457	0.3760	0.178	0.157	0.4914347	3-CD8+ccl5
Atp8b4	5.15E-06	0.3737	0.116	0.093	0.0103036	3-CD8+ccl5
Cd48	9.97E-05	0.3638	0.127	0.109	0.1994095	3-CD8+ccl5
Trp53inp1	2.38E-06	0.3569	0.174	0.145	0.0047635	3-CD8+ccl5
S100a10	0.006232	0.3451	0.348	0.328	1	3-CD8+ccl5
Cd3d	0.008463	0.3280	0.273	0.257	1	3-CD8+ccl5
Gpr183	0.0077305	0.3155	0.133	0.122	1	3-CD8+ccl5
**gene**	**p_val**	**avg_log2FC**	**pct.1**	**pct.2**	**p_val_adj**	**cluster**
Ifit1	3.14E-93	0.9904	0.382	0.071	6.27E-90	4-CD8+T-Slfn5
Cd8b1	4.15E-47	0.8673	0.524	0.164	8.30E-44	4-CD8+T-Slfn5
Ppa1	9.91E-11	0.7841	0.162	0.044	1.98E-07	4-CD8+T-Slfn5
Slfn5	3.82E-18	0.7795	0.289	0.059	7.64E-15	4-CD8+T-Slfn5
Bst2	1.86E-26	0.6243	0.434	0.14	3.72E-23	4-CD8+T-Slfn5
Usp18	2.81E-06	0.6056	0.243	0.052	0.005612	4-CD8+T-Slfn5
Ly6c2	1.14E-38	0.5755	0.582	0.238	2.27E-35	4-CD8+T-Slfn5
Nme2	5.15E-51	0.5531	0.62	0.236	1.03E-47	4-CD8+T-Slfn5
Rsad2	2.78E-17	0.5199	0.121	0.051	5.56E-14	4-CD8+T-Slfn5
Mif	5.01E-22	0.5156	0.459	0.171	1.00E-18	4-CD8+T-Slfn5
Cd8a	5.15E-11	0.5045	0.345	0.116	1.03E-07	4-CD8+T-Slfn5
Itm2a	9.59E-46	0.4899	0.333	0.065	1.92E-42	4-CD8+T-Slfn5
Tdrd3	1.02E-44	0.4868	0.326	0.079	2.04E-41	4-CD8+T-Slfn5
C1qbp	0.0061273	0.4709	0.225	0.051	1	4-CD8+T-Slfn5
Nme1	3.62E-27	0.4690	0.476	0.179	7.24E-24	4-CD8+T-Slfn5
Ms4a4b	1.01E-29	0.4640	0.603	0.273	2.02E-26	4-CD8+T-Slfn5
Gbp2	3.11E-21	0.4565	0.293	0.063	6.22E-18	4-CD8+T-Slfn5
Cxcl10	6.00E-14	0.4443	0.123	0.044	1.20E-10	4-CD8+T-Slfn5
Gbp5	8.92E-32	0.4245	0.301	0.083	1.78E-28	4-CD8+T-Slfn5
LOC102639543	2.92E-14	0.4210	0.37	0.137	5.84E-11	4-CD8+T-Slfn5
Phf11b	0.0034044	0.4068	0.158	0.035	1	4-CD8+T-Slfn5
Srm	0.002526	0.3994	0.158	0.048	1	4-CD8+T-Slfn5
**gene**	**p_val**	**avg_log2FC**	**pct.1**	**pct.2**	**p_val_adj**	**cluster**
Xcl1	9.89E-110	2.7218	0.539	0.117	1.98E-106	5-NKT-Cd160
Rgs1	2.64E-87	2.2453	0.72	0.299	5.28E-84	5-NKT-Cd160
Cd7	1.32E-65	2.1317	0.453	0.134	2.64E-62	5-NKT-Cd160
Fgl2	2.87E-42	1.8155	0.317	0.098	5.74E-39	5-NKT-Cd160
Cd160	2.31E-34	1.6988	0.248	0.069	4.63E-31	5-NKT-Cd160
Cxcr6	9.95E-26	1.5578	0.275	0.111	1.99E-22	5-NKT-Cd160
Gzmb	3.13E-30	1.5077	0.381	0.166	6.26E-27	5-NKT-Cd160
Hspa1b	4.36E-16	1.3368	0.547	0.414	8.71E-13	5-NKT-Cd160
LOC105247125	5.22E-19	1.3102	0.16	0.046	1.04E-15	5-NKT-Cd160
Ccl4	4.91E-15	1.2913	0.555	0.41	9.82E-12	5-NKT-Cd160
Cd226	1.74E-14	1.2610	0.253	0.134	3.47E-11	5-NKT-Cd160

Nr4a2	3.26E-14	1.2055	0.397	0.257	6.53E-11	5-NKT-Cd160
Spry2	1.98E-14	1.1888	0.336	0.189	3.95E-11	5-NKT-Cd160
Klre1	2.74E-26	1.1848	0.365	0.16	5.49E-23	5-NKT-Cd160
Ccr5	3.10E-20	1.1343	0.293	0.139	6.19E-17	5-NKT-Cd160
Ifng	3.08E-08	1.1327	0.245	0.157	6.15E-05	5-NKT-Cd160
Ier5l	2.82E-07	1.1151	0.195	0.123	0.0005635	5-NKT-Cd160
Rgs2	8.17E-20	1.0670	0.56	0.378	1.63E-16	5-NKT-Cd160
Klrc1	1.69E-21	1.0457	0.4	0.213	3.39E-18	5-NKT-Cd160
Tox	3.06E-13	1.0457	0.216	0.115	6.12E-10	5-NKT-Cd160
Klrd1	4.28E-12	1.0278	0.312	0.186	8.56E-09	5-NKT-Cd160
Id2	1.68E-15	1.0130	0.437	0.276	3.36E-12	5-NKT-Cd160
Serpina3g	1.06E-10	1.0105	0.128	0.041	2.13E-07	5-NKT-Cd160
Ccl5	2.89E-49	0.9520	0.955	0.715	5.77E-46	5-NKT-Cd160
Nkg7	3.43E-24	0.9329	0.616	0.395	6.86E-21	5-NKT-Cd160
Ikzf2	2.36E-06	0.9090	0.213	0.143	0.0047251	5-NKT-Cd160
Klrb1b	3.40E-15	0.8430	0.179	0.08	6.81E-12	5-NKT-Cd160
Ahr	2.82E-11	0.8331	0.117	0.047	5.63E-08	5-NKT-Cd160
Klrb1c	3.38E-13	0.8323	0.205	0.101	6.76E-10	5-NKT-Cd160
Nedd4	7.87E-10	0.8094	0.125	0.057	1.57E-06	5-NKT-Cd160
Klrk1	1.41E-08	0.8079	0.235	0.14	2.82E-05	5-NKT-Cd160
Klrb1f	9.71E-05	0.8070	0.133	0.079	0.1941328	5-NKT-Cd160
Ptger4	0.0032949	0.7821	0.216	0.171	1	5-NKT-Cd160
Lag3	0.0004346	0.7695	0.147	0.104	0.8691519	5-NKT-Cd160
Bhlhe40	1.55E-13	0.7664	0.429	0.278	3.10E-10	5-NKT-Cd160
Dusp1	5.86E-12	0.7499	0.68	0.568	1.17E-08	5-NKT-Cd160
Dnajb1	7.85E-07	0.7492	0.389	0.305	0.0015705	5-NKT-Cd160
Serpinb6b	2.55E-10	0.7475	0.365	0.238	5.10E-07	5-NKT-Cd160
Hsp90aa1	6.35E-07	0.7321	0.565	0.488	0.0012709	5-NKT-Cd160
Bcl2a1d	5.39E-10	0.7263	0.179	0.093	1.08E-06	5-NKT-Cd160
Bcl2a1b	1.52E-08	0.7218	0.245	0.147	3.04E-05	5-NKT-Cd160
Glcci1	0.0001198	0.7182	0.165	0.117	0.2396985	5-NKT-Cd160
Hspa1a	0.0027915	0.7148	0.4	0.368	1	5-NKT-Cd160
Maf	0.00444	0.6903	0.213	0.175	1	5-NKT-Cd160
Itgax	0.0015224	0.6783	0.133	0.094	1	5-NKT-Cd160
Isg15	0.000228	0.6662	0.171	0.121	0.4559224	5-NKT-Cd160
Ctla2a	1.45E-06	0.6645	0.264	0.18	0.0029072	5-NKT-Cd160
Gata3	0.0027549	0.6581	0.16	0.124	1	5-NKT-Cd160
Car2	9.37E-08	0.6450	0.12	0.063	0.0001875	5-NKT-Cd160
Ccnd2	5.00E-09	0.6387	0.443	0.325	1.00E-05	5-NKT-Cd160
S100a6	1.78E-11	0.6302	0.616	0.496	3.56E-08	5-NKT-Cd160
Uqcrb	9.57E-08	0.6265	0.248	0.165	0.0001915	5-NKT-Cd160
S100a4	3.80E-08	0.6212	0.336	0.232	7.59E-05	5-NKT-Cd160
Gcnt2	0.0056765	0.6199	0.208	0.162	1	5-NKT-Cd160
Hspd1	1.07E-07	0.6091	0.269	0.183	0.0002149	5-NKT-Cd160
Ctsc	0.0009115	0.5955	0.157	0.111	1	5-NKT-Cd160
Tex2	6.96E-10	0.5600	0.125	0.063	1.39E-06	5-NKT-Cd160
Arl6ip1	2.00E-05	0.5450	0.349	0.272	0.0400397	5-NKT-Cd160
Pglyrp1	0.001446	0.5419	0.168	0.125	1	5-NKT-Cd160
Malt1	2.07E-07	0.5390	0.235	0.158	0.000415	5-NKT-Cd160
Zfp36l1	3.14E-06	0.5344	0.493	0.404	0.0062775	5-NKT-Cd160
Rnf149	0.0006287	0.5324	0.253	0.195	1	5-NKT-Cd160
Hilpda	0.0095143	0.5265	0.133	0.109	1	5-NKT-Cd160
Nr4a3	0.0031583	0.4947	0.301	0.249	1	5-NKT-Cd160
Klra9	6.35E-05	0.4863	0.112	0.068	0.1269296	5-NKT-Cd160
S100a11	0.0002251	0.4841	0.4	0.333	0.4501357	5-NKT-Cd160
Hspe1	0.0046971	0.4834	0.416	0.375	1	5-NKT-Cd160
Tnpo3	1.20E-05	0.4639	0.149	0.103	0.02393	5-NKT-Cd160
Ccl3	0.0019881	0.4630	0.187	0.146	1	5-NKT-Cd160

Serpinb9	1.91E-09	0.4620	0.347	0.235	3.82E-06	5-NKT-Cd160
Trp53inp1	5.10E-06	0.4588	0.2	0.145	0.0101984	5-NKT-Cd160
Mxd4	1.06E-10	0.4518	0.107	0.048	2.12E-07	5-NKT-Cd160
Gimap5	7.65E-07	0.4501	0.245	0.167	0.0015295	5-NKT-Cd160
Bcl2l11	0.002799	0.4496	0.405	0.365	1	5-NKT-Cd160
Lilrb4a	5.02E-06	0.4486	0.157	0.104	0.0100443	5-NKT-Cd160
Calm3	0.0080082	0.4455	0.147	0.118	1	5-NKT-Cd160
Krtcap2	0.000183	0.4392	0.187	0.142	0.3659775	5-NKT-Cd160
Gadd45g	1.90E-05	0.4388	0.104	0.063	0.0380767	5-NKT-Cd160
Enpp4	1.48E-05	0.4331	0.123	0.085	0.0296097	5-NKT-Cd160
Cdkn1a	0.0015863	0.4250	0.147	0.115	1	5-NKT-Cd160
Hmgb2	1.03E-05	0.4204	0.549	0.465	0.0205704	5-NKT-Cd160
Etfb	0.0007716	0.4134	0.123	0.09	1	5-NKT-Cd160
Lgals3bp	0.000476	0.4032	0.123	0.094	0.9520627	5-NKT-Cd160
Ptms	0.0001274	0.4029	0.117	0.078	0.2547153	5-NKT-Cd160
Pdcd4	0.0005116	0.3972	0.213	0.161	1	5-NKT-Cd160
Ell2	0.0075338	0.3958	0.139	0.115	1	5-NKT-Cd160
Hmgb1	0.0003575	0.3919	0.301	0.243	0.7150711	5-NKT-Cd160
Sirt3	2.51E-13	0.3870	0.101	0.042	5.03E-10	5-NKT-Cd160
**gene**	**p_val**	**avg_log2FC**	**pct.1**	**pct.2**	**p_val_adj**	**cluster**
Maf	8.23E-46	2.0319	0.42	0.162	1.65E-42	6-Treg
Ctla4	1.51E-35	2.0121	0.352	0.137	3.02E-32	6-Treg
Tnfrsf4	1.76E-34	1.9167	0.262	0.08	3.51E-31	6-Treg
Areg	2.06E-22	1.7760	0.194	0.061	4.13E-19	6-Treg
Cd4	3.87E-24	1.7689	0.248	0.073	7.74E-21	6-Treg
Nrp1	1.35E-32	1.6929	0.262	0.078	2.71E-29	6-Treg
S100a4	4.23E-47	1.6334	0.527	0.221	8.46E-44	6-Treg
Rora	1.10E-39	1.6124	0.493	0.224	2.20E-36	6-Treg
Ikzf2	3.37E-14	1.4942	0.259	0.141	6.75E-11	6-Treg
Ifi27l2a	2.06E-33	1.4850	0.558	0.304	4.11E-30	6-Treg
Icos	4.72E-22	1.3607	0.394	0.205	9.43E-19	6-Treg
S100a10	5.76E-36	1.3348	0.586	0.315	1.15E-32	6-Treg
S100a6	4.42E-47	1.3073	0.761	0.488	8.84E-44	6-Treg
Itgb1	1.54E-28	1.2164	0.355	0.148	3.08E-25	6-Treg
Il1rl1	1.17E-09	1.1979	0.132	0.052	2.33E-06	6-Treg
Izumo1r	1.28E-19	1.1125	0.132	0.018	2.56E-16	6-Treg
Cd28	5.88E-20	1.0615	0.445	0.252	1.18E-16	6-Treg
Hmgb2	5.24E-30	1.0505	0.699	0.456	1.05E-26	6-Treg
Tnfsf8	8.03E-09	1.0179	0.135	0.063	1.61E-05	6-Treg
Pdcd1	1.84E-07	1.0175	0.149	0.082	0.0003689	6-Treg
Trp53inp1	7.48E-11	0.9477	0.245	0.143	1.50E-07	6-Treg
Traf1	7.96E-11	0.9471	0.248	0.141	1.59E-07	6-Treg
Odc1	1.18E-08	0.9364	0.327	0.218	2.35E-05	6-Treg
Gprin3	0.0023338	0.9348	0.18	0.126	1	6-Treg
Tbc1d4	9.52E-06	0.9281	0.132	0.077	0.0190427	6-Treg
Bcl2a1b	1.01E-15	0.9243	0.287	0.145	2.03E-12	6-Treg
Tnfsf11	7.64E-09	0.8972	0.107	0.031	1.53E-05	6-Treg
Cpm	4.43E-12	0.8765	0.152	0.068	8.87E-09	6-Treg
Bcl2a1d	4.40E-09	0.8545	0.175	0.093	8.80E-06	6-Treg
Ramp3	4.17E-05	0.8511	0.146	0.092	0.0834368	6-Treg
Tmem64	2.22E-08	0.8311	0.248	0.155	4.43E-05	6-Treg
Bcl11b	3.53E-11	0.8106	0.335	0.21	7.05E-08	6-Treg
Gpm6b	1.32E-06	0.8053	0.118	0.061	0.0026429	6-Treg
Cd5	1.30E-06	0.8018	0.152	0.091	0.0025916	6-Treg
Lgals1	4.90E-11	0.7930	0.408	0.268	9.80E-08	6-Treg
Hopx	1.02E-08	0.7864	0.208	0.127	2.04E-05	6-Treg
Pmaip1	6.70E-11	0.7649	0.251	0.148	1.34E-07	6-Treg
Irf2bp2	1.77E-11	0.7581	0.487	0.335	3.54E-08	6-Treg

Trib2	7.25E-07	0.7288	0.163	0.097	0.0014504	6-Treg
Ly6a	4.13E-06	0.6974	0.169	0.099	0.0082588	6-Treg
Gadd45b	1.05E-05	0.6839	0.285	0.204	0.0210469	6-Treg
Cd3g	0.0001717	0.6833	0.22	0.155	0.3434819	6-Treg
Sh2d1a	1.29E-05	0.6786	0.141	0.084	0.0257686	6-Treg
Sit1	2.01E-06	0.6756	0.124	0.074	0.0040159	6-Treg
Ifrd1	2.10E-09	0.6636	0.501	0.373	4.21E-06	6-Treg
Cxcr6	3.54E-07	0.6497	0.183	0.117	0.0007086	6-Treg
Cited2	7.20E-05	0.6497	0.285	0.216	0.1440879	6-Treg
Ass1	0.0001509	0.6314	0.104	0.054	0.3017262	6-Treg
Bhlhe40	1.83E-06	0.6275	0.375	0.282	0.0036666	6-Treg
Vim	8.99E-09	0.6198	0.237	0.14	1.80E-05	6-Treg
Mki67	1.12E-08	0.5955	0.104	0.048	2.24E-05	6-Treg
Smpdl3a	2.85E-08	0.5883	0.138	0.075	5.70E-05	6-Treg
Gapdh	1.27E-09	0.5678	0.417	0.287	2.55E-06	6-Treg
Cdk6	0.0002382	0.5642	0.144	0.098	0.4764941	6-Treg
Hmgcs1	0.0003213	0.5563	0.132	0.096	0.642667	6-Treg
Hilpda	1.83E-06	0.5432	0.169	0.107	0.0036673	6-Treg
Lgals3	9.16E-05	0.5402	0.237	0.172	0.1831946	6-Treg
Hist1h1e	0.0026229	0.5377	0.127	0.096	1	6-Treg
Alcam	1.26E-12	0.5373	0.113	0.044	2.53E-09	6-Treg
Isg15	0.006142	0.5281	0.158	0.122	1	6-Treg
Cflar	6.03E-05	0.5259	0.18	0.124	0.1206215	6-Treg
S100a11	0.0003656	0.5242	0.4	0.334	0.7311932	6-Treg
Il2ra	1.17E-10	0.5091	0.115	0.041	2.34E-07	6-Treg
Pycard	0.0007495	0.5067	0.214	0.159	1	6-Treg
Nfkbiz	6.14E-06	0.4820	0.386	0.292	0.0122723	6-Treg
Sec11c	6.53E-06	0.4799	0.186	0.12	0.0130579	6-Treg
S1pr1	0.0039874	0.4779	0.301	0.239	1	6-Treg
Tox	0.0062921	0.4723	0.158	0.119	1	6-Treg
Spn	0.0004941	0.4696	0.265	0.203	0.9881367	6-Treg
1500009L16Rik	3.27E-13	0.4689	0.107	0.027	6.54E-10	6-Treg
Glrx	0.008019	0.4676	0.104	0.074	1	6-Treg
Kcnn4	4.78E-05	0.4556	0.152	0.104	0.0956856	6-Treg
Il18r1	0.001243	0.4523	0.239	0.189	1	6-Treg
Sh3bgrl	1.98E-07	0.4474	0.141	0.086	0.0003955	6-Treg
Actb	1.05E-14	0.4467	0.955	0.952	2.10E-11	6-Treg
Tmem39a	9.84E-05	0.4457	0.127	0.091	0.1968266	6-Treg
Txn1	0.0003935	0.4425	0.31	0.236	0.7870695	6-Treg
Mt1	2.10E-10	0.4400	0.169	0.093	4.20E-07	6-Treg
Reep4	1.41E-11	0.4303	0.115	0.05	2.81E-08	6-Treg
Whsc1	3.19E-07	0.4292	0.132	0.081	0.0006389	6-Treg
St6gal1	0.0035735	0.4269	0.135	0.102	1	6-Treg
Ccr2	0.0003797	0.4204	0.169	0.12	0.7594927	6-Treg
Gm21596	0.0011278	0.4150	0.189	0.139	1	6-Treg
Sub1	6.54E-06	0.4149	0.682	0.627	0.0130867	6-Treg
L1cam	0.004706	0.4145	0.101	0.071	1	6-Treg
Nfkbia	1.23E-06	0.4140	0.639	0.548	0.0024618	6-Treg
Tiam1	3.10E-12	0.4125	0.115	0.043	6.21E-09	6-Treg
Prdx1	0.0030981	0.4038	0.285	0.231	1	6-Treg
Top1	1.44E-05	0.4012	0.234	0.168	0.0288012	6-Treg
Ddit4	1.61E-08	0.3994	0.11	0.053	3.22E-05	6-Treg
Edem1	0.0020628	0.3978	0.175	0.132	1	6-Treg
Cdk2	0.0005957	0.3821	0.107	0.077	1	6-Treg
**gene**	**p_val**	**avg_log2FC**	**pct.1**	**pct.2**	**p_val_adj**	**cluster**
Igfbp4	8.97E-87	1.1743	0.795	0.115	1.79E-83	7-CD4+T-Igfbp4
St8sia6	0	0.9237	0.862	0.032	0	7-CD4+T-Igfbp4
Dapl1	8.22E-12	0.9080	0.402	0.056	1.64E-08	7-CD4+T-Igfbp4

Fam101b	5.21E-211	0.7317	0.937	0.11	1.04E-207	7-CD4+T-Igfbp4
Elovl6	4.13E-08	0.5279	0.544	0.073	8.27E-05	7-CD4+T-Igfbp4
Dusp10	3.77E-105	0.5190	0.958	0.224	7.54E-102	7-CD4+T-Igfbp4
Ncapd2	1.14E-298	0.4168	0.904	0.078	2.28E-295	7-CD4+T-Igfbp4
Prtn3	2.11E-24	0.3959	0.343	0.034	4.23E-21	7-CD4+T-Igfbp4
Ramp3	7.06E-14	0.3713	0.577	0.075	1.41E-10	7-CD4+T-Igfbp4
Mrpl18	1.67E-15	0.3649	0.368	0.06	3.34E-12	7-CD4+T-Igfbp4
Fam83d	0	0.3307	0.891	0.043	0	7-CD4+T-Igfbp4
Rnase6	6.14E-83	0.3247	0.218	0.041	1.23E-79	7-CD4+T-Igfbp4
Gng10	7.22E-10	0.3106	0.397	0.086	1.44E-06	7-CD4+T-Igfbp4
**gene**	**p_val**	**avg_log2FC**	**pct.1**	**pct.2**	**p_val_adj**	**cluster**
Cd163l1	5.15E-96	3.2755	0.536	0.072	1.03E-92	8-NKT-Zbtb16
S100a4	2.52E-68	2.7284	0.768	0.225	5.05E-65	8-NKT-Zbtb16
S100a6	1.17E-66	2.6070	0.921	0.493	2.34E-63	8-NKT-Zbtb16
Zbtb16	9.92E-12	2.1956	0.272	0.103	1.98E-08	8-NKT-Zbtb16
Actn2	5.16E-22	2.1794	0.311	0.088	1.03E-18	8-NKT-Zbtb16
Lgals1	9.03E-37	2.1132	0.662	0.267	1.81E-33	8-NKT-Zbtb16
Rora	2.64E-46	2.0682	0.682	0.229	5.28E-43	8-NKT-Zbtb16
Lmo4	7.26E-10	2.0399	0.305	0.151	1.45E-06	8-NKT-Zbtb16
S100a10	7.80E-36	1.9228	0.709	0.322	1.56E-32	8-NKT-Zbtb16
Areg	2.92E-09	1.8291	0.179	0.066	5.84E-06	8-NKT-Zbtb16
Tmem176b	2.47E-17	1.8193	0.232	0.043	4.93E-14	8-NKT-Zbtb16
Cxcr6	1.29E-21	1.7689	0.358	0.115	2.58E-18	8-NKT-Zbtb16
Cryba4	1.74E-24	1.6950	0.212	0.027	3.47E-21	8-NKT-Zbtb16
Itgae	1.50E-09	1.6401	0.219	0.09	3.01E-06	8-NKT-Zbtb16
Il17a	0.0003511	1.5394	0.152	0.082	0.7022121	8-NKT-Zbtb16
Sepp1	1.04E-15	1.5169	0.272	0.079	2.09E-12	8-NKT-Zbtb16
1500009L16Rik	5.69E-25	1.4332	0.199	0.028	1.14E-21	8-NKT-Zbtb16
Ikzf2	5.49E-10	1.4288	0.305	0.144	1.10E-06	8-NKT-Zbtb16
Avpi1	3.86E-09	1.3954	0.166	0.053	7.72E-06	8-NKT-Zbtb16
Hilpda	1.09E-08	1.3846	0.245	0.107	2.17E-05	8-NKT-Zbtb16
Icos	1.55E-13	1.3459	0.424	0.21	3.11E-10	8-NKT-Zbtb16
Vim	1.10E-12	1.3245	0.331	0.141	2.20E-09	8-NKT-Zbtb16
Pxdc1	4.50E-07	1.2923	0.113	0.024	0.0008991	8-NKT-Zbtb16
Ltb4r1	3.45E-05	1.2569	0.139	0.065	0.0689728	8-NKT-Zbtb16
Serpinb1a	4.70E-09	1.2557	0.159	0.049	9.40E-06	8-NKT-Zbtb16
Hk2	7.70E-05	1.2434	0.152	0.081	0.1540754	8-NKT-Zbtb16
Tmem64	8.21E-07	1.2312	0.278	0.158	0.0016412	8-NKT-Zbtb16
Lmna	2.17E-09	1.2271	0.205	0.073	4.34E-06	8-NKT-Zbtb16
Hsp90b1	9.43E-13	1.2175	0.583	0.348	1.89E-09	8-NKT-Zbtb16
Ccr2	3.43E-14	1.2163	0.325	0.118	6.86E-11	8-NKT-Zbtb16
Tmem176a	1.40E-09	1.2031	0.152	0.031	2.80E-06	8-NKT-Zbtb16
Rab4a	0.0017101	1.2004	0.166	0.104	1	8-NKT-Zbtb16
S100a11	2.44E-14	1.1796	0.583	0.331	4.89E-11	8-NKT-Zbtb16
Maf	1.04E-10	1.1753	0.351	0.173	2.07E-07	8-NKT-Zbtb16
Gpr183	4.84E-08	1.1702	0.252	0.12	9.68E-05	8-NKT-Zbtb16
Ckb	1.88E-10	1.0911	0.152	0.041	3.76E-07	8-NKT-Zbtb16
Il2ra	7.90E-08	1.0633	0.139	0.043	0.0001579	8-NKT-Zbtb16
Capg	7.12E-10	1.0618	0.166	0.048	1.42E-06	8-NKT-Zbtb16
Nfkbia	1.88E-15	1.0304	0.821	0.547	3.75E-12	8-NKT-Zbtb16
Ddit4	1.01E-06	1.0199	0.146	0.054	0.0020276	8-NKT-Zbtb16
Amica1	2.54E-07	1.0166	0.159	0.063	0.0005082	8-NKT-Zbtb16
Npnt	6.23E-66	1.0139	0.132	0.005	1.25E-62	8-NKT-Zbtb16
Il1r1	0.0003943	1.0028	0.119	0.052	0.7886718	8-NKT-Zbtb16
Il18r1	9.72E-10	0.9751	0.364	0.188	1.94E-06	8-NKT-Zbtb16
Tppp3	5.11E-09	0.9711	0.132	0.028	1.02E-05	8-NKT-Zbtb16
Zfp36l1	1.35E-10	0.9380	0.636	0.403	2.71E-07	8-NKT-Zbtb16
Slc15a3	6.13E-09	0.9337	0.225	0.102	1.23E-05	8-NKT-Zbtb16

Smpdl3a	1.14E-05	0.9255	0.172	0.076	0.0228179	8-NKT-Zbtb16
Fkbp2	1.65E-06	0.9181	0.152	0.067	0.0032993	8-NKT-Zbtb16
Hmgb2	1.51E-05	0.9114	0.603	0.467	0.0301833	8-NKT-Zbtb16
Aprt	5.02E-09	0.9107	0.291	0.136	1.00E-05	8-NKT-Zbtb16
Nrp1	0.0040802	0.9004	0.139	0.087	1	8-NKT-Zbtb16
Ap3s1	2.59E-05	0.8961	0.179	0.087	0.0518574	8-NKT-Zbtb16
Nme2	7.74E-06	0.8823	0.397	0.266	0.0154806	8-NKT-Zbtb16
Manf	0.0001727	0.8720	0.199	0.116	0.345327	8-NKT-Zbtb16
Cd48	9.01E-09	0.8674	0.245	0.108	1.80E-05	8-NKT-Zbtb16
Pdia6	0.0013463	0.8387	0.172	0.102	1	8-NKT-Zbtb16
Gm2a	3.70E-07	0.8366	0.245	0.114	0.0007402	8-NKT-Zbtb16
Runx2	1.34E-06	0.8112	0.278	0.158	0.0026709	8-NKT-Zbtb16
Odc1	3.74E-05	0.8054	0.331	0.222	0.0748259	8-NKT-Zbtb16
Dusp1	4.12E-10	0.7869	0.775	0.57	8.23E-07	8-NKT-Zbtb16
Gadd45b	3.98E-06	0.7817	0.338	0.205	0.007969	8-NKT-Zbtb16
Cish	3.17E-05	0.7809	0.146	0.077	0.0633824	8-NKT-Zbtb16
Rgcc	0.0001333	0.7799	0.212	0.112	0.2666768	8-NKT-Zbtb16
Spcs2	2.68E-06	0.7612	0.272	0.148	0.0053664	8-NKT-Zbtb16
Aqp3	1.70E-08	0.7596	0.113	0.027	3.40E-05	8-NKT-Zbtb16
Gpr65	5.42E-05	0.7539	0.192	0.116	0.108422	8-NKT-Zbtb16
Cmtm7	1.53E-06	0.7430	0.205	0.099	0.0030528	8-NKT-Zbtb16
Calm3	1.78E-08	0.7386	0.252	0.116	3.55E-05	8-NKT-Zbtb16
Spn	3.23E-05	0.6938	0.318	0.204	0.064667	8-NKT-Zbtb16
Ssr3	3.70E-07	0.6855	0.285	0.152	0.0007394	8-NKT-Zbtb16
Pdia4	1.14E-06	0.6838	0.219	0.111	0.0022788	8-NKT-Zbtb16
Cox5a	7.40E-05	0.6810	0.179	0.104	0.1479083	8-NKT-Zbtb16
Itgb1	8.52E-07	0.6772	0.285	0.157	0.0017048	8-NKT-Zbtb16
Nhsl2	7.62E-06	0.6745	0.119	0.052	0.0152349	8-NKT-Zbtb16
Cpm	0.0002699	0.6622	0.126	0.072	0.5398592	8-NKT-Zbtb16
Sec61a1	0.0001126	0.6470	0.146	0.085	0.2252575	8-NKT-Zbtb16
Syvn1	0.0002843	0.6450	0.146	0.082	0.5686753	8-NKT-Zbtb16
Dut	1.84E-05	0.6447	0.159	0.077	0.0367512	8-NKT-Zbtb16
Pdia3	1.37E-05	0.6363	0.278	0.158	0.0274735	8-NKT-Zbtb16
Bcl2a1d	4.46E-06	0.6358	0.179	0.096	0.0089135	8-NKT-Zbtb16
Ndufa1	6.97E-06	0.6308	0.252	0.14	0.0139479	8-NKT-Zbtb16
Sec61b	7.00E-05	0.6283	0.497	0.383	0.1400184	8-NKT-Zbtb16
Exosc8	0.0010026	0.6202	0.106	0.054	1	8-NKT-Zbtb16
Lgals3	0.0039098	0.6104	0.252	0.174	1	8-NKT-Zbtb16
Ssr4	0.0033051	0.6064	0.126	0.076	1	8-NKT-Zbtb16
Sdc4	5.53E-06	0.5983	0.119	0.055	0.0110685	8-NKT-Zbtb16
Calr	0.0002203	0.5952	0.364	0.246	0.440697	8-NKT-Zbtb16
Glrx	0.000531	0.5862	0.132	0.074	1	8-NKT-Zbtb16
Txn1	0.0008444	0.5856	0.338	0.238	1	8-NKT-Zbtb16
Ybx1	1.57E-05	0.5819	0.305	0.186	0.0313674	8-NKT-Zbtb16
Rexo2	1.34E-05	0.5807	0.166	0.089	0.0267461	8-NKT-Zbtb16
Ptpn7	7.27E-05	0.5699	0.166	0.096	0.1453717	8-NKT-Zbtb16
Id2	3.23E-06	0.5662	0.444	0.282	0.0064563	8-NKT-Zbtb16
Pgk1	1.15E-05	0.5529	0.212	0.121	0.0229491	8-NKT-Zbtb16
Bcl11b	0.0007677	0.5481	0.311	0.215	1	8-NKT-Zbtb16
Sdf2l1	6.67E-06	0.5447	0.113	0.048	0.0133446	8-NKT-Zbtb16
Sec61g	0.0011534	0.5418	0.212	0.138	1	8-NKT-Zbtb16
Ndufb2	0.0008691	0.5325	0.113	0.067	1	8-NKT-Zbtb16
Ddost	0.0003218	0.5305	0.152	0.087	0.6435107	8-NKT-Zbtb16
Ifrd1	1.43E-05	0.5299	0.536	0.377	0.0286118	8-NKT-Zbtb16
Pycard	0.0056631	0.5148	0.225	0.161	1	8-NKT-Zbtb16
Pycr2	3.67E-05	0.4718	0.146	0.077	0.0734274	8-NKT-Zbtb16
Milr1	0.005156	0.4704	0.106	0.073	1	8-NKT-Zbtb16
Anxa5	0.0035537	0.4637	0.119	0.074	1	8-NKT-Zbtb16

Cenpa	0.000232	0.4566	0.205	0.128	0.4639192	8-NKT-Zbtb16
Serp1	0.000599	0.4497	0.437	0.313	1	8-NKT-Zbtb16
Gm4184	0.0004546	0.4409	0.457	0.329	0.909133	8-NKT-Zbtb16
Tex2	0.000386	0.4396	0.113	0.066	0.7720395	8-NKT-Zbtb16
Hspa5	0.0007511	0.4137	0.563	0.437	1	8-NKT-Zbtb16
Nme1	0.0024449	0.4134	0.285	0.202	1	8-NKT-Zbtb16
Tmem123	0.0026557	0.4100	0.212	0.15	1	8-NKT-Zbtb16
Actb	6.12E-08	0.4067	0.974	0.951	0.0001225	8-NKT-Zbtb16

Supplementary Table 10

gene	p_val_adj	avg_logFC	pct.1	pct.2	p_val
Lyz2	0	1.93635845	0.931	0.073	9.29E-225
Ftl1	0	1.58310533	0.981	0.189	2.56E-136
Jchain	0	1.48915518	0.951	0.148	9.92E-275
Rps5	0	1.4714686	0.977	0.334	2.44E-235
LOC100862·	0	1.44982311	0.948	0.177	9.25E-278
Apoe	0	1.42622038	0.744	0.055	8.18E-175
Ifitm2	0	1.29290945	0.921	0.169	3.38E-270
Rps6	0	1.28026005	0.871	0.169	8.05E-293
Rps2	6.06E-294	1.27760277	0.962	0.352	8.12E-290
Ifitm3	0	1.21229957	0.893	0.149	1.52E-272
Sh3bgrl3	1.46475085:	1.19542639	0.95	0.256	1.96E-306
Alox5ap	0	1.18641783	0.792	0.095	6.75E-264
LOC105244:	0	1.1699028	0.995	0.636	7.14E-194
Fcer1g	4.86E-298	1.16480351	0.951	0.272	6.51E-294
Lgals3	0	1.10063695	0.765	0.105	1.43E-291
Ccl5	2.17E-118	1.08277236	0.993	0.696	2.90E-114
Rplp0	0.00E+00	1.06922216	0.992	0.666	1.93E-305
Ubb	1.51039042	1.03920335	1	0.583	2.02E-306
Lgals1	2.86E-263	1.03332684	0.815	0.208	3.83E-259
Ppib	0	1.02078359	0.6	0.061	1.58E-201
Elane	1.63E-246	1.01875801	0.369	0.008	2.18E-242
Cst3	0	1.01050365	0.912	0.195	9.78E-255
Slpi	0	1.00618553	0.754	0.117	2.21E-215
Ccl6	0	0.96811199	0.72	0.103	2.47E-240
Manf	0	0.96244569	0.566	0.057	1.06E-289
Ccl9	7.00E-242	0.95609561	0.393	0.02	9.36E-238
Plac8	2.22E-276	0.93122547	0.924	0.28	2.97E-272
Ly6d	0	0.92256871	0.657	0.094	1.06E-211
Gm6745	1.16E-272	0.92255549	0.459	0.034	1.55E-268
Ngp	0	0.9216069	0.731	0.122	2.45E-179
Camp	2.52E-304	0.90340568	0.524	0.052	3.38E-300
Prtn3	7.62E-220	0.90035408	0.325	0.007	1.02E-215
Emd	0	0.90026409	0.933	0.263	8.64E-188
Ly6c2	1.71E-298	0.90005277	0.808	0.194	2.28E-294
Gpx1	0	0.90003582	0.815	0.172	7.00E-233
Uba52	0	0.8850893	1	0.877	5.33E-247
Rpl14	3.01E-260	0.88268061	0.999	0.675	4.03E-256
Mzb1	8.24E-269	0.88247604	0.454	0.037	1.10E-264
15-Sep	6.63E-270	0.8805941	0.448	0.035	8.87E-266
Rps18	6.70E-219	0.87639735	0.995	0.731	8.97E-215
Ifitm1	0	0.87224832	0.8	0.161	1.13E-242
Gm13202	0	0.84854424	0.683	0.111	3.73E-299
Tspo	0	0.83321415	0.853	0.211	1.46E-245
Btf3	8.78E-271	0.81756758	0.902	0.285	1.18E-266
D8Ertd738e	0	0.81503954	0.781	0.165	1.22E-150
Cd14	1.07E-305	0.79381024	0.527	0.063	1.43E-301
Chil3	1.02E-240	0.79252539	0.395	0.032	1.37E-236
Ctsh	8.85E-219	0.78581679	0.381	0.031	1.18E-214
Rpl19	8.39E-226	0.78403029	0.999	0.704	1.12E-221
Fth1	9.47E-271	0.78084695	1	0.78	1.27E-266
Rpl29	2.88E-189	0.776981	0.97	0.453	3.85E-185
H2-Ab1	2.83E-304	0.76702087	0.895	0.263	3.79E-300
Ctsz	2.43E-303	0.76679751	0.527	0.068	3.25E-299
Gm8210	7.04E-164	0.76368085	0.245	0.002	9.43E-160
Rps11	8.38E-181	0.76365909	0.993	0.642	1.12E-176

Gm25380	2.86E-172	0.76249741	0.984	0.596	3.82E-168
Cyba	3.54E-302	0.75821367	0.909	0.283	4.74E-298
Brk1	1.02E-257	0.75735203	0.45	0.05	1.36E-253
Ctss	3.05E-306	0.7573155	0.595	0.097	4.08E-302
Cstb	6.76E-264	0.7526581	0.468	0.057	9.05E-260
Cd52	7.88E-184	0.75195365	0.963	0.448	1.06E-179
Sepw1	0	0.74846487	0.867	0.231	2.12E-59
Rpl15	1.10E-177	0.73807473	0.992	0.652	1.47E-173
Atp5d	1.34505592	0.72816828	0.616	0.108	1.80e-312
Ppp1ca	1.06E-306	0.72722163	0.585	0.098	1.42E-302
Hint1	0	0.7172024	0.818	0.196	5.22E-146
Rpl21	1.26E-164	0.71312303	0.982	0.533	1.68E-160
Vamp8	2.00330427	0.70686023	0.6	0.104	2.68E-307
Cdk2ap2	0	0.70431836	0.74	0.151	1.78E-262
Rps10	1.05E-239	0.70206023	0.905	0.331	1.40E-235
Msrb1	0	0.6963378	0.773	0.177	1.12E-211
Gm4705	0	0.69138283	0.82	0.221	9.14E-300
Nkg7	7.85E-129	0.68721044	0.789	0.354	1.05E-124
Cd74	3.22E-194	0.68422959	0.993	0.499	4.31E-190
Myl12a	0	0.68388358	0.729	0.166	1.24E-273
S100a10	0	0.68353991	0.879	0.257	1.08E-92
Eno1	1.43E-182	0.68322083	0.347	0.038	1.92E-178
Eif5a	0	0.6799358	0.737	0.176	1.15E-285
Rpl12	1.62E-179	0.67852691	0.302	0.022	2.17E-175
Psmb3	1.43E-167	0.67447071	0.273	0.014	1.91E-163
Rpl32	1.81E-171	0.67239022	0.993	0.848	2.42E-167
Nme2	9.00E-298	0.67008415	0.762	0.202	1.20E-293
Ldha	4.16E-308	0.66464306	0.581	0.102	5.57E-304
Myl12b	0	0.66252894	0.604	0.108	3.97E-203
Rpl13	4.60E-183	0.66194846	1	0.912	6.16E-179
Sdf2l1	4.36E-143	0.6603874	0.259	0.019	5.84E-139
Ppia	7.66E-161	0.6588937	0.995	0.617	1.03E-156
Psmb8	0	0.65730691	0.684	0.143	3.64E-173
Fam96a	7.14E-186	0.65639117	0.335	0.034	9.56E-182
Arpc1b	1.47E-307	0.65610086	0.691	0.163	1.96E-303
Rps14	2.83E-160	0.65335777	0.997	0.75	3.79E-156
Edf1	1.07E-270	0.651879	0.484	0.074	1.43E-266
Cnbp	0	0.65078479	0.739	0.178	3.14E-284
Klk8	1.20E-184	0.64628237	0.333	0.035	1.60E-180
Vim	1.73E-275	0.646162	0.528	0.092	2.32E-271
Mgst1	4.32E-160	0.64574771	0.273	0.019	5.78E-156
Rpl6	1.39E-144	0.641627	0.984	0.591	1.86E-140
LOC102635	1.06E-285	0.64037128	0.761	0.216	1.42E-281
Eif1	6.44E-152	0.63810688	0.993	0.621	8.61E-148
H2-Aa	0	0.63552861	0.733	0.159	1.62E-215
Arhgdib	0	0.63194199	0.869	0.263	1.75E-159
Tyrobp	1.20E-212	0.62504835	0.917	0.384	1.60E-208
Prdx1	0	0.62490367	0.725	0.166	3.61E-253
Hp	0	0.6244893	0.665	0.147	2.35E-235
Aldoa	9.18E-273	0.62006	0.513	0.088	1.23E-268
Npc2	5.26E-280	0.6151698	0.844	0.277	7.04E-276
Eef1d	0	0.61485181	0.623	0.121	5.50E-288
Ssr4	6.48E-193	0.61329106	0.337	0.036	8.67E-189
Pfn1	5.43E-143	0.61185182	0.973	0.543	7.26E-139
Ctsg	7.35E-108	0.61065994	0.17	0.002	9.83E-104
Ptprcap	2.18E-277	0.60786972	0.624	0.148	2.92E-273
Rpl17	3.93E-135	0.60423343	0.996	0.78	5.26E-131
Emp3	7.14E-261	0.60261415	0.49	0.084	9.55E-257

Rpl18a	1.77E-280	0.60251548	0.645	0.159	2.37E-276
Atp6v1f	0	0.59265964	0.659	0.135	8.53E-271
Cd3d	8.96E-263	0.59069774	0.679	0.191	1.20E-258
Park7	3.36E-291	0.58932571	0.543	0.099	4.50E-287
Rps3	5.84E-141	0.58532322	0.999	0.774	7.82E-137
Fis1	1.17142964(	0.58169732	0.59	0.116	1.56e-316
LOC100862	2.09E-144	0.58135459	0.999	0.851	2.79E-140
Uqcrfs1	6.62E-273	0.5811076	0.484	0.077	8.86E-269
Cycs	2.87918402	0.58076786	0.627	0.14	3.85E-307
Rgs10	2.18E-216	0.58052012	0.396	0.059	2.92E-212
Rpl18	1.40E-125	0.58032938	0.986	0.647	1.88E-121
Timm23	1.34E-182	0.57773216	0.324	0.038	1.79E-178
Gngt2	1.53E-234	0.57729787	0.389	0.047	2.05E-230
Eno1b	2.29E-111	0.57591489	0.185	0.006	3.06E-107
Nedd8	4.98E-278	0.57428467	0.524	0.098	6.67E-274
Lrrc58	4.37E-193	0.57423097	0.401	0.073	5.86E-189
Rpl5	1.13E-264	0.57065166	0.782	0.251	1.51E-260
Gm5621	4.00105363	0.56999837	0.552	0.102	5.36E-306
Itm2b	0	0.56854939	0.867	0.266	9.98E-260
Retnlg	1.06E-197	0.56747063	0.98	0.472	1.42E-193
Gpx4	1.23E-293	0.56737132	0.548	0.104	1.65E-289
Sumo2	2.66687742	0.5641379	0.623	0.135	3.568e-314
Gng10	2.84E-203	0.56332662	0.366	0.053	3.80E-199
Grpel1	2.08E-124	0.56293068	0.223	0.017	2.78E-120
Pycard	4.09E-299	0.55896054	0.555	0.107	5.48E-295
Ifi30	1.12E-143	0.55599966	0.256	0.024	1.50E-139
Tmsb4x	1.22E-192	0.55579461	1	0.998	1.63E-188
Mien1	2.12E-207	0.55550519	0.377	0.057	2.83E-203
Erh	1.38E-299	0.55464821	0.555	0.108	1.84E-295
Atp5c1	3.39E-150	0.55333608	0.267	0.025	4.54E-146
Pfdn5	1.10E-279	0.55262755	0.897	0.329	1.47E-275
Gabarap	0	0.55152372	0.684	0.165	1.65E-284
Supt4b	5.75E-180	0.54772373	0.32	0.04	7.70E-176
Myl6	5.06E-260	0.54636287	0.818	0.284	6.78E-256
Psmb2	8.20E-193	0.54499738	0.337	0.042	1.10E-188
Cox5a	1.87E-222	0.54387534	0.401	0.063	2.51E-218
Rnase6	3.60E-128	0.54385212	0.23	0.019	4.81E-124
Dynlrb1	3.29E-242	0.54105037	0.439	0.072	4.41E-238
Gm10045	4.82E-119	0.53866214	0.985	0.635	6.45E-115
Slc25a5	4.72E-295	0.5369119	0.63	0.154	6.32E-291
Serf2	9.94E-182	0.53678091	0.977	0.495	1.33E-177
Supt4a	2.12468281	0.53666793	0.635	0.15	2.84E-307
Lamtor5	3.14E-171	0.53573984	0.309	0.039	4.20E-167
Rac2	0.00E+00	0.53565995	0.841	0.276	3.76E-305
Oaz1	3.45E-174	0.53457445	0.981	0.509	4.62E-170
Sri	8.48E-225	0.5333762	0.412	0.071	1.13E-220
Coa3	6.48E-155	0.53186661	0.278	0.03	8.68E-151
Clta	0	0.52860361	0.698	0.182	2.13E-285
G0s2	0	0.52773314	0.739	0.184	3.09E-133
Taldo1	2.38E-233	0.52671994	0.422	0.071	3.18E-229
Ssr2	3.32E-130	0.52579864	0.234	0.021	4.44E-126
Eif3h	0	0.52477459	0.705	0.176	1.98E-277
Ramp1	5.05E-140	0.52388757	0.246	0.024	6.76E-136
Cox6b1	0	0.52205625	0.668	0.163	3.94E-229
Dad1	0	0.52127405	0.665	0.16	3.40E-276
Rpl10a	3.44E-107	0.51951605	0.969	0.622	4.60E-103
Stmn1	9.65E-223	0.51865	0.386	0.058	1.29E-218
Spcs1	6.58E-271	0.51712395	0.468	0.081	8.81E-267

Prelid1	1.13E-187	0.51400856	0.347	0.052	1.52E-183
Atp5f1	3.48E-287	0.5133289	0.547	0.115	4.66E-283
Selk	0	0.51205126	0.752	0.201	1.25E-84
Rpl7	2.21E-120	0.51166925	0.966	0.577	2.96E-116
Ostf1	1.95E-294	0.51026479	0.559	0.121	2.61E-290
H2-DMa	3.57E-176	0.50739848	0.309	0.039	4.78E-172
Ccdc12	8.60E-258	0.50595189	0.487	0.096	1.15E-253
Sap18b	3.27E-246	0.50163235	0.473	0.092	4.38E-242
Med28	7.31E-205	0.50080705	0.374	0.062	9.79E-201
Prdx2	2.10E-207	0.50058176	0.371	0.059	2.80E-203
Cd3g	4.35E-229	0.49819571	0.469	0.1	5.82E-225
Safb	4.61E-87	-0.50038313	0.131	0.106	6.18E-83
Pura	1.48E-99	-0.50195547	0.137	0.105	1.98E-95
Rtf1	2.71E-144	-0.50272621	0.222	0.156	3.62E-140
Serinc1	1.17E-130	-0.50449115	0.182	0.131	1.56E-126
Gpcpd1	7.25E-136	-0.50481248	0.195	0.137	9.70E-132
Pum1	2.52E-91	-0.50501236	0.128	0.107	3.38E-87
Vps4b	1.81E-101	-0.50504041	0.137	0.106	2.42E-97
Smg1	5.76E-80	-0.50513753	0.11	0.095	7.71E-76
Lmnb1	2.60E-145	-0.50561729	0.22	0.151	3.48E-141
Phf21a	1.75E-85	-0.50652845	0.12	0.098	2.34E-81
Pafah1b1	4.03E-140	-0.50762096	0.21	0.143	5.40E-136
Pnn	1.59E-83	-0.50887845	0.127	0.11	2.12E-79
Ankrd44	7.10E-105	-0.5105077	0.165	0.126	9.51E-101
Abi1	2.89E-136	-0.51149473	0.189	0.133	3.87E-132
Map4k4	2.63E-98	-0.51184365	0.133	0.11	3.52E-94
Bcl11b	9.70E-109	-0.51185982	0.192	0.146	1.30E-104
Kmt2e	1.94E-143	-0.5128231	0.211	0.154	2.60E-139
Sh2d2a	4.56E-132	-0.51382601	0.248	0.179	6.11E-128
Gm21811	2.63E-104	-0.51395108	0.159	0.126	3.53E-100
Wdr43	7.33E-77	-0.5153617	0.109	0.099	9.82E-73
Runx1	4.01E-80	-0.5179056	0.125	0.106	5.37E-76
Nfatc1	3.77E-83	-0.51822567	0.132	0.11	5.05E-79
Il18rap	2.44E-66	-0.51917143	0.118	0.106	3.27E-62
Hnrnpul2	1.66E-145	-0.51983626	0.204	0.143	2.23E-141
Lrrfip1	4.64E-145	-0.52130566	0.212	0.15	6.21E-141
Nipbl	3.82E-112	-0.52255592	0.166	0.127	5.11E-108
G3bp1	2.75E-165	-0.52589046	0.254	0.172	3.68E-161
Sf3b1	2.12E-208	-0.52814181	0.344	0.239	2.84E-204
Serpinb6b	9.96E-93	-0.52843973	0.234	0.182	1.33E-88
Tnrc6a	1.37E-92	-0.52876272	0.143	0.121	1.84E-88
Icam1	2.33E-80	-0.52952515	0.132	0.105	3.11E-76
Pcmtd1	5.16E-70	-0.52964379	0.105	0.096	6.91E-66
Cyld	1.15E-71	-0.53027439	0.107	0.104	1.54E-67
Prex1	1.77E-105	-0.53108413	0.156	0.124	2.37E-101
Elovl5	1.45E-116	-0.53120153	0.173	0.13	1.94E-112
Tmem30a	4.77E-125	-0.53131725	0.173	0.13	6.39E-121
Elavl1	2.66E-139	-0.53156429	0.21	0.156	3.56E-135
Ppp6r1	3.25E-95	-0.53218709	0.128	0.107	4.36E-91
Gna13	2.41E-184	-0.53272341	0.275	0.188	3.22E-180
Tardbp	3.21E-100	-0.53370133	0.151	0.127	4.30E-96
Hdc	9.11E-188	-0.53489772	0.265	0.168	1.22E-183
Gatad2b	2.26E-78	-0.53508198	0.113	0.102	3.03E-74
Pitpnc1	1.86E-133	-0.53678036	0.205	0.153	2.49E-129
Chd7	2.73E-149	-0.53777472	0.224	0.159	3.65E-145
Susd6	2.15E-73	-0.53841446	0.103	0.096	2.88E-69
Rbm25	5.79E-148	-0.53911467	0.21	0.153	7.74E-144
Eif2s2	8.32E-265	-0.53915204	0.48	0.334	1.11E-260

Epb41	6.51E-81	-0.54228924	0.11	0.099	8.72E-77
Gem	5.40E-47	-0.54306425	0.106	0.102	7.23E-43
Mapk1	7.84E-146	-0.54367226	0.214	0.159	1.05E-141
Srrm2	1.70E-238	-0.54415394	0.414	0.282	2.28E-234
Trim30a	1.03E-120	-0.54441757	0.181	0.137	1.38E-116
Wdr26	1.89E-130	-0.54531777	0.199	0.15	2.53E-126
Kmt2a	1.50E-78	-0.54602507	0.106	0.101	2.00E-74
Fryl	3.45E-84	-0.54644552	0.133	0.119	4.62E-80
Aplp2	6.93E-71	-0.54909819	0.107	0.102	9.28E-67
Ist1	5.32E-121	-0.55084606	0.165	0.125	7.13E-117
Fyn	6.58E-153	-0.55228675	0.233	0.17	8.80E-149
Nfatc3	2.71E-105	-0.55310226	0.146	0.118	3.63E-101
Tab2	2.52E-113	-0.55387718	0.155	0.124	3.38E-109
Cmah	1.55E-116	-0.55479358	0.158	0.124	2.08E-112
Odc1	2.15E-139	-0.55675966	0.241	0.167	2.88E-135
Rgs2	1.18E-218	-0.55707164	0.495	0.334	1.58E-214
Zbtb20	1.08E-112	-0.55805921	0.17	0.132	1.44E-108
Ddx3x	1.72E-163	-0.55810346	0.25	0.188	2.30E-159
Tagap	4.77E-99	-0.55909786	0.158	0.128	6.39E-95
Icos	8.98E-93	-0.55981301	0.174	0.132	1.20E-88
Skil	3.65E-90	-0.5603836	0.137	0.118	4.88E-86
Etnk1	8.83E-76	-0.56239384	0.102	0.099	1.18E-71
Ppp1r16b	6.86E-73	-0.56358769	0.105	0.1	9.18E-69
Dgat1	7.79E-180	-0.5636357	0.286	0.207	1.04E-175
Ikzf1	7.53E-253	-0.56552848	0.376	0.251	1.01E-248
Hivep2	5.88E-112	-0.56694149	0.169	0.14	7.87E-108
Sf1	1.13E-158	-0.56829276	0.235	0.178	1.52E-154
Ccdc88c	4.29E-105	-0.5711882	0.15	0.129	5.74E-101
Pik3r1	3.29E-119	-0.57270735	0.227	0.181	4.40E-115
Ewsr1	2.19E-175	-0.57544202	0.254	0.183	2.93E-171
Son	1.08E-268	-0.57645775	0.431	0.288	1.45E-264
Fam65b	1.28E-132	-0.57769	0.201	0.159	1.72E-128
Hexim1	4.23E-139	-0.57818364	0.222	0.172	5.66E-135
Spry2	6.83E-42	-0.57831852	0.099	0.116	9.14E-38
Mt-mt-Rnr2	1.18E-209	-0.57890839	0.914	0.753	1.58E-205
Kdm5a	5.79E-80	-0.57907902	0.118	0.109	7.75E-76
Tcf7	6.83E-196	-0.57971655	0.303	0.208	9.14E-192
Zdhhc18	4.55E-101	-0.58172107	0.143	0.124	6.09E-97
Tpt1	2.46E-133	-0.58208609	0.993	0.96	3.29E-129
Cd164	2.28E-187	-0.58213055	0.265	0.197	3.06E-183
Msl2	7.20E-91	-0.58241448	0.122	0.111	9.64E-87
St8sia4	5.80E-104	-0.58485747	0.158	0.136	7.76E-100
Sfpq	2.69E-191	-0.58554031	0.269	0.19	3.59E-187
Phf20l1	2.43E-97	-0.58705699	0.147	0.132	3.25E-93
Strn3	2.70E-67	-0.58738002	0.102	0.103	3.61E-63
Ddx17	1.95E-154	-0.58893406	0.222	0.169	2.61E-150
Tgif1	1.36E-167	-0.58930591	0.26	0.195	1.82E-163
Il18r1	1.30E-83	-0.59221498	0.14	0.126	1.73E-79
Arap2	1.87E-65	-0.59254179	0.094	0.102	2.50E-61
Stk38	2.80E-134	-0.59342381	0.185	0.146	3.75E-130
Hsph1	4.99E-180	-0.59347619	0.283	0.197	6.68E-176
Eif5b	1.05E-140	-0.59484243	0.218	0.175	1.41E-136
Ccnd2	3.98E-206	-0.59530728	0.378	0.275	5.32E-202
Clint1	1.87E-146	-0.59546638	0.192	0.152	2.50E-142
Csrnp1	2.02E-161	-0.59731688	0.238	0.173	2.71E-157
Ddit3	3.51E-204	-0.59765257	0.295	0.208	4.70E-200
Ppig	2.79E-109	-0.59811207	0.185	0.158	3.74E-105
Hp1bp3	9.45E-125	-0.6005678	0.169	0.139	1.26E-120

Map3k1	1.79E-122	-0.6013718	0.181	0.153	2.39E-118
Junb	1.62E-181	-0.60140691	0.956	0.843	2.16E-177
Tgoln1	7.86E-174	-0.60248335	0.257	0.192	1.05E-169
Ankrd12	1.02E-73	-0.60327323	0.121	0.114	1.36E-69
Tra2a	1.81E-129	-0.60427723	0.181	0.147	2.43E-125
Myh9	6.66E-198	-0.60796473	0.269	0.197	8.91E-194
Cdk17	2.36E-78	-0.60893614	0.11	0.11	3.15E-74
Arih1	4.84E-124	-0.60896689	0.178	0.148	6.48E-120
Fnbp1	7.22E-106	-0.60958918	0.133	0.118	9.66E-102
Ppp3ca	2.55E-106	-0.61019233	0.159	0.134	3.41E-102
D16Ertd472	2.02E-162	-0.61114922	0.261	0.198	2.71E-158
Pum2	2.11E-172	-0.61146156	0.227	0.174	2.83E-168
Elf1	4.38E-260	-0.61605782	0.399	0.272	5.86E-256
Nufip2	3.02E-133	-0.6172509	0.182	0.155	4.04E-129
Nr3c1	3.95E-136	-0.6175	0.208	0.167	5.28E-132
S1pr1	3.56E-148	-0.6184032	0.238	0.173	4.76E-144
Rsrc2	7.42E-165	-0.61946194	0.248	0.195	9.93E-161
Hipk1	2.47E-146	-0.62105	0.204	0.167	3.31E-142
Runx3	3.69E-165	-0.62167495	0.261	0.2	4.94E-161
Cd44	1.76E-259	-0.62332981	0.429	0.303	2.35E-255
Nmt1	9.36E-78	-0.6237927	0.11	0.116	1.25E-73
Vps37b	6.76E-228	-0.62392804	0.788	0.666	9.05E-224
Sp110	1.83E-130	-0.62526767	0.17	0.143	2.46E-126
Zc3hav1	2.77E-248	-0.62589461	0.376	0.268	3.71E-244
Pde7a	1.99E-119	-0.62593919	0.181	0.16	2.66E-115
Matr3	4.17E-140	-0.62614757	0.19	0.16	5.58E-136
Ssh2	5.19E-222	-0.62751441	0.32	0.234	6.95E-218
Wsb1	2.13E-125	-0.62778127	0.169	0.144	2.85E-121
Jak1	2.89E-283	-0.62814591	0.502	0.358	3.87E-279
Foxp1	2.41E-208	-0.62998669	0.377	0.282	3.23E-204
Hnrnpdl	2.78E-201	-0.630596	0.295	0.223	3.72E-197
Dusp10	2.96E-138	-0.63070121	0.216	0.167	3.96E-134
Stk4	6.20E-143	-0.63262332	0.207	0.172	8.30E-139
Rassf5	2.02E-117	-0.63323942	0.155	0.138	2.70E-113
Clec2d	5.42E-91	-0.63548029	0.121	0.121	7.26E-87
Ncl	2.37E-274	-0.63623723	0.476	0.348	3.17E-270
Eif4g2	1.27E-283	-0.63676962	0.46	0.337	1.71E-279
Samsn1	1.29E-121	-0.63814529	0.205	0.163	1.72E-117
Srsf6	2.10E-175	-0.63893644	0.248	0.193	2.82E-171
Cmip	2.22E-70	-0.64226785	0.103	0.113	2.98E-66
Ogt	1.64E-96	-0.64388993	0.124	0.123	2.20E-92
Dock10	7.15E-144	-0.64396324	0.197	0.168	9.57E-140
Tnrc6b	4.04E-102	-0.64568014	0.155	0.145	5.40E-98
4932438A13	3.57E-113	-0.65124618	0.154	0.141	4.77E-109
Atp2b1	2.99E-156	-0.65165023	0.249	0.202	4.01E-152
Ddx3y	3.91E-134	-0.65947678	0.208	0.182	5.23E-130
Dhx15	1.73E-114	-0.66389848	0.151	0.14	2.31E-110
Tle4	9.13E-93	-0.67064897	0.136	0.135	1.22E-88
Purb	2.60E-133	-0.67080401	0.174	0.147	3.49E-129
Ugcg	1.46E-174	-0.67189261	0.264	0.204	1.95E-170
Csnk1a1	4.07E-208	-0.67198614	0.286	0.224	5.45E-204
Mgea5	4.09E-105	-0.67632402	0.139	0.143	5.48E-101
Pnisr	1.22E-139	-0.67755587	0.188	0.168	1.64E-135
Cd28	3.53E-153	-0.67945383	0.23	0.188	4.72E-149
Gramd3	1.22E-214	-0.67985614	0.369	0.289	1.63E-210
Aebp2	1.27E-91	-0.68020205	0.121	0.129	1.70E-87
Serpinb9	1.30E-101	-0.68058106	0.216	0.187	1.74E-97
Rnf125	4.36E-214	-0.68062749	0.344	0.274	5.83E-210

Ptbp3	1.22E-226	-0.68067824	0.332	0.258	1.64E-222
Nfkbiz	5.28E-217	-0.68163783	0.348	0.248	7.07E-213
Snrnp70	1.50E-205	-0.68389087	0.283	0.227	2.01E-201
Kdm2b	1.40E-61	-0.68960026	0.082	0.105	1.88E-57
Il4ra	1.73E-169	-0.69020196	0.239	0.2	2.32E-165
Ifrd1	8.36E-217	-0.70202201	0.408	0.326	1.12E-212
Gpr132	1.23E-247	-0.7020611	0.396	0.312	1.64E-243
Lbr	3.47E-187	-0.70234567	0.248	0.209	4.65E-183
Heca	4.94E-123	-0.70944699	0.166	0.158	6.62E-119
Atrx	4.13E-110	-0.71416119	0.155	0.158	5.53E-106
Tnks2	1.11E-100	-0.71422701	0.129	0.141	1.49E-96
Crebrf	3.34E-109	-0.71701839	0.133	0.139	4.48E-105
Rsrp1	1.26E-243	-0.71731353	0.352	0.272	1.69E-239
Polr2a	2.17E-150	-0.71950943	0.188	0.168	2.91E-146
Esyt2	2.24E-144	-0.72139505	0.189	0.177	3.00E-140
Eif3j2	2.29E-103	-0.72355737	0.128	0.144	3.07E-99
Nrip1	6.07E-92	-0.72457251	0.147	0.152	8.12E-88
Il2rb	3.27E-221	-0.726075	0.441	0.363	4.38E-217
Sp100	1.47E-193	-0.73331356	0.272	0.233	1.97E-189
Nabp1	1.11E-135	-0.73374541	0.249	0.231	1.48E-131
Zc3h13	1.65E-65	-0.74461828	0.078	0.113	2.21E-61
Snx18	1.09E-162	-0.75080957	0.227	0.203	1.46E-158
Hnrnph1	1.57E-255	-0.75492131	0.354	0.284	2.10E-251
Celf1	7.29E-136	-0.75633005	0.177	0.176	9.76E-132
Atp1b3	0	-0.75710939	0.565	0.426	9.63E-279
Ythdc1	2.92E-172	-0.76032997	0.235	0.212	3.91E-168
Clk1	2.25E-262	-0.76054029	0.399	0.33	3.01E-258
Atf4	2.05E-273	-0.76133372	0.448	0.361	2.75E-269
Dusp1	1.44E-286	-0.76190457	0.679	0.523	1.93E-282
Hnrnpm	5.42E-236	-0.76430606	0.329	0.286	7.25E-232
Rab8b	5.23E-136	-0.76468652	0.184	0.18	7.00E-132
Ptprc	0	-0.77089316	0.703	0.566	4.79E-196
Ddx21	2.12E-180	-0.77135613	0.246	0.223	2.84E-176
Nr4a3	9.35E-118	-0.77582311	0.18	0.177	1.25E-113
Kdm7a	2.35E-151	-0.78050897	0.204	0.199	3.14E-147
Ahnak	3.96E-233	-0.78739283	0.38	0.311	5.30E-229
Eif3a	3.84E-197	-0.78768633	0.26	0.239	5.14E-193
Ankrd11	2.86E-157	-0.790447	0.215	0.203	3.82E-153
Fus	1.20E-179	-0.79520883	0.254	0.238	1.61E-175
Luc7l2	2.18E-303	-0.7985638	0.405	0.323	2.92E-299
Pcbp2	4.14402273	-0.80330495	0.525	0.432	5.547e-311
Prpf38b	2.77E-188	-0.80420422	0.234	0.217	3.71E-184
Nr4a1	3.93E-254	-0.80896355	0.437	0.343	5.26E-250
Gls	3.65E-143	-0.81944435	0.189	0.204	4.88E-139
Pabpc4	5.47E-84	-0.82821427	0.093	0.132	7.33E-80
Hnrnpa0	4.42E-217	-0.82937736	0.291	0.271	5.91E-213
Mbnl1	6.28E-283	-0.83643857	0.497	0.446	8.41E-279
Rdm1	2.84E-148	-0.83974192	0.331	0.319	3.80E-144
Akna	3.87E-130	-0.84098572	0.162	0.186	5.17E-126
Tob2	8.69E-107	-0.84683994	0.155	0.178	1.16E-102
Tcp11l2	3.65E-177	-0.85836921	0.216	0.215	4.89E-173
Itgal	6.03E-253	-0.86154938	0.405	0.357	8.08E-249
Cnot6l	9.12E-161	-0.86287948	0.19	0.206	1.22E-156
Prrc2c	1.02E-163	-0.86364942	0.22	0.233	1.36E-159
Bcl2l11	1.73E-180	-0.86365263	0.32	0.301	2.32E-176
Slc38a2	1.86E-232	-0.86460626	0.371	0.343	2.49E-228
Zfp36l2	9.77E-267	-0.86589063	0.627	0.542	1.31E-262
Klf6	2.16E-278	-0.86680729	0.595	0.506	2.89E-274

Zcchc11	3.18E-161	-0.86755794	0.222	0.245	4.26E-157
Prpf4b	5.74E-150	-0.8734969	0.21	0.235	7.68E-146
Rora	2.89E-73	-0.8870733	0.122	0.158	3.87E-69
Amd1	8.45E-108	-0.89398143	0.147	0.183	1.13E-103
Ccnl1	6.44E-230	-0.89750893	0.307	0.294	8.62E-226
Ddx6	1.44E-232	-0.91543366	0.294	0.291	1.93E-228
Btg1	3.82E-298	-0.91660402	0.969	0.93	5.11E-294
Ddx5	0	-0.91944403	0.878	0.821	2.54E-186
Cxcr4	5.38E-236	-0.92150166	0.469	0.43	7.20E-232
Bhlhe40	1.57E-132	-0.92352951	0.207	0.215	2.10E-128
Ets1	0	-0.93455281	0.728	0.65	3.78E-259
Stk17b	0	-0.93841393	0.709	0.64	4.30E-232
Tra2b	7.34724478	-0.94481952	0.555	0.513	9.83e-313
Nr4a2	3.59E-99	-0.96284181	0.165	0.196	4.80E-95
P2ry10	1.79E-222	-0.96459837	0.327	0.334	2.40E-218
Zfp36l1	6.78E-220	-0.96807625	0.329	0.333	9.08E-216
Irf2bp2	2.05E-175	-0.99305185	0.238	0.265	2.75E-171
Satb1	1.40E-277	-1.00136675	0.556	0.539	1.88E-273
Dennd4a	3.70E-227	-1.00462231	0.373	0.373	4.96E-223
Celf2	4.66E-224	-1.02774583	0.309	0.34	6.24E-220
Peli1	6.40E-249	-1.03382055	0.359	0.37	8.57E-245
Mt-mt-Rnr1	2.12E-125	-1.0659438	0.151	0.211	2.84E-121
Fosl2	1.60E-285	-1.08309024	0.472	0.466	2.14E-281
Rps28	2.13E-89	-1.12476271	0.075	0.184	2.86E-85
Hspa1b	1.80E-113	-1.15704518	0.348	0.346	2.41E-109
Tnfaip3	7.04E-307	-1.25840922	0.548	0.623	9.43E-303
Hbb-bs	6.98E-38	-2.03918307	0	0.115	9.35E-34

Supplementary Table 9 | Basic information on the human liver microarray data.

Data	Source	Series	Samples	reference
**human liver microarray**	NCBI Gene Expression Omnibus	GSE151158	21healthy, 23 NAFLD(NAS*<=3),17 NAFLD(NAS>5) patients	Michael et al ,2020
**human liver microarray**	NCBI Gene Expression Omnibus	GSE63067	7 healthy, 2 NAFLD, 9 NASH patients	Itziar et al ,2015

*NAS: NAFLD Activity Score
